# Supplementary material for: Transmission of SARS-CoV-2 in free-ranging white-tailed deer in the United States
Source: Nat Commun. 2023 Jul 10;14:4078. doi: 10.1038/s41467-023-39782-x (PMC10333304; doi:10.1038/s41467-023-39782-x)
Supplement: Supplementary file 1 — Supplementary Information [file 41467_2023_39782_MOESM1_ESM.pdf]

Supplementary Information

Transmission of SARS-CoV-2 in free-ranging white-tailed deer in the United States

Authors: Aijing Feng[1,2,3], Sarah Bevins[4], Jeff Chandler[5], Thomas J. DeLiberto[6*], Ria Ghai[7], Kristina Lantz[8], Julianna Lenoch[4], Adam Retchless[9], Susan Shriner[5], Cynthia Y. Tang[1,3,10], Suxiang Sue Tong[9], Mia Torchetti[8], Anna Uehara[9], Xiu-Feng Wan[1,2,3,10,11*]

[1]Center for Influenza and Emerging Infectious Diseases, University of Missouri, Columbia, MO, USA;
[2]Department of Molecular Microbiology and Immunology, School of Medicine, University of Missouri, Columbia, MO, USA;
[3]Bond Life Sciences Center, University of Missouri, Columbia, MO, USA;
[4]USDA APHIS Wildlife Services National Wildlife Disease Program, Fort Collins, CO, USA;
[5]National Wildlife Research Center, Wildlife Services, Animal and Plant Health Inspection Service, US Department of Agriculture, Fort Collins, CO, USA;
[6]USDA APHIS Wildlife Services, Fort Collins, CO, USA;
[7]One Health Office, National Center for Emerging and Zoonotic Infectious Diseases, Centers for Disease Control and Prevention, Atlanta, GA, USA;
[8]National Veterinary Services Laboratories, Animal and Plant Health Inspection Service, United States Department of Agriculture, Ames, IA, USA;
[9]National Center for Immunization and Respiratory Diseases, Centers for Disease Control and Prevention, Atlanta, GA, USA;
[10] MU Institute for Data Science and Informatics, University of Missouri, Columbia, Missouri, USA;
[11]Department of Electrical Engineering & Computer Science, College of Engineering, University of Missouri, Columbia, Missouri, USA.

* **Correspondence:** thomas.j.deLiberto@usda.gov; wanx@missouri.edu.

List of Supplementary Figures

Supplementary Figure 1. The geographical locations of the 39 spillover events with transmission of white-tailed deer to white-tailed deer. The identifier for each individual spillover event is presented in Supplementary Data 2, and phylogenetic analyses of these white-tailed deer SARS-CoV-2 with the associated human SARS-CoV-2 sequences in Supplementary Data 11. Source data is provided in the Source Data file.

Supplementary Figure 2. Parameter optimization for determining transmission events associated with white-tailed deer SARS-CoV-2 viruses. a) Distribution of genomic nucleotide sequence identities between each white-tailed deer SARS-CoV-2 and the human potential precursor viruses selected from public database. The number of b) the total spillover events, c) the Human-Deer spillover events, d) the Human-Deer-Deer spillover events, and e) the Human-Deer-Human spillover events were determined by altering the genomic nucleotide sequence identities varying from 99.5% to 99.95% and the posterior probability varying from 0.60 to 0.90. The source data for each subpanel is available in Source Data file.

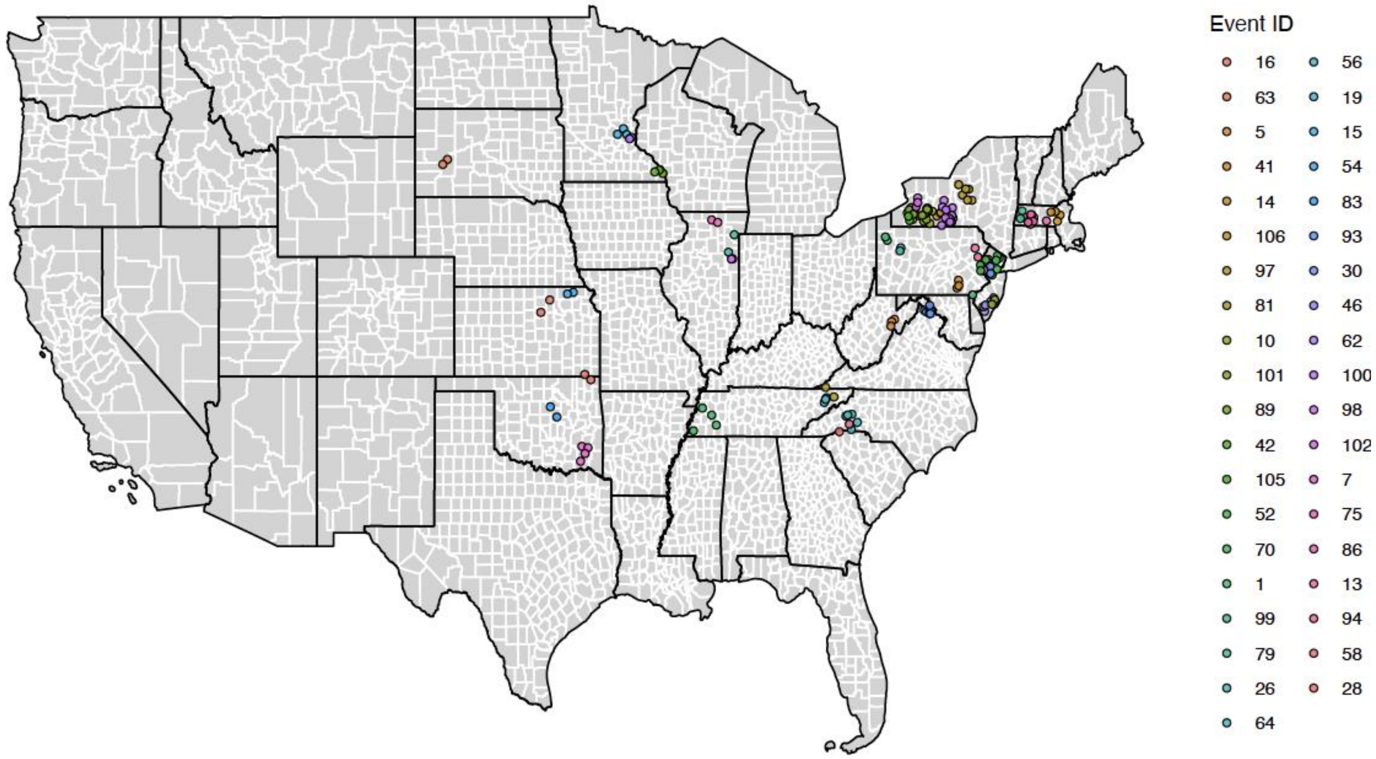

Supplementary Figure 1. The geographical locations of the 39 spillover events with transmission of white-tailed deer to white-tailed deer. The identifier for each individual spillover event is presented in Supplementary Data 2, and phylogenetic analyses of these white-tailed deer SARS-CoV-2 with the associated human SARS-CoV-2 sequences in Supplementary Data 11. Source data is provided in the Source Data file.

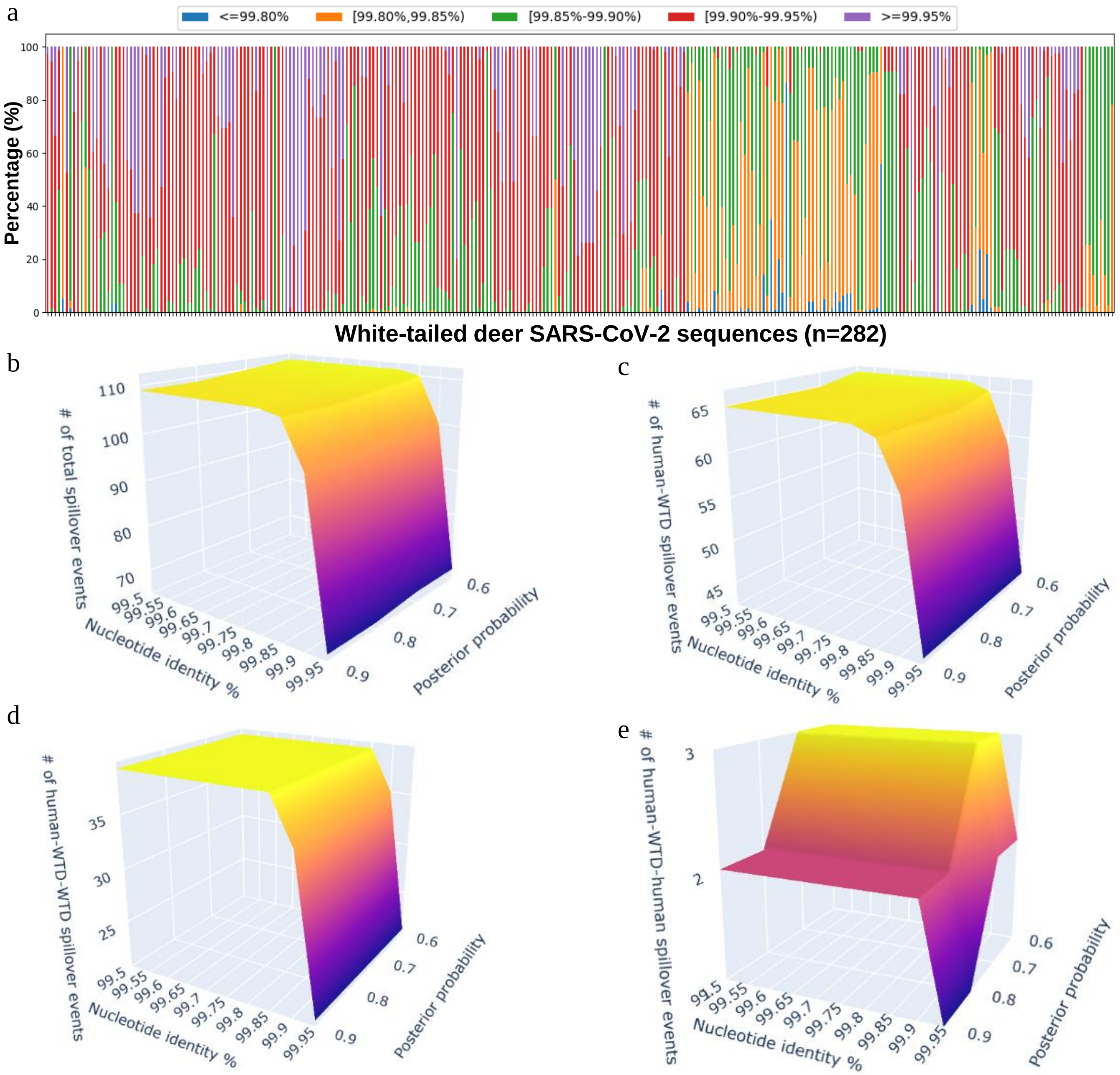

Supplementary Figure 2. Parameter optimization for determining transmission events associated with white-tailed deer SARS-CoV-2 viruses. a) Distribution of genomic nucleotide sequence identities between each white-tailed deer SARS-CoV-2 and the human potential precursor viruses selected from public database. The number of b) the total spillover events, c) the Human-Deer spillover events, d) the Human-Deer-Deer spillover events, and e) the Human-Deer-Human spillover events were determined by altering the genomic nucleotide sequence identities varying from 99.5% to 99.95% and the posterior probability varying from 0.60 to 0.90. The source data for each subpanel is available in Source Data file.
