# Supplementary material for: Transmission of SARS-CoV-2 in free-ranging white-tailed deer in the United States
Source: Nat Commun. 2023 Jul 10;14:4078. doi: 10.1038/s41467-023-39782-x (PMC10333304; doi:10.1038/s41467-023-39782-x)
Supplement: Supplementary file 3 — Description of Additional Supplementary Files [file 41467_2023_39782_MOESM3_ESM.pdf]

Supplementary Data

Transmission of SARS-CoV-2 in free-ranging white-tailed deer in the United States

Authors: Aijing Feng[1,2,3], Sarah Bevins[4], Jeff Chandler[5], Thomas J. DeLiberto[6*], Ria Ghai[7], Kristina Lantz[8], Julianna Lenoch[4], Adam Retchless[9], Susan Shriner[5], Cynthia Y. Tang[1,3,10], Suxiang Sue Tong[9], Mia Torchetti[8], Anna Uehara[9], Xiu-Feng Wan[1,2,3,10,11*]

[1]Center for Influenza and Emerging Infectious Diseases, University of Missouri, Columbia, MO, USA;
[2]Department of Molecular Microbiology and Immunology, School of Medicine, University of Missouri, Columbia, MO, USA;
[3]Bond Life Sciences Center, University of Missouri, Columbia, MO, USA;
[4]USDA APHIS Wildlife Services National Wildlife Disease Program, Fort Collins, CO, USA;
[5]National Wildlife Research Center, Wildlife Services, Animal and Plant Health Inspection Service, US Department of Agriculture, Fort Collins, CO, USA;
[6]USDA APHIS Wildlife Services, Fort Collins, CO, USA;
[7]One Health Office, National Center for Emerging and Zoonotic Infectious Diseases, Centers for Disease Control and Prevention, Atlanta, GA, USA;
[8]National Veterinary Services Laboratories, Animal and Plant Health Inspection Service, United States Department of Agriculture, Ames, IA, USA;
[9]National Center for Immunization and Respiratory Diseases, Centers for Disease Control and Prevention, Atlanta, GA, USA;
[10] MU Institute for Data Science and Informatics, University of Missouri, Columbia, Missouri, USA;
[11]Department of Electrical Engineering & Computer Science, College of Engineering, University of Missouri, Columbia, Missouri, USA.

* **Correspondence:** thomas.j.deLiberto@usda.gov; wanx@missouri.edu.

Supplementary Data 1. Metadata for the white-tailed deer genomic sequences used in this study.

Supplementary Data 2. List of Human-Deer, Human-Deer-Deer, and Human-Deer-Human clusters.

Supplementary Data 3. List of repeated amino acid substitutions identified in the white-tailed deer SARS-CoV-2 sequences. Substitutions that under positive selection are marked with * and those under negative selection with **.

Supplementary Data 4. SARS-CoV-2 positivity in quantitative reverse transcription polymerase chain reaction (qRT-PCR) and surrogate virus neutralization test (sVNT) assays for those white-tailed deer samples from the state of New York.

Supplementary Data 5. List of Bayes factors obtained from phylogeographic analyses for those cross-county transmission events from the state of New York.

Supplementary Data 6. List of human SARS-CoV-2 viruses with North Carolina AY.103 white-tailed deer SARS-CoV-2 specific amino acid substitutions.

Supplementary Data 7. List of human SARS-CoV-2 viruses with Massachusetts white-tailed deer SARS-CoV-2 specific nucleotide polymorphisms.

Supplementary Data 8. List of inter-host single nucleotide variations (iSNVs) from 148 human sequences collected from Missouri. IRMA was used to analyze SNVs and assess intra-host viral genomic diversity. A minority iSNV was identified when the frequency of an allele was at least 5% among the reads.

Supplementary Data 9. List of white-tailed deer adaptive amino acid substitutions that overlap with the epitope reported in human SARS-CoV-2 viruses. The epitopes were obtained from the Immune Epitope Database (https://www.iedb.org).

Supplementary Data 10. Phylogenetic trees for potential precursor human and white-tailed deer SARS-CoV-2 viruses from each 23 states. We constructed a maximum clade credibility tree using white-tailed deer SARS-CoV-

2 sequences from each individual state, along with genetically similar SARS-CoV-2 sequences found in humans. The tree includes posterior probability marks for representative branches.

Supplementary Data 11. Phylogenetic analyses of white-tailed deer SARS-CoV-2 sequences (n = 282) and their potential precursor viruses in humans inferred 109 independent spillovers events of SARS-CoV-2 from humans (directly or indirectly) to white-tailed deer. Three types of spillover events were identified: Human-Deer (green), where each event consists of at least one human precursor sequence and one white-tailed deer SARS-CoV-2 sequence; Human-Deer-Deer (blue), where each event consists of at least one human precursor sequence and at least two white-tailed deer SARS-CoV-2 sequences; Human-Deer-Human (red) where each event consists of at least one human precursor sequence, at least two white-tailed deer sequences, and an additional human SARS-CoV-2 sequence. The spillover event identifiers were shown corresponding in each branch with related color. The nodes in orange were SARS-CoV-2 sequences from white-tailed deer, and those in purple from human. The identifiers for three Human-Deer spillover events with out-of-state human SARS-CoV-2 sequences are shown in black. The estimates of divergence time were obtained by calculating the median node height of the 95% highest posterior density (HPD) interval from a maximum clade credibility tree generated using BEAST. The node bars, depicted in light blue, represent the 95% HPD interval for each node. The timescale of the phylogenetic tree was represented in units of years, and the scale bar indicates the divergence time in years.

Supplementary Data 12. Phylogenetic analyses of the white-tailed deer SARS-CoV-2 sequences (n = 282) and their potential precursor viruses in humans. Because of low coverage at the 5' untranslated region (before position 266) and 3' untranslated region (after position 29,674) of the genome, we excluded these positions from nucleotide and amino acid substitution analyses. In addition, 265 problematic positions summarized at https://github.com/W-L/ProblematicSites_SARS-CoV2 were marked before phylogenetic analyses. The estimates of divergence time were obtained by calculating the median node height of the 95% highest posterior density (HPD) interval from a maximum clade credibility tree generated using BEAST. The node bars, depicted in light blue, represent the 95% HPD interval for each node. The timescale of the phylogenetic tree was represented in units of years, and the scale bar indicates the divergence time in years.
