# Supplementary material for: Transmission of SARS-CoV-2 in free-ranging white-tailed deer in the United States
Source: Nat Commun. 2023 Jul 10;14:4078. doi: 10.1038/s41467-023-39782-x (PMC10333304; doi:10.1038/s41467-023-39782-x)
Supplement: Supplementary file 5 — Supplementary Data 10–12 [file 41467_2023_39782_MOESM5_ESM.zip › Supplementary Data 10-12/Supplementary Data 10.pdf]

Supplementary Data 10. Phylogenetic trees for potential precursor human and white-tailed deer SARS-CoV-2 viruses from each 23 states. We constructed a maximum clade credibility tree using white-tailed deer SARS-CoV-2 sequences from each individual state, along with genetically similar SARS-CoV-2 sequences found in humans. The tree includes posterior probability marks for representative branches.

AR_AY.25	4
AR_AY.54	5
AR_AY.103	6
AR_B.1.617.2	7
DE_B.1.1.7	8
IL_AY.3	9
IL_AY.25.1	10
IL_AY.25	11
IL_AY.26	12
IL_AY.44	13
IL_AY.103	14
IL_AY.118	15
IL_B.1.1.7	16
KS_AY.3	17
KS_AY.39	18
KS_AY.103	19
KY_AY.3	20
KY_AY.103	21
LA_AY.119	22
MA_AY.3	23
MA_AY.4	24
MA_AY.25	25
MA_AY.39.1	26
MA_AY.44	27
MA_AY.103	28
MA_AY.119	29
MA_AY.122	30
MA_B.1.1.7	31
MA_B.1.617.2	32
MD_BA.1.1	33
ME_AY.25	34
ME_AY.103	35
MN_AY.3	36
MN_AY.44	37
MN_AY.100	38
MN_AY.103	39
MN_AY.106	40
MO_AY.3	41
MO_AY.103	42
NC_AY.5.3	43
NC_AY.20	44
NC_AY.25.1	45
NC_AY.39	46

NC_AY.44	47
NC_AY.100	48
NC_AY.103	49
NC_AY.106	50
NC_AY.118	51
NJ_AY.3	52
NJ_AY.4	53
NJ_AY.5	54
NJ_AY.25	55
NJ_AY.39	56
NJ_AY.44	57
NJ_AY.103	58
NJ_AY.111	59
NY_AY.39.1	60
NY_AY.39	61
NY_AY.43	62
NY_AY.98.1	63
NY_AY.103	64
NY_AY.116	65
NY_B.1.1.7	66
NY_B.1.617.2	67
NY_P.1	68
OK_AY.3	69
OK_AY.25	70
OK_AY.118	71
PA_AY.3	72
PA_AY.25.1	73
PA_AY.39	74
PA_AY.44	75
PA_AY.100	76
PA_AY.103	77
PA_AY.107	78
PA_AY.118	79
PA_B.1.1.7	80
PA_B.1	81
PA_P.1	82
SD_AY.100	83
TN_AY.3	84
TN_AY.25	85
TN_AY.39.1	86
TN_AY.39	87
TN_AY.47	88
TN_AY.118	89
WV_AY.103	90
WV_AY.119	91

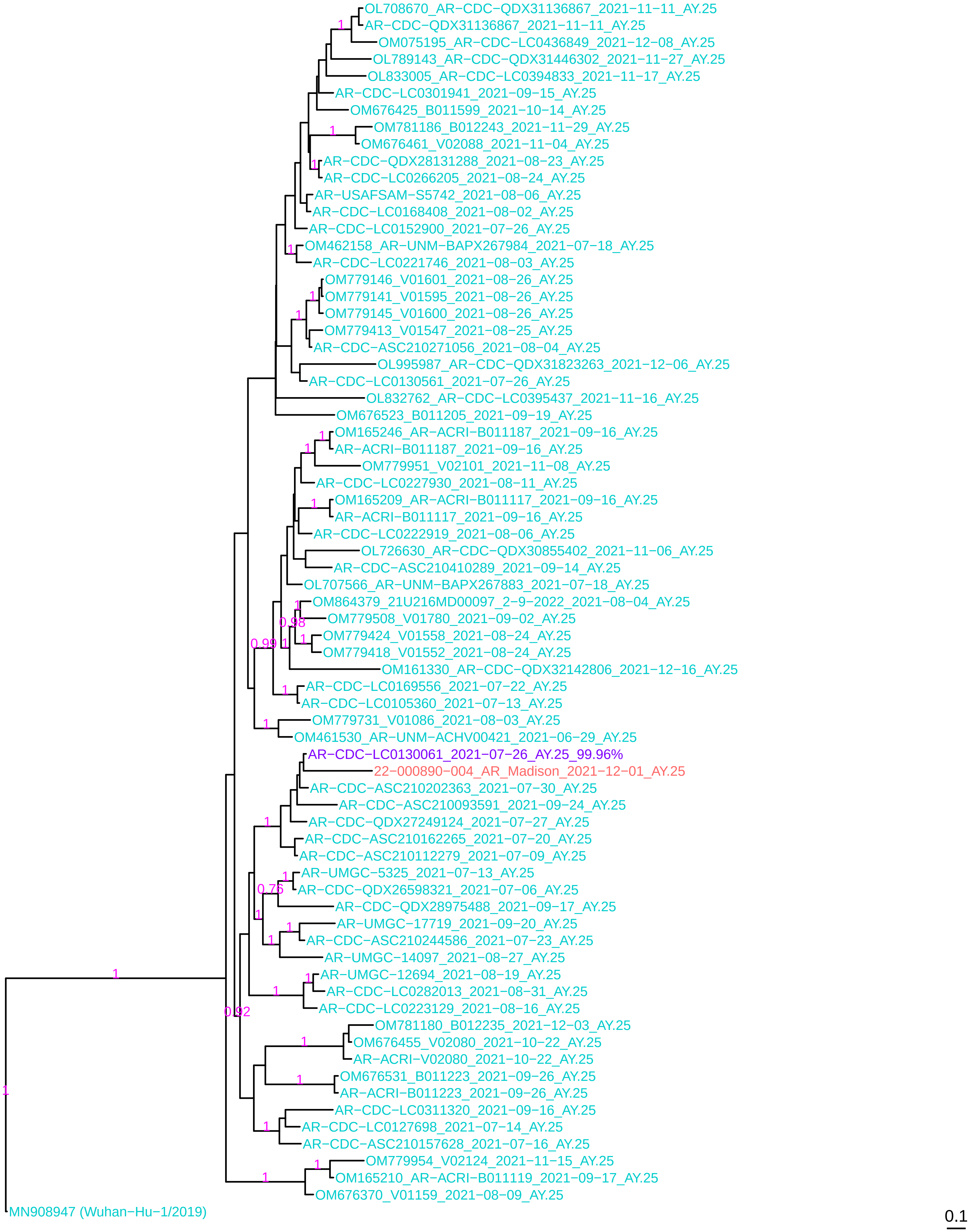

OL708670_AR−CDC−QDX31136867_2021−11−11_AY.25
AR−CDC−QDX31136867_2021−11−11_AY.25
OM075195_AR−CDC−LC0436849_2021−12−08_AY.25
OL789143_AR−CDC−QDX31446302_2021−11−27_AY.25
OL833005_AR−CDC−LC0394833_2021−11−17_AY.25
AR−CDC−LC0301941_2021−09−15_AY.25
OM676425_B011599_2021−10−14_AY.25
OM781186_B012243_2021−11−29_AY.25
OM676461_V02088_2021−11−04_AY.25
AR−CDC−QDX28131288_2021−08−23_AY.25
AR−CDC−LC0266205_2021−08−24_AY.25
AR−USAFSAM−S5742_2021−08−06_AY.25
AR−CDC−LC0168408_2021−08−02_AY.25
AR−CDC−LC0152900_2021−07−26_AY.25
OM462158_AR−UNM−BAPX267984_2021−07−18_AY.25
AR−CDC−LC0221746_2021−08−03_AY.25
OM779146_V01601_2021−08−26_AY.25
OM779141_V01595_2021−08−26_AY.25
OM779145_V01600_2021−08−26_AY.25
OM779413_V01547_2021−08−25_AY.25
AR−CDC−ASC210271056_2021−08−04_AY.25
OL995987_AR−CDC−QDX31823263_2021−12−06_AY.25
AR−CDC−LC0130561_2021−07−26_AY.25
OL832762_AR−CDC−LC0395437_2021−11−16_AY.25
OM676523_B011205_2021−09−19_AY.25
OM165246_AR−ACRI−B011187_2021−09−16_AY.25
AR−ACRI−B011187_2021−09−16_AY.25
OM779951_V02101_2021−11−08_AY.25
AR−CDC−LC0227930_2021−08−11_AY.25
OM165209_AR−ACRI−B011117_2021−09−16_AY.25
AR−ACRI−B011117_2021−09−16_AY.25
AR−CDC−LC0222919_2021−08−06_AY.25
OL726630_AR−CDC−QDX30855402_2021−11−06_AY.25
AR−CDC−ASC210410289_2021−09−14_AY.25
OL707566_AR−UNM−BAPX267883_2021−07−18_AY.25
OM864379_21U216MD00097_2−9−2022_2021−08−04_AY.25
OM779508_V01780_2021−09−02_AY.25
OM779424_V01558_2021−08−24_AY.25
OM779418_V01552_2021−08−24_AY.25
OM161330_AR−CDC−QDX32142806_2021−12−16_AY.25
AR−CDC−LC0169556_2021−07−22_AY.25
AR−CDC−LC0105360_2021−07−13_AY.25
OM779731_V01086_2021−08−03_AY.25
OM461530_AR−UNM−ACHV00421_2021−06−29_AY.25
AR−CDC−LC0130061_2021−07−26_AY.25_99.96%
22−000890−004_AR_Madison_2021−12−01_AY.25
AR−CDC−ASC210202363_2021−07−30_AY.25
AR−CDC−ASC210093591_2021−09−24_AY.25
AR−CDC−QDX27249124_2021−07−27_AY.25
AR−CDC−ASC210162265_2021−07−20_AY.25
AR−CDC−ASC210112279_2021−07−09_AY.25
AR−UMGC−5325_2021−07−13_AY.25
AR−CDC−QDX26598321_2021−07−06_AY.25
AR−CDC−QDX28975488_2021−09−17_AY.25
AR−UMGC−17719_2021−09−20_AY.25
AR−CDC−ASC210244586_2021−07−23_AY.25
AR−UMGC−14097_2021−08−27_AY.25
AR−UMGC−12694_2021−08−19_AY.25
AR−CDC−LC0282013_2021−08−31_AY.25
AR−CDC−LC0223129_2021−08−16_AY.25
OM781180_B012235_2021−12−03_AY.25
OM676455_V02080_2021−10−22_AY.25
AR−ACRI−V02080_2021−10−22_AY.25
OM676531_B011223_2021−09−26_AY.25
AR−ACRI−B011223_2021−09−26_AY.25
AR−CDC−LC0311320_2021−09−16_AY.25
AR−CDC−LC0127698_2021−07−14_AY.25
AR−CDC−ASC210157628_2021−07−16_AY.25
OM779954_V02124_2021−11−15_AY.25
OM165210_AR−ACRI−B011119_2021−09−17_AY.25
OM676370_V01159_2021−08−09_AY.25
MN908947 (Wuhan−Hu−1/2019)
0.1

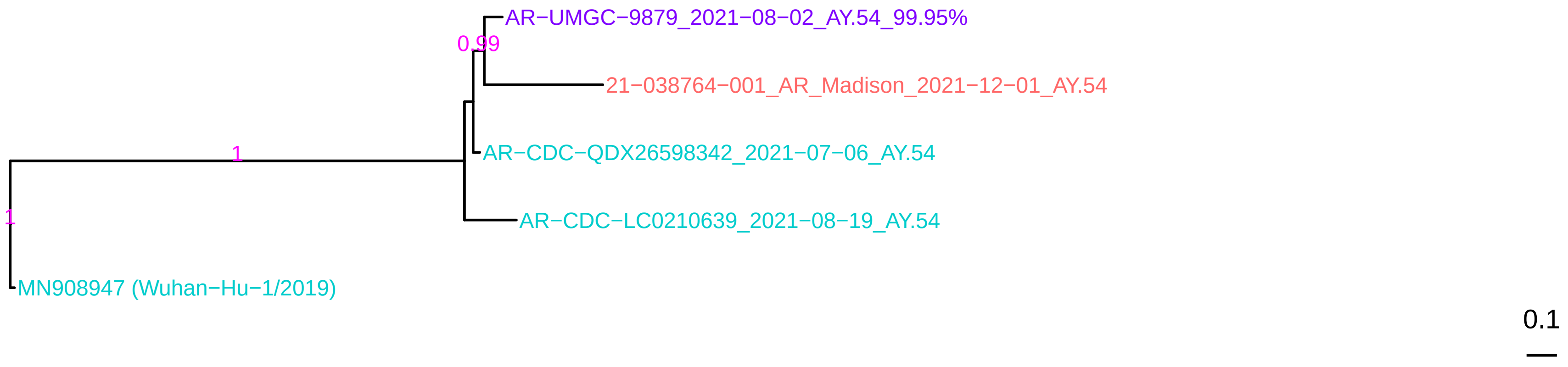

AR−UMGC−9879_2021−08−02_AY.54_99.95%
0.99
21−038764−001_AR_Madison_2021−12−01_AY.54
1
AR−CDC−QDX26598342_2021−07−06_AY.54
AR−CDC−LC0210639_2021−08−19_AY.54
1
MN908947 (Wuhan−Hu−1/2019)
0.1

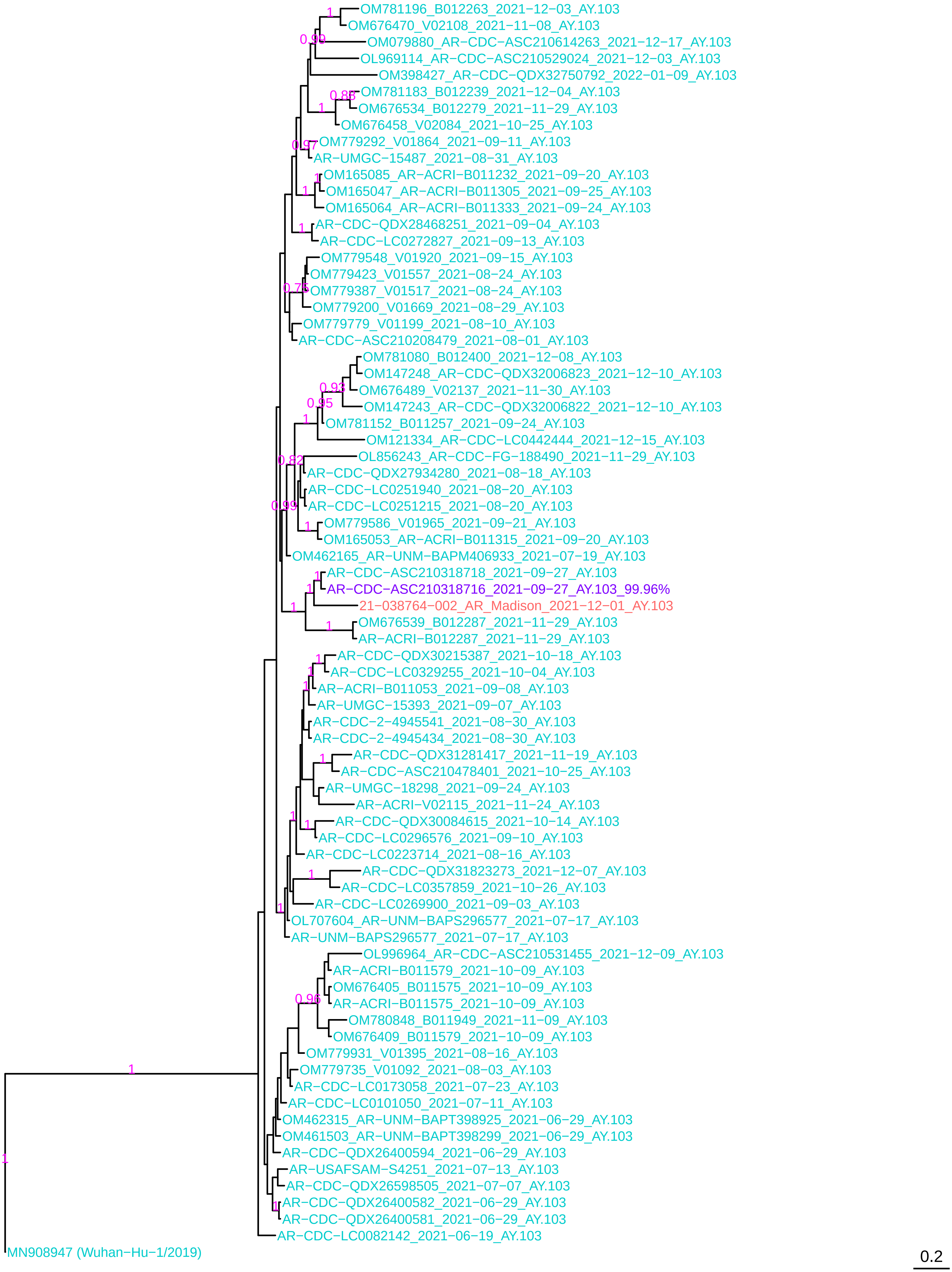

OM781196_B012263_2021−12−03_AY.103
OM676470_V02108_2021−11−08_AY.103
OM079880_AR−CDC−ASC210614263_2021−12−17_AY.103
OL969114_AR−CDC−ASC210529024_2021−12−03_AY.103
OM398427_AR−CDC−QDX32750792_2022−01−09_AY.103
OM781183_B012239_2021−12−04_AY.103
OM676534_B012279_2021−11−29_AY.103
OM676458_V02084_2021−10−25_AY.103
OM779292_V01864_2021−09−11_AY.103
AR−UMGC−15487_2021−08−31_AY.103
OM165085_AR−ACRI−B011232_2021−09−20_AY.103
OM165047_AR−ACRI−B011305_2021−09−25_AY.103
OM165064_AR−ACRI−B011333_2021−09−24_AY.103
AR−CDC−QDX28468251_2021−09−04_AY.103
AR−CDC−LC0272827_2021−09−13_AY.103
OM779548_V01920_2021−09−15_AY.103
OM779423_V01557_2021−08−24_AY.103
OM779387_V01517_2021−08−24_AY.103
OM779200_V01669_2021−08−29_AY.103
OM779779_V01199_2021−08−10_AY.103
AR−CDC−ASC210208479_2021−08−01_AY.103
OM781080_B012400_2021−12−08_AY.103
OM147248_AR−CDC−QDX32006823_2021−12−10_AY.103
OM676489_V02137_2021−11−30_AY.103
OM147243_AR−CDC−QDX32006822_2021−12−10_AY.103
OM781152_B011257_2021−09−24_AY.103
OM121334_AR−CDC−LC0442444_2021−12−15_AY.103
OL856243_AR−CDC−FG−188490_2021−11−29_AY.103
AR−CDC−QDX27934280_2021−08−18_AY.103
AR−CDC−LC0251940_2021−08−20_AY.103
AR−CDC−LC0251215_2021−08−20_AY.103
OM779586_V01965_2021−09−21_AY.103
OM165053_AR−ACRI−B011315_2021−09−20_AY.103
OM462165_AR−UNM−BAPM406933_2021−07−19_AY.103
AR−CDC−ASC210318718_2021−09−27_AY.103
AR−CDC−ASC210318716_2021−09−27_AY.103_99.96%
21−038764−002_AR_Madison_2021−12−01_AY.103
OM676539_B012287_2021−11−29_AY.103
AR−ACRI−B012287_2021−11−29_AY.103
AR−CDC−QDX30215387_2021−10−18_AY.103
AR−CDC−LC0329255_2021−10−04_AY.103
AR−ACRI−B011053_2021−09−08_AY.103
AR−UMGC−15393_2021−09−07_AY.103
AR−CDC−2−4945541_2021−08−30_AY.103
AR−CDC−2−4945434_2021−08−30_AY.103
AR−CDC−QDX31281417_2021−11−19_AY.103
AR−CDC−ASC210478401_2021−10−25_AY.103
AR−UMGC−18298_2021−09−24_AY.103
AR−ACRI−V02115_2021−11−24_AY.103
AR−CDC−QDX30084615_2021−10−14_AY.103
AR−CDC−LC0296576_2021−09−10_AY.103
AR−CDC−LC0223714_2021−08−16_AY.103
AR−CDC−QDX31823273_2021−12−07_AY.103
AR−CDC−LC0357859_2021−10−26_AY.103
AR−CDC−LC0269900_2021−09−03_AY.103
OL707604_AR−UNM−BAPS296577_2021−07−17_AY.103
AR−UNM−BAPS296577_2021−07−17_AY.103
OL996964_AR−CDC−ASC210531455_2021−12−09_AY.103
AR−ACRI−B011579_2021−10−09_AY.103
OM676405_B011575_2021−10−09_AY.103
AR−ACRI−B011575_2021−10−09_AY.103
OM780848_B011949_2021−11−09_AY.103
OM676409_B011579_2021−10−09_AY.103
OM779931_V01395_2021−08−16_AY.103
OM779735_V01092_2021−08−03_AY.103
AR−CDC−LC0173058_2021−07−23_AY.103
AR−CDC−LC0101050_2021−07−11_AY.103
OM462315_AR−UNM−BAPT398925_2021−06−29_AY.103
OM461503_AR−UNM−BAPT398299_2021−06−29_AY.103
AR−CDC−QDX26400594_2021−06−29_AY.103
AR−USAFSAM−S4251_2021−07−13_AY.103
AR−CDC−QDX26598505_2021−07−07_AY.103
AR−CDC−QDX26400582_2021−06−29_AY.103
AR−CDC−QDX26400581_2021−06−29_AY.103
AR−CDC−LC0082142_2021−06−19_AY.103
MN908947 (Wuhan−Hu−1/2019)
0.2

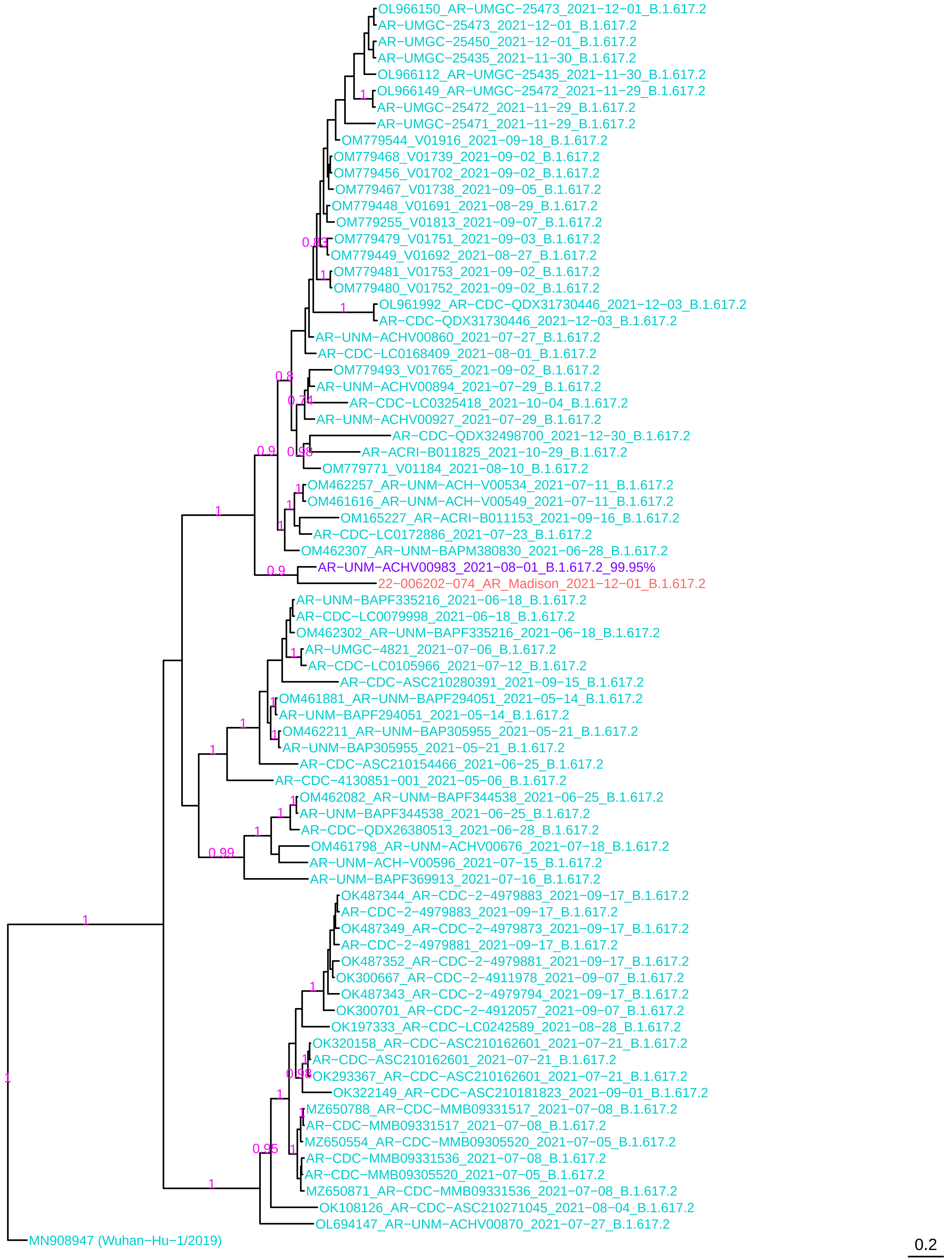

OL966150_AR−UMGC−25473_2021−12−01_B.1.617.2
AR−UMGC−25473_2021−12−01_B.1.617.2
AR−UMGC−25450_2021−12−01_B.1.617.2
AR−UMGC−25435_2021−11−30_B.1.617.2
OL966112_AR−UMGC−25435_2021−11−30_B.1.617.2
OL966149_AR−UMGC−25472_2021−11−29_B.1.617.2
AR−UMGC−25472_2021−11−29_B.1.617.2
AR−UMGC−25471_2021−11−29_B.1.617.2
OM779544_V01916_2021−09−18_B.1.617.2
OM779468_V01739_2021−09−02_B.1.617.2
OM779456_V01702_2021−09−02_B.1.617.2
OM779467_V01738_2021−09−05_B.1.617.2
OM779448_V01691_2021−08−29_B.1.617.2
OM779255_V01813_2021−09−07_B.1.617.2
OM779479_V01751_2021−09−03_B.1.617.2
OM779449_V01692_2021−08−27_B.1.617.2
OM779481_V01753_2021−09−02_B.1.617.2
OM779480_V01752_2021−09−02_B.1.617.2
OL961992_AR−CDC−QDX31730446_2021−12−03_B.1.617.2
AR−CDC−QDX31730446_2021−12−03_B.1.617.2
AR−UNM−ACHV00860_2021−07−27_B.1.617.2
AR−CDC−LC0168409_2021−08−01_B.1.617.2
OM779493_V01765_2021−09−02_B.1.617.2
AR−UNM−ACHV00894_2021−07−29_B.1.617.2
AR−CDC−LC0325418_2021−10−04_B.1.617.2
AR−UNM−ACHV00927_2021−07−29_B.1.617.2
AR−CDC−QDX32498700_2021−12−30_B.1.617.2
AR−ACRI−B011825_2021−10−29_B.1.617.2
OM779771_V01184_2021−08−10_B.1.617.2
OM462257_AR−UNM−ACH−V00534_2021−07−11_B.1.617.2
OM461616_AR−UNM−ACH−V00549_2021−07−11_B.1.617.2
OM165227_AR−ACRI−B011153_2021−09−16_B.1.617.2
AR−CDC−LC0172886_2021−07−23_B.1.617.2
OM462307_AR−UNM−BAPM380830_2021−06−28_B.1.617.2
AR−UNM−ACHV00983_2021−08−01_B.1.617.2_99.95%
22−006202−074_AR_Madison_2021−12−01_B.1.617.2
AR−UNM−BAPF335216_2021−06−18_B.1.617.2
AR−CDC−LC0079998_2021−06−18_B.1.617.2
OM462302_AR−UNM−BAPF335216_2021−06−18_B.1.617.2
AR−UMGC−4821_2021−07−06_B.1.617.2
AR−CDC−LC0105966_2021−07−12_B.1.617.2
AR−CDC−ASC210280391_2021−09−15_B.1.617.2
OM461881_AR−UNM−BAPF294051_2021−05−14_B.1.617.2
AR−UNM−BAPF294051_2021−05−14_B.1.617.2
OM462211_AR−UNM−BAP305955_2021−05−21_B.1.617.2
AR−UNM−BAP305955_2021−05−21_B.1.617.2
AR−CDC−ASC210154466_2021−06−25_B.1.617.2
AR−CDC−4130851−001_2021−05−06_B.1.617.2
OM462082_AR−UNM−BAPF344538_2021−06−25_B.1.617.2
AR−UNM−BAPF344538_2021−06−25_B.1.617.2
AR−CDC−QDX26380513_2021−06−28_B.1.617.2
OM461798_AR−UNM−ACHV00676_2021−07−18_B.1.617.2
AR−UNM−ACH−V00596_2021−07−15_B.1.617.2
AR−UNM−BAPF369913_2021−07−16_B.1.617.2
OK487344_AR−CDC−2−4979883_2021−09−17_B.1.617.2
AR−CDC−2−4979883_2021−09−17_B.1.617.2
OK487349_AR−CDC−2−4979873_2021−09−17_B.1.617.2
AR−CDC−2−4979881_2021−09−17_B.1.617.2
OK487352_AR−CDC−2−4979881_2021−09−17_B.1.617.2
OK300667_AR−CDC−2−4911978_2021−09−07_B.1.617.2
OK487343_AR−CDC−2−4979794_2021−09−17_B.1.617.2
OK300701_AR−CDC−2−4912057_2021−09−07_B.1.617.2
OK197333_AR−CDC−LC0242589_2021−08−28_B.1.617.2
OK320158_AR−CDC−ASC210162601_2021−07−21_B.1.617.2
AR−CDC−ASC210162601_2021−07−21_B.1.617.2
OK293367_AR−CDC−ASC210162601_2021−07−21_B.1.617.2
OK322149_AR−CDC−ASC210181823_2021−09−01_B.1.617.2
MZ650788_AR−CDC−MMB09331517_2021−07−08_B.1.617.2
AR−CDC−MMB09331517_2021−07−08_B.1.617.2
MZ650554_AR−CDC−MMB09305520_2021−07−05_B.1.617.2
AR−CDC−MMB09331536_2021−07−08_B.1.617.2
AR−CDC−MMB09305520_2021−07−05_B.1.617.2
MZ650871_AR−CDC−MMB09331536_2021−07−08_B.1.617.2
OK108126_AR−CDC−ASC210271045_2021−08−04_B.1.617.2
OL694147_AR−UNM−ACHV00870_2021−07−27_B.1.617.2
MN908947 (Wuhan−Hu−1/2019)
0.2

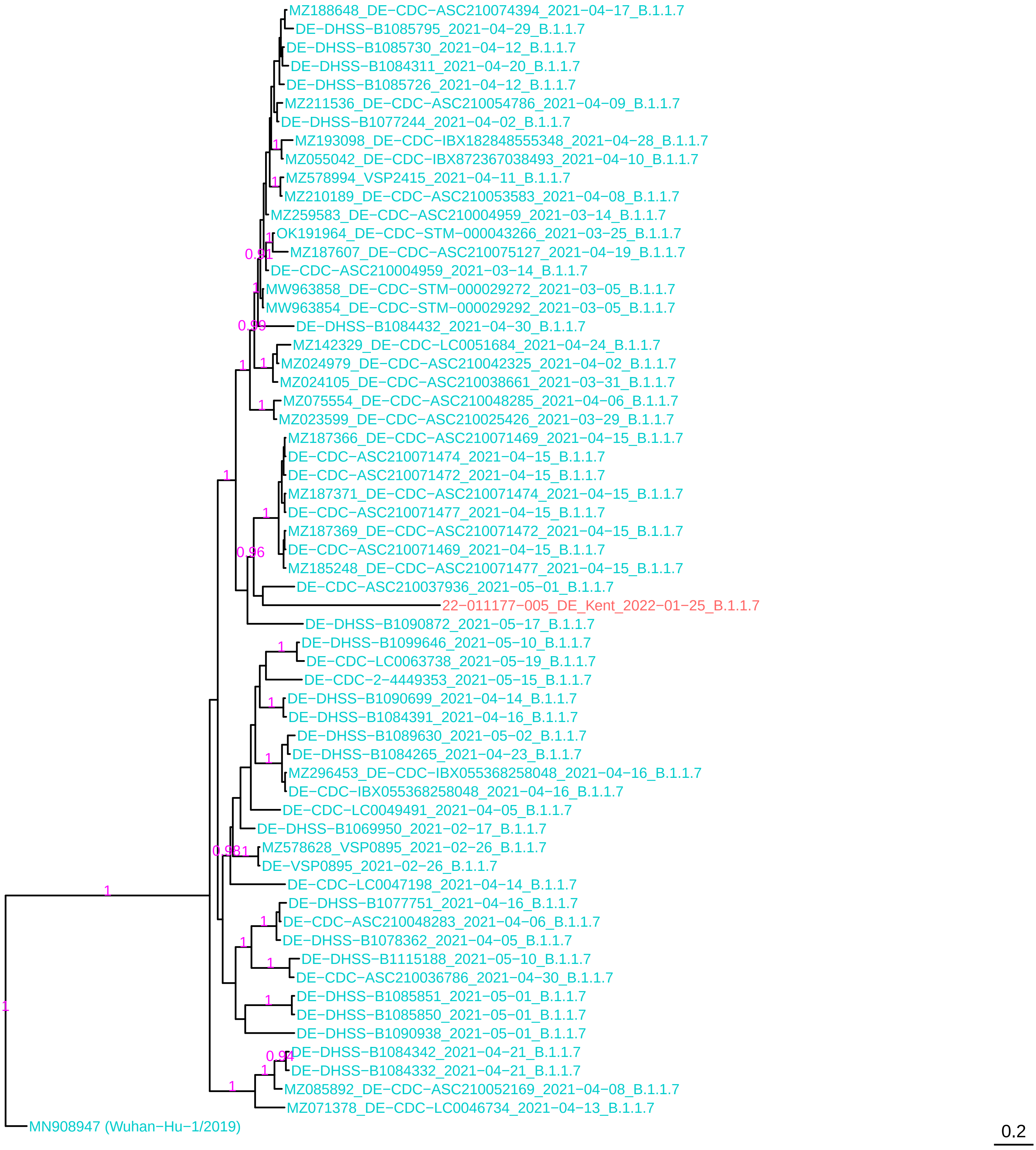
MZ188648_DE−CDC−ASC210074394_2021−04−17_B.1.1.7
DE−DHSS−B1085795_2021−04−29_B.1.1.7
DE−DHSS−B1085730_2021−04−12_B.1.1.7
DE−DHSS−B1084311_2021−04−20_B.1.1.7
DE−DHSS−B1085726_2021−04−12_B.1.1.7
MZ211536_DE−CDC−ASC210054786_2021−04−09_B.1.1.7
DE−DHSS−B1077244_2021−04−02_B.1.1.7
1
MZ193098_DE−CDC−IBX182848555348_2021−04−28_B.1.1.7
MZ055042_DE−CDC−IBX872367038493_2021−04−10_B.1.1.7
1
MZ578994_VSP2415_2021−04−11_B.1.1.7
MZ210189_DE−CDC−ASC210053583_2021−04−08_B.1.1.7
MZ259583_DE−CDC−ASC210004959_2021−03−14_B.1.1.7
1
OK191964_DE−CDC−STM−000043266_2021−03−25_B.1.1.7
0.91
MZ187607_DE−CDC−ASC210075127_2021−04−19_B.1.1.7
DE−CDC−ASC210004959_2021−03−14_B.1.1.7
1
MW963858_DE−CDC−STM−000029272_2021−03−05_B.1.1.7
MW963854_DE−CDC−STM−000029292_2021−03−05_B.1.1.7
0.99
DE−DHSS−B1084432_2021−04−30_B.1.1.7
MZ142329_DE−CDC−LC0051684_2021−04−24_B.1.1.7
1
1
MZ024979_DE−CDC−ASC210042325_2021−04−02_B.1.1.7
MZ024105_DE−CDC−ASC210038661_2021−03−31_B.1.1.7
1
MZ075554_DE−CDC−ASC210048285_2021−04−06_B.1.1.7
MZ023599_DE−CDC−ASC210025426_2021−03−29_B.1.1.7
MZ187366_DE−CDC−ASC210071469_2021−04−15_B.1.1.7
DE−CDC−ASC210071474_2021−04−15_B.1.1.7
1
DE−CDC−ASC210071472_2021−04−15_B.1.1.7
MZ187371_DE−CDC−ASC210071474_2021−04−15_B.1.1.7
1
DE−CDC−ASC210071477_2021−04−15_B.1.1.7
MZ187369_DE−CDC−ASC210071472_2021−04−15_B.1.1.7
0.96
DE−CDC−ASC210071469_2021−04−15_B.1.1.7
MZ185248_DE−CDC−ASC210071477_2021−04−15_B.1.1.7
DE−CDC−ASC210037936_2021−05−01_B.1.1.7
22−011177−005_DE_Kent_2022−01−25_B.1.1.7
DE−DHSS−B1090872_2021−05−17_B.1.1.7
1
DE−DHSS−B1099646_2021−05−10_B.1.1.7
DE−CDC−LC0063738_2021−05−19_B.1.1.7
DE−CDC−2−4449353_2021−05−15_B.1.1.7
1
DE−DHSS−B1090699_2021−04−14_B.1.1.7
DE−DHSS−B1084391_2021−04−16_B.1.1.7
DE−DHSS−B1089630_2021−05−02_B.1.1.7
1
DE−DHSS−B1084265_2021−04−23_B.1.1.7
MZ296453_DE−CDC−IBX055368258048_2021−04−16_B.1.1.7
DE−CDC−IBX055368258048_2021−04−16_B.1.1.7
DE−CDC−LC0049491_2021−04−05_B.1.1.7
DE−DHSS−B1069950_2021−02−17_B.1.1.7
0.98
1
MZ578628_VSP0895_2021−02−26_B.1.1.7
DE−VSP0895_2021−02−26_B.1.1.7
DE−CDC−LC0047198_2021−04−14_B.1.1.7
1
DE−DHSS−B1077751_2021−04−16_B.1.1.7
1
DE−CDC−ASC210048283_2021−04−06_B.1.1.7
DE−DHSS−B1078362_2021−04−05_B.1.1.7
1
DE−DHSS−B1115188_2021−05−10_B.1.1.7
DE−CDC−ASC210036786_2021−04−30_B.1.1.7
1
DE−DHSS−B1085851_2021−05−01_B.1.1.7
DE−DHSS−B1085850_2021−05−01_B.1.1.7
1
DE−DHSS−B1090938_2021−05−01_B.1.1.7
0.94
DE−DHSS−B1084342_2021−04−21_B.1.1.7
1
DE−DHSS−B1084332_2021−04−21_B.1.1.7
1
MZ085892_DE−CDC−ASC210052169_2021−04−08_B.1.1.7
MZ071378_DE−CDC−LC0046734_2021−04−13_B.1.1.7
1
MN908947 (Wuhan−Hu−1/2019)
0.2

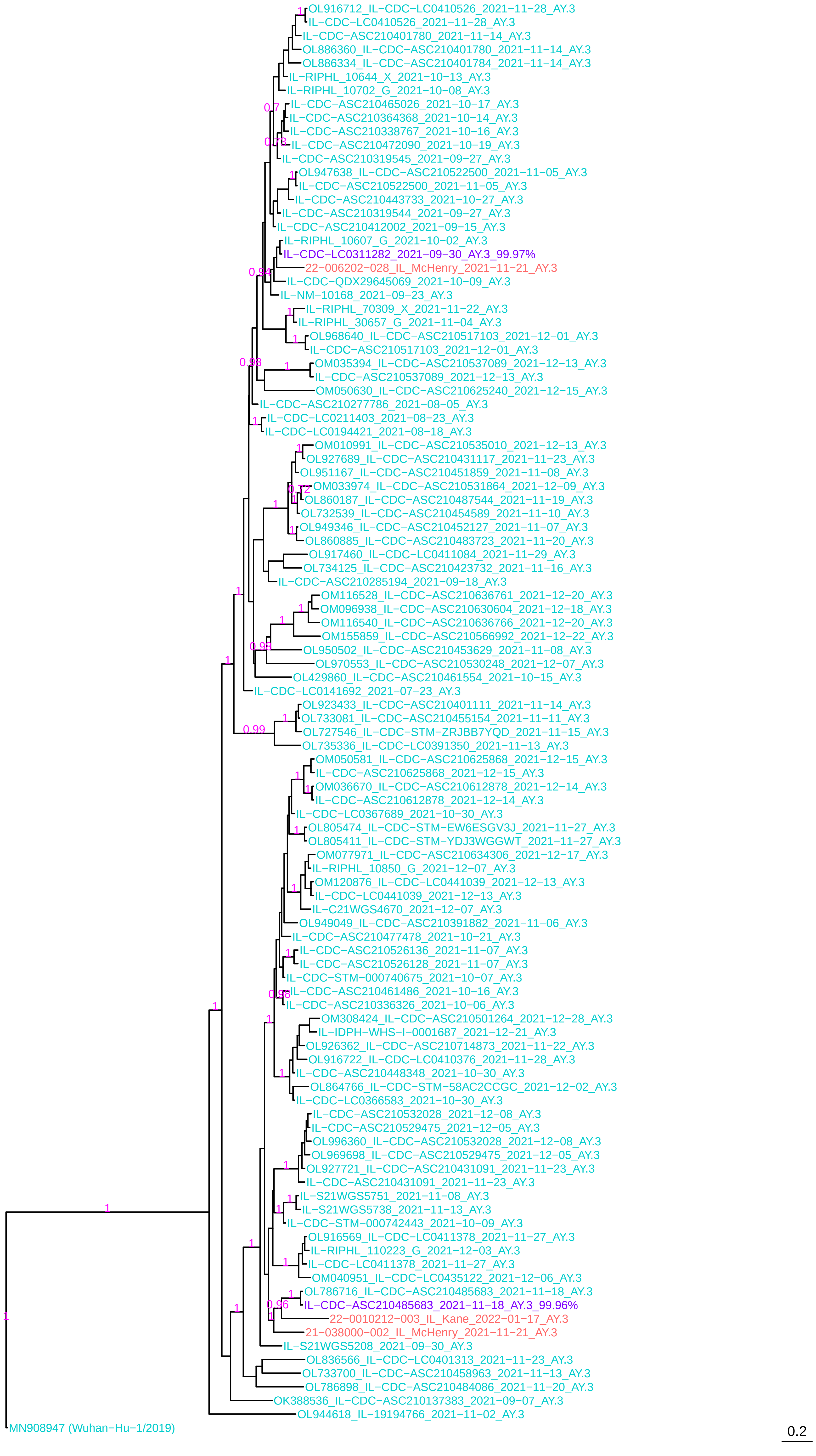
OL916712_IL-CDC-LC0410526_2021-11-28_AY.3
IL-CDC-LC0410526_2021-11-28_AY.3
IL-CDC-ASC210401780_2021-11-14_AY.3
OL886360_IL-CDC-ASC210401780_2021-11-14_AY.3
OL886334_IL-CDC-ASC210401784_2021-11-14_AY.3
IL-RIPHL_10644_X_2021-10-13_AY.3
IL-RIPHL_10702_G_2021-10-08_AY.3
IL-CDC-ASC210465026_2021-10-17_AY.3
IL-CDC-ASC210364368_2021-10-14_AY.3
IL-CDC-ASC210338767_2021-10-16_AY.3
IL-CDC-ASC210472090_2021-10-19_AY.3
IL-CDC-ASC210319545_2021-09-27_AY.3
OL947638_IL-CDC-ASC210522500_2021-11-05_AY.3
IL-CDC-ASC210522500_2021-11-05_AY.3
IL-CDC-ASC210443733_2021-10-27_AY.3
IL-CDC-ASC210319544_2021-09-27_AY.3
IL-CDC-ASC210412002_2021-09-15_AY.3
IL-RIPHL_10607_G_2021-10-02_AY.3
IL-CDC-LC0311282_2021-09-30_AY.3_99.97%
22-006202-028_IL_McHenry_2021-11-21_AY.3
IL-CDC-QDX29645069_2021-10-09_AY.3
IL-NM-10168_2021-09-23_AY.3
IL-RIPHL_70309_X_2021-11-22_AY.3
IL-RIPHL_30657_G_2021-11-04_AY.3
OL968640_IL-CDC-ASC210517103_2021-12-01_AY.3
IL-CDC-ASC210517103_2021-12-01_AY.3
OM035394_IL-CDC-ASC210537089_2021-12-13_AY.3
IL-CDC-ASC210537089_2021-12-13_AY.3
OM050630_IL-CDC-ASC210625240_2021-12-15_AY.3
IL-CDC-ASC210277786_2021-08-05_AY.3
IL-CDC-LC0211403_2021-08-23_AY.3
IL-CDC-LC0194421_2021-08-18_AY.3
OM010991_IL-CDC-ASC210535010_2021-12-13_AY.3
OL927689_IL-CDC-ASC210431117_2021-11-23_AY.3
OL951167_IL-CDC-ASC210451859_2021-11-08_AY.3
OM033974_IL-CDC-ASC210531864_2021-12-09_AY.3
OL860187_IL-CDC-ASC210487544_2021-11-19_AY.3
OL732539_IL-CDC-ASC210454589_2021-11-10_AY.3
OL949346_IL-CDC-ASC210452127_2021-11-07_AY.3
OL860885_IL-CDC-ASC210483723_2021-11-20_AY.3
OL917460_IL-CDC-LC0411084_2021-11-29_AY.3
OL734125_IL-CDC-ASC210423732_2021-11-16_AY.3
IL-CDC-ASC210285194_2021-09-18_AY.3
OM116528_IL-CDC-ASC210636761_2021-12-20_AY.3
OM096938_IL-CDC-ASC210630604_2021-12-18_AY.3
OM116540_IL-CDC-ASC210636766_2021-12-20_AY.3
OM155859_IL-CDC-ASC210566992_2021-12-22_AY.3
OL950502_IL-CDC-ASC210453629_2021-11-08_AY.3
OL970553_IL-CDC-ASC210530248_2021-12-07_AY.3
OL429860_IL-CDC-ASC210461554_2021-10-15_AY.3
IL-CDC-LC0141692_2021-07-23_AY.3
OL923433_IL-CDC-ASC210401111_2021-11-14_AY.3
OL733081_IL-CDC-ASC210455154_2021-11-11_AY.3
OL727546_IL-CDC-STM-ZRJBB7YQD_2021-11-15_AY.3
OL735336_IL-CDC-LC0391350_2021-11-13_AY.3
OM050581_IL-CDC-ASC210625868_2021-12-15_AY.3
IL-CDC-ASC210625868_2021-12-15_AY.3
OM036670_IL-CDC-ASC210612878_2021-12-14_AY.3
IL-CDC-ASC210612878_2021-12-14_AY.3
IL-CDC-LC0367689_2021-10-30_AY.3
OL805474_IL-CDC-STM-EW6ESGV3J_2021-11-27_AY.3
OL805411_IL-CDC-STM-YDJ3WGGWT_2021-11-27_AY.3
OM077971_IL-CDC-ASC210634306_2021-12-17_AY.3
IL-RIPHL_10850_G_2021-12-07_AY.3
OM120876_IL-CDC-LC0441039_2021-12-13_AY.3
IL-CDC-LC0441039_2021-12-13_AY.3
IL-C21WGS4670_2021-12-07_AY.3
OL949049_IL-CDC-ASC210391882_2021-11-06_AY.3
IL-CDC-ASC210477478_2021-10-21_AY.3
IL-CDC-ASC210526136_2021-11-07_AY.3
IL-CDC-ASC210526128_2021-11-07_AY.3
IL-CDC-STM-000740675_2021-10-07_AY.3
IL-CDC-ASC210461486_2021-10-16_AY.3
IL-CDC-ASC210336326_2021-10-06_AY.3
OM308424_IL-CDC-ASC210501264_2021-12-28_AY.3
IL-IDPH-WHS-I-0001687_2021-12-21_AY.3
OL926362_IL-CDC-ASC210714873_2021-11-22_AY.3
OL916722_IL-CDC-LC0410376_2021-11-28_AY.3
IL-CDC-ASC210448348_2021-10-30_AY.3
OL864766_IL-CDC-STM-58AC2CCGC_2021-12-02_AY.3
IL-CDC-LC0366583_2021-10-30_AY.3
IL-CDC-ASC210532028_2021-12-08_AY.3
IL-CDC-ASC210529475_2021-12-05_AY.3
OL996360_IL-CDC-ASC210532028_2021-12-08_AY.3
OL969698_IL-CDC-ASC210529475_2021-12-05_AY.3
OL927721_IL-CDC-ASC210431091_2021-11-23_AY.3
IL-CDC-ASC210431091_2021-11-23_AY.3
IL-S21WGS5751_2021-11-08_AY.3
IL-S21WGS5738_2021-11-13_AY.3
IL-CDC-STM-000742443_2021-10-09_AY.3
OL916569_IL-CDC-LC0411378_2021-11-27_AY.3
IL-RIPHL_110223_G_2021-12-03_AY.3
IL-CDC-LC0411378_2021-11-27_AY.3
OM040951_IL-CDC-LC0435122_2021-12-06_AY.3
OL786716_IL-CDC-ASC210485683_2021-11-18_AY.3
IL-CDC-ASC210485683_2021-11-18_AY.3_99.96%
22-0010212-003_IL_Kane_2022-01-17_AY.3
21-038000-002_IL_McHenry_2021-11-21_AY.3
IL-S21WGS5208_2021-09-30_AY.3
OL836566_IL-CDC-LC0401313_2021-11-23_AY.3
OL733700_IL-CDC-ASC210458963_2021-11-13_AY.3
OL786898_IL-CDC-ASC210484086_2021-11-20_AY.3
OK388536_IL-CDC-ASC210137383_2021-09-07_AY.3
OL944618_IL-19194766_2021-11-02_AY.3
MN908947 (Wuhan-Hu-1/2019)
0.2

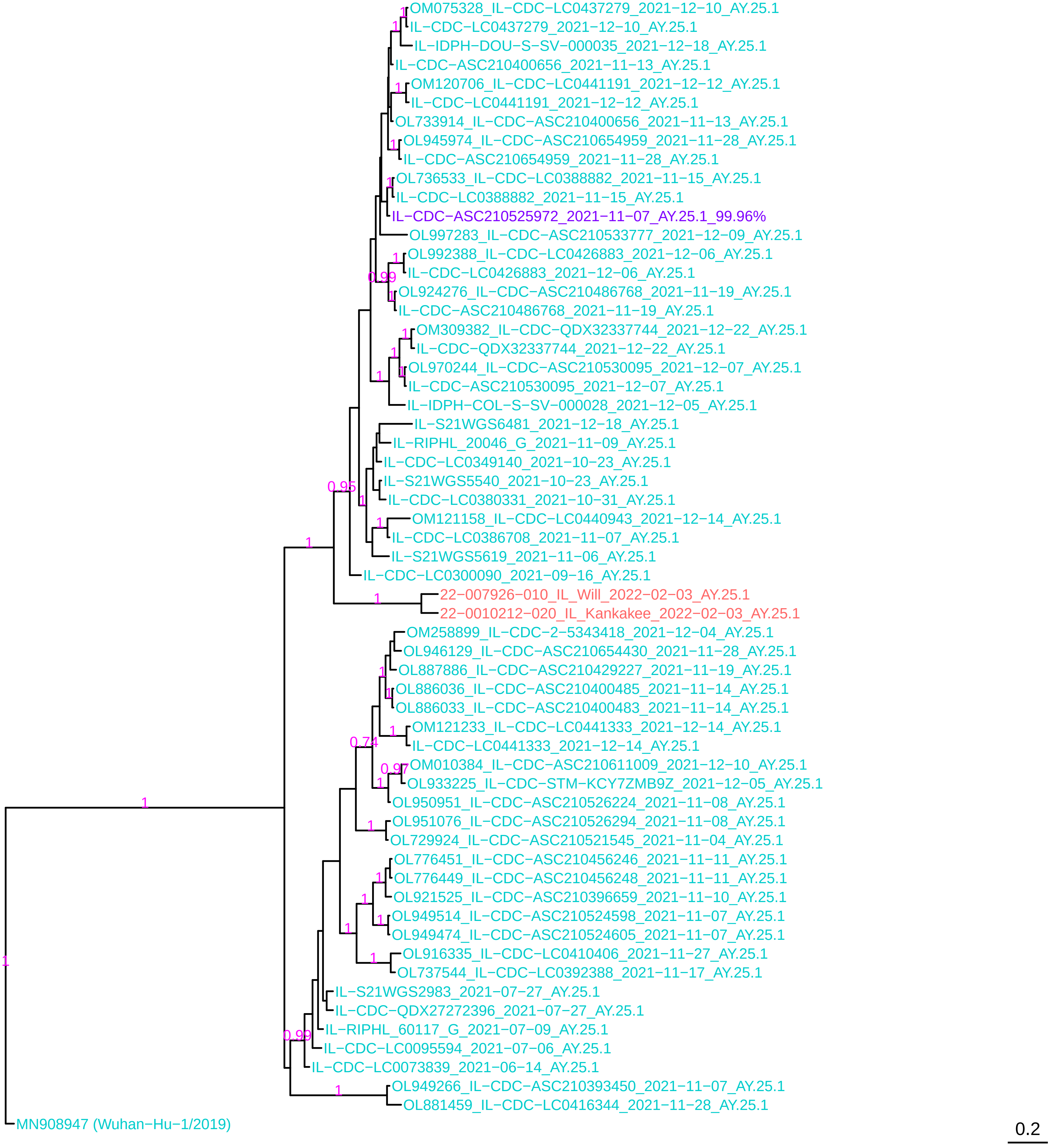

OM075328_IL−CDC−LC0437279_2021−12−10_AY.25.1
IL−CDC−LC0437279_2021−12−10_AY.25.1
IL−IDPH−DOU−S−SV−000035_2021−12−18_AY.25.1
IL−CDC−ASC210400656_2021−11−13_AY.25.1
OM120706_IL−CDC−LC0441191_2021−12−12_AY.25.1
IL−CDC−LC0441191_2021−12−12_AY.25.1
OL733914_IL−CDC−ASC210400656_2021−11−13_AY.25.1
OL945974_IL−CDC−ASC210654959_2021−11−28_AY.25.1
IL−CDC−ASC210654959_2021−11−28_AY.25.1
OL736533_IL−CDC−LC0388882_2021−11−15_AY.25.1
IL−CDC−LC0388882_2021−11−15_AY.25.1
IL−CDC−ASC210525972_2021−11−07_AY.25.1_99.96%
OL997283_IL−CDC−ASC210533777_2021−12−09_AY.25.1
OL992388_IL−CDC−LC0426883_2021−12−06_AY.25.1
IL−CDC−LC0426883_2021−12−06_AY.25.1
OL924276_IL−CDC−ASC210486768_2021−11−19_AY.25.1
IL−CDC−ASC210486768_2021−11−19_AY.25.1
OM309382_IL−CDC−QDX32337744_2021−12−22_AY.25.1
IL−CDC−QDX32337744_2021−12−22_AY.25.1
OL970244_IL−CDC−ASC210530095_2021−12−07_AY.25.1
IL−CDC−ASC210530095_2021−12−07_AY.25.1
IL−IDPH−COL−S−SV−000028_2021−12−05_AY.25.1
IL−S21WGS6481_2021−12−18_AY.25.1
IL−RIPHL_20046_G_2021−11−09_AY.25.1
IL−CDC−LC0349140_2021−10−23_AY.25.1
IL−S21WGS5540_2021−10−23_AY.25.1
IL−CDC−LC0380331_2021−10−31_AY.25.1
OM121158_IL−CDC−LC0440943_2021−12−14_AY.25.1
IL−CDC−LC0386708_2021−11−07_AY.25.1
IL−S21WGS5619_2021−11−06_AY.25.1
IL−CDC−LC0300090_2021−09−16_AY.25.1
22−007926−010_IL_Will_2022−02−03_AY.25.1
22−0010212−020_IL_Kankakee_2022−02−03_AY.25.1
OM258899_IL−CDC−2−5343418_2021−12−04_AY.25.1
OL946129_IL−CDC−ASC210654430_2021−11−28_AY.25.1
OL887886_IL−CDC−ASC210429227_2021−11−19_AY.25.1
OL886036_IL−CDC−ASC210400485_2021−11−14_AY.25.1
OL886033_IL−CDC−ASC210400483_2021−11−14_AY.25.1
OM121233_IL−CDC−LC0441333_2021−12−14_AY.25.1
IL−CDC−LC0441333_2021−12−14_AY.25.1
OM010384_IL−CDC−ASC210611009_2021−12−10_AY.25.1
OL933225_IL−CDC−STM−KCY7ZMB9Z_2021−12−05_AY.25.1
OL950951_IL−CDC−ASC210526224_2021−11−08_AY.25.1
OL951076_IL−CDC−ASC210526294_2021−11−08_AY.25.1
OL729924_IL−CDC−ASC210521545_2021−11−04_AY.25.1
OL776451_IL−CDC−ASC210456246_2021−11−11_AY.25.1
OL776449_IL−CDC−ASC210456248_2021−11−11_AY.25.1
OL921525_IL−CDC−ASC210396659_2021−11−10_AY.25.1
OL949514_IL−CDC−ASC210524598_2021−11−07_AY.25.1
OL949474_IL−CDC−ASC210524605_2021−11−07_AY.25.1
OL916335_IL−CDC−LC0410406_2021−11−27_AY.25.1
OL737544_IL−CDC−LC0392388_2021−11−17_AY.25.1
IL−S21WGS2983_2021−07−27_AY.25.1
IL−CDC−QDX27272396_2021−07−27_AY.25.1
IL−RIPHL_60117_G_2021−07−09_AY.25.1
IL−CDC−LC0095594_2021−07−06_AY.25.1
IL−CDC−LC0073839_2021−06−14_AY.25.1
OL949266_IL−CDC−ASC210393450_2021−11−07_AY.25.1
OL881459_IL−CDC−LC0416344_2021−11−28_AY.25.1
MN908947 (Wuhan−Hu−1/2019)
0.99
0.95
0.74
0.97
0.2

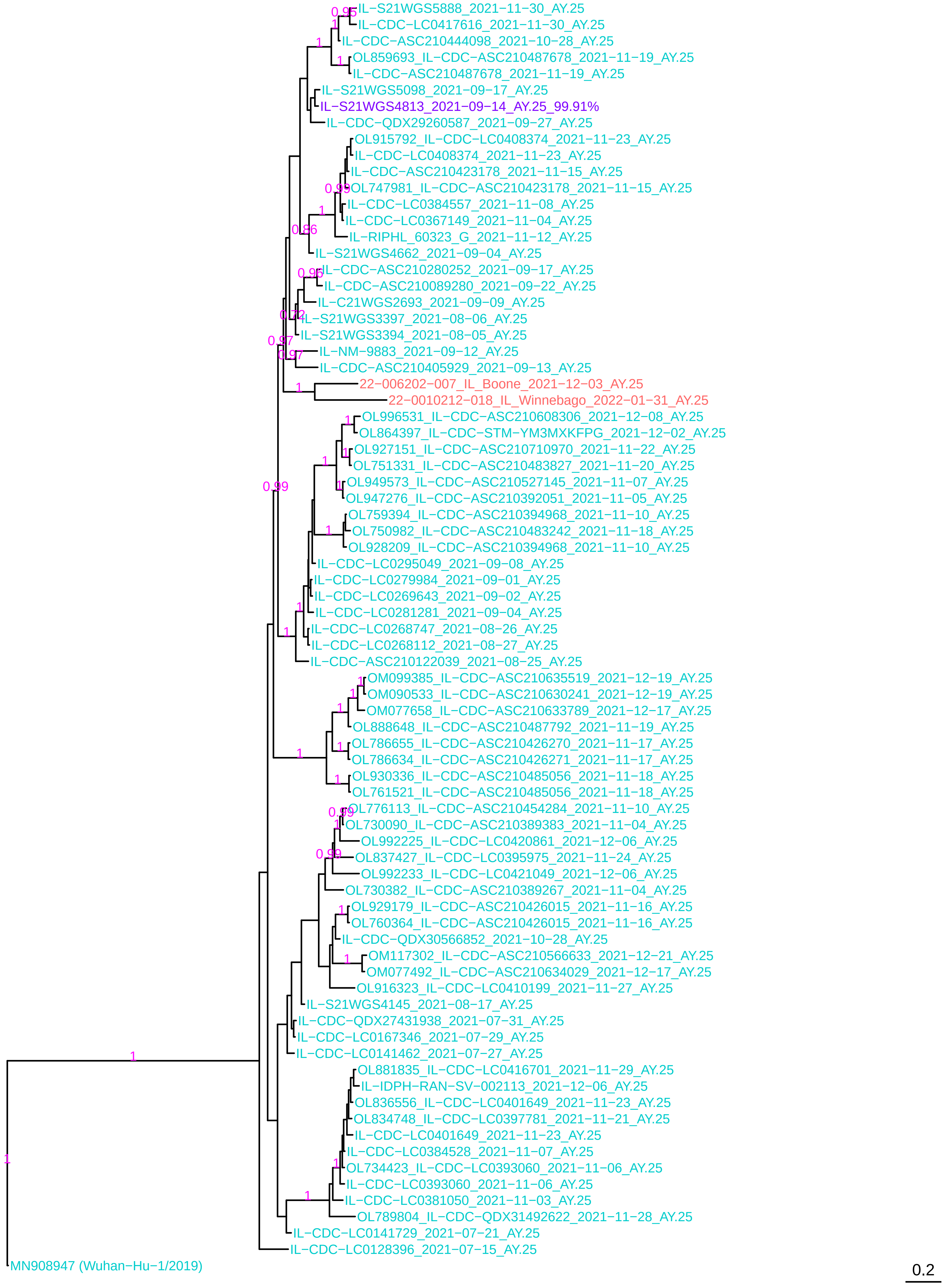
IL−S21WGS5888_2021−11−30_AY.25
IL−CDC−LC0417616_2021−11−30_AY.25
IL−CDC−ASC210444098_2021−10−28_AY.25
OL859693_IL−CDC−ASC210487678_2021−11−19_AY.25
IL−CDC−ASC210487678_2021−11−19_AY.25
IL−S21WGS5098_2021−09−17_AY.25
IL−S21WGS4813_2021−09−14_AY.25_99.91%
IL−CDC−QDX29260587_2021−09−27_AY.25
OL915792_IL−CDC−LC0408374_2021−11−23_AY.25
IL−CDC−LC0408374_2021−11−23_AY.25
IL−CDC−ASC210423178_2021−11−15_AY.25
OL747981_IL−CDC−ASC210423178_2021−11−15_AY.25
IL−CDC−LC0384557_2021−11−08_AY.25
IL−CDC−LC0367149_2021−11−04_AY.25
IL−RIPHL_60323_G_2021−11−12_AY.25
IL−S21WGS4662_2021−09−04_AY.25
IL−CDC−ASC210280252_2021−09−17_AY.25
IL−CDC−ASC210089280_2021−09−22_AY.25
IL−C21WGS2693_2021−09−09_AY.25
IL−S21WGS3397_2021−08−06_AY.25
IL−S21WGS3394_2021−08−05_AY.25
IL−NM−9883_2021−09−12_AY.25
IL−CDC−ASC210405929_2021−09−13_AY.25
22−006202−007_IL_Boone_2021−12−03_AY.25
22−0010212−018_IL_Winnebago_2022−01−31_AY.25
OL996531_IL−CDC−ASC210608306_2021−12−08_AY.25
OL864397_IL−CDC−STM−YM3MXKFPG_2021−12−02_AY.25
OL927151_IL−CDC−ASC210710970_2021−11−22_AY.25
OL751331_IL−CDC−ASC210483827_2021−11−20_AY.25
OL949573_IL−CDC−ASC210527145_2021−11−07_AY.25
OL947276_IL−CDC−ASC210392051_2021−11−05_AY.25
OL759394_IL−CDC−ASC210394968_2021−11−10_AY.25
OL750982_IL−CDC−ASC210483242_2021−11−18_AY.25
OL928209_IL−CDC−ASC210394968_2021−11−10_AY.25
IL−CDC−LC0295049_2021−09−08_AY.25
IL−CDC−LC0279984_2021−09−01_AY.25
IL−CDC−LC0269643_2021−09−02_AY.25
IL−CDC−LC0281281_2021−09−04_AY.25
IL−CDC−LC0268747_2021−08−26_AY.25
IL−CDC−LC0268112_2021−08−27_AY.25
IL−CDC−ASC210122039_2021−08−25_AY.25
OM099385_IL−CDC−ASC210635519_2021−12−19_AY.25
OM090533_IL−CDC−ASC210630241_2021−12−19_AY.25
OM077658_IL−CDC−ASC210633789_2021−12−17_AY.25
OL888648_IL−CDC−ASC210487792_2021−11−19_AY.25
OL786655_IL−CDC−ASC210426270_2021−11−17_AY.25
OL786634_IL−CDC−ASC210426271_2021−11−17_AY.25
OL930336_IL−CDC−ASC210485056_2021−11−18_AY.25
OL761521_IL−CDC−ASC210485056_2021−11−18_AY.25
OL776113_IL−CDC−ASC210454284_2021−11−10_AY.25
OL730090_IL−CDC−ASC210389383_2021−11−04_AY.25
OL992225_IL−CDC−LC0420861_2021−12−06_AY.25
OL837427_IL−CDC−LC0395975_2021−11−24_AY.25
OL992233_IL−CDC−LC0421049_2021−12−06_AY.25
OL730382_IL−CDC−ASC210389267_2021−11−04_AY.25
OL929179_IL−CDC−ASC210426015_2021−11−16_AY.25
OL760364_IL−CDC−ASC210426015_2021−11−16_AY.25
IL−CDC−QDX30566852_2021−10−28_AY.25
OM117302_IL−CDC−ASC210566633_2021−12−21_AY.25
OM077492_IL−CDC−ASC210634029_2021−12−17_AY.25
OL916323_IL−CDC−LC0410199_2021−11−27_AY.25
IL−S21WGS4145_2021−08−17_AY.25
IL−CDC−QDX27431938_2021−07−31_AY.25
IL−CDC−LC0167346_2021−07−29_AY.25
IL−CDC−LC0141462_2021−07−27_AY.25
OL881835_IL−CDC−LC0416701_2021−11−29_AY.25
IL−IDPH−RAN−SV−002113_2021−12−06_AY.25
OL836556_IL−CDC−LC0401649_2021−11−23_AY.25
OL834748_IL−CDC−LC0397781_2021−11−21_AY.25
IL−CDC−LC0401649_2021−11−23_AY.25
IL−CDC−LC0384528_2021−11−07_AY.25
OL734423_IL−CDC−LC0393060_2021−11−06_AY.25
IL−CDC−LC0393060_2021−11−06_AY.25
IL−CDC−LC0381050_2021−11−03_AY.25
OL789804_IL−CDC−QDX31492622_2021−11−28_AY.25
IL−CDC−LC0141729_2021−07−21_AY.25
IL−CDC−LC0128396_2021−07−15_AY.25
MN908947 (Wuhan−Hu−1/2019)
0.2

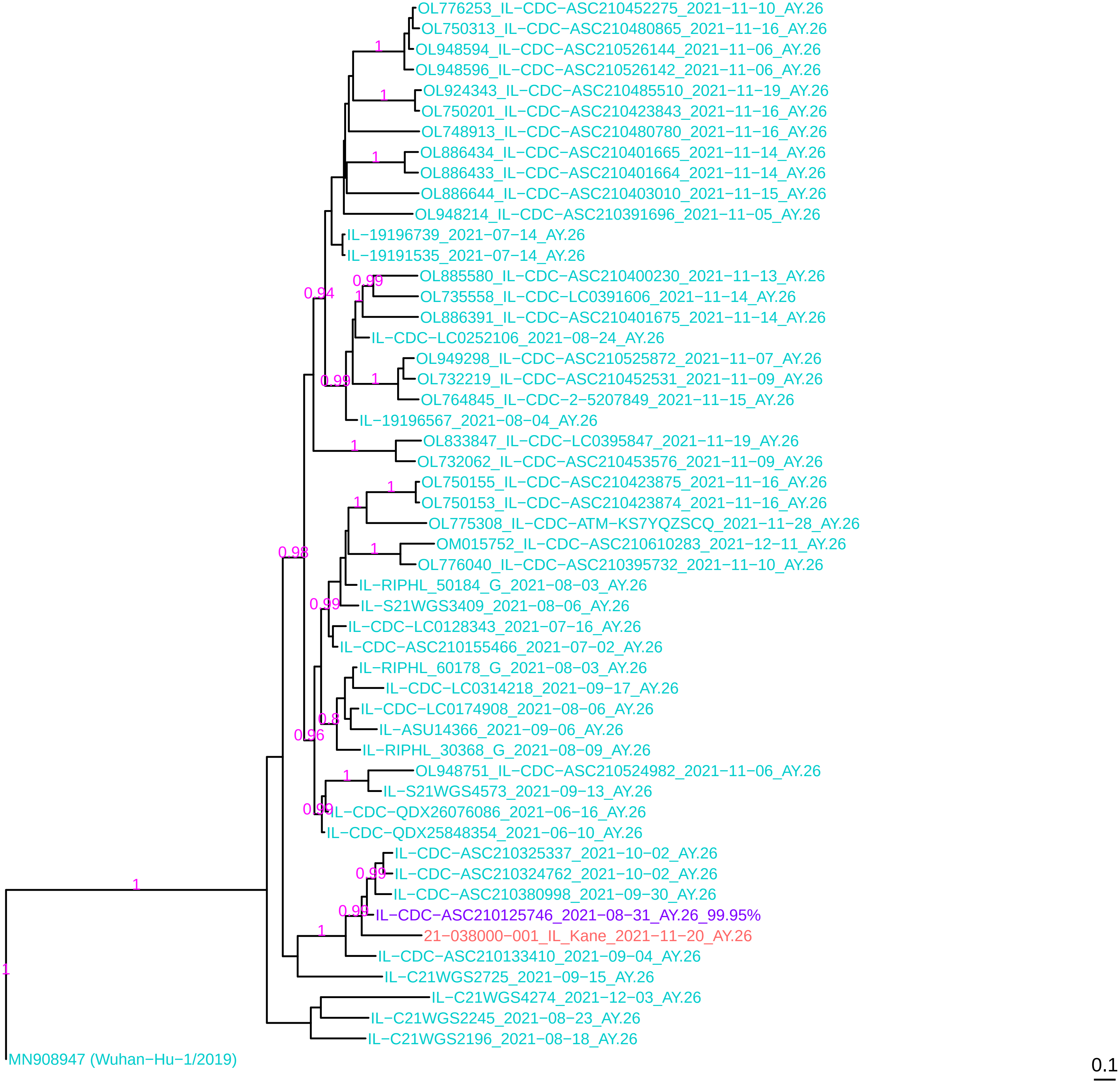
OL776253_IL−CDC−ASC210452275_2021−11−10_AY.26
OL750313_IL−CDC−ASC210480865_2021−11−16_AY.26
OL948594_IL−CDC−ASC210526144_2021−11−06_AY.26
OL948596_IL−CDC−ASC210526142_2021−11−06_AY.26
OL924343_IL−CDC−ASC210485510_2021−11−19_AY.26
OL750201_IL−CDC−ASC210423843_2021−11−16_AY.26
OL748913_IL−CDC−ASC210480780_2021−11−16_AY.26
OL886434_IL−CDC−ASC210401665_2021−11−14_AY.26
OL886433_IL−CDC−ASC210401664_2021−11−14_AY.26
OL886644_IL−CDC−ASC210403010_2021−11−15_AY.26
OL948214_IL−CDC−ASC210391696_2021−11−05_AY.26
IL−19196739_2021−07−14_AY.26
IL−19191535_2021−07−14_AY.26
OL885580_IL−CDC−ASC210400230_2021−11−13_AY.26
OL735558_IL−CDC−LC0391606_2021−11−14_AY.26
OL886391_IL−CDC−ASC210401675_2021−11−14_AY.26
IL−CDC−LC0252106_2021−08−24_AY.26
OL949298_IL−CDC−ASC210525872_2021−11−07_AY.26
OL732219_IL−CDC−ASC210452531_2021−11−09_AY.26
OL764845_IL−CDC−2−5207849_2021−11−15_AY.26
IL−19196567_2021−08−04_AY.26
OL833847_IL−CDC−LC0395847_2021−11−19_AY.26
OL732062_IL−CDC−ASC210453576_2021−11−09_AY.26
OL750155_IL−CDC−ASC210423875_2021−11−16_AY.26
OL750153_IL−CDC−ASC210423874_2021−11−16_AY.26
OL775308_IL−CDC−ATM−KS7YQZSCQ_2021−11−28_AY.26
OM015752_IL−CDC−ASC210610283_2021−12−11_AY.26
OL776040_IL−CDC−ASC210395732_2021−11−10_AY.26
IL−RIPHL_50184_G_2021−08−03_AY.26
IL−S21WGS3409_2021−08−06_AY.26
IL−CDC−LC0128343_2021−07−16_AY.26
IL−CDC−ASC210155466_2021−07−02_AY.26
IL−RIPHL_60178_G_2021−08−03_AY.26
IL−CDC−LC0314218_2021−09−17_AY.26
IL−CDC−LC0174908_2021−08−06_AY.26
IL−ASU14366_2021−09−06_AY.26
IL−RIPHL_30368_G_2021−08−09_AY.26
OL948751_IL−CDC−ASC210524982_2021−11−06_AY.26
IL−S21WGS4573_2021−09−13_AY.26
IL−CDC−QDX26076086_2021−06−16_AY.26
IL−CDC−QDX25848354_2021−06−10_AY.26
IL−CDC−ASC210325337_2021−10−02_AY.26
IL−CDC−ASC210324762_2021−10−02_AY.26
IL−CDC−ASC210380998_2021−09−30_AY.26
IL−CDC−ASC210125746_2021−08−31_AY.26_99.95%
21−038000−001_IL_Kane_2021−11−20_AY.26
IL−CDC−ASC210133410_2021−09−04_AY.26
IL−C21WGS2725_2021−09−15_AY.26
IL−C21WGS4274_2021−12−03_AY.26
IL−C21WGS2245_2021−08−23_AY.26
IL−C21WGS2196_2021−08−18_AY.26
MN908947 (Wuhan−Hu−1/2019)
1
1
1
0.99
1
0.94
0.99
1
1
1
1
0.98
0.99
0.8
0.96
1
0.99
0.99
0.98
1
1
1
0.1

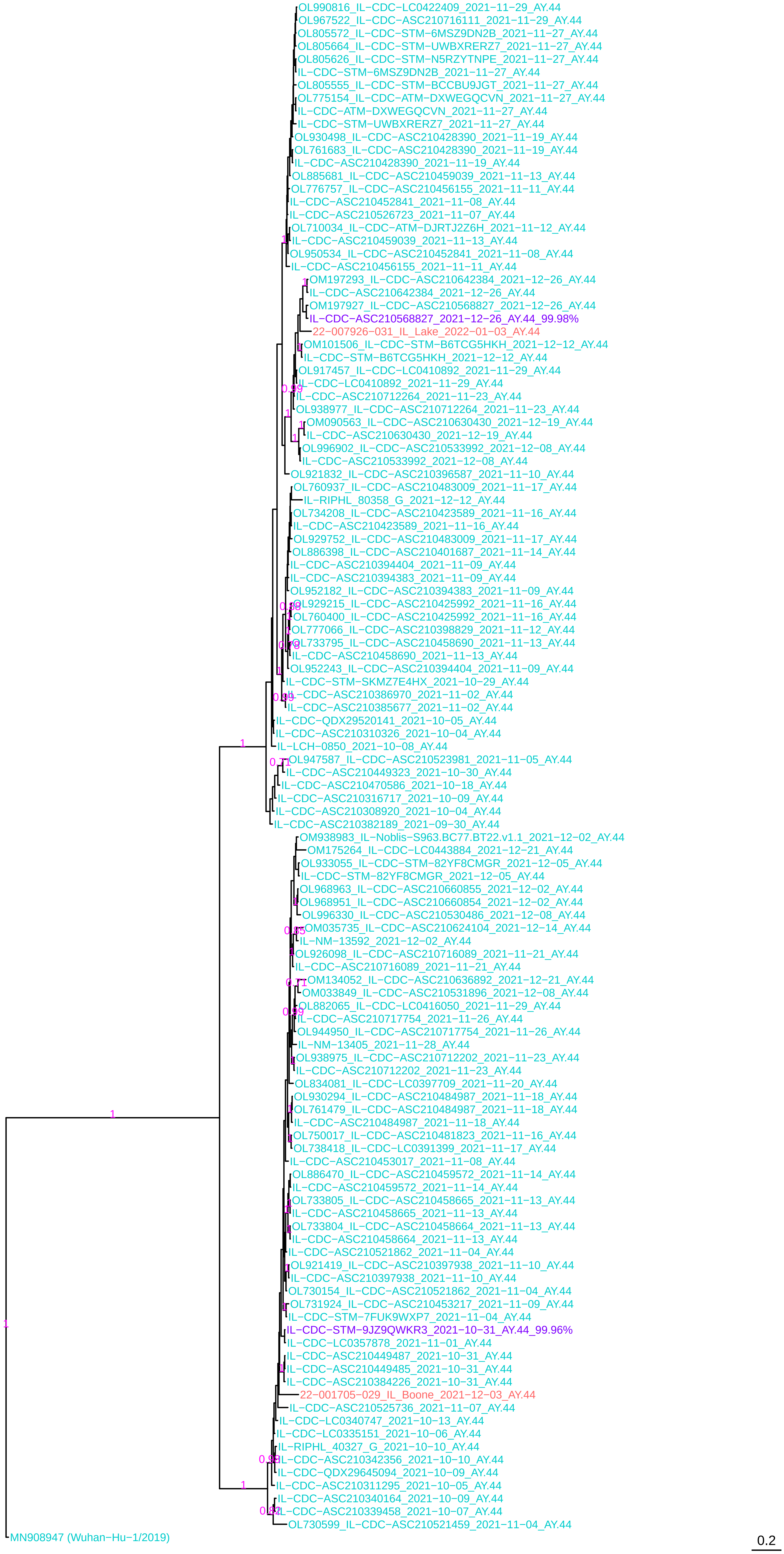
OL990816_IL-CDC-LC0422409_2021-11-29_AY.44
OL967522_IL-CDC-ASC210716111_2021-11-29_AY.44
OL805572_IL-CDC-STM-6MSZ9DN2B_2021-11-27_AY.44
OL805664_IL-CDC-STM-UWBXRERZ7_2021-11-27_AY.44
OL805626_IL-CDC-STM-N5RZYTNPE_2021-11-27_AY.44
IL-CDC-STM-6MSZ9DN2B_2021-11-27_AY.44
OL805555_IL-CDC-STM-BCCBU9JGT_2021-11-27_AY.44
OL775154_IL-CDC-ATM-DXWEGQCVN_2021-11-27_AY.44
IL-CDC-ATM-DXWEGQCVN_2021-11-27_AY.44
IL-CDC-STM-UWBXRERZ7_2021-11-27_AY.44
OL930498_IL-CDC-ASC210428390_2021-11-19_AY.44
OL761683_IL-CDC-ASC210428390_2021-11-19_AY.44
IL-CDC-ASC210428390_2021-11-19_AY.44
OL885681_IL-CDC-ASC210459039_2021-11-13_AY.44
OL776757_IL-CDC-ASC210456155_2021-11-11_AY.44
IL-CDC-ASC210452841_2021-11-08_AY.44
IL-CDC-ASC210526723_2021-11-07_AY.44
OL710034_IL-CDC-ATM-DJRTJ2Z6H_2021-11-12_AY.44
IL-CDC-ASC210459039_2021-11-13_AY.44
OL950534_IL-CDC-ASC210452841_2021-11-08_AY.44
IL-CDC-ASC210456155_2021-11-11_AY.44
OM197293_IL-CDC-ASC210642384_2021-12-26_AY.44
IL-CDC-ASC210642384_2021-12-26_AY.44
OM197927_IL-CDC-ASC210568827_2021-12-26_AY.44
IL-CDC-ASC210568827_2021-12-26_AY.44_99.98%
22-007926-031_IL_Lake_2022-01-03_AY.44
OM101506_IL-CDC-STM-B6TCG5HKH_2021-12-12_AY.44
IL-CDC-STM-B6TCG5HKH_2021-12-12_AY.44
OL917457_IL-CDC-LC0410892_2021-11-29_AY.44
IL-CDC-LC0410892_2021-11-29_AY.44
IL-CDC-ASC210712264_2021-11-23_AY.44
OL938977_IL-CDC-ASC210712264_2021-11-23_AY.44
OM090563_IL-CDC-ASC210630430_2021-12-19_AY.44
IL-CDC-ASC210630430_2021-12-19_AY.44
OL996902_IL-CDC-ASC210533992_2021-12-08_AY.44
IL-CDC-ASC210533992_2021-12-08_AY.44
OL921832_IL-CDC-ASC210396587_2021-11-10_AY.44
OL760937_IL-CDC-ASC210483009_2021-11-17_AY.44
IL-RIPHL_80358_G_2021-12-12_AY.44
OL734208_IL-CDC-ASC210423589_2021-11-16_AY.44
IL-CDC-ASC210423589_2021-11-16_AY.44
OL929752_IL-CDC-ASC210483009_2021-11-17_AY.44
OL886398_IL-CDC-ASC210401687_2021-11-14_AY.44
IL-CDC-ASC210394404_2021-11-09_AY.44
IL-CDC-ASC210394383_2021-11-09_AY.44
OL952182_IL-CDC-ASC210394383_2021-11-09_AY.44
OL929215_IL-CDC-ASC210425992_2021-11-16_AY.44
OL760400_IL-CDC-ASC210425992_2021-11-16_AY.44
OL777066_IL-CDC-ASC210398829_2021-11-12_AY.44
OL733795_IL-CDC-ASC210458690_2021-11-13_AY.44
IL-CDC-ASC210458690_2021-11-13_AY.44
OL952243_IL-CDC-ASC210394404_2021-11-09_AY.44
IL-CDC-STM-SKMZ7E4HX_2021-10-29_AY.44
IL-CDC-ASC210386970_2021-11-02_AY.44
IL-CDC-ASC210385677_2021-11-02_AY.44
IL-CDC-QDX29520141_2021-10-05_AY.44
IL-CDC-ASC210310326_2021-10-04_AY.44
IL-LCH-0850_2021-10-08_AY.44
OL947587_IL-CDC-ASC210523981_2021-11-05_AY.44
IL-CDC-ASC210449323_2021-10-30_AY.44
IL-CDC-ASC210470586_2021-10-18_AY.44
IL-CDC-ASC210316717_2021-10-09_AY.44
IL-CDC-ASC210308920_2021-10-04_AY.44
IL-CDC-ASC210382189_2021-09-30_AY.44
OM938983_IL-Noblis-S963.BC77.BT22.v1.1_2021-12-02_AY.44
OM175264_IL-CDC-LC0443884_2021-12-21_AY.44
OL933055_IL-CDC-STM-82YF8CMGR_2021-12-05_AY.44
IL-CDC-STM-82YF8CMGR_2021-12-05_AY.44
OL968963_IL-CDC-ASC210660855_2021-12-02_AY.44
OL968951_IL-CDC-ASC210660854_2021-12-02_AY.44
OL996330_IL-CDC-ASC210530486_2021-12-08_AY.44
OM035735_IL-CDC-ASC210624104_2021-12-14_AY.44
IL-NM-13592_2021-12-02_AY.44
OL926098_IL-CDC-ASC210716089_2021-11-21_AY.44
IL-CDC-ASC210716089_2021-11-21_AY.44
OM134052_IL-CDC-ASC210636892_2021-12-21_AY.44
OM033849_IL-CDC-ASC210531896_2021-12-08_AY.44
OL882065_IL-CDC-LC0416050_2021-11-29_AY.44
IL-CDC-ASC210717754_2021-11-26_AY.44
OL944950_IL-CDC-ASC210717754_2021-11-26_AY.44
IL-NM-13405_2021-11-28_AY.44
OL938975_IL-CDC-ASC210712202_2021-11-23_AY.44
IL-CDC-ASC210712202_2021-11-23_AY.44
OL834081_IL-CDC-LC0397709_2021-11-20_AY.44
OL930294_IL-CDC-ASC210484987_2021-11-18_AY.44
OL761479_IL-CDC-ASC210484987_2021-11-18_AY.44
IL-CDC-ASC210484987_2021-11-18_AY.44
OL750017_IL-CDC-ASC210481823_2021-11-16_AY.44
OL738418_IL-CDC-LC0391399_2021-11-17_AY.44
IL-CDC-ASC210453017_2021-11-08_AY.44
OL886470_IL-CDC-ASC210459572_2021-11-14_AY.44
IL-CDC-ASC210459572_2021-11-14_AY.44
OL733805_IL-CDC-ASC210458665_2021-11-13_AY.44
IL-CDC-ASC210458665_2021-11-13_AY.44
OL733804_IL-CDC-ASC210458664_2021-11-13_AY.44
IL-CDC-ASC210458664_2021-11-13_AY.44
IL-CDC-ASC210521862_2021-11-04_AY.44
OL921419_IL-CDC-ASC210397938_2021-11-10_AY.44
IL-CDC-ASC210397938_2021-11-10_AY.44
OL730154_IL-CDC-ASC210521862_2021-11-04_AY.44
OL731924_IL-CDC-ASC210453217_2021-11-09_AY.44
IL-CDC-STM-7FUK9WXP7_2021-11-04_AY.44
IL-CDC-STM-9JZ9QWKR3_2021-10-31_AY.44_99.96%
IL-CDC-LC0357878_2021-11-01_AY.44
IL-CDC-ASC210449487_2021-10-31_AY.44
IL-CDC-ASC210449485_2021-10-31_AY.44
IL-CDC-ASC210384226_2021-10-31_AY.44
22-001705-029_IL_Boone_2021-12-03_AY.44
IL-CDC-ASC210525736_2021-11-07_AY.44
IL-CDC-LC0340747_2021-10-13_AY.44
IL-CDC-LC0335151_2021-10-06_AY.44
IL-RIPHL_40327_G_2021-10-10_AY.44
IL-CDC-ASC210342356_2021-10-10_AY.44
IL-CDC-QDX29645094_2021-10-09_AY.44
IL-CDC-ASC210311295_2021-10-05_AY.44
IL-CDC-ASC210340164_2021-10-09_AY.44
IL-CDC-ASC210339458_2021-10-07_AY.44
OL730599_IL-CDC-ASC210521459_2021-11-04_AY.44
MN908947 (Wuhan-Hu-1/2019)
0.2

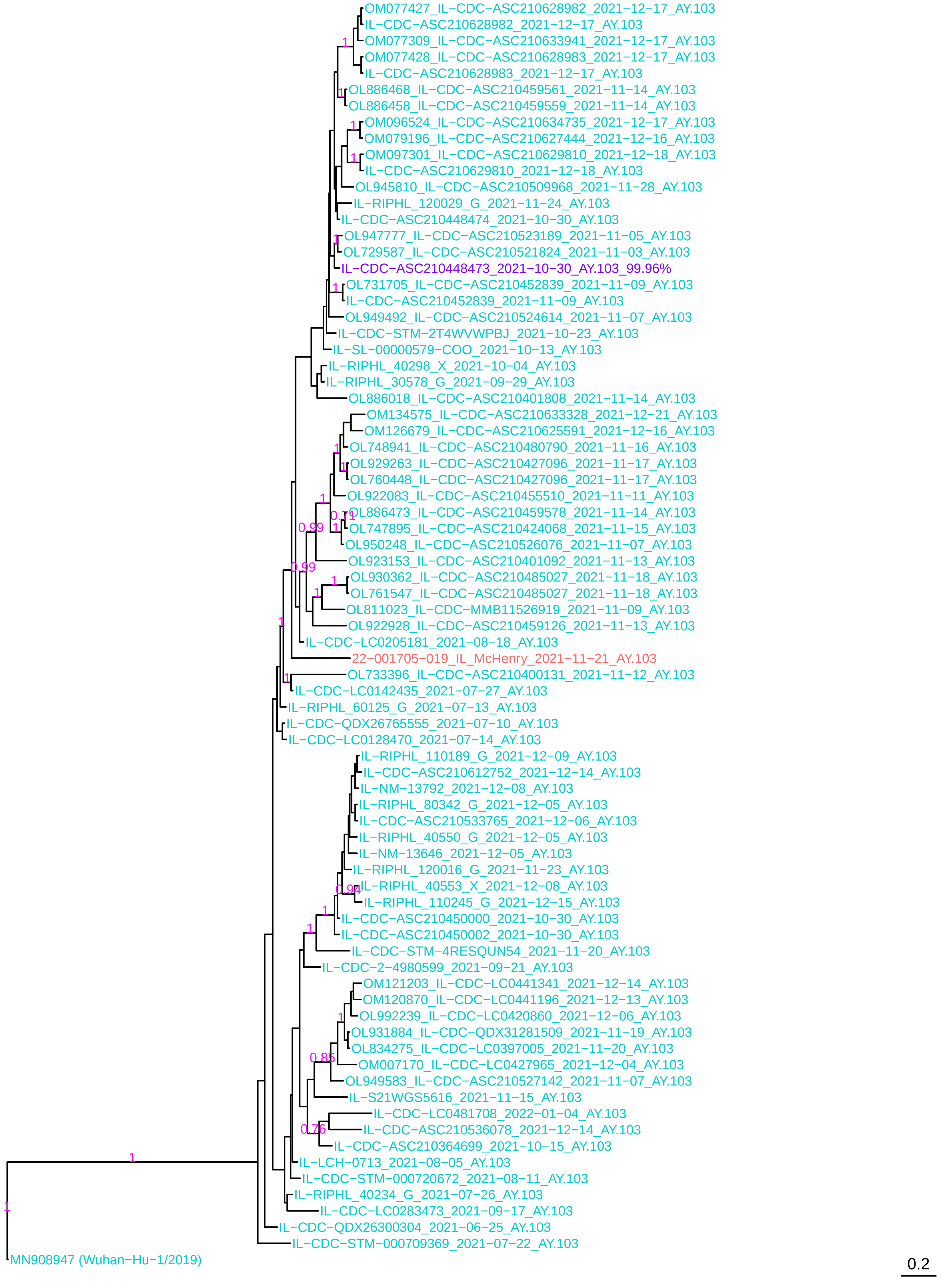
OM077427_IL−CDC−ASC210628982_2021−12−17_AY.103
IL−CDC−ASC210628982_2021−12−17_AY.103
OM077309_IL−CDC−ASC210633941_2021−12−17_AY.103
OM077428_IL−CDC−ASC210628983_2021−12−17_AY.103
IL−CDC−ASC210628983_2021−12−17_AY.103
OL886468_IL−CDC−ASC210459561_2021−11−14_AY.103
OL886458_IL−CDC−ASC210459559_2021−11−14_AY.103
OM096524_IL−CDC−ASC210634735_2021−12−17_AY.103
OM079196_IL−CDC−ASC210627444_2021−12−16_AY.103
OM097301_IL−CDC−ASC210629810_2021−12−18_AY.103
IL−CDC−ASC210629810_2021−12−18_AY.103
OL945810_IL−CDC−ASC210509968_2021−11−28_AY.103
IL−RIPHL_120029_G_2021−11−24_AY.103
IL−CDC−ASC210448474_2021−10−30_AY.103
OL947777_IL−CDC−ASC210523189_2021−11−05_AY.103
OL729587_IL−CDC−ASC210521824_2021−11−03_AY.103
IL−CDC−ASC210448473_2021−10−30_AY.103_99.96%
OL731705_IL−CDC−ASC210452839_2021−11−09_AY.103
IL−CDC−ASC210452839_2021−11−09_AY.103
OL949492_IL−CDC−ASC210524614_2021−11−07_AY.103
IL−CDC−STM−2T4WVWPBJ_2021−10−23_AY.103
IL−SL−00000579−COO_2021−10−13_AY.103
IL−RIPHL_40298_X_2021−10−04_AY.103
IL−RIPHL_30578_G_2021−09−29_AY.103
OL886018_IL−CDC−ASC210401808_2021−11−14_AY.103
OM134575_IL−CDC−ASC210633328_2021−12−21_AY.103
OM126679_IL−CDC−ASC210625591_2021−12−16_AY.103
OL748941_IL−CDC−ASC210480790_2021−11−16_AY.103
OL929263_IL−CDC−ASC210427096_2021−11−17_AY.103
OL760448_IL−CDC−ASC210427096_2021−11−17_AY.103
OL922083_IL−CDC−ASC210455510_2021−11−11_AY.103
OL886473_IL−CDC−ASC210459578_2021−11−14_AY.103
OL747895_IL−CDC−ASC210424068_2021−11−15_AY.103
OL950248_IL−CDC−ASC210526076_2021−11−07_AY.103
OL923153_IL−CDC−ASC210401092_2021−11−13_AY.103
OL930362_IL−CDC−ASC210485027_2021−11−18_AY.103
OL761547_IL−CDC−ASC210485027_2021−11−18_AY.103
OL811023_IL−CDC−MMB11526919_2021−11−09_AY.103
OL922928_IL−CDC−ASC210459126_2021−11−13_AY.103
IL−CDC−LC0205181_2021−08−18_AY.103
22−001705−019_IL_McHenry_2021−11−21_AY.103
OL733396_IL−CDC−ASC210400131_2021−11−12_AY.103
IL−CDC−LC0142435_2021−07−27_AY.103
IL−RIPHL_60125_G_2021−07−13_AY.103
IL−CDC−QDX26765555_2021−07−10_AY.103
IL−CDC−LC0128470_2021−07−14_AY.103
IL−RIPHL_110189_G_2021−12−09_AY.103
IL−CDC−ASC210612752_2021−12−14_AY.103
IL−NM−13792_2021−12−08_AY.103
IL−RIPHL_80342_G_2021−12−05_AY.103
IL−CDC−ASC210533765_2021−12−06_AY.103
IL−RIPHL_40550_G_2021−12−05_AY.103
IL−NM−13646_2021−12−05_AY.103
IL−RIPHL_120016_G_2021−11−23_AY.103
IL−RIPHL_40553_X_2021−12−08_AY.103
IL−RIPHL_110245_G_2021−12−15_AY.103
IL−CDC−ASC210450000_2021−10−30_AY.103
IL−CDC−ASC210450002_2021−10−30_AY.103
IL−CDC−STM−4RESQUN54_2021−11−20_AY.103
IL−CDC−2−4980599_2021−09−21_AY.103
OM121203_IL−CDC−LC0441341_2021−12−14_AY.103
OM120870_IL−CDC−LC0441196_2021−12−13_AY.103
OL992239_IL−CDC−LC0420860_2021−12−06_AY.103
OL931884_IL−CDC−QDX31281509_2021−11−19_AY.103
OL834275_IL−CDC−LC0397005_2021−11−20_AY.103
OM007170_IL−CDC−LC0427965_2021−12−04_AY.103
OL949583_IL−CDC−ASC210527142_2021−11−07_AY.103
IL−S21WGS5616_2021−11−15_AY.103
IL−CDC−LC0481708_2022−01−04_AY.103
IL−CDC−ASC210536078_2021−12−14_AY.103
IL−CDC−ASC210364699_2021−10−15_AY.103
IL−LCH−0713_2021−08−05_AY.103
IL−CDC−STM−000720672_2021−08−11_AY.103
IL−RIPHL_40234_G_2021−07−26_AY.103
IL−CDC−LC0283473_2021−09−17_AY.103
IL−CDC−QDX26300304_2021−06−25_AY.103
IL−CDC−STM−000709369_2021−07−22_AY.103
MN908947 (Wuhan−Hu−1/2019)
0.2

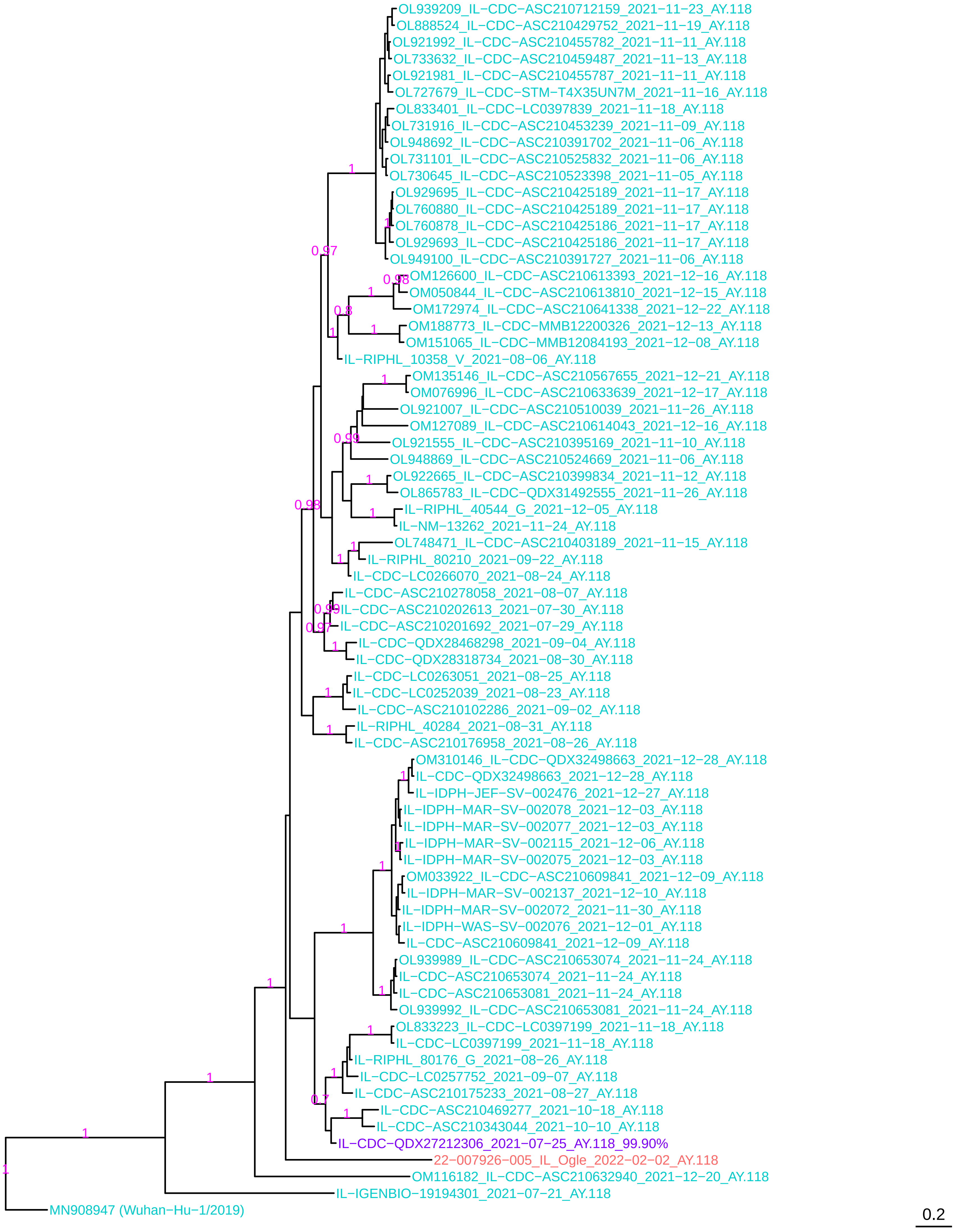
OL939209_IL−CDC−ASC210712159_2021−11−23_AY.118
OL888524_IL−CDC−ASC210429752_2021−11−19_AY.118
OL921992_IL−CDC−ASC210455782_2021−11−11_AY.118
OL733632_IL−CDC−ASC210459487_2021−11−13_AY.118
OL921981_IL−CDC−ASC210455787_2021−11−11_AY.118
OL727679_IL−CDC−STM−T4X35UN7M_2021−11−16_AY.118
OL833401_IL−CDC−LC0397839_2021−11−18_AY.118
OL731916_IL−CDC−ASC210453239_2021−11−09_AY.118
OL948692_IL−CDC−ASC210391702_2021−11−06_AY.118
OL731101_IL−CDC−ASC210525832_2021−11−06_AY.118
OL730645_IL−CDC−ASC210523398_2021−11−05_AY.118
OL929695_IL−CDC−ASC210425189_2021−11−17_AY.118
OL760880_IL−CDC−ASC210425189_2021−11−17_AY.118
OL760878_IL−CDC−ASC210425186_2021−11−17_AY.118
OL929693_IL−CDC−ASC210425186_2021−11−17_AY.118
OL949100_IL−CDC−ASC210391727_2021−11−06_AY.118
OM126600_IL−CDC−ASC210613393_2021−12−16_AY.118
OM050844_IL−CDC−ASC210613810_2021−12−15_AY.118
OM172974_IL−CDC−ASC210641338_2021−12−22_AY.118
OM188773_IL−CDC−MMB12200326_2021−12−13_AY.118
OM151065_IL−CDC−MMB12084193_2021−12−08_AY.118
IL−RIPHL_10358_V_2021−08−06_AY.118
OM135146_IL−CDC−ASC210567655_2021−12−21_AY.118
OM076996_IL−CDC−ASC210633639_2021−12−17_AY.118
OL921007_IL−CDC−ASC210510039_2021−11−26_AY.118
OM127089_IL−CDC−ASC210614043_2021−12−16_AY.118
OL921555_IL−CDC−ASC210395169_2021−11−10_AY.118
OL948869_IL−CDC−ASC210524669_2021−11−06_AY.118
OL922665_IL−CDC−ASC210399834_2021−11−12_AY.118
OL865783_IL−CDC−QDX31492555_2021−11−26_AY.118
IL−RIPHL_40544_G_2021−12−05_AY.118
IL−NM−13262_2021−11−24_AY.118
OL748471_IL−CDC−ASC210403189_2021−11−15_AY.118
IL−RIPHL_80210_2021−09−22_AY.118
IL−CDC−LC0266070_2021−08−24_AY.118
IL−CDC−ASC210278058_2021−08−07_AY.118
IL−CDC−ASC210202613_2021−07−30_AY.118
IL−CDC−ASC210201692_2021−07−29_AY.118
IL−CDC−QDX28468298_2021−09−04_AY.118
IL−CDC−QDX28318734_2021−08−30_AY.118
IL−CDC−LC0263051_2021−08−25_AY.118
IL−CDC−LC0252039_2021−08−23_AY.118
IL−CDC−ASC210102286_2021−09−02_AY.118
IL−RIPHL_40284_2021−08−31_AY.118
IL−CDC−ASC210176958_2021−08−26_AY.118
OM310146_IL−CDC−QDX32498663_2021−12−28_AY.118
IL−CDC−QDX32498663_2021−12−28_AY.118
IL−IDPH−JEF−SV−002476_2021−12−27_AY.118
IL−IDPH−MAR−SV−002078_2021−12−03_AY.118
IL−IDPH−MAR−SV−002077_2021−12−03_AY.118
IL−IDPH−MAR−SV−002115_2021−12−06_AY.118
IL−IDPH−MAR−SV−002075_2021−12−03_AY.118
OM033922_IL−CDC−ASC210609841_2021−12−09_AY.118
IL−IDPH−MAR−SV−002137_2021−12−10_AY.118
IL−IDPH−MAR−SV−002072_2021−11−30_AY.118
IL−IDPH−WAS−SV−002076_2021−12−01_AY.118
IL−CDC−ASC210609841_2021−12−09_AY.118
OL939989_IL−CDC−ASC210653074_2021−11−24_AY.118
IL−CDC−ASC210653074_2021−11−24_AY.118
IL−CDC−ASC210653081_2021−11−24_AY.118
OL939992_IL−CDC−ASC210653081_2021−11−24_AY.118
OL833223_IL−CDC−LC0397199_2021−11−18_AY.118
IL−CDC−LC0397199_2021−11−18_AY.118
IL−RIPHL_80176_G_2021−08−26_AY.118
IL−CDC−LC0257752_2021−09−07_AY.118
IL−CDC−ASC210175233_2021−08−27_AY.118
IL−CDC−ASC210469277_2021−10−18_AY.118
IL−CDC−ASC210343044_2021−10−10_AY.118
IL−CDC−QDX27212306_2021−07−25_AY.118_99.90%
22−007926−005_IL_Ogle_2022−02−02_AY.118
OM116182_IL−CDC−ASC210632940_2021−12−20_AY.118
IL−IGENBIO−19194301_2021−07−21_AY.118
MN908947 (Wuhan−Hu−1/2019)
0.2

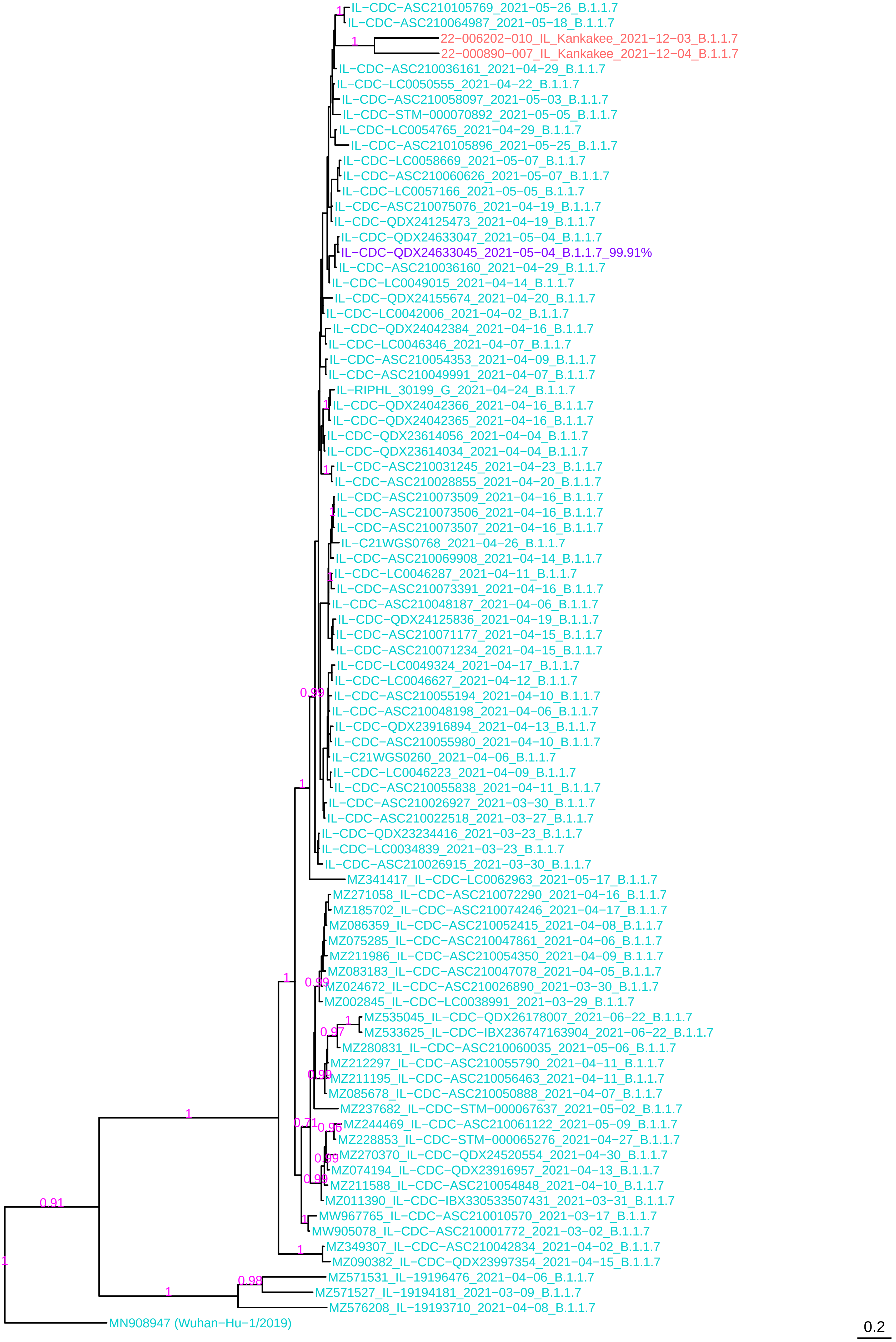
IL-CDC-ASC210105769_2021-05-26_B.1.1.7
IL-CDC-ASC210064987_2021-05-18_B.1.1.7
22-006202-010_IL_Kankakee_2021-12-03_B.1.1.7
22-000890-007_IL_Kankakee_2021-12-04_B.1.1.7
IL-CDC-ASC210036161_2021-04-29_B.1.1.7
IL-CDC-LC0050555_2021-04-22_B.1.1.7
IL-CDC-ASC210058097_2021-05-03_B.1.1.7
IL-CDC-STM-000070892_2021-05-05_B.1.1.7
IL-CDC-LC0054765_2021-04-29_B.1.1.7
IL-CDC-ASC210105896_2021-05-25_B.1.1.7
IL-CDC-LC0058669_2021-05-07_B.1.1.7
IL-CDC-ASC210060626_2021-05-07_B.1.1.7
IL-CDC-LC0057166_2021-05-05_B.1.1.7
IL-CDC-ASC210075076_2021-04-19_B.1.1.7
IL-CDC-QDX24125473_2021-04-19_B.1.1.7
IL-CDC-QDX24633047_2021-05-04_B.1.1.7
IL-CDC-QDX24633045_2021-05-04_B.1.1.7_99.91%
IL-CDC-ASC210036160_2021-04-29_B.1.1.7
IL-CDC-LC0049015_2021-04-14_B.1.1.7
IL-CDC-QDX24155674_2021-04-20_B.1.1.7
IL-CDC-LC0042006_2021-04-02_B.1.1.7
IL-CDC-QDX24042384_2021-04-16_B.1.1.7
IL-CDC-LC0046346_2021-04-07_B.1.1.7
IL-CDC-ASC210054353_2021-04-09_B.1.1.7
IL-CDC-ASC210049991_2021-04-07_B.1.1.7
IL-RIPHL_30199_G_2021-04-24_B.1.1.7
IL-CDC-QDX24042366_2021-04-16_B.1.1.7
IL-CDC-QDX24042365_2021-04-16_B.1.1.7
IL-CDC-QDX23614056_2021-04-04_B.1.1.7
IL-CDC-QDX23614034_2021-04-04_B.1.1.7
IL-CDC-ASC210031245_2021-04-23_B.1.1.7
IL-CDC-ASC210028855_2021-04-20_B.1.1.7
IL-CDC-ASC210073509_2021-04-16_B.1.1.7
IL-CDC-ASC210073506_2021-04-16_B.1.1.7
IL-CDC-ASC210073507_2021-04-16_B.1.1.7
IL-C21WGS0768_2021-04-26_B.1.1.7
IL-CDC-ASC210069908_2021-04-14_B.1.1.7
IL-CDC-LC0046287_2021-04-11_B.1.1.7
IL-CDC-ASC210073391_2021-04-16_B.1.1.7
IL-CDC-ASC210048187_2021-04-06_B.1.1.7
IL-CDC-QDX24125836_2021-04-19_B.1.1.7
IL-CDC-ASC210071177_2021-04-15_B.1.1.7
IL-CDC-ASC210071234_2021-04-15_B.1.1.7
IL-CDC-LC0049324_2021-04-17_B.1.1.7
IL-CDC-LC0046627_2021-04-12_B.1.1.7
IL-CDC-ASC210055194_2021-04-10_B.1.1.7
IL-CDC-ASC210048198_2021-04-06_B.1.1.7
IL-CDC-QDX23916894_2021-04-13_B.1.1.7
IL-CDC-ASC210055980_2021-04-10_B.1.1.7
IL-C21WGS0260_2021-04-06_B.1.1.7
IL-CDC-LC0046223_2021-04-09_B.1.1.7
IL-CDC-ASC210055838_2021-04-11_B.1.1.7
IL-CDC-ASC210026927_2021-03-30_B.1.1.7
IL-CDC-ASC210022518_2021-03-27_B.1.1.7
IL-CDC-QDX23234416_2021-03-23_B.1.1.7
IL-CDC-LC0034839_2021-03-23_B.1.1.7
IL-CDC-ASC210026915_2021-03-30_B.1.1.7
MZ341417_IL-CDC-LC0062963_2021-05-17_B.1.1.7
MZ271058_IL-CDC-ASC210072290_2021-04-16_B.1.1.7
MZ185702_IL-CDC-ASC210074246_2021-04-17_B.1.1.7
MZ086359_IL-CDC-ASC210052415_2021-04-08_B.1.1.7
MZ075285_IL-CDC-ASC210047861_2021-04-06_B.1.1.7
MZ211986_IL-CDC-ASC210054350_2021-04-09_B.1.1.7
MZ083183_IL-CDC-ASC210047078_2021-04-05_B.1.1.7
MZ024672_IL-CDC-ASC210026890_2021-03-30_B.1.1.7
MZ002845_IL-CDC-LC0038991_2021-03-29_B.1.1.7
MZ535045_IL-CDC-QDX26178007_2021-06-22_B.1.1.7
MZ533625_IL-CDC-IBX236747163904_2021-06-22_B.1.1.7
MZ280831_IL-CDC-ASC210060035_2021-05-06_B.1.1.7
MZ212297_IL-CDC-ASC210055790_2021-04-11_B.1.1.7
MZ211195_IL-CDC-ASC210056463_2021-04-11_B.1.1.7
MZ085678_IL-CDC-ASC210050888_2021-04-07_B.1.1.7
MZ237682_IL-CDC-STM-000067637_2021-05-02_B.1.1.7
MZ244469_IL-CDC-ASC210061122_2021-05-09_B.1.1.7
MZ228853_IL-CDC-STM-000065276_2021-04-27_B.1.1.7
MZ270370_IL-CDC-QDX24520554_2021-04-30_B.1.1.7
MZ074194_IL-CDC-QDX23916957_2021-04-13_B.1.1.7
MZ211588_IL-CDC-ASC210054848_2021-04-10_B.1.1.7
MZ011390_IL-CDC-IBX330533507431_2021-03-31_B.1.1.7
MW967765_IL-CDC-ASC210010570_2021-03-17_B.1.1.7
MW905078_IL-CDC-ASC210001772_2021-03-02_B.1.1.7
MZ349307_IL-CDC-ASC210042834_2021-04-02_B.1.1.7
MZ090382_IL-CDC-QDX23997354_2021-04-15_B.1.1.7
MZ571531_IL-19196476_2021-04-06_B.1.1.7
MZ571527_IL-19194181_2021-03-09_B.1.1.7
MZ576208_IL-19193710_2021-04-08_B.1.1.7
MN908947 (Wuhan-Hu-1/2019)
0.2

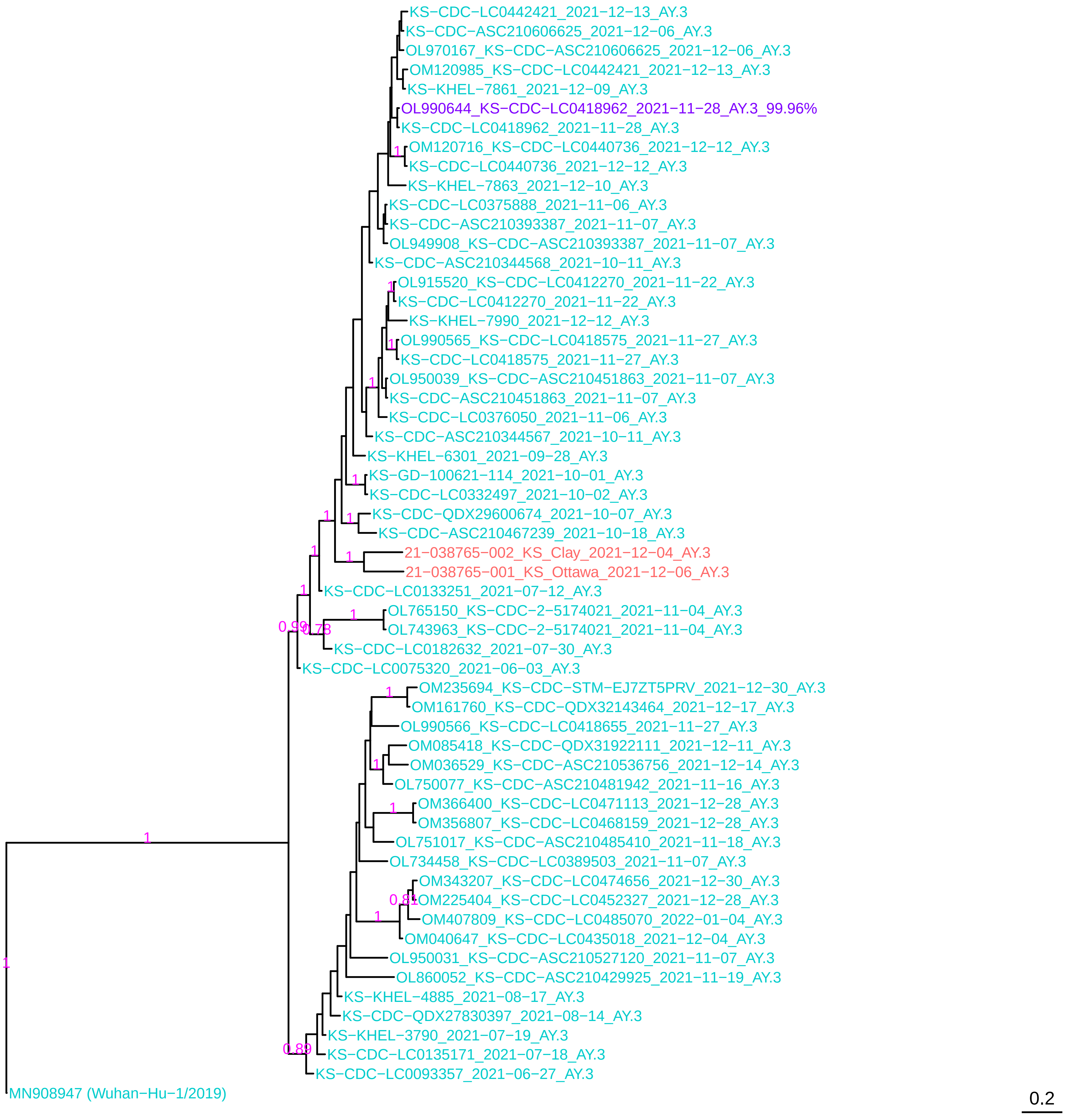
KS−CDC−LC0442421_2021−12−13_AY.3
KS−CDC−ASC210606625_2021−12−06_AY.3
OL970167_KS−CDC−ASC210606625_2021−12−06_AY.3
OM120985_KS−CDC−LC0442421_2021−12−13_AY.3
KS−KHEL−7861_2021−12−09_AY.3
OL990644_KS−CDC−LC0418962_2021−11−28_AY.3_99.96%
KS−CDC−LC0418962_2021−11−28_AY.3
OM120716_KS−CDC−LC0440736_2021−12−12_AY.3
KS−CDC−LC0440736_2021−12−12_AY.3
KS−KHEL−7863_2021−12−10_AY.3
KS−CDC−LC0375888_2021−11−06_AY.3
KS−CDC−ASC210393387_2021−11−07_AY.3
OL949908_KS−CDC−ASC210393387_2021−11−07_AY.3
KS−CDC−ASC210344568_2021−10−11_AY.3
OL915520_KS−CDC−LC0412270_2021−11−22_AY.3
KS−CDC−LC0412270_2021−11−22_AY.3
KS−KHEL−7990_2021−12−12_AY.3
OL990565_KS−CDC−LC0418575_2021−11−27_AY.3
KS−CDC−LC0418575_2021−11−27_AY.3
OL950039_KS−CDC−ASC210451863_2021−11−07_AY.3
KS−CDC−ASC210451863_2021−11−07_AY.3
KS−CDC−LC0376050_2021−11−06_AY.3
KS−CDC−ASC210344567_2021−10−11_AY.3
KS−KHEL−6301_2021−09−28_AY.3
KS−GD−100621−114_2021−10−01_AY.3
KS−CDC−LC0332497_2021−10−02_AY.3
KS−CDC−QDX29600674_2021−10−07_AY.3
KS−CDC−ASC210467239_2021−10−18_AY.3
21−038765−002_KS_Clay_2021−12−04_AY.3
21−038765−001_KS_Ottawa_2021−12−06_AY.3
KS−CDC−LC0133251_2021−07−12_AY.3
OL765150_KS−CDC−2−5174021_2021−11−04_AY.3
OL743963_KS−CDC−2−5174021_2021−11−04_AY.3
KS−CDC−LC0182632_2021−07−30_AY.3
KS−CDC−LC0075320_2021−06−03_AY.3
OM235694_KS−CDC−STM−EJ7ZT5PRV_2021−12−30_AY.3
OM161760_KS−CDC−QDX32143464_2021−12−17_AY.3
OL990566_KS−CDC−LC0418655_2021−11−27_AY.3
OM085418_KS−CDC−QDX31922111_2021−12−11_AY.3
OM036529_KS−CDC−ASC210536756_2021−12−14_AY.3
OL750077_KS−CDC−ASC210481942_2021−11−16_AY.3
OM366400_KS−CDC−LC0471113_2021−12−28_AY.3
OM356807_KS−CDC−LC0468159_2021−12−28_AY.3
OL751017_KS−CDC−ASC210485410_2021−11−18_AY.3
OL734458_KS−CDC−LC0389503_2021−11−07_AY.3
OM343207_KS−CDC−LC0474656_2021−12−30_AY.3
OM225404_KS−CDC−LC0452327_2021−12−28_AY.3
OM407809_KS−CDC−LC0485070_2022−01−04_AY.3
OM040647_KS−CDC−LC0435018_2021−12−04_AY.3
OL950031_KS−CDC−ASC210527120_2021−11−07_AY.3
OL860052_KS−CDC−ASC210429925_2021−11−19_AY.3
KS−KHEL−4885_2021−08−17_AY.3
KS−CDC−QDX27830397_2021−08−14_AY.3
KS−KHEL−3790_2021−07−19_AY.3
KS−CDC−LC0135171_2021−07−18_AY.3
KS−CDC−LC0093357_2021−06−27_AY.3
MN908947 (Wuhan−Hu−1/2019)
0.99
0.78
0.81
0.89
0.2

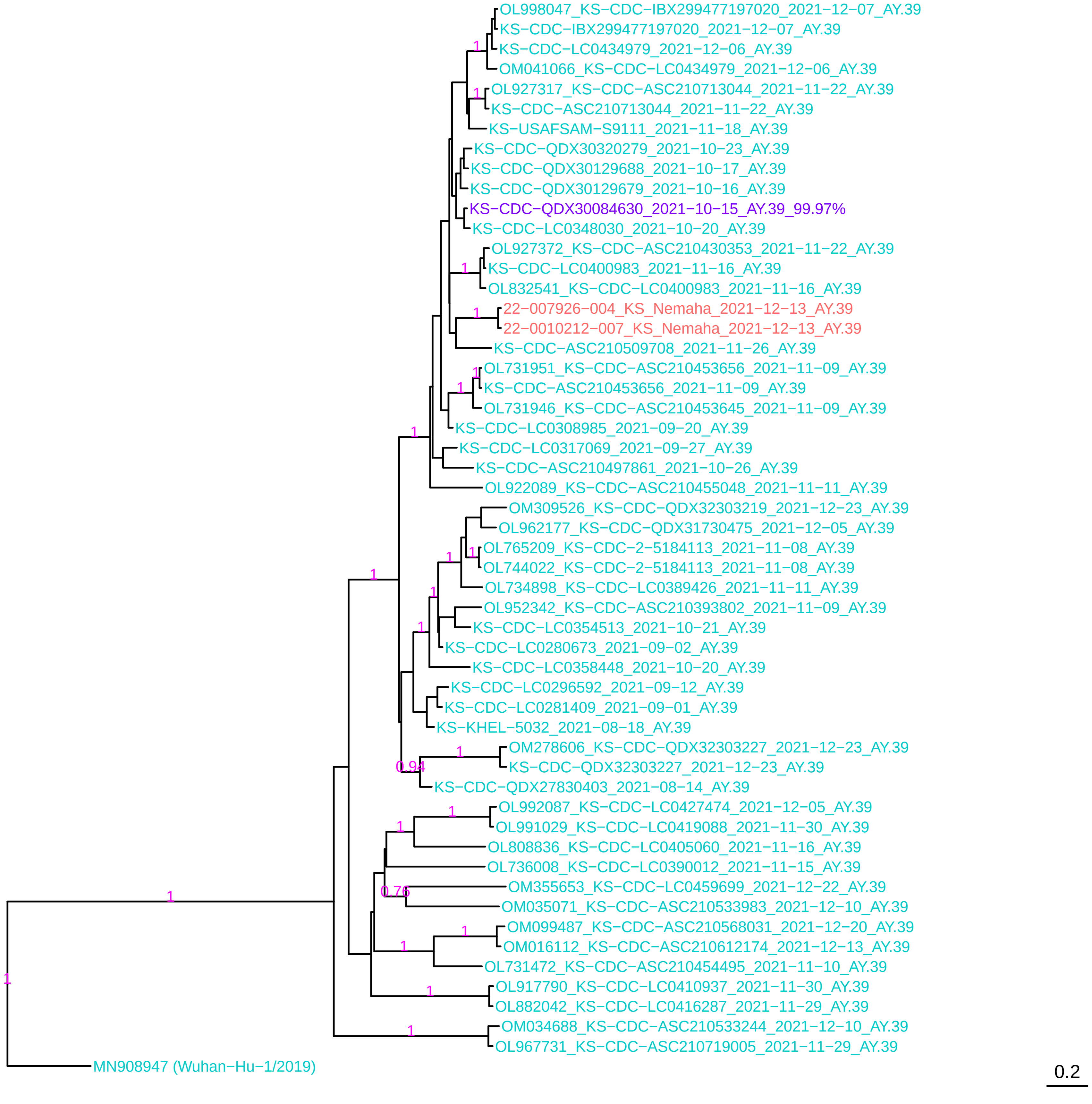
OL998047_KS−CDC−IBX299477197020_2021−12−07_AY.39
KS−CDC−IBX299477197020_2021−12−07_AY.39
KS−CDC−LC0434979_2021−12−06_AY.39
OM041066_KS−CDC−LC0434979_2021−12−06_AY.39
OL927317_KS−CDC−ASC210713044_2021−11−22_AY.39
KS−CDC−ASC210713044_2021−11−22_AY.39
KS−USAFSAM−S9111_2021−11−18_AY.39
KS−CDC−QDX30320279_2021−10−23_AY.39
KS−CDC−QDX30129688_2021−10−17_AY.39
KS−CDC−QDX30129679_2021−10−16_AY.39
KS−CDC−QDX30084630_2021−10−15_AY.39_99.97%
KS−CDC−LC0348030_2021−10−20_AY.39
OL927372_KS−CDC−ASC210430353_2021−11−22_AY.39
KS−CDC−LC0400983_2021−11−16_AY.39
OL832541_KS−CDC−LC0400983_2021−11−16_AY.39
22−007926−004_KS_Nemaha_2021−12−13_AY.39
22−0010212−007_KS_Nemaha_2021−12−13_AY.39
KS−CDC−ASC210509708_2021−11−26_AY.39
OL731951_KS−CDC−ASC210453656_2021−11−09_AY.39
KS−CDC−ASC210453656_2021−11−09_AY.39
OL731946_KS−CDC−ASC210453645_2021−11−09_AY.39
KS−CDC−LC0308985_2021−09−20_AY.39
KS−CDC−LC0317069_2021−09−27_AY.39
KS−CDC−ASC210497861_2021−10−26_AY.39
OL922089_KS−CDC−ASC210455048_2021−11−11_AY.39
OM309526_KS−CDC−QDX32303219_2021−12−23_AY.39
OL962177_KS−CDC−QDX31730475_2021−12−05_AY.39
OL765209_KS−CDC−2−5184113_2021−11−08_AY.39
OL744022_KS−CDC−2−5184113_2021−11−08_AY.39
OL734898_KS−CDC−LC0389426_2021−11−11_AY.39
OL952342_KS−CDC−ASC210393802_2021−11−09_AY.39
KS−CDC−LC0354513_2021−10−21_AY.39
KS−CDC−LC0280673_2021−09−02_AY.39
KS−CDC−LC0358448_2021−10−20_AY.39
KS−CDC−LC0296592_2021−09−12_AY.39
KS−CDC−LC0281409_2021−09−01_AY.39
KS−KHEL−5032_2021−08−18_AY.39
OM278606_KS−CDC−QDX32303227_2021−12−23_AY.39
KS−CDC−QDX32303227_2021−12−23_AY.39
KS−CDC−QDX27830403_2021−08−14_AY.39
OL992087_KS−CDC−LC0427474_2021−12−05_AY.39
OL991029_KS−CDC−LC0419088_2021−11−30_AY.39
OL808836_KS−CDC−LC0405060_2021−11−16_AY.39
OL736008_KS−CDC−LC0390012_2021−11−15_AY.39
OM355653_KS−CDC−LC0459699_2021−12−22_AY.39
OM035071_KS−CDC−ASC210533983_2021−12−10_AY.39
OM099487_KS−CDC−ASC210568031_2021−12−20_AY.39
OM016112_KS−CDC−ASC210612174_2021−12−13_AY.39
OL731472_KS−CDC−ASC210454495_2021−11−10_AY.39
OL917790_KS−CDC−LC0410937_2021−11−30_AY.39
OL882042_KS−CDC−LC0416287_2021−11−29_AY.39
OM034688_KS−CDC−ASC210533244_2021−12−10_AY.39
OL967731_KS−CDC−ASC210719005_2021−11−29_AY.39
MN908947 (Wuhan−Hu−1/2019)
0.94
0.76
0.2

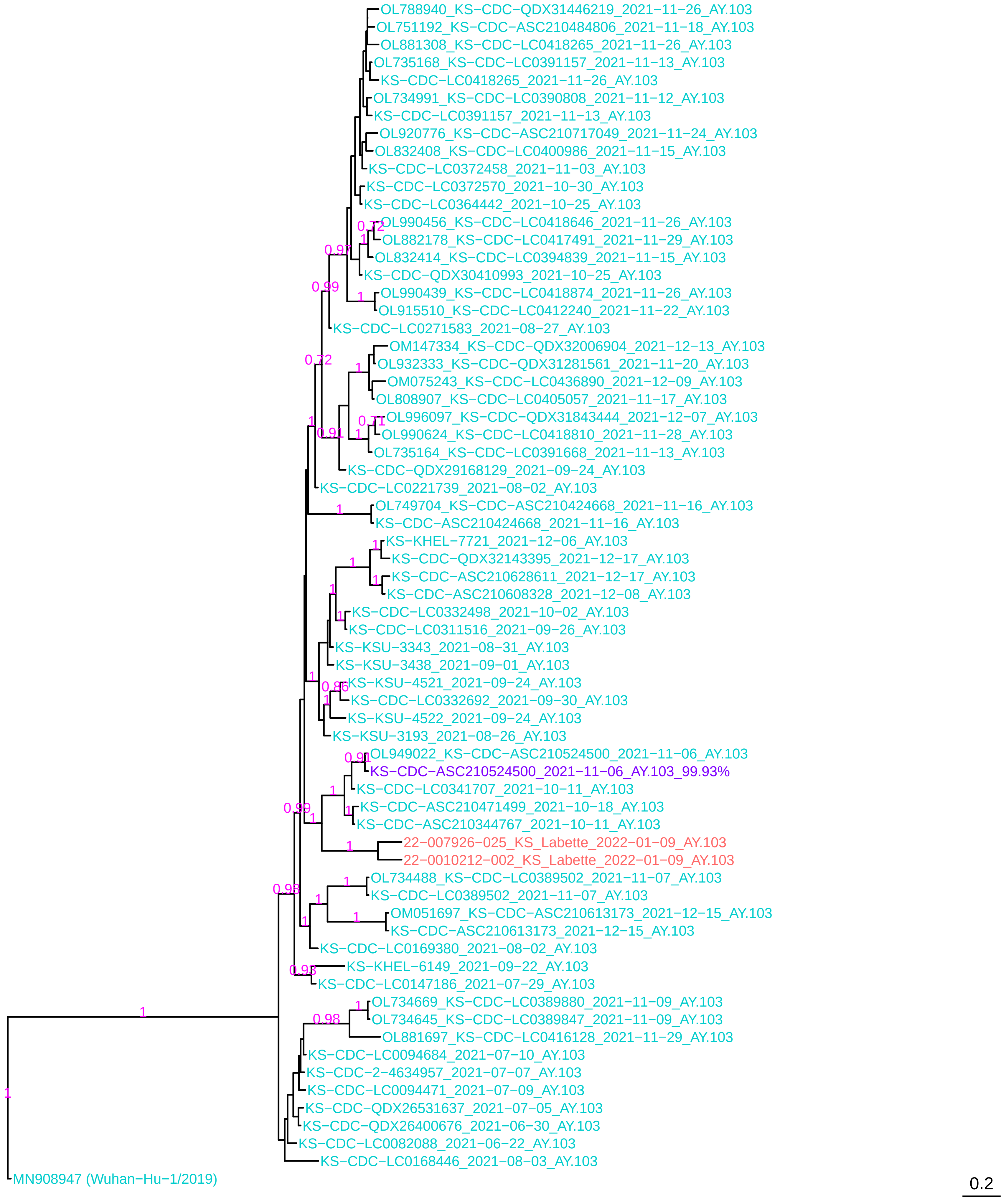
OL788940_KS-CDC-QDX31446219_2021-11-26_AY.103
OL751192_KS-CDC-ASC210484806_2021-11-18_AY.103
OL881308_KS-CDC-LC0418265_2021-11-26_AY.103
OL735168_KS-CDC-LC0391157_2021-11-13_AY.103
KS-CDC-LC0418265_2021-11-26_AY.103
OL734991_KS-CDC-LC0390808_2021-11-12_AY.103
KS-CDC-LC0391157_2021-11-13_AY.103
OL920776_KS-CDC-ASC210717049_2021-11-24_AY.103
OL832408_KS-CDC-LC0400986_2021-11-15_AY.103
KS-CDC-LC0372458_2021-11-03_AY.103
KS-CDC-LC0372570_2021-10-30_AY.103
KS-CDC-LC0364442_2021-10-25_AY.103
OL990456_KS-CDC-LC0418646_2021-11-26_AY.103
OL882178_KS-CDC-LC0417491_2021-11-29_AY.103
OL832414_KS-CDC-LC0394839_2021-11-15_AY.103
KS-CDC-QDX30410993_2021-10-25_AY.103
OL990439_KS-CDC-LC0418874_2021-11-26_AY.103
OL915510_KS-CDC-LC0412240_2021-11-22_AY.103
KS-CDC-LC0271583_2021-08-27_AY.103
OM147334_KS-CDC-QDX32006904_2021-12-13_AY.103
OL932333_KS-CDC-QDX31281561_2021-11-20_AY.103
OM075243_KS-CDC-LC0436890_2021-12-09_AY.103
OL808907_KS-CDC-LC0405057_2021-11-17_AY.103
OL996097_KS-CDC-QDX31843444_2021-12-07_AY.103
OL990624_KS-CDC-LC0418810_2021-11-28_AY.103
OL735164_KS-CDC-LC0391668_2021-11-13_AY.103
KS-CDC-QDX29168129_2021-09-24_AY.103
KS-CDC-LC0221739_2021-08-02_AY.103
OL749704_KS-CDC-ASC210424668_2021-11-16_AY.103
KS-CDC-ASC210424668_2021-11-16_AY.103
KS-KHEL-7721_2021-12-06_AY.103
KS-CDC-QDX32143395_2021-12-17_AY.103
KS-CDC-ASC210628611_2021-12-17_AY.103
KS-CDC-ASC210608328_2021-12-08_AY.103
KS-CDC-LC0332498_2021-10-02_AY.103
KS-CDC-LC0311516_2021-09-26_AY.103
KS-KSU-3343_2021-08-31_AY.103
KS-KSU-3438_2021-09-01_AY.103
KS-KSU-4521_2021-09-24_AY.103
KS-CDC-LC0332692_2021-09-30_AY.103
KS-KSU-4522_2021-09-24_AY.103
KS-KSU-3193_2021-08-26_AY.103
OL949022_KS-CDC-ASC210524500_2021-11-06_AY.103
KS-CDC-ASC210524500_2021-11-06_AY.103_99.93%
KS-CDC-LC0341707_2021-10-11_AY.103
KS-CDC-ASC210471499_2021-10-18_AY.103
KS-CDC-ASC210344767_2021-10-11_AY.103
22-007926-025_KS_Labette_2022-01-09_AY.103
22-0010212-002_KS_Labette_2022-01-09_AY.103
OL734488_KS-CDC-LC0389502_2021-11-07_AY.103
KS-CDC-LC0389502_2021-11-07_AY.103
OM051697_KS-CDC-ASC210613173_2021-12-15_AY.103
KS-CDC-ASC210613173_2021-12-15_AY.103
KS-CDC-LC0169380_2021-08-02_AY.103
KS-KHEL-6149_2021-09-22_AY.103
KS-CDC-LC0147186_2021-07-29_AY.103
OL734669_KS-CDC-LC0389880_2021-11-09_AY.103
OL734645_KS-CDC-LC0389847_2021-11-09_AY.103
OL881697_KS-CDC-LC0416128_2021-11-29_AY.103
KS-CDC-LC0094684_2021-07-10_AY.103
KS-CDC-2-4634957_2021-07-07_AY.103
KS-CDC-LC0094471_2021-07-09_AY.103
KS-CDC-QDX26531637_2021-07-05_AY.103
KS-CDC-QDX26400676_2021-06-30_AY.103
KS-CDC-LC0082088_2021-06-22_AY.103
KS-CDC-LC0168446_2021-08-03_AY.103
MN908947 (Wuhan-Hu-1/2019)
0.2

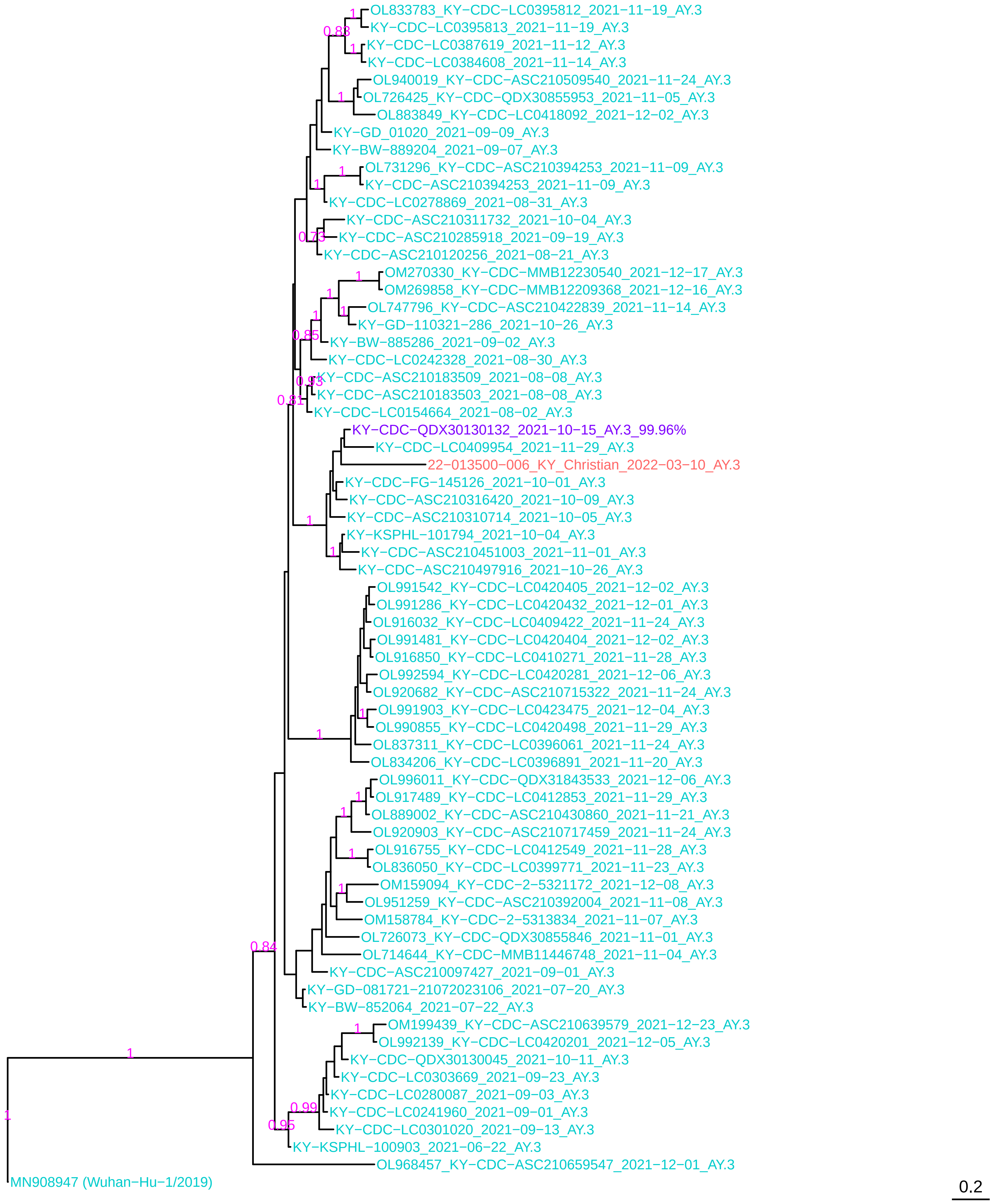

OL833783_KY−CDC−LC0395812_2021−11−19_AY.3
KY−CDC−LC0395813_2021−11−19_AY.3
KY−CDC−LC0387619_2021−11−12_AY.3
KY−CDC−LC0384608_2021−11−14_AY.3
OL940019_KY−CDC−ASC210509540_2021−11−24_AY.3
OL726425_KY−CDC−QDX30855953_2021−11−05_AY.3
OL883849_KY−CDC−LC0418092_2021−12−02_AY.3
KY−GD_01020_2021−09−09_AY.3
KY−BW−889204_2021−09−07_AY.3
OL731296_KY−CDC−ASC210394253_2021−11−09_AY.3
KY−CDC−ASC210394253_2021−11−09_AY.3
KY−CDC−LC0278869_2021−08−31_AY.3
KY−CDC−ASC210311732_2021−10−04_AY.3
KY−CDC−ASC210285918_2021−09−19_AY.3
KY−CDC−ASC210120256_2021−08−21_AY.3
OM270330_KY−CDC−MMB12230540_2021−12−17_AY.3
OM269858_KY−CDC−MMB12209368_2021−12−16_AY.3
OL747796_KY−CDC−ASC210422839_2021−11−14_AY.3
KY−GD−110321−286_2021−10−26_AY.3
KY−BW−885286_2021−09−02_AY.3
KY−CDC−LC0242328_2021−08−30_AY.3
KY−CDC−ASC210183509_2021−08−08_AY.3
KY−CDC−ASC210183503_2021−08−08_AY.3
KY−CDC−LC0154664_2021−08−02_AY.3
KY−CDC−QDX30130132_2021−10−15_AY.3_99.96%
KY−CDC−LC0409954_2021−11−29_AY.3
22−013500−006_KY_Christian_2022−03−10_AY.3
KY−CDC−FG−145126_2021−10−01_AY.3
KY−CDC−ASC210316420_2021−10−09_AY.3
KY−CDC−ASC210310714_2021−10−05_AY.3
KY−KSPHL−101794_2021−10−04_AY.3
KY−CDC−ASC210451003_2021−11−01_AY.3
KY−CDC−ASC210497916_2021−10−26_AY.3
OL991542_KY−CDC−LC0420405_2021−12−02_AY.3
OL991286_KY−CDC−LC0420432_2021−12−01_AY.3
OL916032_KY−CDC−LC0409422_2021−11−24_AY.3
OL991481_KY−CDC−LC0420404_2021−12−02_AY.3
OL916850_KY−CDC−LC0410271_2021−11−28_AY.3
OL992594_KY−CDC−LC0420281_2021−12−06_AY.3
OL920682_KY−CDC−ASC210715322_2021−11−24_AY.3
OL991903_KY−CDC−LC0423475_2021−12−04_AY.3
OL990855_KY−CDC−LC0420498_2021−11−29_AY.3
OL837311_KY−CDC−LC0396061_2021−11−24_AY.3
OL834206_KY−CDC−LC0396891_2021−11−20_AY.3
OL996011_KY−CDC−QDX31843533_2021−12−06_AY.3
OL917489_KY−CDC−LC0412853_2021−11−29_AY.3
OL889002_KY−CDC−ASC210430860_2021−11−21_AY.3
OL920903_KY−CDC−ASC210717459_2021−11−24_AY.3
OL916755_KY−CDC−LC0412549_2021−11−28_AY.3
OL836050_KY−CDC−LC0399771_2021−11−23_AY.3
OM159094_KY−CDC−2−5321172_2021−12−08_AY.3
OL951259_KY−CDC−ASC210392004_2021−11−08_AY.3
OM158784_KY−CDC−2−5313834_2021−11−07_AY.3
OL726073_KY−CDC−QDX30855846_2021−11−01_AY.3
OL714644_KY−CDC−MMB11446748_2021−11−04_AY.3
KY−CDC−ASC210097427_2021−09−01_AY.3
KY−GD−081721−21072023106_2021−07−20_AY.3
KY−BW−852064_2021−07−22_AY.3
OM199439_KY−CDC−ASC210639579_2021−12−23_AY.3
OL992139_KY−CDC−LC0420201_2021−12−05_AY.3
KY−CDC−QDX30130045_2021−10−11_AY.3
KY−CDC−LC0303669_2021−09−23_AY.3
KY−CDC−LC0280087_2021−09−03_AY.3
KY−CDC−LC0241960_2021−09−01_AY.3
KY−CDC−LC0301020_2021−09−13_AY.3
KY−KSPHL−100903_2021−06−22_AY.3
OL968457_KY−CDC−ASC210659547_2021−12−01_AY.3
MN908947 (Wuhan−Hu−1/2019)
0.2

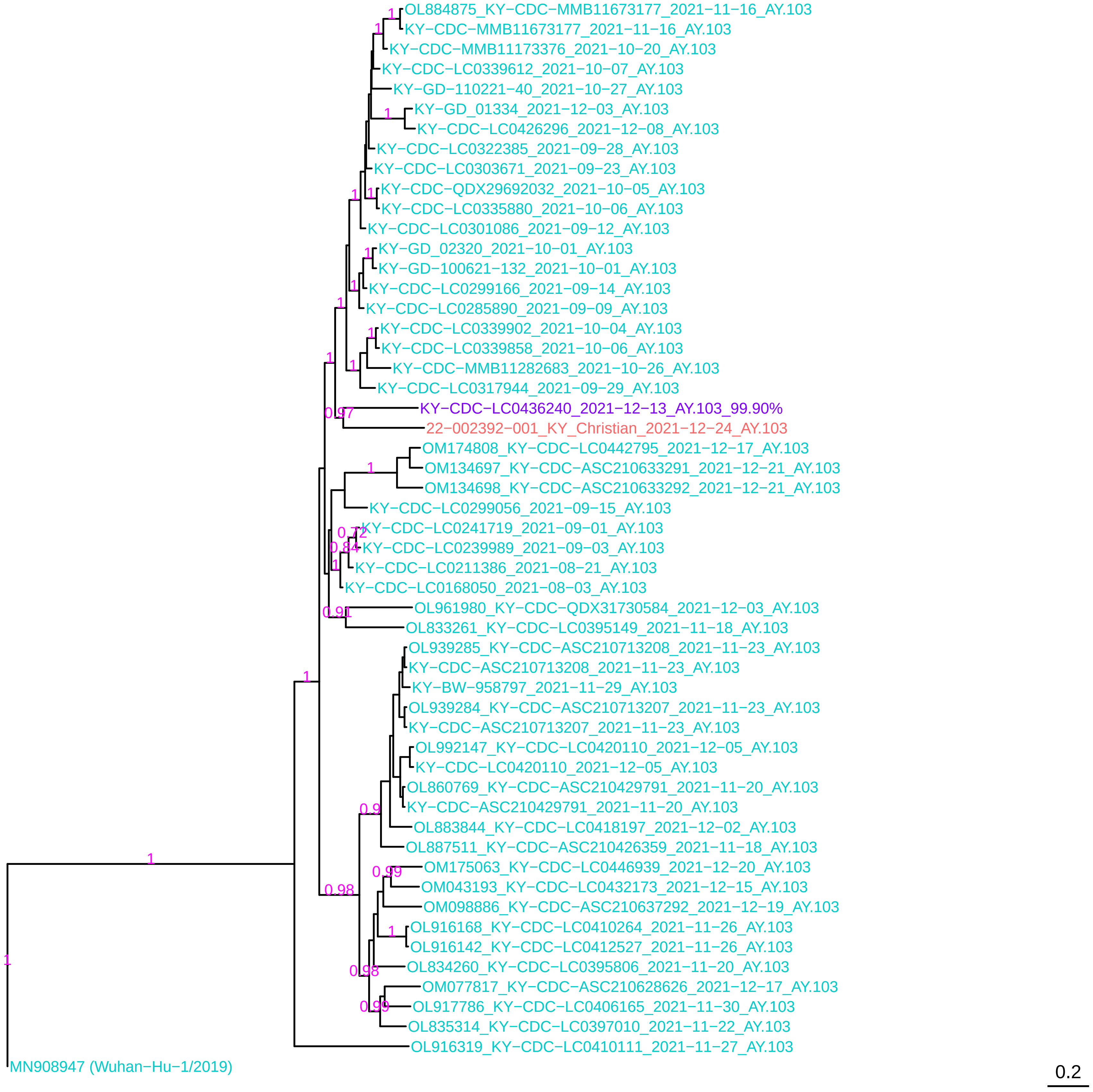
OL884875_KY−CDC−MMB11673177_2021−11−16_AY.103
KY−CDC−MMB11673177_2021−11−16_AY.103
KY−CDC−MMB11173376_2021−10−20_AY.103
KY−CDC−LC0339612_2021−10−07_AY.103
KY−GD−110221−40_2021−10−27_AY.103
KY−GD_01334_2021−12−03_AY.103
KY−CDC−LC0426296_2021−12−08_AY.103
KY−CDC−LC0322385_2021−09−28_AY.103
KY−CDC−LC0303671_2021−09−23_AY.103
KY−CDC−QDX29692032_2021−10−05_AY.103
KY−CDC−LC0335880_2021−10−06_AY.103
KY−CDC−LC0301086_2021−09−12_AY.103
KY−GD_02320_2021−10−01_AY.103
KY−GD−100621−132_2021−10−01_AY.103
KY−CDC−LC0299166_2021−09−14_AY.103
KY−CDC−LC0285890_2021−09−09_AY.103
KY−CDC−LC0339902_2021−10−04_AY.103
KY−CDC−LC0339858_2021−10−06_AY.103
KY−CDC−MMB11282683_2021−10−26_AY.103
KY−CDC−LC0317944_2021−09−29_AY.103
KY−CDC−LC0436240_2021−12−13_AY.103_99.90%
22−002392−001_KY_Christian_2021−12−24_AY.103
OM174808_KY−CDC−LC0442795_2021−12−17_AY.103
OM134697_KY−CDC−ASC210633291_2021−12−21_AY.103
OM134698_KY−CDC−ASC210633292_2021−12−21_AY.103
KY−CDC−LC0299056_2021−09−15_AY.103
KY−CDC−LC0241719_2021−09−01_AY.103
KY−CDC−LC0239989_2021−09−03_AY.103
KY−CDC−LC0211386_2021−08−21_AY.103
KY−CDC−LC0168050_2021−08−03_AY.103
OL961980_KY−CDC−QDX31730584_2021−12−03_AY.103
OL833261_KY−CDC−LC0395149_2021−11−18_AY.103
OL939285_KY−CDC−ASC210713208_2021−11−23_AY.103
KY−CDC−ASC210713208_2021−11−23_AY.103
KY−BW−958797_2021−11−29_AY.103
OL939284_KY−CDC−ASC210713207_2021−11−23_AY.103
KY−CDC−ASC210713207_2021−11−23_AY.103
OL992147_KY−CDC−LC0420110_2021−12−05_AY.103
KY−CDC−LC0420110_2021−12−05_AY.103
OL860769_KY−CDC−ASC210429791_2021−11−20_AY.103
KY−CDC−ASC210429791_2021−11−20_AY.103
OL883844_KY−CDC−LC0418197_2021−12−02_AY.103
OL887511_KY−CDC−ASC210426359_2021−11−18_AY.103
OM175063_KY−CDC−LC0446939_2021−12−20_AY.103
OM043193_KY−CDC−LC0432173_2021−12−15_AY.103
OM098886_KY−CDC−ASC210637292_2021−12−19_AY.103
OL916168_KY−CDC−LC0410264_2021−11−26_AY.103
OL916142_KY−CDC−LC0412527_2021−11−26_AY.103
OL834260_KY−CDC−LC0395806_2021−11−20_AY.103
OM077817_KY−CDC−ASC210628626_2021−12−17_AY.103
OL917786_KY−CDC−LC0406165_2021−11−30_AY.103
OL835314_KY−CDC−LC0397010_2021−11−22_AY.103
OL916319_KY−CDC−LC0410111_2021−11−27_AY.103
MN908947 (Wuhan−Hu−1/2019)
0.2

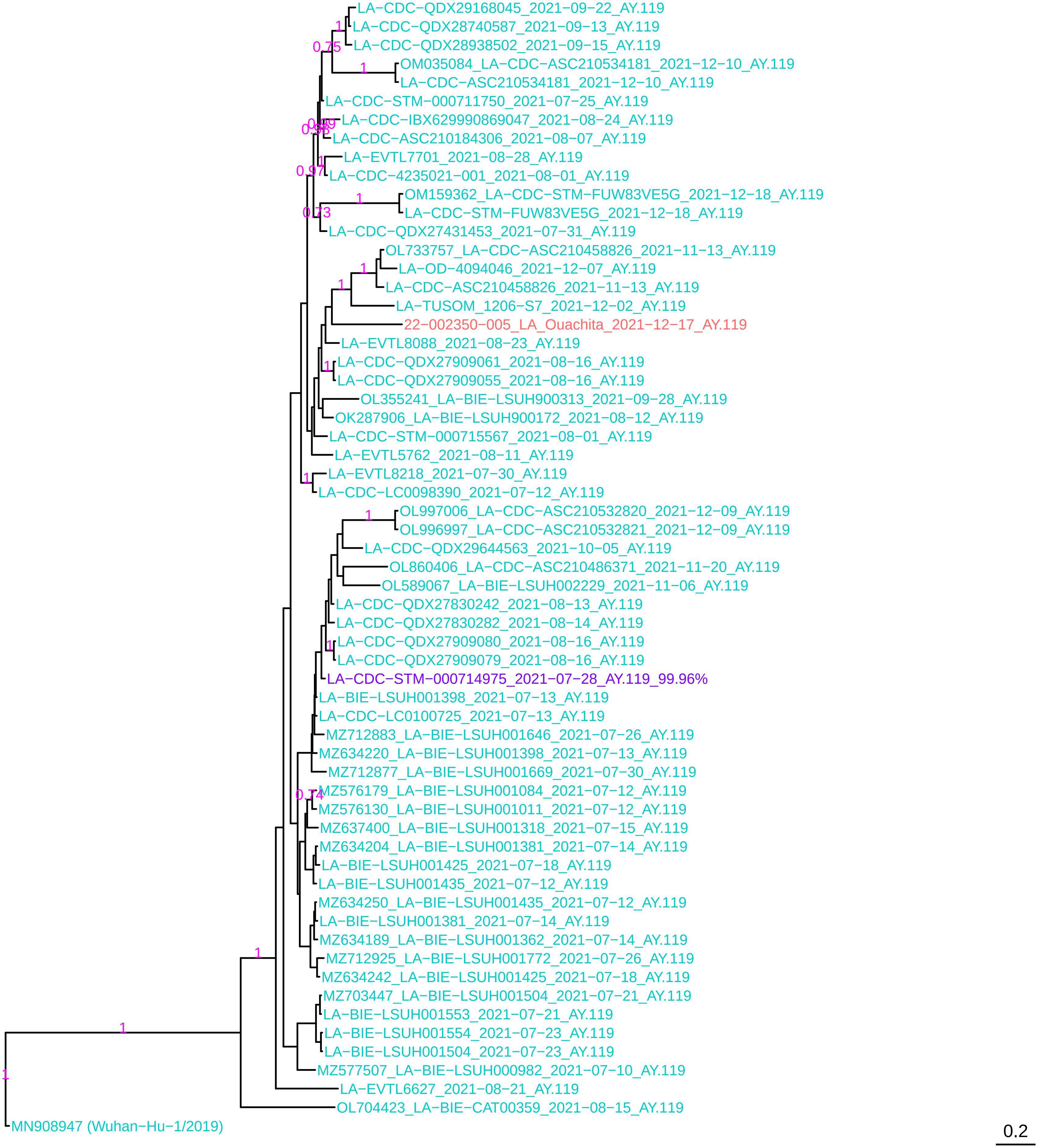
LA-CDC-QDX29168045_2021-09-22_AY.119
LA-CDC-QDX28740587_2021-09-13_AY.119
LA-CDC-QDX28938502_2021-09-15_AY.119
OM035084_LA-CDC-ASC210534181_2021-12-10_AY.119
LA-CDC-ASC210534181_2021-12-10_AY.119
LA-CDC-STM-000711750_2021-07-25_AY.119
LA-CDC-IBX629990869047_2021-08-24_AY.119
LA-CDC-ASC210184306_2021-08-07_AY.119
LA-EVTL7701_2021-08-28_AY.119
LA-CDC-4235021-001_2021-08-01_AY.119
OM159362_LA-CDC-STM-FUW83VE5G_2021-12-18_AY.119
LA-CDC-STM-FUW83VE5G_2021-12-18_AY.119
LA-CDC-QDX27431453_2021-07-31_AY.119
OL733757_LA-CDC-ASC210458826_2021-11-13_AY.119
LA-OD-4094046_2021-12-07_AY.119
LA-CDC-ASC210458826_2021-11-13_AY.119
LA-TUSOM_1206-S7_2021-12-02_AY.119
22-002350-005_LA_Ouachita_2021-12-17_AY.119
LA-EVTL8088_2021-08-23_AY.119
LA-CDC-QDX27909061_2021-08-16_AY.119
LA-CDC-QDX27909055_2021-08-16_AY.119
OL355241_LA-BIE-LSUH900313_2021-09-28_AY.119
OK287906_LA-BIE-LSUH900172_2021-08-12_AY.119
LA-CDC-STM-000715567_2021-08-01_AY.119
LA-EVTL5762_2021-08-11_AY.119
LA-EVTL8218_2021-07-30_AY.119
LA-CDC-LC0098390_2021-07-12_AY.119
OL997006_LA-CDC-ASC210532820_2021-12-09_AY.119
OL996997_LA-CDC-ASC210532821_2021-12-09_AY.119
LA-CDC-QDX29644563_2021-10-05_AY.119
OL860406_LA-CDC-ASC210486371_2021-11-20_AY.119
OL589067_LA-BIE-LSUH002229_2021-11-06_AY.119
LA-CDC-QDX27830242_2021-08-13_AY.119
LA-CDC-QDX27830282_2021-08-14_AY.119
LA-CDC-QDX27909080_2021-08-16_AY.119
LA-CDC-QDX27909079_2021-08-16_AY.119
LA-CDC-STM-000714975_2021-07-28_AY.119_99.96%
LA-BIE-LSUH001398_2021-07-13_AY.119
LA-CDC-LC0100725_2021-07-13_AY.119
MZ712883_LA-BIE-LSUH001646_2021-07-26_AY.119
MZ634220_LA-BIE-LSUH001398_2021-07-13_AY.119
MZ712877_LA-BIE-LSUH001669_2021-07-30_AY.119
MZ576179_LA-BIE-LSUH001084_2021-07-12_AY.119
MZ576130_LA-BIE-LSUH001011_2021-07-12_AY.119
MZ637400_LA-BIE-LSUH001318_2021-07-15_AY.119
MZ634204_LA-BIE-LSUH001381_2021-07-14_AY.119
LA-BIE-LSUH001425_2021-07-18_AY.119
LA-BIE-LSUH001435_2021-07-12_AY.119
MZ634250_LA-BIE-LSUH001435_2021-07-12_AY.119
LA-BIE-LSUH001381_2021-07-14_AY.119
MZ634189_LA-BIE-LSUH001362_2021-07-14_AY.119
MZ712925_LA-BIE-LSUH001772_2021-07-26_AY.119
MZ634242_LA-BIE-LSUH001425_2021-07-18_AY.119
MZ703447_LA-BIE-LSUH001504_2021-07-21_AY.119
LA-BIE-LSUH001553_2021-07-21_AY.119
LA-BIE-LSUH001554_2021-07-23_AY.119
LA-BIE-LSUH001504_2021-07-23_AY.119
MZ577507_LA-BIE-LSUH000982_2021-07-10_AY.119
LA-EVTL6627_2021-08-21_AY.119
OL704423_LA-BIE-CAT00359_2021-08-15_AY.119
MN908947 (Wuhan-Hu-1/2019)
0.2

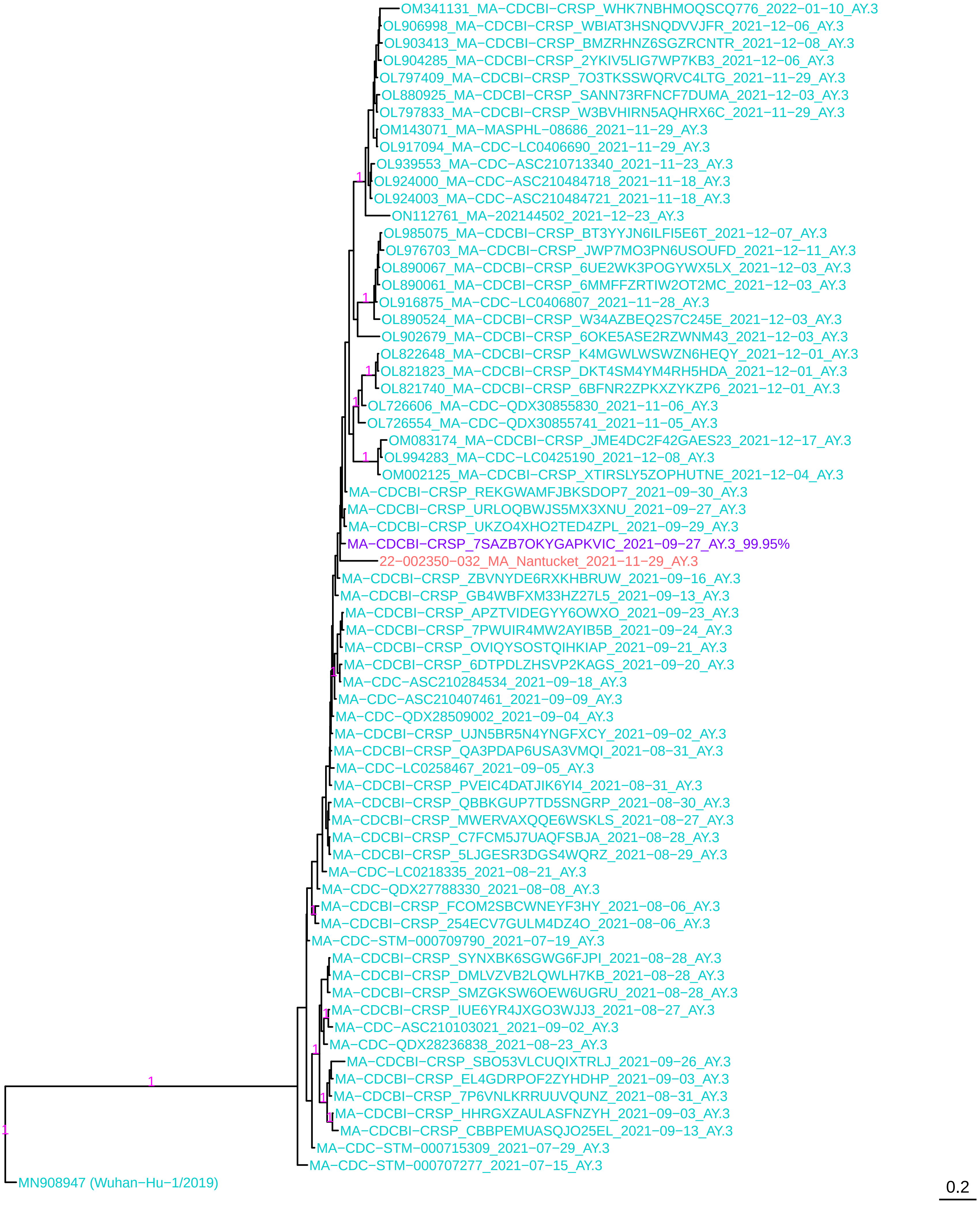
OM341131_MA−CDCBI−CRSP_WHK7NBHMOQSCQ776_2022−01−10_AY.3
OL906998_MA−CDCBI−CRSP_WBIAT3HSNQDVVJFR_2021−12−06_AY.3
OL903413_MA−CDCBI−CRSP_BMZRHNZ6SGZRCNTR_2021−12−08_AY.3
OL904285_MA−CDCBI−CRSP_2YKIV5LIG7WP7KB3_2021−12−06_AY.3
OL797409_MA−CDCBI−CRSP_7O3TKSSWQRVC4LTG_2021−11−29_AY.3
OL880925_MA−CDCBI−CRSP_SANN73RFNCF7DUMA_2021−12−03_AY.3
OL797833_MA−CDCBI−CRSP_W3BVHIRN5AQHRX6C_2021−11−29_AY.3
OM143071_MA−MASPHL−08686_2021−11−29_AY.3
OL917094_MA−CDC−LC0406690_2021−11−29_AY.3
OL939553_MA−CDC−ASC210713340_2021−11−23_AY.3
OL924000_MA−CDC−ASC210484718_2021−11−18_AY.3
OL924003_MA−CDC−ASC210484721_2021−11−18_AY.3
ON112761_MA−202144502_2021−12−23_AY.3
OL985075_MA−CDCBI−CRSP_BT3YYJN6ILFI5E6T_2021−12−07_AY.3
OL976703_MA−CDCBI−CRSP_JWP7MO3PN6USOUFD_2021−12−11_AY.3
OL890067_MA−CDCBI−CRSP_6UE2WK3POGYWX5LX_2021−12−03_AY.3
OL890061_MA−CDCBI−CRSP_6MMFFZRTIW2OT2MC_2021−12−03_AY.3
OL916875_MA−CDC−LC0406807_2021−11−28_AY.3
OL890524_MA−CDCBI−CRSP_W34AZBEQ2S7C245E_2021−12−03_AY.3
OL902679_MA−CDCBI−CRSP_6OKE5ASE2RZWNM43_2021−12−03_AY.3
OL822648_MA−CDCBI−CRSP_K4MGWLWSWZN6HEQY_2021−12−01_AY.3
OL821823_MA−CDCBI−CRSP_DKT4SM4YM4RH5HDA_2021−12−01_AY.3
OL821740_MA−CDCBI−CRSP_6BFNR2ZPKXZYKZP6_2021−12−01_AY.3
OL726606_MA−CDC−QDX30855830_2021−11−06_AY.3
OL726554_MA−CDC−QDX30855741_2021−11−05_AY.3
OM083174_MA−CDCBI−CRSP_JME4DC2F42GAES23_2021−12−17_AY.3
OL994283_MA−CDC−LC0425190_2021−12−08_AY.3
OM002125_MA−CDCBI−CRSP_XTIRSLY5ZOPHUTNE_2021−12−04_AY.3
MA−CDCBI−CRSP_REKGWAMFJBKSDOP7_2021−09−30_AY.3
MA−CDCBI−CRSP_URLOQBWJS5MX3XNU_2021−09−27_AY.3
MA−CDCBI−CRSP_UKZO4XHO2TED4ZPL_2021−09−29_AY.3
MA−CDCBI−CRSP_7SAZB7OKYGAPKVIC_2021−09−27_AY.3_99.95%
22−002350−032_MA_Nantucket_2021−11−29_AY.3
MA−CDCBI−CRSP_ZBVNYDE6RXKHBRUW_2021−09−16_AY.3
MA−CDCBI−CRSP_GB4WBFXM33HZ27L5_2021−09−13_AY.3
MA−CDCBI−CRSP_APZTVIDEGYY6OWXO_2021−09−23_AY.3
MA−CDCBI−CRSP_7PWUIR4MW2AYIB5B_2021−09−24_AY.3
MA−CDCBI−CRSP_OVIQYSOSTQIHKIAP_2021−09−21_AY.3
MA−CDCBI−CRSP_6DTPDLZHSVP2KAGS_2021−09−20_AY.3
MA−CDC−ASC210284534_2021−09−18_AY.3
MA−CDC−ASC210407461_2021−09−09_AY.3
MA−CDC−QDX28509002_2021−09−04_AY.3
MA−CDCBI−CRSP_UJN5BR5N4YNGFXCY_2021−09−02_AY.3
MA−CDCBI−CRSP_QA3PDAP6USA3VMQI_2021−08−31_AY.3
MA−CDC−LC0258467_2021−09−05_AY.3
MA−CDCBI−CRSP_PVEIC4DATJIK6YI4_2021−08−31_AY.3
MA−CDCBI−CRSP_QBBKGUP7TD5SNGRP_2021−08−30_AY.3
MA−CDCBI−CRSP_MWERVAXQQE6WSKLS_2021−08−27_AY.3
MA−CDCBI−CRSP_C7FCM5J7UAQFSBJA_2021−08−28_AY.3
MA−CDCBI−CRSP_5LJGESR3DGS4WQRZ_2021−08−29_AY.3
MA−CDC−LC0218335_2021−08−21_AY.3
MA−CDC−QDX27788330_2021−08−08_AY.3
MA−CDCBI−CRSP_FCOM2SBCWNEYF3HY_2021−08−06_AY.3
MA−CDCBI−CRSP_254ECV7GULM4DZ4O_2021−08−06_AY.3
MA−CDC−STM−000709790_2021−07−19_AY.3
MA−CDCBI−CRSP_SYNXBK6SGWG6FJPI_2021−08−28_AY.3
MA−CDCBI−CRSP_DMLVZVB2LQWLH7KB_2021−08−28_AY.3
MA−CDCBI−CRSP_SMZGKSW6OEW6UGRU_2021−08−28_AY.3
MA−CDCBI−CRSP_IUE6YR4JXGO3WJJ3_2021−08−27_AY.3
MA−CDC−ASC210103021_2021−09−02_AY.3
MA−CDC−QDX28236838_2021−08−23_AY.3
MA−CDCBI−CRSP_SBO53VLCUQIXTRLJ_2021−09−26_AY.3
MA−CDCBI−CRSP_EL4GDRPOF2ZYHDHP_2021−09−03_AY.3
MA−CDCBI−CRSP_7P6VNLKRRUUVQUNZ_2021−08−31_AY.3
MA−CDCBI−CRSP_HHRGXZAULASFNZYH_2021−09−03_AY.3
MA−CDCBI−CRSP_CBBPEMUASQJO25EL_2021−09−13_AY.3
MA−CDC−STM−000715309_2021−07−29_AY.3
MA−CDC−STM−000707277_2021−07−15_AY.3
MN908947 (Wuhan−Hu−1/2019)
0.2

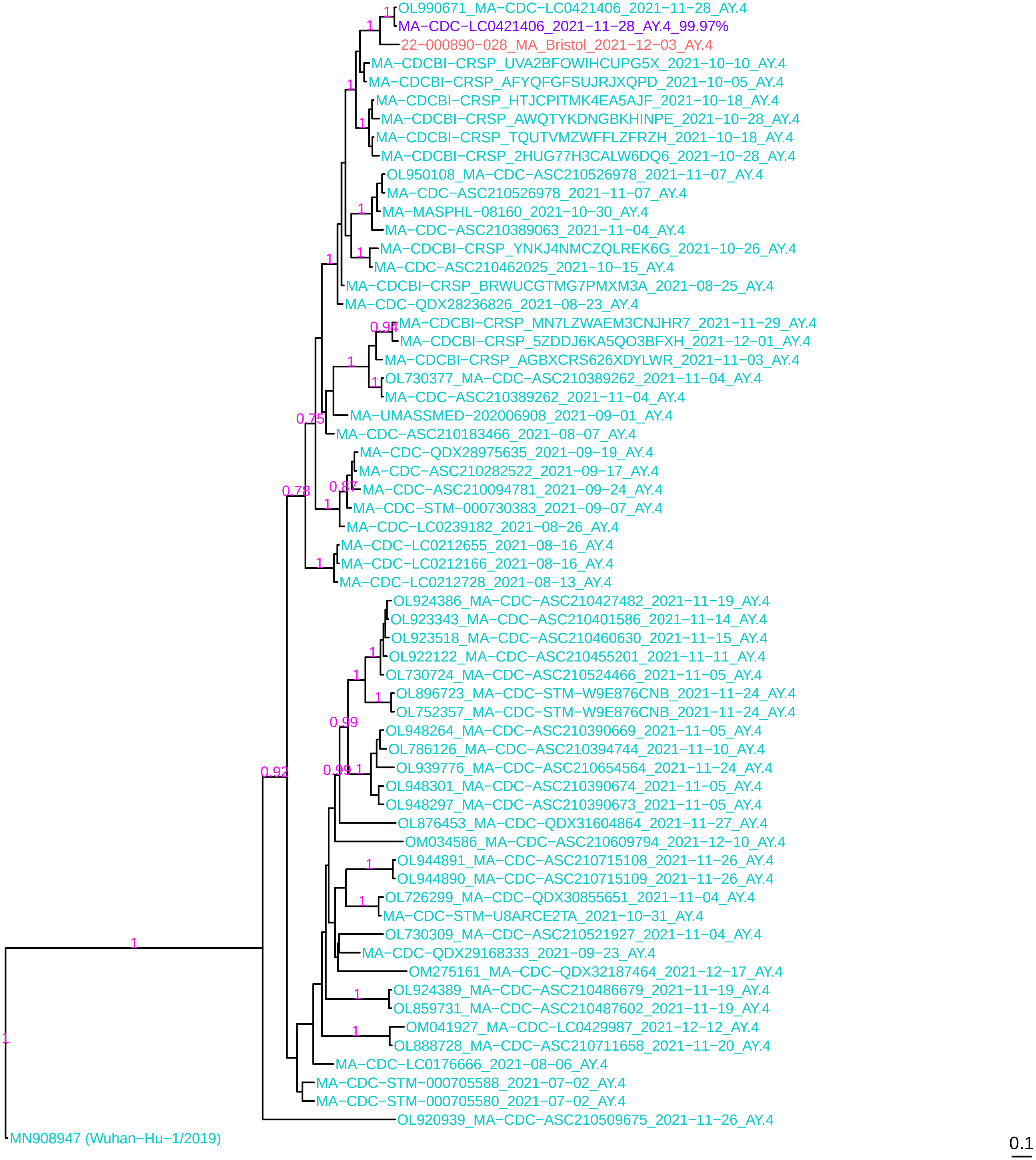
OL990671_MA−CDC−LC0421406_2021−11−28_AY.4
MA−CDC−LC0421406_2021−11−28_AY.4_99.97%
22−000890−028_MA_Bristol_2021−12−03_AY.4
MA−CDCBI−CRSP_UVA2BFOWIHCUPG5X_2021−10−10_AY.4
MA−CDCBI−CRSP_AFYQFGFSUJRJXQPD_2021−10−05_AY.4
MA−CDCBI−CRSP_HTJCPITMK4EA5AJF_2021−10−18_AY.4
MA−CDCBI−CRSP_AWQTYKDNGBKHINPE_2021−10−28_AY.4
MA−CDCBI−CRSP_TQUTVMZWFFLZFRZH_2021−10−18_AY.4
MA−CDCBI−CRSP_2HUG77H3CALW6DQ6_2021−10−28_AY.4
OL950108_MA−CDC−ASC210526978_2021−11−07_AY.4
MA−CDC−ASC210526978_2021−11−07_AY.4
MA−MASPHL−08160_2021−10−30_AY.4
MA−CDC−ASC210389063_2021−11−04_AY.4
MA−CDCBI−CRSP_YNKJ4NMCZQLREK6G_2021−10−26_AY.4
MA−CDC−ASC210462025_2021−10−15_AY.4
MA−CDCBI−CRSP_BRWUCGTMG7PMXM3A_2021−08−25_AY.4
MA−CDC−QDX28236826_2021−08−23_AY.4
MA−CDCBI−CRSP_MN7LZWAEM3CNJHR7_2021−11−29_AY.4
MA−CDCBI−CRSP_5ZDDJ6KA5QO3BFXH_2021−12−01_AY.4
MA−CDCBI−CRSP_AGBXCRS626XDYLWR_2021−11−03_AY.4
OL730377_MA−CDC−ASC210389262_2021−11−04_AY.4
MA−CDC−ASC210389262_2021−11−04_AY.4
MA−UMASSMED−202006908_2021−09−01_AY.4
MA−CDC−ASC210183466_2021−08−07_AY.4
MA−CDC−QDX28975635_2021−09−19_AY.4
MA−CDC−ASC210282522_2021−09−17_AY.4
MA−CDC−ASC210094781_2021−09−24_AY.4
MA−CDC−STM−000730383_2021−09−07_AY.4
MA−CDC−LC0239182_2021−08−26_AY.4
MA−CDC−LC0212655_2021−08−16_AY.4
MA−CDC−LC0212166_2021−08−16_AY.4
MA−CDC−LC0212728_2021−08−13_AY.4
OL924386_MA−CDC−ASC210427482_2021−11−19_AY.4
OL923343_MA−CDC−ASC210401586_2021−11−14_AY.4
OL923518_MA−CDC−ASC210460630_2021−11−15_AY.4
OL922122_MA−CDC−ASC210455201_2021−11−11_AY.4
OL730724_MA−CDC−ASC210524466_2021−11−05_AY.4
OL896723_MA−CDC−STM−W9E876CNB_2021−11−24_AY.4
OL752357_MA−CDC−STM−W9E876CNB_2021−11−24_AY.4
OL948264_MA−CDC−ASC210390669_2021−11−05_AY.4
OL786126_MA−CDC−ASC210394744_2021−11−10_AY.4
OL939776_MA−CDC−ASC210654564_2021−11−24_AY.4
OL948301_MA−CDC−ASC210390674_2021−11−05_AY.4
OL948297_MA−CDC−ASC210390673_2021−11−05_AY.4
OL876453_MA−CDC−QDX31604864_2021−11−27_AY.4
OM034586_MA−CDC−ASC210609794_2021−12−10_AY.4
OL944891_MA−CDC−ASC210715108_2021−11−26_AY.4
OL944890_MA−CDC−ASC210715109_2021−11−26_AY.4
OL726299_MA−CDC−QDX30855651_2021−11−04_AY.4
MA−CDC−STM−U8ARCE2TA_2021−10−31_AY.4
OL730309_MA−CDC−ASC210521927_2021−11−04_AY.4
MA−CDC−QDX29168333_2021−09−23_AY.4
OM275161_MA−CDC−QDX32187464_2021−12−17_AY.4
OL924389_MA−CDC−ASC210486679_2021−11−19_AY.4
OL859731_MA−CDC−ASC210487602_2021−11−19_AY.4
OM041927_MA−CDC−LC0429987_2021−12−12_AY.4
OL888728_MA−CDC−ASC210711658_2021−11−20_AY.4
MA−CDC−LC0176666_2021−08−06_AY.4
MA−CDC−STM−000705588_2021−07−02_AY.4
MA−CDC−STM−000705580_2021−07−02_AY.4
OL920939_MA−CDC−ASC210509675_2021−11−26_AY.4
MN908947 (Wuhan−Hu−1/2019)
0.1

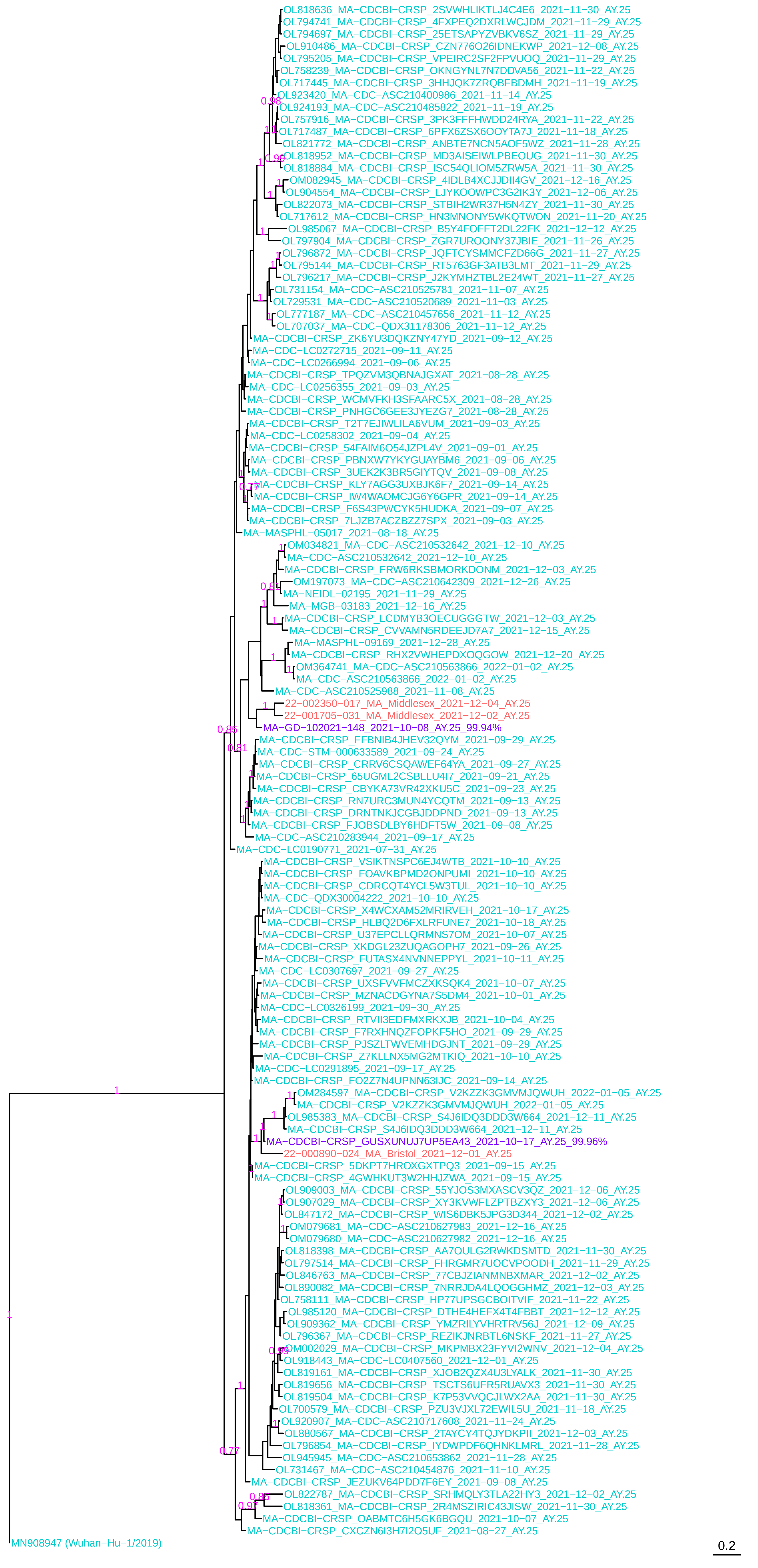

OL818636_MA-CDCBI-CRSP_2SVWHLIKTLJ4C4E6_2021-11-30_AY.25
OL794741_MA-CDCBI-CRSP_4FXPEQ2DXRLWCJDM_2021-11-29_AY.25
OL794697_MA-CDCBI-CRSP_25ETSAPYZVBKV6SZ_2021-11-29_AY.25
OL910486_MA-CDCBI-CRSP_CZN776O26IDNEKWP_2021-12-08_AY.25
OL795205_MA-CDCBI-CRSP_VPEIRC2SF2FPVUOQ_2021-11-29_AY.25
OL758239_MA-CDCBI-CRSP_OKNGYNL7N7DDVA56_2021-11-22_AY.25
OL717445_MA-CDCBI-CRSP_3HHJQK7ZRQBFBDMH_2021-11-19_AY.25
OL923420_MA-CDC-ASC210400986_2021-11-14_AY.25
OL924193_MA-CDC-ASC210485822_2021-11-19_AY.25
OL757916_MA-CDCBI-CRSP_3PK3FFFHWDD24RYA_2021-11-22_AY.25
OL717487_MA-CDCBI-CRSP_6PFX6ZSX6OOYTA7J_2021-11-18_AY.25
OL821772_MA-CDCBI-CRSP_ANBTE7NCN5AOF5WZ_2021-11-28_AY.25
OL818952_MA-CDCBI-CRSP_MD3AISEIWLPBEOUG_2021-11-30_AY.25
OL818884_MA-CDCBI-CRSP_ISC54QLIOM5ZRW5A_2021-11-30_AY.25
OM082945_MA-CDCBI-CRSP_4IDLB4XCJJDII4GV_2021-12-16_AY.25
OL904554_MA-CDCBI-CRSP_LJYKOOWPC3G2IK3Y_2021-12-06_AY.25
OL822073_MA-CDCBI-CRSP_STBIH2WR37H5N4ZY_2021-11-30_AY.25
OL717612_MA-CDCBI-CRSP_HN3MNONY5WKQTWON_2021-11-20_AY.25
OL985067_MA-CDCBI-CRSP_B5Y4FOFFT2DL22FK_2021-12-12_AY.25
OL797904_MA-CDCBI-CRSP_ZGR7UROONY37JBIE_2021-11-26_AY.25
OL796872_MA-CDCBI-CRSP_JQFTCYSMMCFZD66G_2021-11-27_AY.25
OL795144_MA-CDCBI-CRSP_RT5763GF3ATB3LMT_2021-11-29_AY.25
OL796217_MA-CDCBI-CRSP_J2KYMHZTBL2E24WT_2021-11-27_AY.25
OL731154_MA-CDC-ASC210525781_2021-11-07_AY.25
OL729531_MA-CDC-ASC210520689_2021-11-03_AY.25
OL777187_MA-CDC-ASC210457656_2021-11-12_AY.25
OL707037_MA-CDC-QDX31178306_2021-11-12_AY.25
MA-CDCBI-CRSP_ZK6YU3DQKZNY47YD_2021-09-12_AY.25
MA-CDC-LC0272715_2021-09-11_AY.25
MA-CDC-LC0266994_2021-09-06_AY.25
MA-CDCBI-CRSP_TPQZVM3QBNAJGXAT_2021-08-28_AY.25
MA-CDC-LC0256355_2021-09-03_AY.25
MA-CDCBI-CRSP_WCMVFKH3SFAARC5X_2021-08-28_AY.25
MA-CDCBI-CRSP_PNHGC6GEE3JYEZG7_2021-08-28_AY.25
MA-CDCBI-CRSP_T2T7EJIWLILA6VUM_2021-09-03_AY.25
MA-CDC-LC0258302_2021-09-04_AY.25
MA-CDCBI-CRSP_54FAIM6O54JZPL4V_2021-09-01_AY.25
MA-CDCBI-CRSP_PBNXW7YKYGUAYBM6_2021-09-06_AY.25
MA-CDCBI-CRSP_3UEK2K3BR5GIYTQV_2021-09-08_AY.25
MA-CDCBI-CRSP_KLY7AGG3UXBJK6F7_2021-09-14_AY.25
MA-CDCBI-CRSP_IW4WAOMCJG6Y6GPR_2021-09-14_AY.25
MA-CDCBI-CRSP_F6S43PWCYK5HUDKA_2021-09-07_AY.25
MA-CDCBI-CRSP_7LJZB7ACZBZZ7SPX_2021-09-03_AY.25
MA-MASPHL-05017_2021-08-18_AY.25
OM034821_MA-CDC-ASC210532642_2021-12-10_AY.25
MA-CDC-ASC210532642_2021-12-10_AY.25
MA-CDCBI-CRSP_FRW6RKSBMORKDONM_2021-12-03_AY.25
OM197073_MA-CDC-ASC210642309_2021-12-26_AY.25
MA-NEIDL-02195_2021-11-29_AY.25
MA-MGB-03183_2021-12-16_AY.25
MA-CDCBI-CRSP_LCDMYB3OECUGGGTW_2021-12-03_AY.25
MA-CDCBI-CRSP_CVVAMN5RDEEJD7A7_2021-12-15_AY.25
MA-MASPHL-09169_2021-12-28_AY.25
MA-CDCBI-CRSP_RHX2VWHEPDXOQGOW_2021-12-20_AY.25
OM364741_MA-CDC-ASC210563866_2022-01-02_AY.25
MA-CDC-ASC210563866_2022-01-02_AY.25
MA-CDC-ASC210525988_2021-11-08_AY.25
22-002350-017_MA_Middlesex_2021-12-04_AY.25
22-001705-031_MA_Middlesex_2021-12-02_AY.25
MA-GD-102021-148_2021-10-08_AY.25_99.94%
MA-CDCBI-CRSP_FFBNIB4JHEV32QYM_2021-09-29_AY.25
MA-CDC-STM-000633589_2021-09-24_AY.25
MA-CDCBI-CRSP_CRRV6CSQAWEF64YA_2021-09-27_AY.25
MA-CDCBI-CRSP_65UGML2CSBLLU4I7_2021-09-21_AY.25
MA-CDCBI-CRSP_CBYKA73VR42XKU5C_2021-09-23_AY.25
MA-CDCBI-CRSP_RN7URC3MUN4YCQTM_2021-09-13_AY.25
MA-CDCBI-CRSP_DRNTNKJCGBJDDPND_2021-09-13_AY.25
MA-CDCBI-CRSP_FJOBSDLBY6HDFT5W_2021-09-08_AY.25
MA-CDC-ASC210283944_2021-09-17_AY.25
MA-CDC-LC0190771_2021-07-31_AY.25
MA-CDCBI-CRSP_VSIKTNSPC6EJ4WTB_2021-10-10_AY.25
MA-CDCBI-CRSP_FOAVKBPMD2ONPUMI_2021-10-10_AY.25
MA-CDCBI-CRSP_CDRCQT4YCL5W3TUL_2021-10-10_AY.25
MA-CDC-QDX30004222_2021-10-10_AY.25
MA-CDCBI-CRSP_X4WCXAM52MRIRVEH_2021-10-17_AY.25
MA-CDCBI-CRSP_HLBQ2D6FXLRFUNE7_2021-10-18_AY.25
MA-CDCBI-CRSP_U37EPCLLQRMNS7OM_2021-10-07_AY.25
MA-CDCBI-CRSP_XKDGL23ZUQAGOPH7_2021-09-26_AY.25
MA-CDCBI-CRSP_FUTASX4NVNNEPPYL_2021-10-11_AY.25
MA-CDC-LC0307697_2021-09-27_AY.25
MA-CDCBI-CRSP_UXSFVVFMCZXKSQK4_2021-10-07_AY.25
MA-CDCBI-CRSP_MZNACDGYNA7S5DM4_2021-10-01_AY.25
MA-CDC-LC0326199_2021-09-30_AY.25
MA-CDCBI-CRSP_RTVII3EDFMXRKXJB_2021-10-04_AY.25
MA-CDCBI-CRSP_F7RXHNQZFOPKF5HO_2021-09-29_AY.25
MA-CDCBI-CRSP_PJSZLTWVEMHDGJNT_2021-09-29_AY.25
MA-CDCBI-CRSP_Z7KLLNX5MG2MTKIQ_2021-10-10_AY.25
MA-CDC-LC0291895_2021-09-17_AY.25
MA-CDCBI-CRSP_FO2Z7N4UPNN63IJC_2021-09-14_AY.25
OM284597_MA-CDCBI-CRSP_V2KZZK3GMVMJQWUH_2022-01-05_AY.25
MA-CDCBI-CRSP_V2KZZK3GMVMJQWUH_2022-01-05_AY.25
OL985383_MA-CDCBI-CRSP_S4J6IDQ3DDD3W664_2021-12-11_AY.25
MA-CDCBI-CRSP_S4J6IDQ3DDD3W664_2021-12-11_AY.25
MA-CDCBI-CRSP_GUSXUNUJ7UP5EA43_2021-10-17_AY.25_99.96%
22-000890-024_MA_Bristol_2021-12-01_AY.25
MA-CDCBI-CRSP_5DKPT7HROXGXTPQ3_2021-09-15_AY.25
MA-CDCBI-CRSP_4GWHKUT3W2HHJZWA_2021-09-15_AY.25
OL909003_MA-CDCBI-CRSP_55YJOS3MXASCV3QZ_2021-12-06_AY.25
OL907029_MA-CDCBI-CRSP_XY3KVWFLZPTBZXY3_2021-12-06_AY.25
OL847172_MA-CDCBI-CRSP_WIS6DBK5JPG3D344_2021-12-02_AY.25
OM079681_MA-CDC-ASC210627983_2021-12-16_AY.25
OM079680_MA-CDC-ASC210627982_2021-12-16_AY.25
OL818398_MA-CDCBI-CRSP_AA7OULG2RWKDSMTD_2021-11-30_AY.25
OL797514_MA-CDCBI-CRSP_FHRGMR7UOCVPOODH_2021-11-29_AY.25
OL846763_MA-CDCBI-CRSP_77CBJZIANMNBXMAR_2021-12-02_AY.25
OL890082_MA-CDCBI-CRSP_7NRRJDA4LQOGGHMZ_2021-12-03_AY.25
OL758111_MA-CDCBI-CRSP_HP77UPSGCBOITVIF_2021-11-22_AY.25
OL985120_MA-CDCBI-CRSP_DTHE4HEFX4T4FBBT_2021-12-12_AY.25
OL909362_MA-CDCBI-CRSP_YMZRILYVHRTRV56J_2021-12-09_AY.25
OL796367_MA-CDCBI-CRSP_REZIKJNRBTL6NSKF_2021-11-27_AY.25
OM002029_MA-CDCBI-CRSP_MKPMBX23FYVI2WNV_2021-12-04_AY.25
OL918443_MA-CDC-LC0407560_2021-12-01_AY.25
OL819161_MA-CDCBI-CRSP_XJOB2QZX4U3LYALK_2021-11-30_AY.25
OL819656_MA-CDCBI-CRSP_TSCTS6UFR5RUAVX3_2021-11-30_AY.25
OL819504_MA-CDCBI-CRSP_K7P53VVQCJLWX2AA_2021-11-30_AY.25
OL700579_MA-CDCBI-CRSP_PZU3VJXL72EWIL5U_2021-11-18_AY.25
OL920907_MA-CDC-ASC210717608_2021-11-24_AY.25
OL880567_MA-CDCBI-CRSP_2TAYCY4TQJYDKPII_2021-12-03_AY.25
OL796854_MA-CDCBI-CRSP_IYDWPDF6QHNKLMRL_2021-11-28_AY.25
OL945945_MA-CDC-ASC210653862_2021-11-28_AY.25
OL731467_MA-CDC-ASC210454876_2021-11-10_AY.25
MA-CDCBI-CRSP_JEZUKV64PDD7F6EY_2021-09-08_AY.25
OL822787_MA-CDCBI-CRSP_SRHMQLY3TLA22HY3_2021-12-02_AY.25
OL818361_MA-CDCBI-CRSP_2R4MSZIRIC43JISW_2021-11-30_AY.25
MA-CDCBI-CRSP_OABMTC6H5GK6BGQU_2021-10-07_AY.25
MA-CDCBI-CRSP_CXCZN6I3H7I2O5UF_2021-08-27_AY.25
MN908947 (Wuhan-Hu-1/2019)
0.2

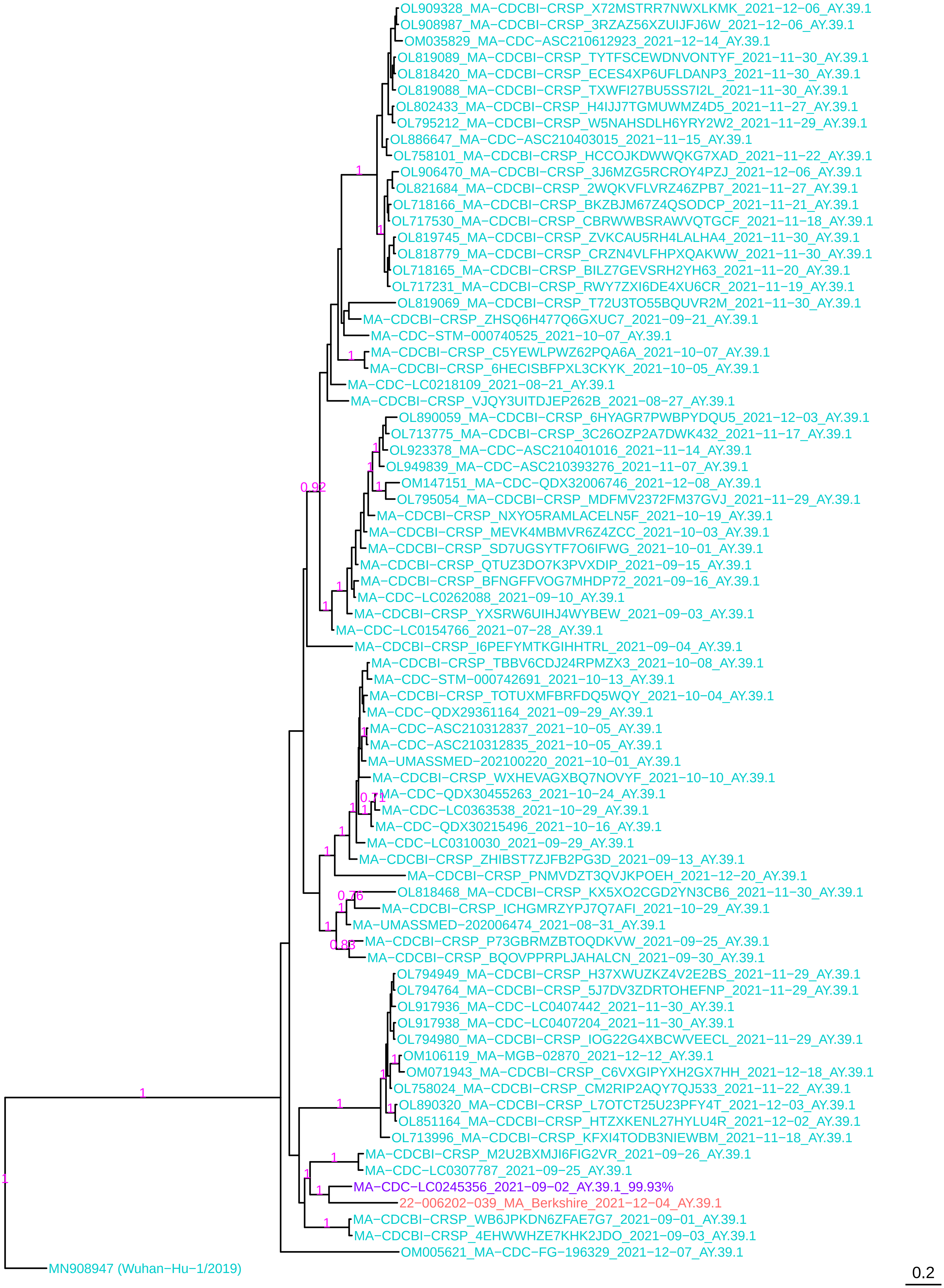

OL909328_MA−CDCBI−CRSP_X72MSTRR7NWXLKMK_2021−12−06_AY.39.1
OL908987_MA−CDCBI−CRSP_3RZAZ56XZUIJFJ6W_2021−12−06_AY.39.1
OM035829_MA−CDC−ASC210612923_2021−12−14_AY.39.1
OL819089_MA−CDCBI−CRSP_TYTFSCEWDNVONTYF_2021−11−30_AY.39.1
OL818420_MA−CDCBI−CRSP_ECES4XP6UFLDANP3_2021−11−30_AY.39.1
OL819088_MA−CDCBI−CRSP_TXWFI27BU5SS7I2L_2021−11−30_AY.39.1
OL802433_MA−CDCBI−CRSP_H4IJJ7TGMUWMZ4D5_2021−11−27_AY.39.1
OL795212_MA−CDCBI−CRSP_W5NAHSDLH6YRY2W2_2021−11−29_AY.39.1
OL886647_MA−CDC−ASC210403015_2021−11−15_AY.39.1
OL758101_MA−CDCBI−CRSP_HCCOJKDWWQKG7XAD_2021−11−22_AY.39.1
OL906470_MA−CDCBI−CRSP_3J6MZG5RCROY4PZJ_2021−12−06_AY.39.1
OL821684_MA−CDCBI−CRSP_2WQKVFLVRZ46ZPB7_2021−11−27_AY.39.1
OL718166_MA−CDCBI−CRSP_BKZBJM67Z4QSODCP_2021−11−21_AY.39.1
OL717530_MA−CDCBI−CRSP_CBRWWBSRAWVQTGCF_2021−11−18_AY.39.1
OL819745_MA−CDCBI−CRSP_ZVKCAU5RH4LALHA4_2021−11−30_AY.39.1
OL818779_MA−CDCBI−CRSP_CRZN4VLFHPXQAKWW_2021−11−30_AY.39.1
OL718165_MA−CDCBI−CRSP_BILZ7GEVSRH2YH63_2021−11−20_AY.39.1
OL717231_MA−CDCBI−CRSP_RWY7ZXI6DE4XU6CR_2021−11−19_AY.39.1
OL819069_MA−CDCBI−CRSP_T72U3TO55BQUVR2M_2021−11−30_AY.39.1
MA−CDCBI−CRSP_ZHSQ6H477Q6GXUC7_2021−09−21_AY.39.1
MA−CDC−STM−000740525_2021−10−07_AY.39.1
MA−CDCBI−CRSP_C5YEWLPWZ62PQA6A_2021−10−07_AY.39.1
MA−CDCBI−CRSP_6HECISBFPXL3CKYK_2021−10−05_AY.39.1
MA−CDC−LC0218109_2021−08−21_AY.39.1
MA−CDCBI−CRSP_VJQY3UITDJEP262B_2021−08−27_AY.39.1
OL890059_MA−CDCBI−CRSP_6HYAGR7PWBPYDQU5_2021−12−03_AY.39.1
OL713775_MA−CDCBI−CRSP_3C26OZP2A7DWK432_2021−11−17_AY.39.1
OL923378_MA−CDC−ASC210401016_2021−11−14_AY.39.1
OL949839_MA−CDC−ASC210393276_2021−11−07_AY.39.1
OM147151_MA−CDC−QDX32006746_2021−12−08_AY.39.1
OL795054_MA−CDCBI−CRSP_MDFMV2372FM37GVJ_2021−11−29_AY.39.1
MA−CDCBI−CRSP_NXYO5RAMLACELN5F_2021−10−19_AY.39.1
MA−CDCBI−CRSP_MEVK4MBMVR6Z4ZCC_2021−10−03_AY.39.1
MA−CDCBI−CRSP_SD7UGSYTF7O6IFWG_2021−10−01_AY.39.1
MA−CDCBI−CRSP_QTUZ3DO7K3PVXDIP_2021−09−15_AY.39.1
MA−CDCBI−CRSP_BFNGFFVOG7MHDP72_2021−09−16_AY.39.1
MA−CDC−LC0262088_2021−09−10_AY.39.1
MA−CDCBI−CRSP_YXSRW6UIHJ4WYBEW_2021−09−03_AY.39.1
MA−CDC−LC0154766_2021−07−28_AY.39.1
MA−CDCBI−CRSP_I6PEFYMTKGIHHTRL_2021−09−04_AY.39.1
MA−CDCBI−CRSP_TBBV6CDJ24RPMZX3_2021−10−08_AY.39.1
MA−CDC−STM−000742691_2021−10−13_AY.39.1
MA−CDCBI−CRSP_TOTUXMFBRFDQ5WQY_2021−10−04_AY.39.1
MA−CDC−QDX29361164_2021−09−29_AY.39.1
MA−CDC−ASC210312837_2021−10−05_AY.39.1
MA−CDC−ASC210312835_2021−10−05_AY.39.1
MA−UMASSMED−202100220_2021−10−01_AY.39.1
MA−CDCBI−CRSP_WXHEVAGXBQ7NOVYF_2021−10−10_AY.39.1
MA−CDC−QDX30455263_2021−10−24_AY.39.1
MA−CDC−LC0363538_2021−10−29_AY.39.1
MA−CDC−QDX30215496_2021−10−16_AY.39.1
MA−CDC−LC0310030_2021−09−29_AY.39.1
MA−CDCBI−CRSP_ZHIBST7ZJFB2PG3D_2021−09−13_AY.39.1
MA−CDCBI−CRSP_PNMVDZT3QVJKPOEH_2021−12−20_AY.39.1
OL818468_MA−CDCBI−CRSP_KX5XO2CGD2YN3CB6_2021−11−30_AY.39.1
MA−CDCBI−CRSP_ICHGMRZYPJ7Q7AFI_2021−10−29_AY.39.1
MA−UMASSMED−202006474_2021−08−31_AY.39.1
MA−CDCBI−CRSP_P73GBRMZBTOQDKVW_2021−09−25_AY.39.1
MA−CDCBI−CRSP_BQOVPPRPLJAHALCN_2021−09−30_AY.39.1
OL794949_MA−CDCBI−CRSP_H37XWUZKZ4V2E2BS_2021−11−29_AY.39.1
OL794764_MA−CDCBI−CRSP_5J7DV3ZDRTOHEFNP_2021−11−29_AY.39.1
OL917936_MA−CDC−LC0407442_2021−11−30_AY.39.1
OL917938_MA−CDC−LC0407204_2021−11−30_AY.39.1
OL794980_MA−CDCBI−CRSP_IOG22G4XBCWVEECL_2021−11−29_AY.39.1
OM106119_MA−MGB−02870_2021−12−12_AY.39.1
OM071943_MA−CDCBI−CRSP_C6VXGIPYXH2GX7HH_2021−12−18_AY.39.1
OL758024_MA−CDCBI−CRSP_CM2RIP2AQY7QJ533_2021−11−22_AY.39.1
OL890320_MA−CDCBI−CRSP_L7OTCT25U23PFY4T_2021−12−03_AY.39.1
OL851164_MA−CDCBI−CRSP_HTZXKENL27HYLU4R_2021−12−02_AY.39.1
OL713996_MA−CDCBI−CRSP_KFXI4TODB3NIEWBM_2021−11−18_AY.39.1
MA−CDCBI−CRSP_M2U2BXMJI6FIG2VR_2021−09−26_AY.39.1
MA−CDC−LC0307787_2021−09−25_AY.39.1
MA−CDC−LC0245356_2021−09−02_AY.39.1_99.93%
22−006202−039_MA_Berkshire_2021−12−04_AY.39.1
MA−CDCBI−CRSP_WB6JPKDN6ZFAE7G7_2021−09−01_AY.39.1
MA−CDCBI−CRSP_4EHWWHZE7KHK2JDO_2021−09−03_AY.39.1
OM005621_MA−CDC−FG−196329_2021−12−07_AY.39.1
MN908947 (Wuhan−Hu−1/2019)
0.2

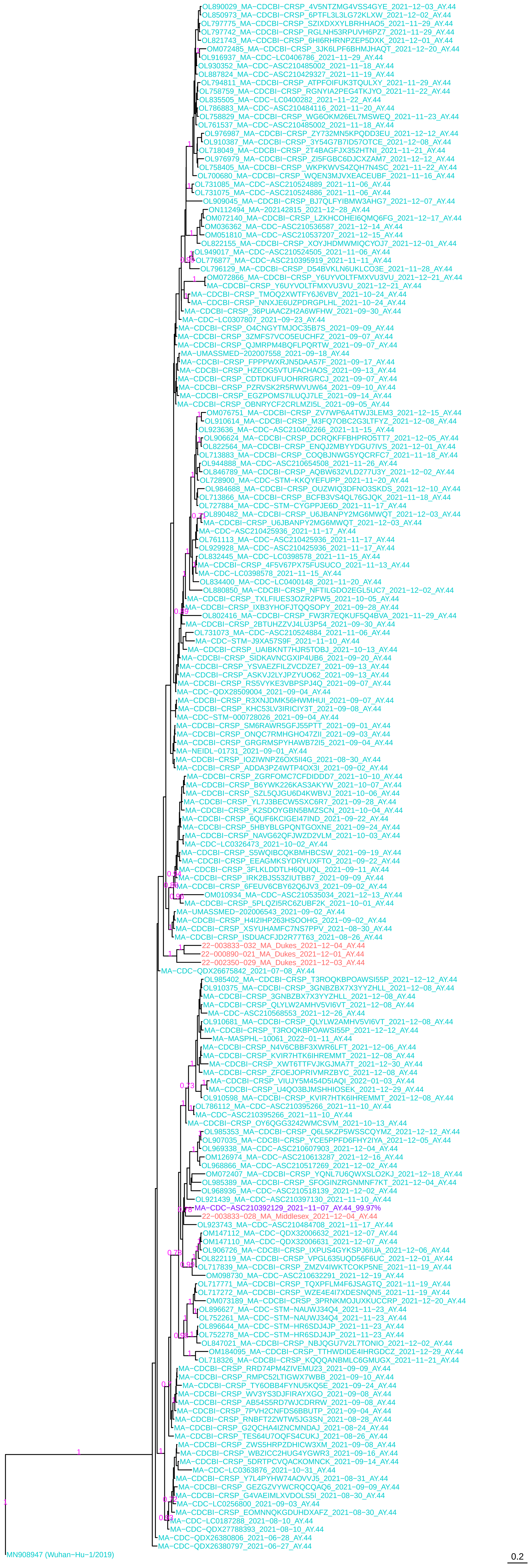
OL890029_MA-CDCBI-CRSP_4V5NTZMG4VSS4GYE_2021-12-03_AY.44
OL850973_MA-CDCBI-CRSP_6PTFL3L3LG72KLXW_2021-12-02_AY.44
OL797775_MA-CDCBI-CRSP_SZIXDXXYLBRHHAO5_2021-11-29_AY.44
OL797742_MA-CDCBI-CRSP_RGLNH53RPUVH6PZ7_2021-11-29_AY.44
OL821743_MA-CDCBI-CRSP_6HI6RHRNPZEP5DXK_2021-12-01_AY.44
OM072485_MA-CDCBI-CRSP_3JK6LPF6BHMJHAQT_2021-12-20_AY.44
OL916937_MA-CDC-LC0406786_2021-11-29_AY.44
OL930352_MA-CDC-ASC210485002_2021-11-18_AY.44
OL887824_MA-CDC-ASC210429327_2021-11-19_AY.44
OL794811_MA-CDCBI-CRSP_ATPFOIFUK3TQULXY_2021-11-29_AY.44
OL758759_MA-CDCBI-CRSP_RGNYIA2PEG4TKJYO_2021-11-22_AY.44
OL835505_MA-CDC-LC0400282_2021-11-22_AY.44
OL786883_MA-CDC-ASC210484116_2021-11-20_AY.44
OL758829_MA-CDCBI-CRSP_WG6OKM26EL7MSWEQ_2021-11-23_AY.44
OL761537_MA-CDC-ASC210485002_2021-11-18_AY.44
OL976987_MA-CDCBI-CRSP_ZY732MN5KPQDD3EU_2021-12-12_AY.44
OL910387_MA-CDCBI-CRSP_3Y54G7B7ID57OTCE_2021-12-08_AY.44
OL718049_MA-CDCBI-CRSP_2T4BAGFJX352HTNI_2021-11-21_AY.44
OL976979_MA-CDCBI-CRSP_ZI5FGBC6DJCXZAM7_2021-12-12_AY.44
OL758405_MA-CDCBI-CRSP_WKPKWVS4ZQH7N4SC_2021-11-22_AY.44
OL700680_MA-CDCBI-CRSP_WQEN3MJVXEACEUBF_2021-11-16_AY.44
OL731085_MA-CDC-ASC210524889_2021-11-06_AY.44
OL731075_MA-CDC-ASC210524886_2021-11-06_AY.44
OL909045_MA-CDCBI-CRSP_BJ7QLFYIBMW3AHG7_2021-12-07_AY.44
ON112494_MA-202142815_2021-12-28_AY.44
OM072140_MA-CDCBI-CRSP_LZKHCOHEI6QMQ6FG_2021-12-17_AY.44
OM036362_MA-CDC-ASC210536587_2021-12-14_AY.44
OM051810_MA-CDC-ASC210537207_2021-12-15_AY.44
OL822155_MA-CDCBI-CRSP_XOYJHDMWMIQCYOJ7_2021-12-01_AY.44
OL949017_MA-CDC-ASC210524505_2021-11-06_AY.44
OL776877_MA-CDC-ASC210395919_2021-11-11_AY.44
OL796129_MA-CDCBI-CRSP_D54BVKLN6UKLCO3E_2021-11-28_AY.44
OM072866_MA-CDCBI-CRSP_Y6UYVOLTFMXVU3VU_2021-12-21_AY.44
MA-CDCBI-CRSP_Y6UYVOLTFMXVU3VU_2021-12-21_AY.44
MA-CDCBI-CRSP_TMOQ2XWTFY6J6VBV_2021-10-24_AY.44
MA-CDCBI-CRSP_NNXJE6UZPDRGPLHL_2021-10-24_AY.44
MA-CDCBI-CRSP_36PUAACZH2A6WFHW_2021-09-30_AY.44
MA-CDC-LC0307807_2021-09-23_AY.44
MA-CDCBI-CRSP_O4CNGYTMJOC35B7S_2021-09-09_AY.44
MA-CDCBI-CRSP_3ZMFS7VCO5EUCHFZ_2021-09-07_AY.44
MA-CDCBI-CRSP_QJMRPM4BQFLPQRTW_2021-09-07_AY.44
MA-UMASSMED-202007558_2021-09-18_AY.44
MA-CDCBI-CRSP_FPPPWXRJN5DAA57F_2021-09-17_AY.44
MA-CDCBI-CRSP_HZEOG5VTUFACHAOS_2021-09-13_AY.44
MA-CDCBI-CRSP_CDTDKUFUOHRRGRCJ_2021-09-07_AY.44
MA-CDCBI-CRSP_PZRVSK2R5RWVUW64_2021-09-10_AY.44
MA-CDCBI-CRSP_EGZPOMS7ILUQJ7LE_2021-09-14_AY.44
MA-CDCBI-CRSP_OBNRYCF2CRLMZI5L_2021-09-05_AY.44
OM076751_MA-CDCBI-CRSP_ZV7WP6A4TWJ3LEM3_2021-12-15_AY.44
OL910614_MA-CDCBI-CRSP_M3FQ7OBC2G3LTFYZ_2021-12-08_AY.44
OL923636_MA-CDC-ASC210402266_2021-11-15_AY.44
OL906624_MA-CDCBI-CRSP_DCRQKFFBHPRO5TT7_2021-12-05_AY.44
OL822564_MA-CDCBI-CRSP_ENQJ2MBYYDGU7IVS_2021-12-01_AY.44
OL713883_MA-CDCBI-CRSP_COQBJNWG5YQCRFC7_2021-11-18_AY.44
OL944888_MA-CDC-ASC210654508_2021-11-26_AY.44
OL846789_MA-CDCBI-CRSP_AQBW632VLD277U3Y_2021-12-02_AY.44
OL728900_MA-CDC-STM-KKQYEFUPP_2021-11-20_AY.44
OL984688_MA-CDCBI-CRSP_OUZWIQ3DFNO3SKDS_2021-12-10_AY.44
OL713866_MA-CDCBI-CRSP_BCFB3VS4QL76GJQK_2021-11-18_AY.44
OL727884_MA-CDC-STM-CYGPPJE6D_2021-11-17_AY.44
OL890482_MA-CDCBI-CRSP_U6JBANPY2MG6MWQT_2021-12-03_AY.44
MA-CDCBI-CRSP_U6JBANPY2MG6MWQT_2021-12-03_AY.44
MA-CDC-ASC210425936_2021-11-17_AY.44
OL761113_MA-CDC-ASC210425936_2021-11-17_AY.44
OL929928_MA-CDC-ASC210425936_2021-11-17_AY.44
OL832445_MA-CDC-LC0398578_2021-11-15_AY.44
MA-CDCBI-CRSP_4F5V67PX75FUSUCO_2021-11-13_AY.44
MA-CDC-LC0398578_2021-11-15_AY.44
OL834400_MA-CDC-LC0400148_2021-11-20_AY.44
OL880850_MA-CDCBI-CRSP_NFTILGDO2EGL5UC7_2021-12-02_AY.44
MA-CDCBI-CRSP_TXLFIUES3OZR2PW5_2021-10-05_AY.44
MA-CDCBI-CRSP_IXB3YHOFJTQQSOPY_2021-09-28_AY.44
OL802416_MA-CDCBI-CRSP_FW3R7EQKUF5Q4BVA_2021-11-29_AY.44
MA-CDCBI-CRSP_2BTUHZZVJ4LU3P54_2021-09-30_AY.44
OL731073_MA-CDC-ASC210524884_2021-11-06_AY.44
MA-CDC-STM-J9XA57S9F_2021-11-10_AY.44
MA-CDCBI-CRSP_UAIBKNT7HJR5TOBJ_2021-10-13_AY.44
MA-CDCBI-CRSP_SIDKAVNCGXIP4UB6_2021-09-20_AY.44
MA-CDCBI-CRSP_YSVAEZFILZVCDZE7_2021-09-13_AY.44
MA-CDCBI-CRSP_ASKVJ2LYJPZYUO62_2021-09-13_AY.44
MA-CDCBI-CRSP_RS5VYKE3VBPSPJ4Q_2021-09-07_AY.44
MA-CDC-QDX28509004_2021-09-04_AY.44
MA-CDCBI-CRSP_R3XNJDMK56HWMHUI_2021-09-07_AY.44
MA-CDCBI-CRSP_KHC53LV3IRICIY3T_2021-09-08_AY.44
MA-CDC-STM-000728026_2021-09-04_AY.44
MA-CDCBI-CRSP_SM6RAWR5GFJ55PTT_2021-09-01_AY.44
MA-CDCBI-CRSP_ONQC7RMHGHO47ZII_2021-09-03_AY.44
MA-CDCBI-CRSP_GRGRMSPYHAWB72I5_2021-09-04_AY.44
MA-NEIDL-01731_2021-09-01_AY.44
MA-CDCBI-CRSP_IOZIWNPZ6OX5II4G_2021-08-30_AY.44
MA-CDCBI-CRSP_ADDA3PZ4WTP4OX3I_2021-09-02_AY.44
MA-CDCBI-CRSP_ZGRFOMC7CFDIDDD7_2021-10-10_AY.44
MA-CDCBI-CRSP_B6YWK226KAS3AKYW_2021-10-07_AY.44
MA-CDCBI-CRSP_SZL5QJGU6D4KWBVJ_2021-10-06_AY.44
MA-CDCBI-CRSP_YL7J3BECW5SXC6R7_2021-09-28_AY.44
MA-CDCBI-CRSP_K2SDOYGBN5BMZSCN_2021-10-04_AY.44
MA-CDCBI-CRSP_6QUF6KCIGEI47IND_2021-09-22_AY.44
MA-CDCBI-CRSP_5HBYBLGPQNTGOXNE_2021-09-24_AY.44
MA-CDCBI-CRSP_NAVG62QFJWZD2VLM_2021-10-03_AY.44
MA-CDC-LC0326473_2021-10-02_AY.44
MA-CDCBI-CRSP_S5WQIBCQKBMHBCSW_2021-09-19_AY.44
MA-CDCBI-CRSP_EEAGMKSYDRYUXFTO_2021-09-22_AY.44
MA-CDCBI-CRSP_3FLKLDDTLH6QUIQL_2021-09-11_AY.44
MA-CDCBI-CRSP_IRK2BJS53ZIUTBB7_2021-09-09_AY.44
MA-CDCBI-CRSP_6FEUV6CBY62Q6JV3_2021-09-02_AY.44
OM010934_MA-CDC-ASC210535034_2021-12-13_AY.44
MA-CDCBI-CRSP_5PLQZI5RC6ZUBF2K_2021-10-01_AY.44
MA-UMASSMED-202006543_2021-09-02_AY.44
MA-CDCBI-CRSP_H4I2IHP263HSOOHG_2021-09-02_AY.44
MA-CDCBI-CRSP_XSYUHAMFC7NS7PPV_2021-08-30_AY.44
MA-CDCBI-CRSP_ISDUACFJD2R77T63_2021-08-26_AY.44
22-003833-032_MA_Dukes_2021-12-04_AY.44
22-000890-021_MA_Dukes_2021-12-01_AY.44
22-002350-029_MA_Dukes_2021-12-03_AY.44
MA-CDC-QDX26675842_2021-07-08_AY.44
OL985402_MA-CDCBI-CRSP_T3ROQKBPOAWSI55P_2021-12-12_AY.44
OL910375_MA-CDCBI-CRSP_3GNBZBX7X3YYZHLL_2021-12-08_AY.44
MA-CDCBI-CRSP_3GNBZBX7X3YYZHLL_2021-12-08_AY.44
MA-CDCBI-CRSP_QLYLW2AMHV5VI6VT_2021-12-08_AY.44
MA-CDC-ASC210568553_2021-12-26_AY.44
OL910681_MA-CDCBI-CRSP_QLYLW2AMHV5VI6VT_2021-12-08_AY.44
MA-CDCBI-CRSP_T3ROQKBPOAWSI55P_2021-12-12_AY.44
MA-MASPHL-10061_2022-01-11_AY.44
MA-CDCBI-CRSP_N4V6CBBF3XWR6LFT_2021-12-06_AY.44
MA-CDCBI-CRSP_KVIR7HTK6IHREMMT_2021-12-08_AY.44
MA-CDCBI-CRSP_XWT6TTFVJKGJMA7T_2021-12-30_AY.44
MA-CDCBI-CRSP_ZFOEJOPRIVMRZBYC_2021-12-08_AY.44
MA-CDCBI-CRSP_VIUJY5M454D5IAQI_2022-01-03_AY.44
MA-CDCBI-CRSP_U4QO3BJMSHHIOSEK_2021-12-29_AY.44
OL910598_MA-CDCBI-CRSP_KVIR7HTK6IHREMMT_2021-12-08_AY.44
OL786112_MA-CDC-ASC210395266_2021-11-10_AY.44
MA-CDC-ASC210395266_2021-11-10_AY.44
MA-CDCBI-CRSP_OY6QGG3242WMCSVM_2021-10-13_AY.44
OL985353_MA-CDCBI-CRSP_Q6L5KZP5WSSCQYMZ_2021-12-12_AY.44
OL907035_MA-CDCBI-CRSP_YCE5PFFD6FHY2IYA_2021-12-05_AY.44
OL969338_MA-CDC-ASC210607903_2021-12-04_AY.44
OM126974_MA-CDC-ASC210613287_2021-12-16_AY.44
OL968866_MA-CDC-ASC210517269_2021-12-02_AY.44
OM072407_MA-CDCBI-CRSP_YQNL7U6QWXSLO2KJ_2021-12-18_AY.44
OL985389_MA-CDCBI-CRSP_SFOGINZRGNMNF7KT_2021-12-04_AY.44
OL968936_MA-CDC-ASC210518139_2021-12-02_AY.44
OL921439_MA-CDC-ASC210397130_2021-11-10_AY.44
MA-CDC-ASC210392129_2021-11-07_AY.44_99.97%
22-003833-028_MA_Middlesex_2021-12-04_AY.44
OL923743_MA-CDC-ASC210484708_2021-11-17_AY.44
OM147112_MA-CDC-QDX32006632_2021-12-07_AY.44
OM147110_MA-CDC-QDX32006631_2021-12-07_AY.44
OL906726_MA-CDCBI-CRSP_IXPUS4GYKSPJ6IUA_2021-12-06_AY.44
OL822119_MA-CDCBI-CRSP_VPGL635UQD56F6UC_2021-12-01_AY.44
OL717839_MA-CDCBI-CRSP_ZMZV4IWKTCOKP5NE_2021-11-19_AY.44
OM098730_MA-CDC-ASC210632291_2021-12-19_AY.44
OL717771_MA-CDCBI-CRSP_TQXPFLM4F6JSAGTQ_2021-11-19_AY.44
OL717272_MA-CDCBI-CRSP_WZE4E4I7XDESNQN5_2021-11-19_AY.44
OM073189_MA-CDCBI-CRSP_3PRNKMOJUXKUCCRP_2021-12-20_AY.44
OL896627_MA-CDC-STM-NAUWJ34Q4_2021-11-23_AY.44
OL752261_MA-CDC-STM-NAUWJ34Q4_2021-11-23_AY.44
OL896644_MA-CDC-STM-HR6SDJ4JP_2021-11-23_AY.44
OL752278_MA-CDC-STM-HR6SDJ4JP_2021-11-23_AY.44
OL847021_MA-CDCBI-CRSP_NBJQGU7V2L7TONIO_2021-12-02_AY.44
OM184095_MA-CDCBI-CRSP_TTHWDIDE4IHRGDCZ_2021-12-29_AY.44
OL718326_MA-CDCBI-CRSP_KQQQANBMLC6GMUGX_2021-11-21_AY.44
MA-CDCBI-CRSP_RRD74PM4ZIVEMU23_2021-09-09_AY.44
MA-CDCBI-CRSP_RMPC52LTIGWX7WBB_2021-09-10_AY.44
MA-CDCBI-CRSP_TY6OBB4FYNU5KQ5E_2021-09-24_AY.44
MA-CDCBI-CRSP_WV3YS3DJFIRAYXGO_2021-09-08_AY.44
MA-CDCBI-CRSP_AB54S5RD7WJCDRRW_2021-09-08_AY.44
MA-CDCBI-CRSP_7PVH2CNFDS6BBUTP_2021-09-04_AY.44
MA-CDCBI-CRSP_RNBFT2ZWTW5JG3SN_2021-08-28_AY.44
MA-CDCBI-CRSP_G2QCHA4IZNCMNDAJ_2021-08-24_AY.44
MA-CDCBI-CRSP_TES64U7OQFS4CUKJ_2021-08-26_AY.44
MA-CDCBI-CRSP_ZWS5HRPZDHICW3XM_2021-09-08_AY.44
MA-CDCBI-CRSP_WBZICC2HUG4YGWR3_2021-09-16_AY.44
MA-CDCBI-CRSP_5DRTPCVQACKOMNCK_2021-09-14_AY.44
MA-CDC-LC0363876_2021-10-31_AY.44
MA-CDCBI-CRSP_Y7L4PYHW74AOVVJ5_2021-08-31_AY.44
MA-CDCBI-CRSP_GEZGZVYWCRQCQAQ6_2021-09-09_AY.44
MA-CDCBI-CRSP_G4VAEIMLXVDOLS5I_2021-08-30_AY.44
MA-CDC-LC0256800_2021-09-03_AY.44
MA-CDCBI-CRSP_EOMNNQKGDUHDXAFZ_2021-08-30_AY.44
MA-CDC-LC0187288_2021-08-10_AY.44
MA-CDC-QDX27788393_2021-08-10_AY.44
MA-CDC-QDX26380806_2021-06-28_AY.44
MA-CDC-QDX26380797_2021-06-27_AY.44
MN908947 (Wuhan-Hu-1/2019)
0.2

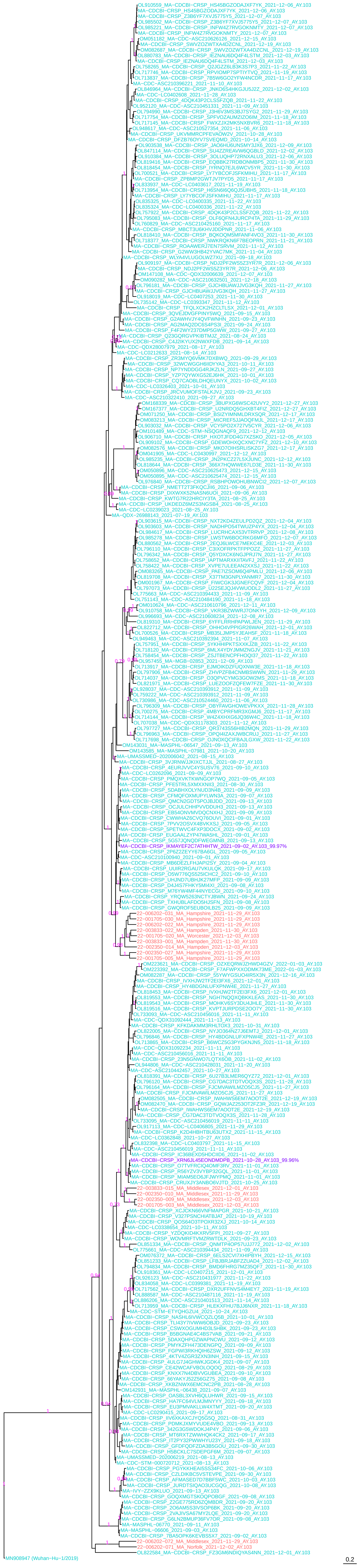
MN908947 (Wuhan-Hu-1/2019)
0.2

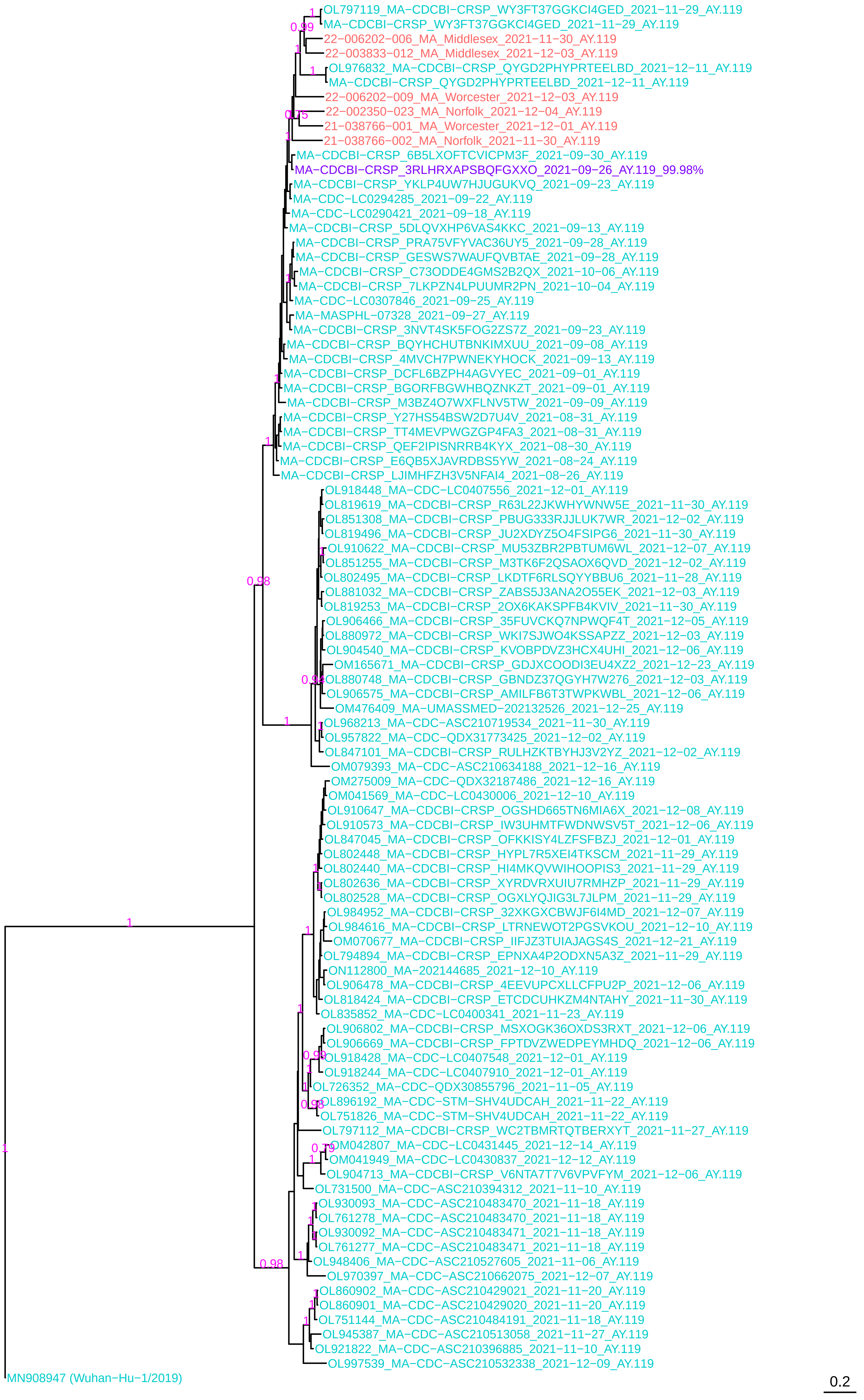
OL797119_MA−CDCBI−CRSP_WY3FT37GGKCI4GED_2021−11−29_AY.119
MA−CDCBI−CRSP_WY3FT37GGKCI4GED_2021−11−29_AY.119
22−006202−006_MA_Middlesex_2021−11−30_AY.119
22−003833−012_MA_Middlesex_2021−12−03_AY.119
OL976832_MA−CDCBI−CRSP_QYGD2PHYPRTEELBD_2021−12−11_AY.119
MA−CDCBI−CRSP_QYGD2PHYPRTEELBD_2021−12−11_AY.119
22−006202−009_MA_Worcester_2021−12−03_AY.119
22−002350−023_MA_Norfolk_2021−12−04_AY.119
21−038766−001_MA_Worcester_2021−12−01_AY.119
21−038766−002_MA_Norfolk_2021−11−30_AY.119
MA−CDCBI−CRSP_6B5LXOFTCVICPM3F_2021−09−30_AY.119
MA−CDCBI−CRSP_3RLHRXAPSBQFGXXO_2021−09−26_AY.119_99.98%
MA−CDCBI−CRSP_YKLP4UW7HJUGUKVQ_2021−09−23_AY.119
MA−CDC−LC0294285_2021−09−22_AY.119
MA−CDC−LC0290421_2021−09−18_AY.119
MA−CDCBI−CRSP_5DLQVXHP6VAS4KKC_2021−09−13_AY.119
MA−CDCBI−CRSP_PRA75VFYVAC36UY5_2021−09−28_AY.119
MA−CDCBI−CRSP_GESWS7WAUFQVBTAE_2021−09−28_AY.119
MA−CDCBI−CRSP_C73ODDE4GMS2B2QX_2021−10−06_AY.119
MA−CDCBI−CRSP_7LKPZN4LPUUMR2PN_2021−10−04_AY.119
MA−CDC−LC0307846_2021−09−25_AY.119
MA−MASPHL−07328_2021−09−27_AY.119
MA−CDCBI−CRSP_3NVT4SK5FOG2ZS7Z_2021−09−23_AY.119
MA−CDCBI−CRSP_BQYHCHUTBNKIMXUU_2021−09−08_AY.119
MA−CDCBI−CRSP_4MVCH7PWNEKYHOCK_2021−09−13_AY.119
MA−CDCBI−CRSP_DCFL6BZPH4AGVYEC_2021−09−01_AY.119
MA−CDCBI−CRSP_BGORFBGWHBQZNKZT_2021−09−01_AY.119
MA−CDCBI−CRSP_M3BZ4O7WXFLNV5TW_2021−09−09_AY.119
MA−CDCBI−CRSP_Y27HS54BSW2D7U4V_2021−08−31_AY.119
MA−CDCBI−CRSP_TT4MEVPWGZGP4FA3_2021−08−31_AY.119
MA−CDCBI−CRSP_QEF2IPISNRRB4KYX_2021−08−30_AY.119
MA−CDCBI−CRSP_E6QB5XJAVRDBS5YW_2021−08−24_AY.119
MA−CDCBI−CRSP_LJIMHFZH3V5NFAI4_2021−08−26_AY.119
OL918448_MA−CDC−LC0407556_2021−12−01_AY.119
OL819619_MA−CDCBI−CRSP_R63L22JKWHYWNW5E_2021−11−30_AY.119
OL851308_MA−CDCBI−CRSP_PBUG333RJJLUK7WR_2021−12−02_AY.119
OL819496_MA−CDCBI−CRSP_JU2XDYZ5O4FSIPG6_2021−11−30_AY.119
OL910622_MA−CDCBI−CRSP_MU53ZBR2PBTUM6WL_2021−12−07_AY.119
OL851255_MA−CDCBI−CRSP_M3TK6F2QSAOX6QVD_2021−12−02_AY.119
OL802495_MA−CDCBI−CRSP_LKDTF6RLSQYYBBU6_2021−11−28_AY.119
OL881032_MA−CDCBI−CRSP_ZABS5J3ANA2O55EK_2021−12−03_AY.119
OL819253_MA−CDCBI−CRSP_2OX6KAKSPFB4KVIV_2021−11−30_AY.119
OL906466_MA−CDCBI−CRSP_35FUVCKQ7NPWQF4T_2021−12−05_AY.119
OL880972_MA−CDCBI−CRSP_WKI7SJWO4KSSAPZZ_2021−12−03_AY.119
OL904540_MA−CDCBI−CRSP_KVOBPDVZ3HCX4UHI_2021−12−06_AY.119
OM165671_MA−CDCBI−CRSP_GDJXCOODI3EU4XZ2_2021−12−23_AY.119
OL880748_MA−CDCBI−CRSP_GBNDZ37QGYH7W276_2021−12−03_AY.119
OL906575_MA−CDCBI−CRSP_AMILFB6T3TWPKWBL_2021−12−06_AY.119
OM476409_MA−UMASSMED−202132526_2021−12−25_AY.119
OL968213_MA−CDC−ASC210719534_2021−11−30_AY.119
OL957822_MA−CDC−QDX31773425_2021−12−02_AY.119
OL847101_MA−CDCBI−CRSP_RULHZKTBYHJ3V2YZ_2021−12−02_AY.119
OM079393_MA−CDC−ASC210634188_2021−12−16_AY.119
OM275009_MA−CDC−QDX32187486_2021−12−16_AY.119
OM041569_MA−CDC−LC0430006_2021−12−10_AY.119
OL910647_MA−CDCBI−CRSP_OGSHD665TN6MIA6X_2021−12−08_AY.119
OL910573_MA−CDCBI−CRSP_IW3UHMTFWDNWSV5T_2021−12−06_AY.119
OL847045_MA−CDCBI−CRSP_OFKKISY4LZFSFBZJ_2021−12−01_AY.119
OL802448_MA−CDCBI−CRSP_HYPL7R5XEI4TKSCM_2021−11−29_AY.119
OL802440_MA−CDCBI−CRSP_HI4MKQVWIHOOPIS3_2021−11−29_AY.119
OL802636_MA−CDCBI−CRSP_XYRDVRXUIU7RMHZP_2021−11−29_AY.119
OL802528_MA−CDCBI−CRSP_OGXLYQJIG3L7JLPM_2021−11−29_AY.119
OL984952_MA−CDCBI−CRSP_32XKGXCBWJF6I4MD_2021−12−07_AY.119
OL984616_MA−CDCBI−CRSP_LTRNEWOT2PGSVKOU_2021−12−10_AY.119
OM070677_MA−CDCBI−CRSP_IIFJZ3TUIAJAGS4S_2021−12−21_AY.119
OL794894_MA−CDCBI−CRSP_EPNXA4P2ODXN5A3Z_2021−11−29_AY.119
ON112800_MA−202144685_2021−12−10_AY.119
OL906478_MA−CDCBI−CRSP_4EEVUPCXLLCFPU2P_2021−12−06_AY.119
OL818424_MA−CDCBI−CRSP_ETCDCUHKZM4NTAHY_2021−11−30_AY.119
OL835852_MA−CDC−LC0400341_2021−11−23_AY.119
OL906802_MA−CDCBI−CRSP_MSXOGK36OXDS3RXT_2021−12−06_AY.119
OL906669_MA−CDCBI−CRSP_FPTDVZWEDPEYMHDQ_2021−12−06_AY.119
OL918428_MA−CDC−LC0407548_2021−12−01_AY.119
OL918244_MA−CDC−LC0407910_2021−12−01_AY.119
OL726352_MA−CDC−QDX30855796_2021−11−05_AY.119
OL896192_MA−CDC−STM−SHV4UDCAH_2021−11−22_AY.119
OL751826_MA−CDC−STM−SHV4UDCAH_2021−11−22_AY.119
OL797112_MA−CDCBI−CRSP_WC2TBMRTQTBERXYT_2021−11−27_AY.119
OM042807_MA−CDC−LC0431445_2021−12−14_AY.119
OM041949_MA−CDC−LC0430837_2021−12−12_AY.119
OL904713_MA−CDCBI−CRSP_V6NTA7T7V6VPVFYM_2021−12−06_AY.119
OL731500_MA−CDC−ASC210394312_2021−11−10_AY.119
OL930093_MA−CDC−ASC210483470_2021−11−18_AY.119
OL761278_MA−CDC−ASC210483470_2021−11−18_AY.119
OL930092_MA−CDC−ASC210483471_2021−11−18_AY.119
OL761277_MA−CDC−ASC210483471_2021−11−18_AY.119
OL948406_MA−CDC−ASC210527605_2021−11−06_AY.119
OL970397_MA−CDC−ASC210662075_2021−12−07_AY.119
OL860902_MA−CDC−ASC210429021_2021−11−20_AY.119
OL860901_MA−CDC−ASC210429020_2021−11−20_AY.119
OL751144_MA−CDC−ASC210484191_2021−11−18_AY.119
OL945387_MA−CDC−ASC210513058_2021−11−27_AY.119
OL921822_MA−CDC−ASC210396885_2021−11−10_AY.119
OL997539_MA−CDC−ASC210532338_2021−12−09_AY.119
MN908947 (Wuhan−Hu−1/2019)
0.2

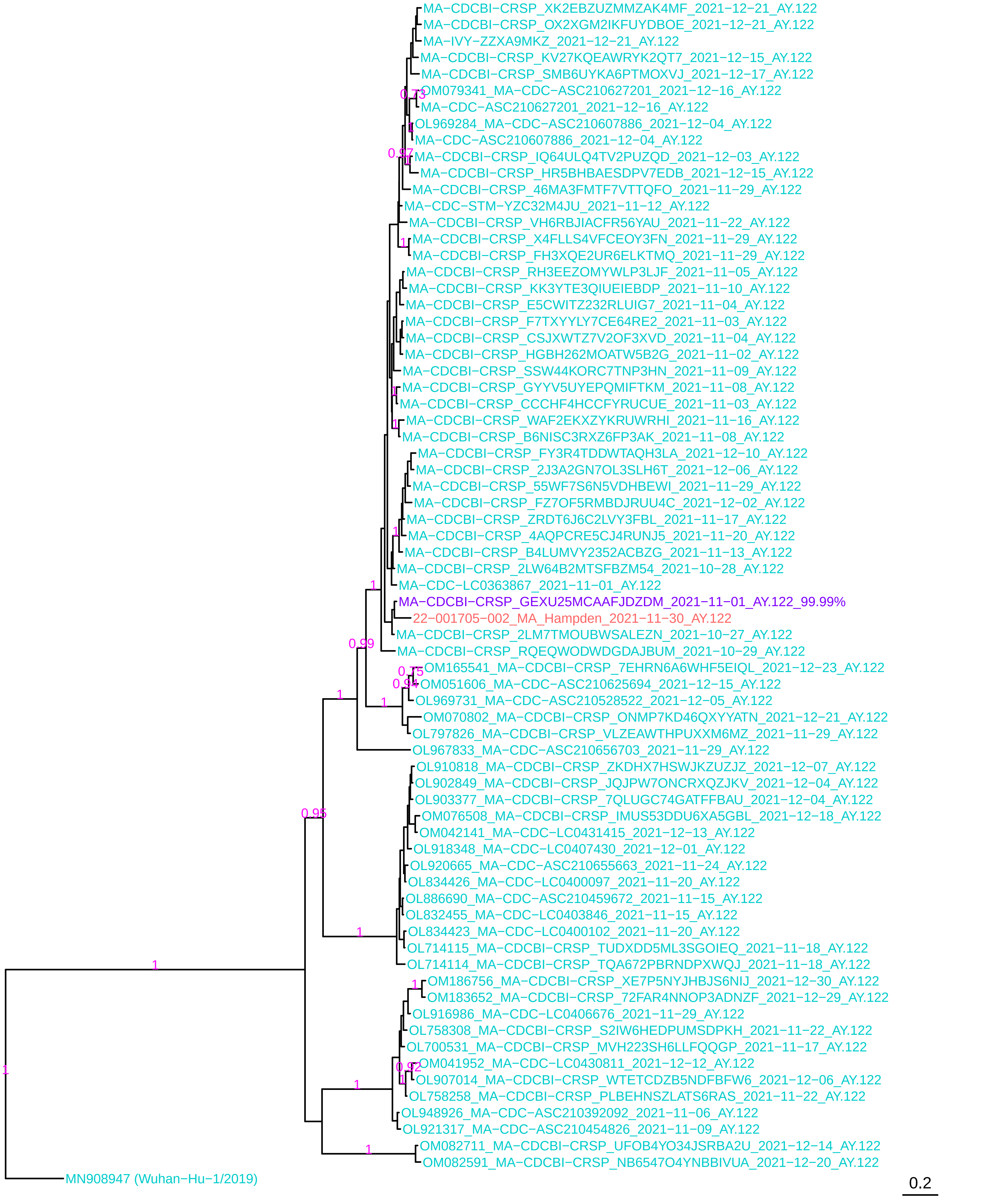
MA−CDCBI−CRSP_XK2EBZUZMMZAK4MF_2021−12−21_AY.122
MA−CDCBI−CRSP_OX2XGM2IKFUYDBOE_2021−12−21_AY.122
MA−IVY−ZZXA9MKZ_2021−12−21_AY.122
MA−CDCBI−CRSP_KV27KQEAWRYK2QT7_2021−12−15_AY.122
MA−CDCBI−CRSP_SMB6UYKA6PTMOXVJ_2021−12−17_AY.122
0.73
OM079341_MA−CDC−ASC210627201_2021−12−16_AY.122
MA−CDC−ASC210627201_2021−12−16_AY.122
1
OL969284_MA−CDC−ASC210607886_2021−12−04_AY.122
MA−CDC−ASC210607886_2021−12−04_AY.122
0.97
MA−CDCBI−CRSP_IQ64ULQ4TV2PUZQD_2021−12−03_AY.122
MA−CDCBI−CRSP_HR5BHBAESDPV7EDB_2021−12−15_AY.122
MA−CDCBI−CRSP_46MA3FMTF7VTTQFO_2021−11−29_AY.122
MA−CDC−STM−YZC32M4JU_2021−11−12_AY.122
MA−CDCBI−CRSP_VH6RBJIACFR56YAU_2021−11−22_AY.122
1
MA−CDCBI−CRSP_X4FLLS4VFCEOY3FN_2021−11−29_AY.122
MA−CDCBI−CRSP_FH3XQE2UR6ELKTMQ_2021−11−29_AY.122
MA−CDCBI−CRSP_RH3EEZOMYWLP3LJF_2021−11−05_AY.122
MA−CDCBI−CRSP_KK3YTE3QIUEIEBDP_2021−11−10_AY.122
MA−CDCBI−CRSP_E5CWITZ232RLUIG7_2021−11−04_AY.122
MA−CDCBI−CRSP_F7TXYYLY7CE64RE2_2021−11−03_AY.122
MA−CDCBI−CRSP_CSJXWTZ7V2OF3XVD_2021−11−04_AY.122
MA−CDCBI−CRSP_HGBH262MOATW5B2G_2021−11−02_AY.122
MA−CDCBI−CRSP_SSW44KORC7TNP3HN_2021−11−09_AY.122
1
MA−CDCBI−CRSP_GYYV5UYEPQMIFTKM_2021−11−08_AY.122
MA−CDCBI−CRSP_CCCHF4HCCFYRUCUE_2021−11−03_AY.122
1
MA−CDCBI−CRSP_WAF2EKXZYKRUWRHI_2021−11−16_AY.122
MA−CDCBI−CRSP_B6NISC3RXZ6FP3AK_2021−11−08_AY.122
MA−CDCBI−CRSP_FY3R4TDDWTAQH3LA_2021−12−10_AY.122
MA−CDCBI−CRSP_2J3A2GN7OL3SLH6T_2021−12−06_AY.122
MA−CDCBI−CRSP_55WF7S6N5VDHBEWI_2021−11−29_AY.122
MA−CDCBI−CRSP_FZ7OF5RMBDJRUU4C_2021−12−02_AY.122
MA−CDCBI−CRSP_ZRDT6J6C2LVY3FBL_2021−11−17_AY.122
1
MA−CDCBI−CRSP_4AQPCRE5CJ4RUNJ5_2021−11−20_AY.122
MA−CDCBI−CRSP_B4LUMVY2352ACBZG_2021−11−13_AY.122
MA−CDCBI−CRSP_2LW64B2MTSFBZM54_2021−10−28_AY.122
1
MA−CDC−LC0363867_2021−11−01_AY.122
MA−CDCBI−CRSP_GEXU25MCAAFJDZDM_2021−11−01_AY.122_99.99%
22−001705−002_MA_Hampden_2021−11−30_AY.122
0.99
MA−CDCBI−CRSP_2LM7TMOUBWSALEZN_2021−10−27_AY.122
MA−CDCBI−CRSP_RQEQWODWDGDAJBUM_2021−10−29_AY.122
0.75
OM165541_MA−CDCBI−CRSP_7EHRN6A6WHF5EIQL_2021−12−23_AY.122
0.94
OM051606_MA−CDC−ASC210625694_2021−12−15_AY.122
1
1
OL969731_MA−CDC−ASC210528522_2021−12−05_AY.122
OM070802_MA−CDCBI−CRSP_ONMP7KD46QXYYATN_2021−12−21_AY.122
OL797826_MA−CDCBI−CRSP_VLZEAWTHPUXXM6MZ_2021−11−29_AY.122
OL967833_MA−CDC−ASC210656703_2021−11−29_AY.122
OL910818_MA−CDCBI−CRSP_ZKDHX7HSWJKZUZJZ_2021−12−07_AY.122
OL902849_MA−CDCBI−CRSP_JQJPW7ONCRXQZJKV_2021−12−04_AY.122
OL903377_MA−CDCBI−CRSP_7QLUGC74GATFFBAU_2021−12−04_AY.122
0.95
OM076508_MA−CDCBI−CRSP_IMUS53DDU6XA5GBL_2021−12−18_AY.122
OM042141_MA−CDC−LC0431415_2021−12−13_AY.122
OL918348_MA−CDC−LC0407430_2021−12−01_AY.122
OL920665_MA−CDC−ASC210655663_2021−11−24_AY.122
OL834426_MA−CDC−LC0400097_2021−11−20_AY.122
OL886690_MA−CDC−ASC210459672_2021−11−15_AY.122
OL832455_MA−CDC−LC0403846_2021−11−15_AY.122
1
OL834423_MA−CDC−LC0400102_2021−11−20_AY.122
OL714115_MA−CDCBI−CRSP_TUDXDD5ML3SGOIEQ_2021−11−18_AY.122
1
OL714114_MA−CDCBI−CRSP_TQA672PBRNDPXWQJ_2021−11−18_AY.122
1
OM186756_MA−CDCBI−CRSP_XE7P5NYJHBJS6NIJ_2021−12−30_AY.122
OM183652_MA−CDCBI−CRSP_72FAR4NNOP3ADNZF_2021−12−29_AY.122
OL916986_MA−CDC−LC0406676_2021−11−29_AY.122
OL758308_MA−CDCBI−CRSP_S2IW6HEDPUMSDPKH_2021−11−22_AY.122
OL700531_MA−CDCBI−CRSP_MVH223SH6LLFQQGP_2021−11−17_AY.122
0.92
OM041952_MA−CDC−LC0430811_2021−12−12_AY.122
1
1
OL907014_MA−CDCBI−CRSP_WTETCDZB5NDFBFW6_2021−12−06_AY.122
1
OL758258_MA−CDCBI−CRSP_PLBEHNSZLATS6RAS_2021−11−22_AY.122
OL948926_MA−CDC−ASC210392092_2021−11−06_AY.122
OL921317_MA−CDC−ASC210454826_2021−11−09_AY.122
1
OM082711_MA−CDCBI−CRSP_UFOB4YO34JSRBA2U_2021−12−14_AY.122
OM082591_MA−CDCBI−CRSP_NB6547O4YNBBIVUA_2021−12−20_AY.122
MN908947 (Wuhan−Hu−1/2019)
0.2

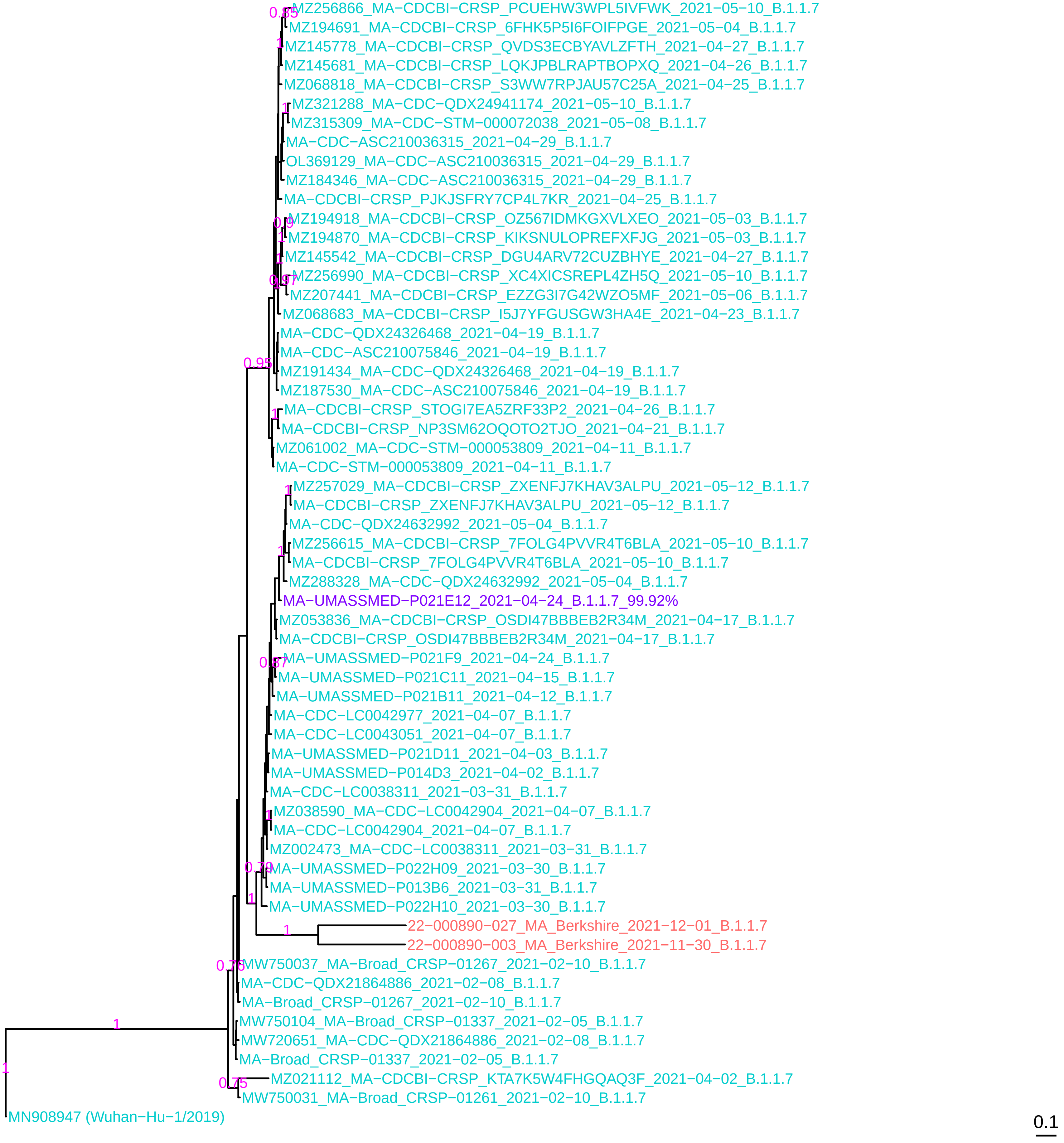
MZ256866_MA−CDCBI−CRSP_PCUEHW3WPL5IVFWK_2021−05−10_B.1.1.7
MZ194691_MA−CDCBI−CRSP_6FHK5P5I6FOIFPGE_2021−05−04_B.1.1.7
MZ145778_MA−CDCBI−CRSP_QVDS3ECBYAVLZFTH_2021−04−27_B.1.1.7
MZ145681_MA−CDCBI−CRSP_LQKJPBLRAPTBOPXQ_2021−04−26_B.1.1.7
MZ068818_MA−CDCBI−CRSP_S3WW7RPJAU57C25A_2021−04−25_B.1.1.7
MZ321288_MA−CDC−QDX24941174_2021−05−10_B.1.1.7
MZ315309_MA−CDC−STM−000072038_2021−05−08_B.1.1.7
MA−CDC−ASC210036315_2021−04−29_B.1.1.7
OL369129_MA−CDC−ASC210036315_2021−04−29_B.1.1.7
MZ184346_MA−CDC−ASC210036315_2021−04−29_B.1.1.7
MA−CDCBI−CRSP_PJKJSFRY7CP4L7KR_2021−04−25_B.1.1.7
MZ194918_MA−CDCBI−CRSP_OZ567IDMKGXVLXEO_2021−05−03_B.1.1.7
MZ194870_MA−CDCBI−CRSP_KIKSNULOPREFXFJG_2021−05−03_B.1.1.7
MZ145542_MA−CDCBI−CRSP_DGU4ARV72CUZBHYE_2021−04−27_B.1.1.7
MZ256990_MA−CDCBI−CRSP_XC4XICSREPL4ZH5Q_2021−05−10_B.1.1.7
MZ207441_MA−CDCBI−CRSP_EZZG3I7G42WZO5MF_2021−05−06_B.1.1.7
MZ068683_MA−CDCBI−CRSP_I5J7YFGUSGW3HA4E_2021−04−23_B.1.1.7
MA−CDC−QDX24326468_2021−04−19_B.1.1.7
MA−CDC−ASC210075846_2021−04−19_B.1.1.7
MZ191434_MA−CDC−QDX24326468_2021−04−19_B.1.1.7
MZ187530_MA−CDC−ASC210075846_2021−04−19_B.1.1.7
MA−CDCBI−CRSP_STOGI7EA5ZRF33P2_2021−04−26_B.1.1.7
MA−CDCBI−CRSP_NP3SM62OQOTO2TJO_2021−04−21_B.1.1.7
MZ061002_MA−CDC−STM−000053809_2021−04−11_B.1.1.7
MA−CDC−STM−000053809_2021−04−11_B.1.1.7
MZ257029_MA−CDCBI−CRSP_ZXENFJ7KHAV3ALPU_2021−05−12_B.1.1.7
MA−CDCBI−CRSP_ZXENFJ7KHAV3ALPU_2021−05−12_B.1.1.7
MA−CDC−QDX24632992_2021−05−04_B.1.1.7
MZ256615_MA−CDCBI−CRSP_7FOLG4PVVR4T6BLA_2021−05−10_B.1.1.7
MA−CDCBI−CRSP_7FOLG4PVVR4T6BLA_2021−05−10_B.1.1.7
MZ288328_MA−CDC−QDX24632992_2021−05−04_B.1.1.7
MA−UMASSMED−P021E12_2021−04−24_B.1.1.7_99.92%
MZ053836_MA−CDCBI−CRSP_OSDI47BBBEB2R34M_2021−04−17_B.1.1.7
MA−CDCBI−CRSP_OSDI47BBBEB2R34M_2021−04−17_B.1.1.7
MA−UMASSMED−P021F9_2021−04−24_B.1.1.7
MA−UMASSMED−P021C11_2021−04−15_B.1.1.7
MA−UMASSMED−P021B11_2021−04−12_B.1.1.7
MA−CDC−LC0042977_2021−04−07_B.1.1.7
MA−CDC−LC0043051_2021−04−07_B.1.1.7
MA−UMASSMED−P021D11_2021−04−03_B.1.1.7
MA−UMASSMED−P014D3_2021−04−02_B.1.1.7
MA−CDC−LC0038311_2021−03−31_B.1.1.7
MZ038590_MA−CDC−LC0042904_2021−04−07_B.1.1.7
MA−CDC−LC0042904_2021−04−07_B.1.1.7
MZ002473_MA−CDC−LC0038311_2021−03−31_B.1.1.7
MA−UMASSMED−P022H09_2021−03−30_B.1.1.7
MA−UMASSMED−P013B6_2021−03−31_B.1.1.7
MA−UMASSMED−P022H10_2021−03−30_B.1.1.7
22−000890−027_MA_Berkshire_2021−12−01_B.1.1.7
22−000890−003_MA_Berkshire_2021−11−30_B.1.1.7
MW750037_MA−Broad_CRSP−01267_2021−02−10_B.1.1.7
MA−CDC−QDX21864886_2021−02−08_B.1.1.7
MA−Broad_CRSP−01267_2021−02−10_B.1.1.7
MW750104_MA−Broad_CRSP−01337_2021−02−05_B.1.1.7
MW720651_MA−CDC−QDX21864886_2021−02−08_B.1.1.7
MA−Broad_CRSP−01337_2021−02−05_B.1.1.7
MZ021112_MA−CDCBI−CRSP_KTA7K5W4FHGQAQ3F_2021−04−02_B.1.1.7
MW750031_MA−Broad_CRSP−01261_2021−02−10_B.1.1.7
MN908947 (Wuhan−Hu−1/2019)
0.1

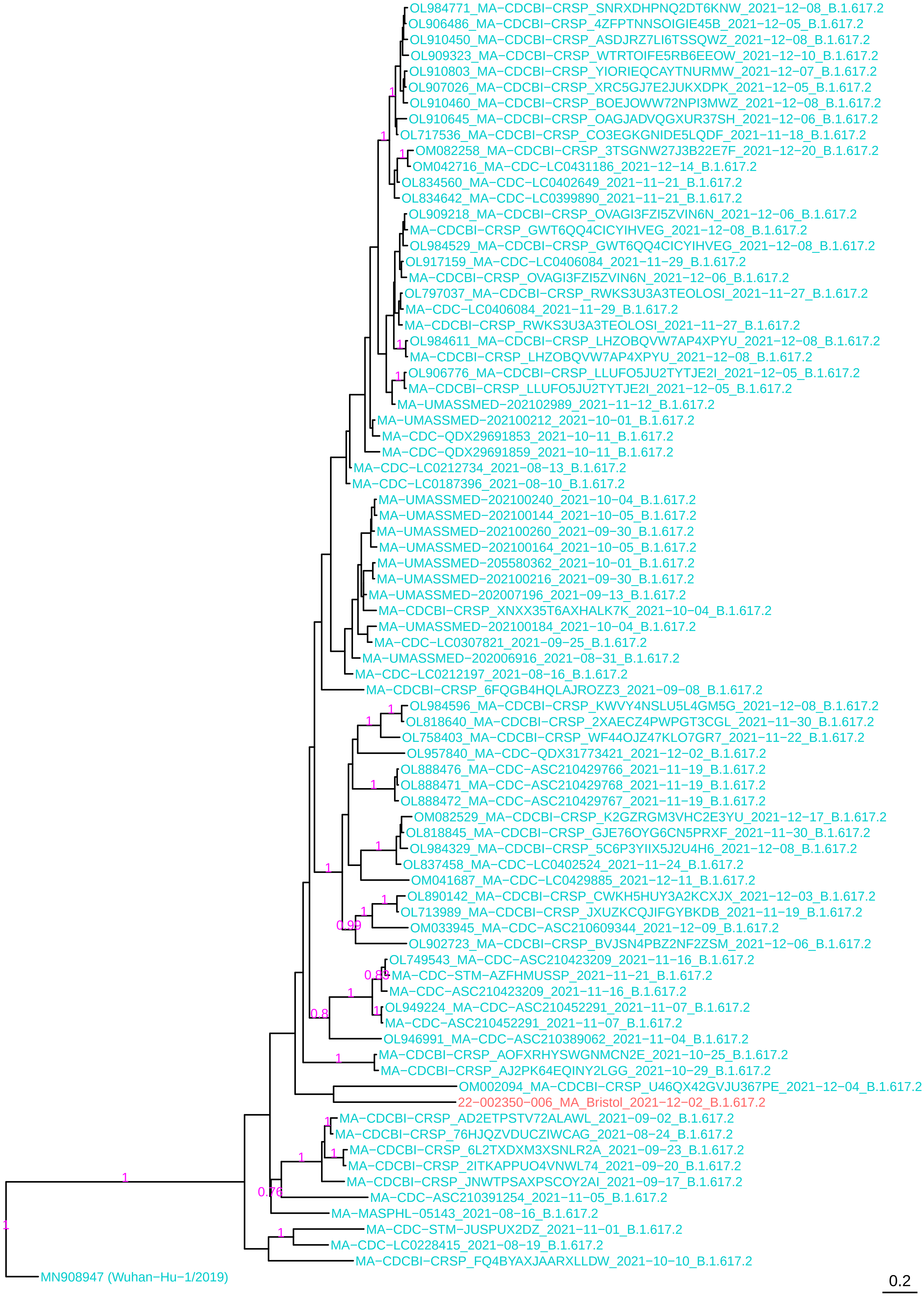
OL984771_MA−CDCBI−CRSP_SNRXDHPNQ2DT6KNW_2021−12−08_B.1.617.2
OL906486_MA−CDCBI−CRSP_4ZFPTNNSOIGIE45B_2021−12−05_B.1.617.2
OL910450_MA−CDCBI−CRSP_ASDJRZ7LI6TSSQWZ_2021−12−08_B.1.617.2
OL909323_MA−CDCBI−CRSP_WTRTOIFE5RB6EEOW_2021−12−10_B.1.617.2
OL910803_MA−CDCBI−CRSP_YIORIEQCAYTNURMW_2021−12−07_B.1.617.2
OL907026_MA−CDCBI−CRSP_XRC5GJ7E2JUKXDPK_2021−12−05_B.1.617.2
OL910460_MA−CDCBI−CRSP_BOEJOWW72NPI3MWZ_2021−12−08_B.1.617.2
OL910645_MA−CDCBI−CRSP_OAGJADVQGXUR37SH_2021−12−06_B.1.617.2
OL717536_MA−CDCBI−CRSP_CO3EGKGNIDE5LQDF_2021−11−18_B.1.617.2
OM082258_MA−CDCBI−CRSP_3TSGNW27J3B22E7F_2021−12−20_B.1.617.2
OM042716_MA−CDC−LC0431186_2021−12−14_B.1.617.2
OL834560_MA−CDC−LC0402649_2021−11−21_B.1.617.2
OL834642_MA−CDC−LC0399890_2021−11−21_B.1.617.2
OL909218_MA−CDCBI−CRSP_OVAGI3FZI5ZVIN6N_2021−12−06_B.1.617.2
MA−CDCBI−CRSP_GWT6QQ4CICYIHVEG_2021−12−08_B.1.617.2
OL984529_MA−CDCBI−CRSP_GWT6QQ4CICYIHVEG_2021−12−08_B.1.617.2
OL917159_MA−CDC−LC0406084_2021−11−29_B.1.617.2
MA−CDCBI−CRSP_OVAGI3FZI5ZVIN6N_2021−12−06_B.1.617.2
OL797037_MA−CDCBI−CRSP_RWKS3U3A3TEOLOSI_2021−11−27_B.1.617.2
MA−CDC−LC0406084_2021−11−29_B.1.617.2
MA−CDCBI−CRSP_RWKS3U3A3TEOLOSI_2021−11−27_B.1.617.2
OL984611_MA−CDCBI−CRSP_LHZOBQVW7AP4XPYU_2021−12−08_B.1.617.2
MA−CDCBI−CRSP_LHZOBQVW7AP4XPYU_2021−12−08_B.1.617.2
OL906776_MA−CDCBI−CRSP_LLUFO5JU2TYTJE2I_2021−12−05_B.1.617.2
MA−CDCBI−CRSP_LLUFO5JU2TYTJE2I_2021−12−05_B.1.617.2
MA−UMASSMED−202102989_2021−11−12_B.1.617.2
MA−UMASSMED−202100212_2021−10−01_B.1.617.2
MA−CDC−QDX29691853_2021−10−11_B.1.617.2
MA−CDC−QDX29691859_2021−10−11_B.1.617.2
MA−CDC−LC0212734_2021−08−13_B.1.617.2
MA−CDC−LC0187396_2021−08−10_B.1.617.2
MA−UMASSMED−202100240_2021−10−04_B.1.617.2
MA−UMASSMED−202100144_2021−10−05_B.1.617.2
MA−UMASSMED−202100260_2021−09−30_B.1.617.2
MA−UMASSMED−202100164_2021−10−05_B.1.617.2
MA−UMASSMED−205580362_2021−10−01_B.1.617.2
MA−UMASSMED−202100216_2021−09−30_B.1.617.2
MA−UMASSMED−202007196_2021−09−13_B.1.617.2
MA−CDCBI−CRSP_XNXX35T6AXHALK7K_2021−10−04_B.1.617.2
MA−UMASSMED−202100184_2021−10−04_B.1.617.2
MA−CDC−LC0307821_2021−09−25_B.1.617.2
MA−UMASSMED−202006916_2021−08−31_B.1.617.2
MA−CDC−LC0212197_2021−08−16_B.1.617.2
MA−CDCBI−CRSP_6FQGB4HQLAJROZZ3_2021−09−08_B.1.617.2
OL984596_MA−CDCBI−CRSP_KWVY4NSLU5L4GM5G_2021−12−08_B.1.617.2
OL818640_MA−CDCBI−CRSP_2XAECZ4PWPGT3CGL_2021−11−30_B.1.617.2
OL758403_MA−CDCBI−CRSP_WF44OJZ47KLO7GR7_2021−11−22_B.1.617.2
OL957840_MA−CDC−QDX31773421_2021−12−02_B.1.617.2
OL888476_MA−CDC−ASC210429766_2021−11−19_B.1.617.2
OL888471_MA−CDC−ASC210429768_2021−11−19_B.1.617.2
OL888472_MA−CDC−ASC210429767_2021−11−19_B.1.617.2
OM082529_MA−CDCBI−CRSP_K2GZRGM3VHC2E3YU_2021−12−17_B.1.617.2
OL818845_MA−CDCBI−CRSP_GJE76OYG6CN5PRXF_2021−11−30_B.1.617.2
OL984329_MA−CDCBI−CRSP_5C6P3YIIX5J2U4H6_2021−12−08_B.1.617.2
OL837458_MA−CDC−LC0402524_2021−11−24_B.1.617.2
OM041687_MA−CDC−LC0429885_2021−12−11_B.1.617.2
OL890142_MA−CDCBI−CRSP_CWKH5HUY3A2KCXJX_2021−12−03_B.1.617.2
OL713989_MA−CDCBI−CRSP_JXUZKCQJIFGYBKDB_2021−11−19_B.1.617.2
OM033945_MA−CDC−ASC210609344_2021−12−09_B.1.617.2
OL902723_MA−CDCBI−CRSP_BVJSN4PBZ2NF2ZSM_2021−12−06_B.1.617.2
OL749543_MA−CDC−ASC210423209_2021−11−16_B.1.617.2
MA−CDC−STM−AZFHMUSSP_2021−11−21_B.1.617.2
MA−CDC−ASC210423209_2021−11−16_B.1.617.2
OL949224_MA−CDC−ASC210452291_2021−11−07_B.1.617.2
MA−CDC−ASC210452291_2021−11−07_B.1.617.2
OL946991_MA−CDC−ASC210389062_2021−11−04_B.1.617.2
MA−CDCBI−CRSP_AOFXRHYSWGNMCN2E_2021−10−25_B.1.617.2
MA−CDCBI−CRSP_AJ2PK64EQINY2LGG_2021−10−29_B.1.617.2
OM002094_MA−CDCBI−CRSP_U46QX42GVJU367PE_2021−12−04_B.1.617.2
22−002350−006_MA_Bristol_2021−12−02_B.1.617.2
MA−CDCBI−CRSP_AD2ETPSTV72ALAWL_2021−09−02_B.1.617.2
MA−CDCBI−CRSP_76HJQZVDUCZIWCAG_2021−08−24_B.1.617.2
MA−CDCBI−CRSP_6L2TXDXM3XSNLR2A_2021−09−23_B.1.617.2
MA−CDCBI−CRSP_2ITKAPPUO4VNWL74_2021−09−20_B.1.617.2
MA−CDCBI−CRSP_JNWTPSAXPSCOY2AI_2021−09−17_B.1.617.2
MA−CDC−ASC210391254_2021−11−05_B.1.617.2
MA−MASPHL−05143_2021−08−16_B.1.617.2
MA−CDC−STM−JUSPUX2DZ_2021−11−01_B.1.617.2
MA−CDC−LC0228415_2021−08−19_B.1.617.2
MA−CDCBI−CRSP_FQ4BYAXJAARXLLDW_2021−10−10_B.1.617.2
MN908947 (Wuhan−Hu−1/2019)
0.2

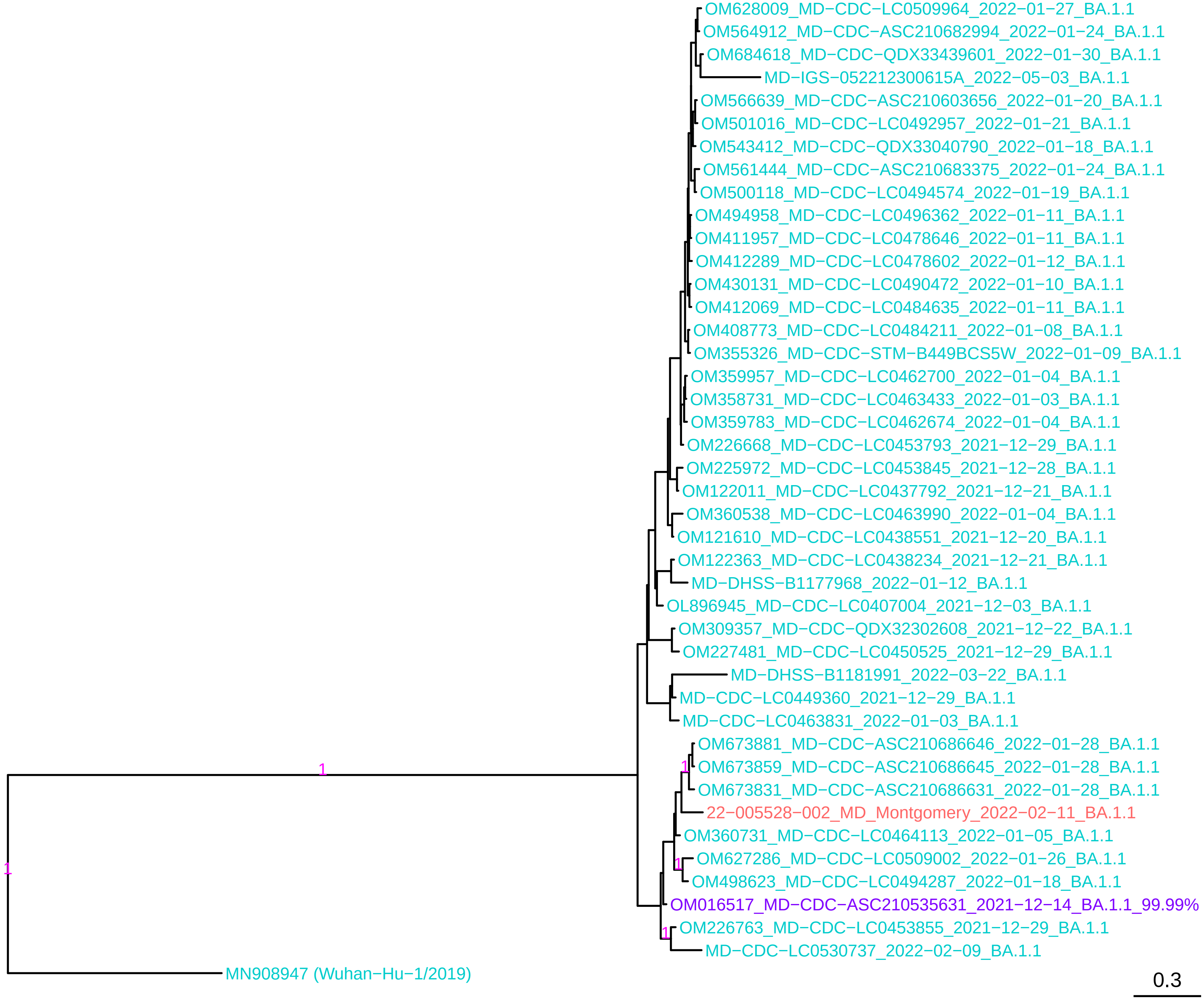
OM628009_MD−CDC−LC0509964_2022−01−27_BA.1.1
OM564912_MD−CDC−ASC210682994_2022−01−24_BA.1.1
OM684618_MD−CDC−QDX33439601_2022−01−30_BA.1.1
MD−IGS−052212300615A_2022−05−03_BA.1.1
OM566639_MD−CDC−ASC210603656_2022−01−20_BA.1.1
OM501016_MD−CDC−LC0492957_2022−01−21_BA.1.1
OM543412_MD−CDC−QDX33040790_2022−01−18_BA.1.1
OM561444_MD−CDC−ASC210683375_2022−01−24_BA.1.1
OM500118_MD−CDC−LC0494574_2022−01−19_BA.1.1
OM494958_MD−CDC−LC0496362_2022−01−11_BA.1.1
OM411957_MD−CDC−LC0478646_2022−01−11_BA.1.1
OM412289_MD−CDC−LC0478602_2022−01−12_BA.1.1
OM430131_MD−CDC−LC0490472_2022−01−10_BA.1.1
OM412069_MD−CDC−LC0484635_2022−01−11_BA.1.1
OM408773_MD−CDC−LC0484211_2022−01−08_BA.1.1
OM355326_MD−CDC−STM−B449BCS5W_2022−01−09_BA.1.1
OM359957_MD−CDC−LC0462700_2022−01−04_BA.1.1
OM358731_MD−CDC−LC0463433_2022−01−03_BA.1.1
OM359783_MD−CDC−LC0462674_2022−01−04_BA.1.1
OM226668_MD−CDC−LC0453793_2021−12−29_BA.1.1
OM225972_MD−CDC−LC0453845_2021−12−28_BA.1.1
OM122011_MD−CDC−LC0437792_2021−12−21_BA.1.1
OM360538_MD−CDC−LC0463990_2022−01−04_BA.1.1
OM121610_MD−CDC−LC0438551_2021−12−20_BA.1.1
OM122363_MD−CDC−LC0438234_2021−12−21_BA.1.1
MD−DHSS−B1177968_2022−01−12_BA.1.1
OL896945_MD−CDC−LC0407004_2021−12−03_BA.1.1
OM309357_MD−CDC−QDX32302608_2021−12−22_BA.1.1
OM227481_MD−CDC−LC0450525_2021−12−29_BA.1.1
MD−DHSS−B1181991_2022−03−22_BA.1.1
MD−CDC−LC0449360_2021−12−29_BA.1.1
MD−CDC−LC0463831_2022−01−03_BA.1.1
OM673881_MD−CDC−ASC210686646_2022−01−28_BA.1.1
1
OM673859_MD−CDC−ASC210686645_2022−01−28_BA.1.1
OM673831_MD−CDC−ASC210686631_2022−01−28_BA.1.1
22−005528−002_MD_Montgomery_2022−02−11_BA.1.1
OM360731_MD−CDC−LC0464113_2022−01−05_BA.1.1
OM627286_MD−CDC−LC0509002_2022−01−26_BA.1.1
1
OM498623_MD−CDC−LC0494287_2022−01−18_BA.1.1
OM016517_MD−CDC−ASC210535631_2021−12−14_BA.1.1_99.99%
OM226763_MD−CDC−LC0453855_2021−12−29_BA.1.1
1
MD−CDC−LC0530737_2022−02−09_BA.1.1
1
1
MN908947 (Wuhan−Hu−1/2019)
0.3

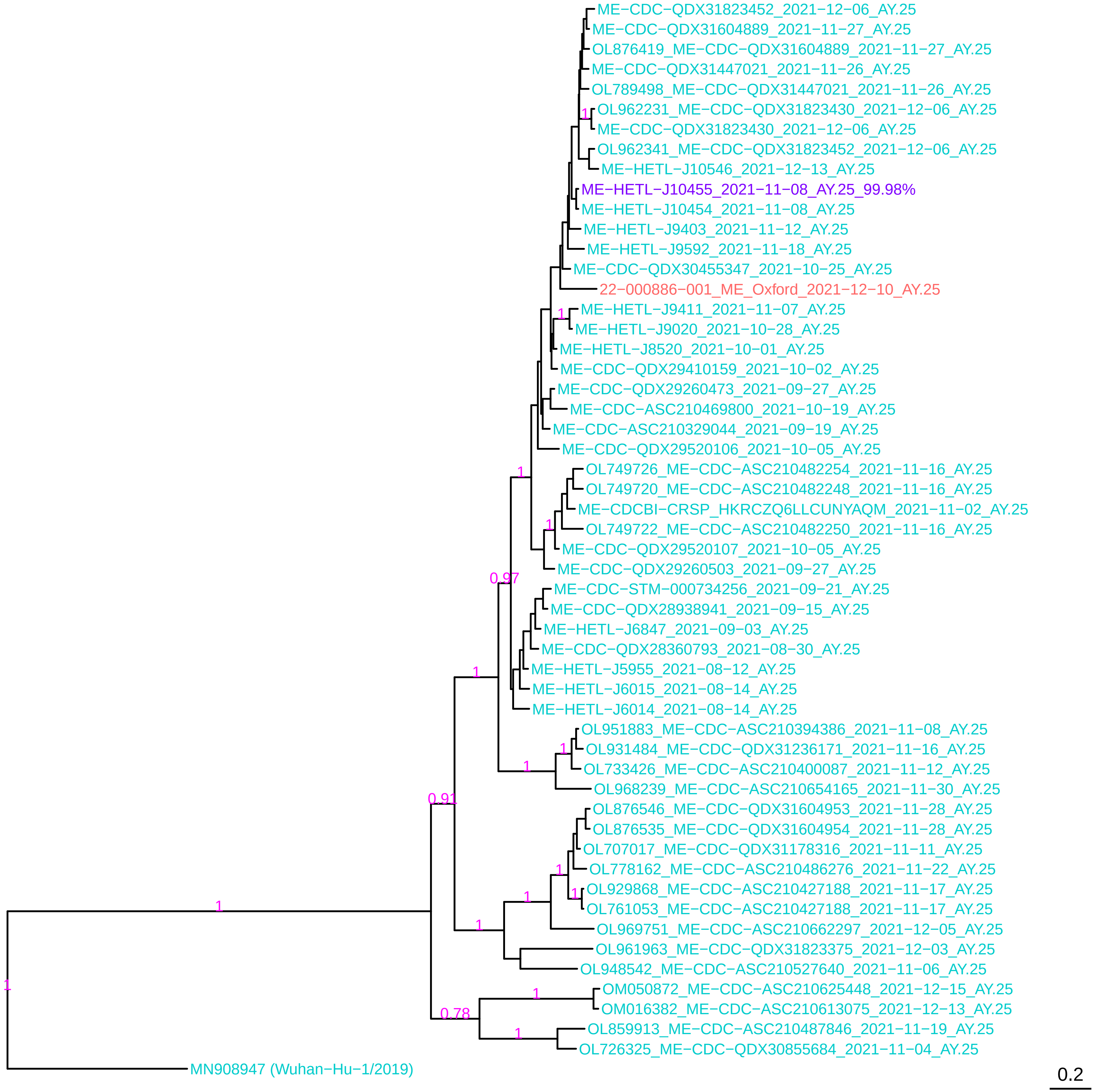
ME−CDC−QDX31823452_2021−12−06_AY.25
ME−CDC−QDX31604889_2021−11−27_AY.25
OL876419_ME−CDC−QDX31604889_2021−11−27_AY.25
ME−CDC−QDX31447021_2021−11−26_AY.25
OL789498_ME−CDC−QDX31447021_2021−11−26_AY.25
OL962231_ME−CDC−QDX31823430_2021−12−06_AY.25
ME−CDC−QDX31823430_2021−12−06_AY.25
OL962341_ME−CDC−QDX31823452_2021−12−06_AY.25
ME−HETL−J10546_2021−12−13_AY.25
ME−HETL−J10455_2021−11−08_AY.25_99.98%
ME−HETL−J10454_2021−11−08_AY.25
ME−HETL−J9403_2021−11−12_AY.25
ME−HETL−J9592_2021−11−18_AY.25
ME−CDC−QDX30455347_2021−10−25_AY.25
22−000886−001_ME_Oxford_2021−12−10_AY.25
ME−HETL−J9411_2021−11−07_AY.25
ME−HETL−J9020_2021−10−28_AY.25
ME−HETL−J8520_2021−10−01_AY.25
ME−CDC−QDX29410159_2021−10−02_AY.25
ME−CDC−QDX29260473_2021−09−27_AY.25
ME−CDC−ASC210469800_2021−10−19_AY.25
ME−CDC−ASC210329044_2021−09−19_AY.25
ME−CDC−QDX29520106_2021−10−05_AY.25
OL749726_ME−CDC−ASC210482254_2021−11−16_AY.25
OL749720_ME−CDC−ASC210482248_2021−11−16_AY.25
ME−CDCBI−CRSP_HKRCZQ6LLCUNYAQM_2021−11−02_AY.25
OL749722_ME−CDC−ASC210482250_2021−11−16_AY.25
ME−CDC−QDX29520107_2021−10−05_AY.25
ME−CDC−QDX29260503_2021−09−27_AY.25
ME−CDC−STM−000734256_2021−09−21_AY.25
ME−CDC−QDX28938941_2021−09−15_AY.25
ME−HETL−J6847_2021−09−03_AY.25
ME−CDC−QDX28360793_2021−08−30_AY.25
ME−HETL−J5955_2021−08−12_AY.25
ME−HETL−J6015_2021−08−14_AY.25
ME−HETL−J6014_2021−08−14_AY.25
OL951883_ME−CDC−ASC210394386_2021−11−08_AY.25
OL931484_ME−CDC−QDX31236171_2021−11−16_AY.25
OL733426_ME−CDC−ASC210400087_2021−11−12_AY.25
OL968239_ME−CDC−ASC210654165_2021−11−30_AY.25
OL876546_ME−CDC−QDX31604953_2021−11−28_AY.25
OL876535_ME−CDC−QDX31604954_2021−11−28_AY.25
OL707017_ME−CDC−QDX31178316_2021−11−11_AY.25
OL778162_ME−CDC−ASC210486276_2021−11−22_AY.25
OL929868_ME−CDC−ASC210427188_2021−11−17_AY.25
OL761053_ME−CDC−ASC210427188_2021−11−17_AY.25
OL969751_ME−CDC−ASC210662297_2021−12−05_AY.25
OL961963_ME−CDC−QDX31823375_2021−12−03_AY.25
OL948542_ME−CDC−ASC210527640_2021−11−06_AY.25
OM050872_ME−CDC−ASC210625448_2021−12−15_AY.25
OM016382_ME−CDC−ASC210613075_2021−12−13_AY.25
OL859913_ME−CDC−ASC210487846_2021−11−19_AY.25
OL726325_ME−CDC−QDX30855684_2021−11−04_AY.25
MN908947 (Wuhan−Hu−1/2019)
1
1
0.97
1
1
1
0.91
1
1
1
1
1
1
1
0.78
1
1
0.2

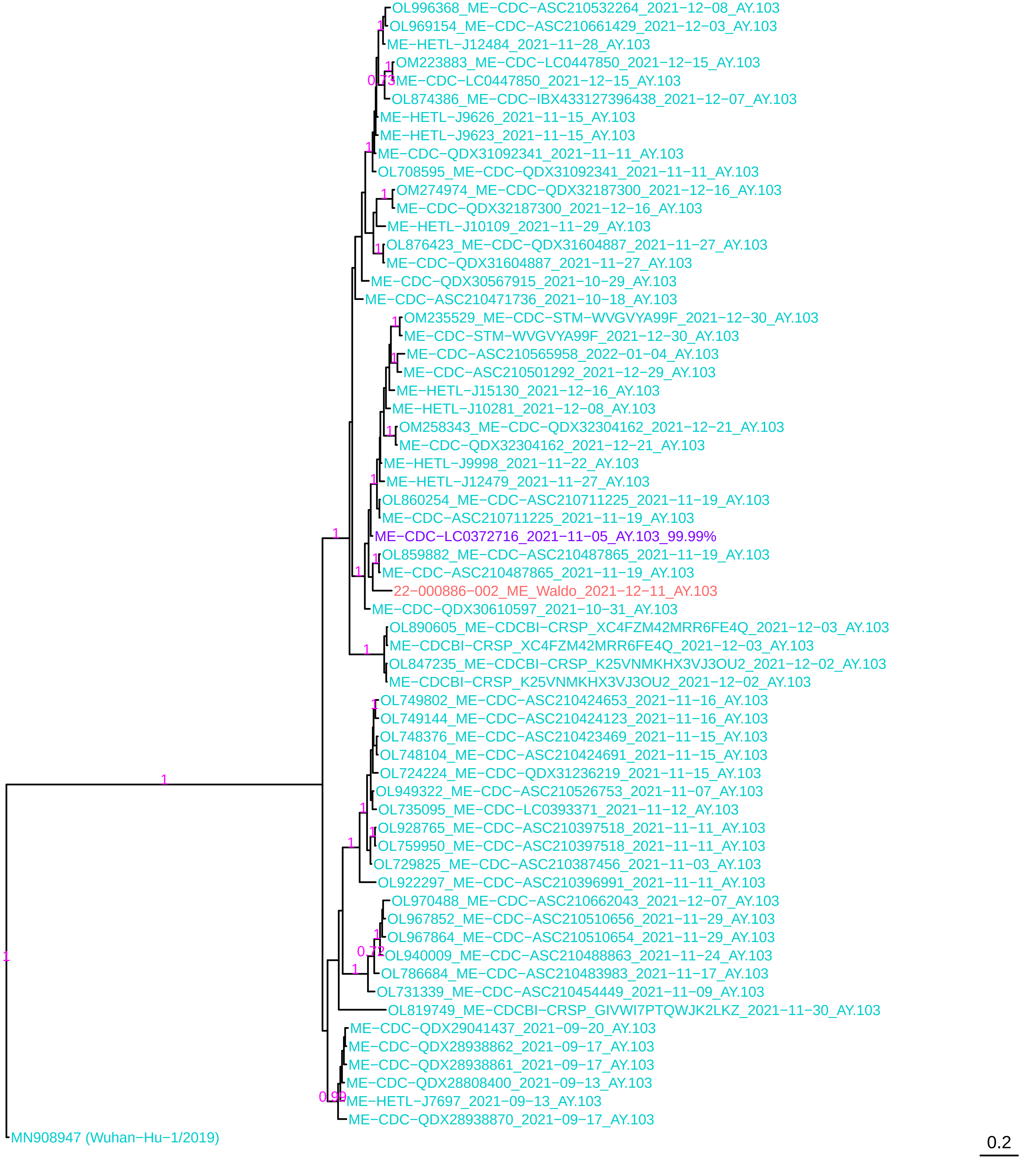
OL996368_ME−CDC−ASC210532264_2021−12−08_AY.103
OL969154_ME−CDC−ASC210661429_2021−12−03_AY.103
ME−HETL−J12484_2021−11−28_AY.103
OM223883_ME−CDC−LC0447850_2021−12−15_AY.103
ME−CDC−LC0447850_2021−12−15_AY.103
OL874386_ME−CDC−IBX433127396438_2021−12−07_AY.103
ME−HETL−J9626_2021−11−15_AY.103
ME−HETL−J9623_2021−11−15_AY.103
ME−CDC−QDX31092341_2021−11−11_AY.103
OL708595_ME−CDC−QDX31092341_2021−11−11_AY.103
OM274974_ME−CDC−QDX32187300_2021−12−16_AY.103
ME−CDC−QDX32187300_2021−12−16_AY.103
ME−HETL−J10109_2021−11−29_AY.103
OL876423_ME−CDC−QDX31604887_2021−11−27_AY.103
ME−CDC−QDX31604887_2021−11−27_AY.103
ME−CDC−QDX30567915_2021−10−29_AY.103
ME−CDC−ASC210471736_2021−10−18_AY.103
OM235529_ME−CDC−STM−WVGVYA99F_2021−12−30_AY.103
ME−CDC−STM−WVGVYA99F_2021−12−30_AY.103
ME−CDC−ASC210565958_2022−01−04_AY.103
ME−CDC−ASC210501292_2021−12−29_AY.103
ME−HETL−J15130_2021−12−16_AY.103
ME−HETL−J10281_2021−12−08_AY.103
OM258343_ME−CDC−QDX32304162_2021−12−21_AY.103
ME−CDC−QDX32304162_2021−12−21_AY.103
ME−HETL−J9998_2021−11−22_AY.103
ME−HETL−J12479_2021−11−27_AY.103
OL860254_ME−CDC−ASC210711225_2021−11−19_AY.103
ME−CDC−ASC210711225_2021−11−19_AY.103
ME−CDC−LC0372716_2021−11−05_AY.103_99.99%
OL859882_ME−CDC−ASC210487865_2021−11−19_AY.103
ME−CDC−ASC210487865_2021−11−19_AY.103
22−000886−002_ME_Waldo_2021−12−11_AY.103
ME−CDC−QDX30610597_2021−10−31_AY.103
OL890605_ME−CDCBI−CRSP_XC4FZM42MRR6FE4Q_2021−12−03_AY.103
ME−CDCBI−CRSP_XC4FZM42MRR6FE4Q_2021−12−03_AY.103
OL847235_ME−CDCBI−CRSP_K25VNMKHX3VJ3OU2_2021−12−02_AY.103
ME−CDCBI−CRSP_K25VNMKHX3VJ3OU2_2021−12−02_AY.103
OL749802_ME−CDC−ASC210424653_2021−11−16_AY.103
OL749144_ME−CDC−ASC210424123_2021−11−16_AY.103
OL748376_ME−CDC−ASC210423469_2021−11−15_AY.103
OL748104_ME−CDC−ASC210424691_2021−11−15_AY.103
OL724224_ME−CDC−QDX31236219_2021−11−15_AY.103
OL949322_ME−CDC−ASC210526753_2021−11−07_AY.103
OL735095_ME−CDC−LC0393371_2021−11−12_AY.103
OL928765_ME−CDC−ASC210397518_2021−11−11_AY.103
OL759950_ME−CDC−ASC210397518_2021−11−11_AY.103
OL729825_ME−CDC−ASC210387456_2021−11−03_AY.103
OL922297_ME−CDC−ASC210396991_2021−11−11_AY.103
OL970488_ME−CDC−ASC210662043_2021−12−07_AY.103
OL967852_ME−CDC−ASC210510656_2021−11−29_AY.103
OL967864_ME−CDC−ASC210510654_2021−11−29_AY.103
OL940009_ME−CDC−ASC210488863_2021−11−24_AY.103
OL786684_ME−CDC−ASC210483983_2021−11−17_AY.103
OL731339_ME−CDC−ASC210454449_2021−11−09_AY.103
OL819749_ME−CDCBI−CRSP_GIVWI7PTQWJK2LKZ_2021−11−30_AY.103
ME−CDC−QDX29041437_2021−09−20_AY.103
ME−CDC−QDX28938862_2021−09−17_AY.103
ME−CDC−QDX28938861_2021−09−17_AY.103
ME−CDC−QDX28808400_2021−09−13_AY.103
ME−HETL−J7697_2021−09−13_AY.103
ME−CDC−QDX28938870_2021−09−17_AY.103
MN908947 (Wuhan−Hu−1/2019)
0.2

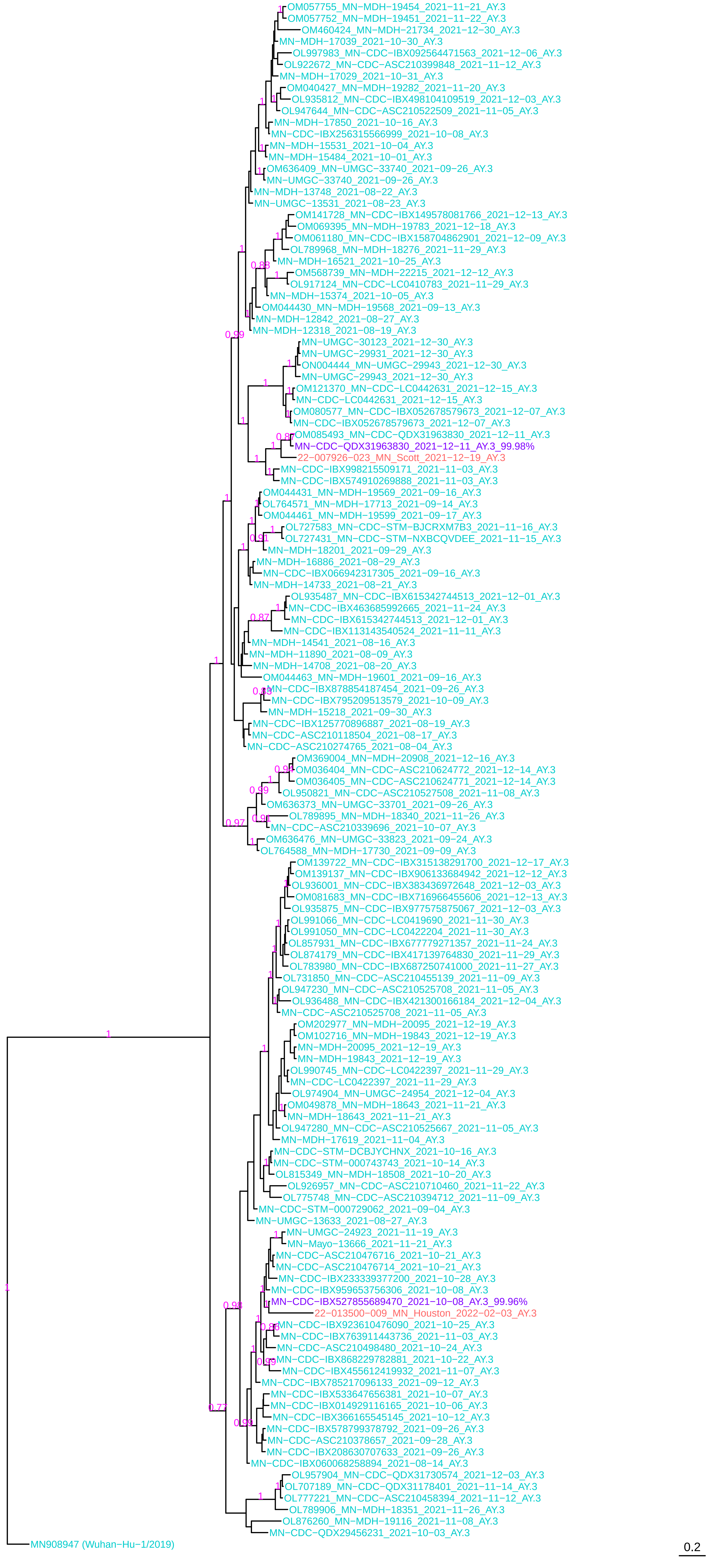

OM057755_MN-MDH-19454_2021-11-21_AY.3
OM057752_MN-MDH-19451_2021-11-22_AY.3
OM460424_MN-MDH-21734_2021-12-30_AY.3
MN-MDH-17039_2021-10-30_AY.3
OL997983_MN-CDC-IBX092564471563_2021-12-06_AY.3
OL922672_MN-CDC-ASC210399848_2021-11-12_AY.3
MN-MDH-17029_2021-10-31_AY.3
OM040427_MN-MDH-19282_2021-11-20_AY.3
OL935812_MN-CDC-IBX498104109519_2021-12-03_AY.3
OL947644_MN-CDC-ASC210522509_2021-11-05_AY.3
MN-MDH-17850_2021-10-16_AY.3
MN-CDC-IBX256315566999_2021-10-08_AY.3
MN-MDH-15531_2021-10-04_AY.3
MN-MDH-15484_2021-10-01_AY.3
OM636409_MN-UMGC-33740_2021-09-26_AY.3
MN-UMGC-33740_2021-09-26_AY.3
MN-MDH-13748_2021-08-22_AY.3
MN-UMGC-13531_2021-08-23_AY.3
OM141728_MN-CDC-IBX149578081766_2021-12-13_AY.3
OM069395_MN-MDH-19783_2021-12-18_AY.3
OM061180_MN-CDC-IBX158704862901_2021-12-09_AY.3
OL789968_MN-MDH-18276_2021-11-29_AY.3
MN-MDH-16521_2021-10-25_AY.3
OM568739_MN-MDH-22215_2021-12-12_AY.3
OL917124_MN-CDC-LC0410783_2021-11-29_AY.3
MN-MDH-15374_2021-10-05_AY.3
OM044430_MN-MDH-19568_2021-09-13_AY.3
MN-MDH-12842_2021-08-27_AY.3
MN-MDH-12318_2021-08-19_AY.3
MN-UMGC-30123_2021-12-30_AY.3
MN-UMGC-29931_2021-12-30_AY.3
ON004444_MN-UMGC-29943_2021-12-30_AY.3
MN-UMGC-29943_2021-12-30_AY.3
OM121370_MN-CDC-LC0442631_2021-12-15_AY.3
MN-CDC-LC0442631_2021-12-15_AY.3
OM080577_MN-CDC-IBX052678579673_2021-12-07_AY.3
MN-CDC-IBX052678579673_2021-12-07_AY.3
OM085493_MN-CDC-QDX31963830_2021-12-11_AY.3
MN-CDC-QDX31963830_2021-12-11_AY.3_99.98%
22-007926-023_MN_Scott_2021-12-19_AY.3
MN-CDC-IBX998215509171_2021-11-03_AY.3
MN-CDC-IBX574910269888_2021-11-03_AY.3
OM044431_MN-MDH-19569_2021-09-16_AY.3
OL764571_MN-MDH-17713_2021-09-14_AY.3
OM044461_MN-MDH-19599_2021-09-17_AY.3
OL727583_MN-CDC-STM-BJCRXM7B3_2021-11-16_AY.3
OL727431_MN-CDC-STM-NXBCQVDEE_2021-11-15_AY.3
MN-MDH-18201_2021-09-29_AY.3
MN-MDH-16886_2021-08-29_AY.3
MN-CDC-IBX066942317305_2021-09-16_AY.3
MN-MDH-14733_2021-08-21_AY.3
OL935487_MN-CDC-IBX615342744513_2021-12-01_AY.3
MN-CDC-IBX463685992665_2021-11-24_AY.3
MN-CDC-IBX615342744513_2021-12-01_AY.3
MN-CDC-IBX113143540524_2021-11-11_AY.3
MN-MDH-14541_2021-08-16_AY.3
MN-MDH-11890_2021-08-09_AY.3
MN-MDH-14708_2021-08-20_AY.3
OM044463_MN-MDH-19601_2021-09-16_AY.3
MN-CDC-IBX878854187454_2021-09-26_AY.3
MN-CDC-IBX795209513579_2021-10-09_AY.3
MN-MDH-15218_2021-09-30_AY.3
MN-CDC-IBX125770896887_2021-08-19_AY.3
MN-CDC-ASC210118504_2021-08-17_AY.3
MN-CDC-ASC210274765_2021-08-04_AY.3
OM369004_MN-MDH-20908_2021-12-16_AY.3
OM036404_MN-CDC-ASC210624772_2021-12-14_AY.3
OM036405_MN-CDC-ASC210624771_2021-12-14_AY.3
OL950821_MN-CDC-ASC210527508_2021-11-08_AY.3
OM636373_MN-UMGC-33701_2021-09-26_AY.3
OL789895_MN-MDH-18340_2021-11-26_AY.3
MN-CDC-ASC210339696_2021-10-07_AY.3
OM636476_MN-UMGC-33823_2021-09-24_AY.3
OL764588_MN-MDH-17730_2021-09-09_AY.3
OM139722_MN-CDC-IBX315138291700_2021-12-17_AY.3
OM139137_MN-CDC-IBX906133684942_2021-12-12_AY.3
OL936001_MN-CDC-IBX383436972648_2021-12-03_AY.3
OM081683_MN-CDC-IBX716966455606_2021-12-13_AY.3
OL935875_MN-CDC-IBX977575875067_2021-12-03_AY.3
OL991066_MN-CDC-LC0419690_2021-11-30_AY.3
OL991050_MN-CDC-LC0422204_2021-11-30_AY.3
OL857931_MN-CDC-IBX677779271357_2021-11-24_AY.3
OL874179_MN-CDC-IBX417139764830_2021-11-29_AY.3
OL783980_MN-CDC-IBX687250741000_2021-11-27_AY.3
OL731850_MN-CDC-ASC210455139_2021-11-09_AY.3
OL947230_MN-CDC-ASC210525708_2021-11-05_AY.3
OL936488_MN-CDC-IBX421300166184_2021-12-04_AY.3
MN-CDC-ASC210525708_2021-11-05_AY.3
OM202977_MN-MDH-20095_2021-12-19_AY.3
OM102716_MN-MDH-19843_2021-12-19_AY.3
MN-MDH-20095_2021-12-19_AY.3
MN-MDH-19843_2021-12-19_AY.3
OL990745_MN-CDC-LC0422397_2021-11-29_AY.3
MN-CDC-LC0422397_2021-11-29_AY.3
OL974904_MN-UMGC-24954_2021-12-04_AY.3
OM049878_MN-MDH-18643_2021-11-21_AY.3
MN-MDH-18643_2021-11-21_AY.3
OL947280_MN-CDC-ASC210525667_2021-11-05_AY.3
MN-MDH-17619_2021-11-04_AY.3
MN-CDC-STM-DCBJYCHNX_2021-10-16_AY.3
MN-CDC-STM-000743743_2021-10-14_AY.3
OL815349_MN-MDH-18508_2021-10-20_AY.3
OL926957_MN-CDC-ASC210710460_2021-11-22_AY.3
OL775748_MN-CDC-ASC210394712_2021-11-09_AY.3
MN-CDC-STM-000729062_2021-09-04_AY.3
MN-UMGC-13633_2021-08-27_AY.3
MN-UMGC-24923_2021-11-19_AY.3
MN-Mayo-13666_2021-11-21_AY.3
MN-CDC-ASC210476716_2021-10-21_AY.3
MN-CDC-ASC210476714_2021-10-21_AY.3
MN-CDC-IBX233339377200_2021-10-28_AY.3
MN-CDC-IBX959653756306_2021-10-08_AY.3
MN-CDC-IBX527855689470_2021-10-08_AY.3_99.96%
22-013500-009_MN_Houston_2022-02-03_AY.3
MN-CDC-IBX923610476090_2021-10-25_AY.3
MN-CDC-IBX763911443736_2021-11-03_AY.3
MN-CDC-ASC210498480_2021-10-24_AY.3
MN-CDC-IBX868229782881_2021-10-22_AY.3
MN-CDC-IBX455612419932_2021-11-07_AY.3
MN-CDC-IBX785217096133_2021-09-12_AY.3
MN-CDC-IBX533647656381_2021-10-07_AY.3
MN-CDC-IBX014929116165_2021-10-06_AY.3
MN-CDC-IBX366165545145_2021-10-12_AY.3
MN-CDC-IBX578799378792_2021-09-26_AY.3
MN-CDC-ASC210378657_2021-09-28_AY.3
MN-CDC-IBX208630707633_2021-09-26_AY.3
MN-CDC-IBX060068258894_2021-08-14_AY.3
OL957904_MN-CDC-QDX31730574_2021-12-03_AY.3
OL707189_MN-CDC-QDX31178401_2021-11-14_AY.3
OL777221_MN-CDC-ASC210458394_2021-11-12_AY.3
OL789906_MN-MDH-18351_2021-11-26_AY.3
OL876260_MN-MDH-19116_2021-11-08_AY.3
MN-CDC-QDX29456231_2021-10-03_AY.3
MN908947 (Wuhan-Hu-1/2019)
0.2

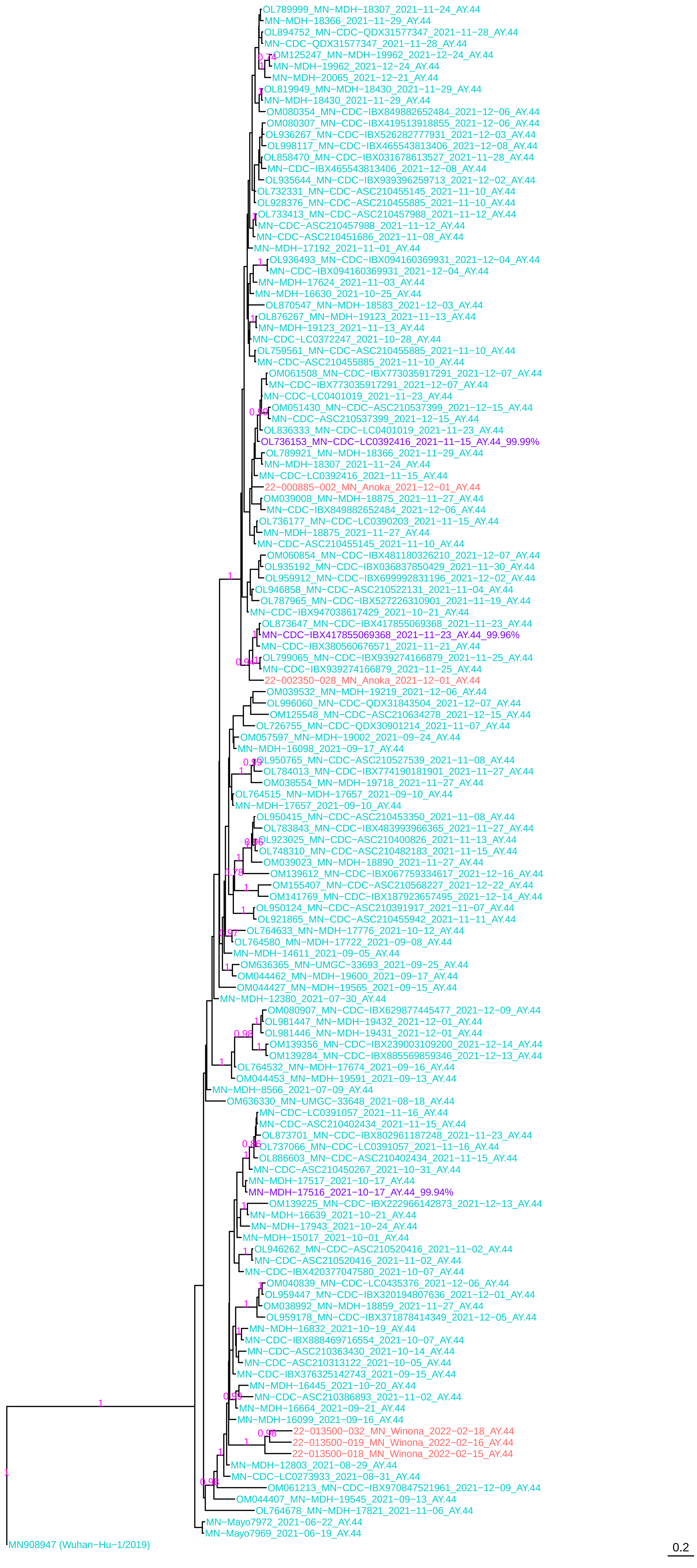

OL789999_MN-MDH-18307_2021-11-24_AY.44
MN-MDH-18366_2021-11-29_AY.44
OL894752_MN-CDC-QDX31577347_2021-11-28_AY.44
MN-CDC-QDX31577347_2021-11-28_AY.44
OM125247_MN-MDH-19962_2021-12-24_AY.44
MN-MDH-19962_2021-12-24_AY.44
MN-MDH-20065_2021-12-21_AY.44
OL819949_MN-MDH-18430_2021-11-29_AY.44
MN-MDH-18430_2021-11-29_AY.44
OM080354_MN-CDC-IBX849882652484_2021-12-06_AY.44
OM080307_MN-CDC-IBX419513918855_2021-12-06_AY.44
OL936267_MN-CDC-IBX526282777931_2021-12-03_AY.44
OL998117_MN-CDC-IBX465543813406_2021-12-08_AY.44
OL858470_MN-CDC-IBX031678613527_2021-11-28_AY.44
MN-CDC-IBX465543813406_2021-12-08_AY.44
OL935644_MN-CDC-IBX939396259713_2021-12-02_AY.44
OL732331_MN-CDC-ASC210455145_2021-11-10_AY.44
OL928376_MN-CDC-ASC210455885_2021-11-10_AY.44
OL733413_MN-CDC-ASC210457988_2021-11-12_AY.44
MN-CDC-ASC210457988_2021-11-12_AY.44
MN-CDC-ASC210451686_2021-11-08_AY.44
MN-MDH-17192_2021-11-01_AY.44
OL936493_MN-CDC-IBX094160369931_2021-12-04_AY.44
MN-CDC-IBX094160369931_2021-12-04_AY.44
MN-MDH-17624_2021-11-03_AY.44
MN-MDH-16630_2021-10-25_AY.44
OL870547_MN-MDH-18583_2021-12-03_AY.44
OL876267_MN-MDH-19123_2021-11-13_AY.44
MN-MDH-19123_2021-11-13_AY.44
MN-CDC-LC0372247_2021-10-28_AY.44
OL759561_MN-CDC-ASC210455885_2021-11-10_AY.44
MN-CDC-ASC210455885_2021-11-10_AY.44
OM061508_MN-CDC-IBX773035917291_2021-12-07_AY.44
MN-CDC-IBX773035917291_2021-12-07_AY.44
MN-CDC-LC0401019_2021-11-23_AY.44
OM051430_MN-CDC-ASC210537399_2021-12-15_AY.44
MN-CDC-ASC210537399_2021-12-15_AY.44
OL836333_MN-CDC-LC0401019_2021-11-23_AY.44
OL736153_MN-CDC-LC0392416_2021-11-15_AY.44_99.99%
OL789921_MN-MDH-18366_2021-11-29_AY.44
MN-MDH-18307_2021-11-24_AY.44
MN-CDC-LC0392416_2021-11-15_AY.44
22-000885-002_MN_Anoka_2021-12-01_AY.44
OM039008_MN-MDH-18875_2021-11-27_AY.44
MN-CDC-IBX849882652484_2021-12-06_AY.44
OL736177_MN-CDC-LC0390203_2021-11-15_AY.44
MN-MDH-18875_2021-11-27_AY.44
MN-CDC-ASC210455145_2021-11-10_AY.44
OM060854_MN-CDC-IBX481180326210_2021-12-07_AY.44
OL935192_MN-CDC-IBX036837850429_2021-11-30_AY.44
OL959912_MN-CDC-IBX699992831196_2021-12-02_AY.44
OL946858_MN-CDC-ASC210522131_2021-11-04_AY.44
OL787965_MN-CDC-IBX527226310901_2021-11-19_AY.44
MN-CDC-IBX947038617429_2021-10-21_AY.44
OL873647_MN-CDC-IBX417855069368_2021-11-23_AY.44
MN-CDC-IBX417855069368_2021-11-23_AY.44_99.96%
MN-CDC-IBX380560676571_2021-11-21_AY.44
OL799065_MN-CDC-IBX939274166879_2021-11-25_AY.44
MN-CDC-IBX939274166879_2021-11-25_AY.44
22-002350-028_MN_Anoka_2021-12-01_AY.44
OM039532_MN-MDH-19219_2021-12-06_AY.44
OL996060_MN-CDC-QDX31843504_2021-12-07_AY.44
OM125548_MN-CDC-ASC210634278_2021-12-15_AY.44
OL726755_MN-CDC-QDX30901214_2021-11-07_AY.44
OM057597_MN-MDH-19002_2021-09-24_AY.44
MN-MDH-16098_2021-09-17_AY.44
OL950765_MN-CDC-ASC210527539_2021-11-08_AY.44
OL784013_MN-CDC-IBX774190181901_2021-11-27_AY.44
OM038554_MN-MDH-19718_2021-11-27_AY.44
OL764515_MN-MDH-17657_2021-09-10_AY.44
MN-MDH-17657_2021-09-10_AY.44
OL950415_MN-CDC-ASC210453350_2021-11-08_AY.44
OL783843_MN-CDC-IBX483993966365_2021-11-27_AY.44
OL923025_MN-CDC-ASC210400826_2021-11-13_AY.44
OL748310_MN-CDC-ASC210482183_2021-11-15_AY.44
OM039023_MN-MDH-18890_2021-11-27_AY.44
OM139612_MN-CDC-IBX067759334617_2021-12-16_AY.44
OM155407_MN-CDC-ASC210568227_2021-12-22_AY.44
OM141769_MN-CDC-IBX187923657495_2021-12-14_AY.44
OL950124_MN-CDC-ASC210391917_2021-11-07_AY.44
OL921865_MN-CDC-ASC210455942_2021-11-11_AY.44
OL764633_MN-MDH-17776_2021-10-12_AY.44
OL764580_MN-MDH-17722_2021-09-08_AY.44
MN-MDH-14611_2021-09-05_AY.44
OM636365_MN-UMGC-33693_2021-09-25_AY.44
OM044462_MN-MDH-19600_2021-09-17_AY.44
OM044427_MN-MDH-19565_2021-09-15_AY.44
MN-MDH-12380_2021-07-30_AY.44
OM080907_MN-CDC-IBX629877445477_2021-12-09_AY.44
OL981447_MN-MDH-19432_2021-12-01_AY.44
OL981446_MN-MDH-19431_2021-12-01_AY.44
OM139356_MN-CDC-IBX239003109200_2021-12-14_AY.44
OM139284_MN-CDC-IBX885569859346_2021-12-13_AY.44
OL764532_MN-MDH-17674_2021-09-16_AY.44
OM044453_MN-MDH-19591_2021-09-13_AY.44
MN-MDH-8566_2021-07-09_AY.44
OM636330_MN-UMGC-33648_2021-08-18_AY.44
MN-CDC-LC0391057_2021-11-16_AY.44
MN-CDC-ASC210402434_2021-11-15_AY.44
OL873701_MN-CDC-IBX802961187248_2021-11-23_AY.44
OL737066_MN-CDC-LC0391057_2021-11-16_AY.44
OL886603_MN-CDC-ASC210402434_2021-11-15_AY.44
MN-CDC-ASC210450267_2021-10-31_AY.44
MN-MDH-17517_2021-10-17_AY.44
MN-MDH-17516_2021-10-17_AY.44_99.94%
OM139225_MN-CDC-IBX222966142873_2021-12-13_AY.44
MN-MDH-16639_2021-10-21_AY.44
MN-MDH-17943_2021-10-24_AY.44
MN-MDH-15017_2021-10-01_AY.44
OL946262_MN-CDC-ASC210520416_2021-11-02_AY.44
MN-CDC-ASC210520416_2021-11-02_AY.44
MN-CDC-IBX420377047580_2021-10-07_AY.44
OM040839_MN-CDC-LC0435376_2021-12-06_AY.44
OL959447_MN-CDC-IBX320194807636_2021-12-01_AY.44
OM038992_MN-MDH-18859_2021-11-27_AY.44
OL959178_MN-CDC-IBX371878414349_2021-12-05_AY.44
MN-MDH-16832_2021-10-19_AY.44
MN-CDC-IBX888469716554_2021-10-07_AY.44
MN-CDC-ASC210363430_2021-10-14_AY.44
MN-CDC-ASC210313122_2021-10-05_AY.44
MN-CDC-IBX376325142743_2021-09-15_AY.44
MN-MDH-16445_2021-10-20_AY.44
MN-CDC-ASC210386893_2021-11-02_AY.44
MN-MDH-16664_2021-09-21_AY.44
MN-MDH-16099_2021-09-16_AY.44
22-013500-032_MN_Winona_2022-02-18_AY.44
22-013500-019_MN_Winona_2022-02-16_AY.44
22-013500-018_MN_Winona_2022-02-15_AY.44
MN-MDH-12803_2021-08-29_AY.44
MN-CDC-LC0273933_2021-08-31_AY.44
OM061213_MN-CDC-IBX970847521961_2021-12-09_AY.44
OM044407_MN-MDH-19545_2021-09-13_AY.44
OL764678_MN-MDH-17821_2021-11-06_AY.44
MN-Mayo7972_2021-06-22_AY.44
MN-Mayo7969_2021-06-19_AY.44
MN908947 (Wuhan-Hu-1/2019)
0.2

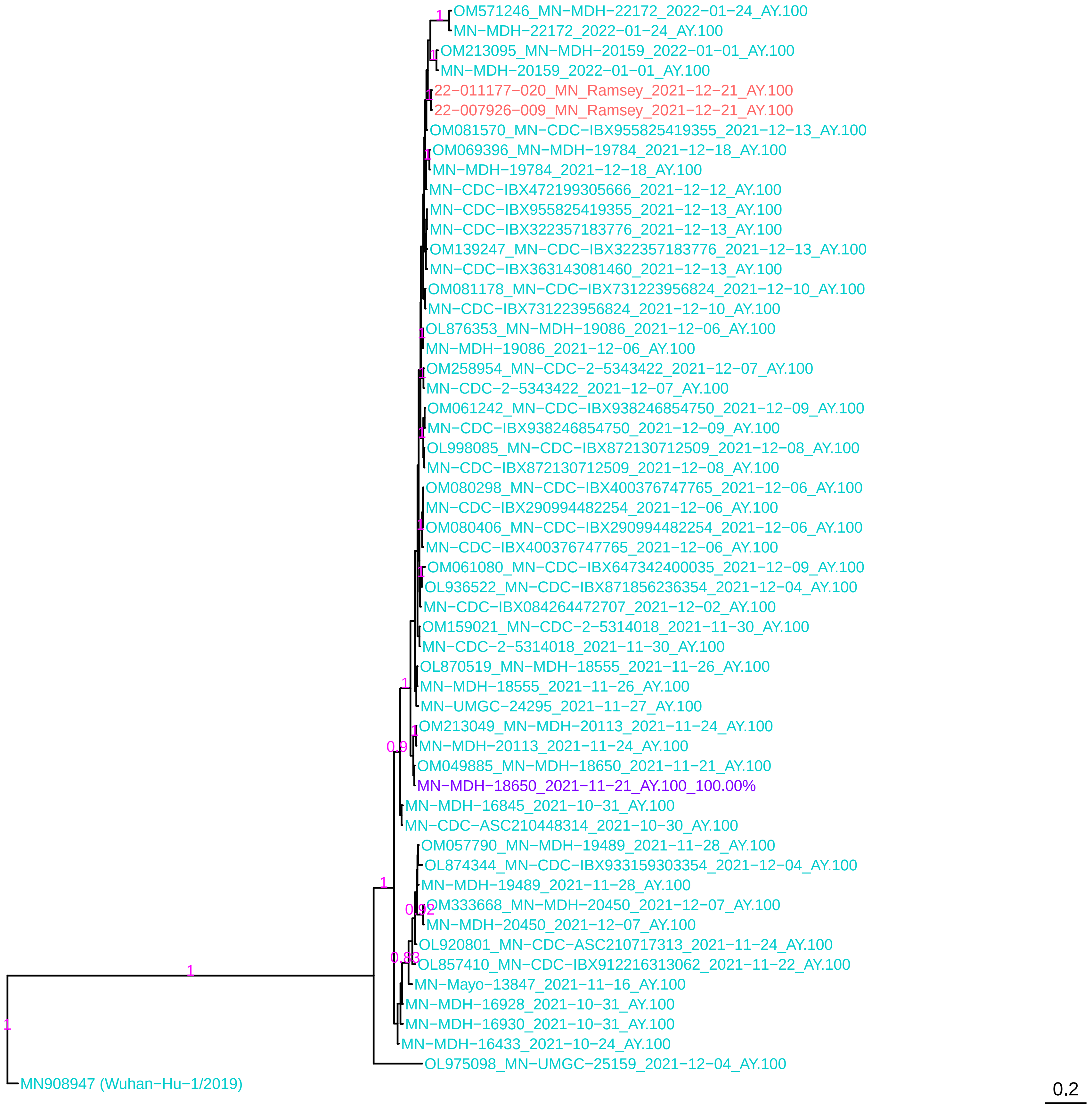
OM571246_MN−MDH−22172_2022−01−24_AY.100
MN−MDH−22172_2022−01−24_AY.100
OM213095_MN−MDH−20159_2022−01−01_AY.100
MN−MDH−20159_2022−01−01_AY.100
22−011177−020_MN_Ramsey_2021−12−21_AY.100
22−007926−009_MN_Ramsey_2021−12−21_AY.100
OM081570_MN−CDC−IBX955825419355_2021−12−13_AY.100
OM069396_MN−MDH−19784_2021−12−18_AY.100
MN−MDH−19784_2021−12−18_AY.100
MN−CDC−IBX472199305666_2021−12−12_AY.100
MN−CDC−IBX955825419355_2021−12−13_AY.100
MN−CDC−IBX322357183776_2021−12−13_AY.100
OM139247_MN−CDC−IBX322357183776_2021−12−13_AY.100
MN−CDC−IBX363143081460_2021−12−13_AY.100
OM081178_MN−CDC−IBX731223956824_2021−12−10_AY.100
MN−CDC−IBX731223956824_2021−12−10_AY.100
OL876353_MN−MDH−19086_2021−12−06_AY.100
MN−MDH−19086_2021−12−06_AY.100
OM258954_MN−CDC−2−5343422_2021−12−07_AY.100
MN−CDC−2−5343422_2021−12−07_AY.100
OM061242_MN−CDC−IBX938246854750_2021−12−09_AY.100
MN−CDC−IBX938246854750_2021−12−09_AY.100
OL998085_MN−CDC−IBX872130712509_2021−12−08_AY.100
MN−CDC−IBX872130712509_2021−12−08_AY.100
OM080298_MN−CDC−IBX400376747765_2021−12−06_AY.100
MN−CDC−IBX290994482254_2021−12−06_AY.100
OM080406_MN−CDC−IBX290994482254_2021−12−06_AY.100
MN−CDC−IBX400376747765_2021−12−06_AY.100
OM061080_MN−CDC−IBX647342400035_2021−12−09_AY.100
OL936522_MN−CDC−IBX871856236354_2021−12−04_AY.100
MN−CDC−IBX084264472707_2021−12−02_AY.100
OM159021_MN−CDC−2−5314018_2021−11−30_AY.100
MN−CDC−2−5314018_2021−11−30_AY.100
OL870519_MN−MDH−18555_2021−11−26_AY.100
MN−MDH−18555_2021−11−26_AY.100
MN−UMGC−24295_2021−11−27_AY.100
OM213049_MN−MDH−20113_2021−11−24_AY.100
MN−MDH−20113_2021−11−24_AY.100
OM049885_MN−MDH−18650_2021−11−21_AY.100
MN−MDH−18650_2021−11−21_AY.100_100.00%
MN−MDH−16845_2021−10−31_AY.100
MN−CDC−ASC210448314_2021−10−30_AY.100
OM057790_MN−MDH−19489_2021−11−28_AY.100
OL874344_MN−CDC−IBX933159303354_2021−12−04_AY.100
MN−MDH−19489_2021−11−28_AY.100
OM333668_MN−MDH−20450_2021−12−07_AY.100
MN−MDH−20450_2021−12−07_AY.100
OL920801_MN−CDC−ASC210717313_2021−11−24_AY.100
OL857410_MN−CDC−IBX912216313062_2021−11−22_AY.100
MN−Mayo−13847_2021−11−16_AY.100
MN−MDH−16928_2021−10−31_AY.100
MN−MDH−16930_2021−10−31_AY.100
MN−MDH−16433_2021−10−24_AY.100
OL975098_MN−UMGC−25159_2021−12−04_AY.100
MN908947 (Wuhan−Hu−1/2019)
0.9
0.92
0.83
0.2

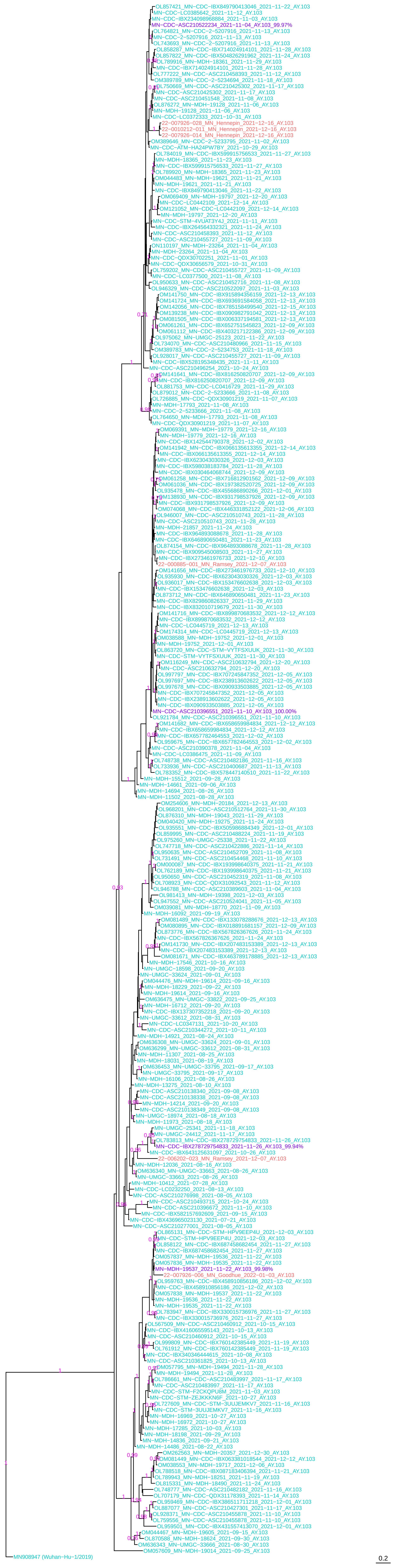
MN908947 (Wuhan-Hu-1/2019)
0.2

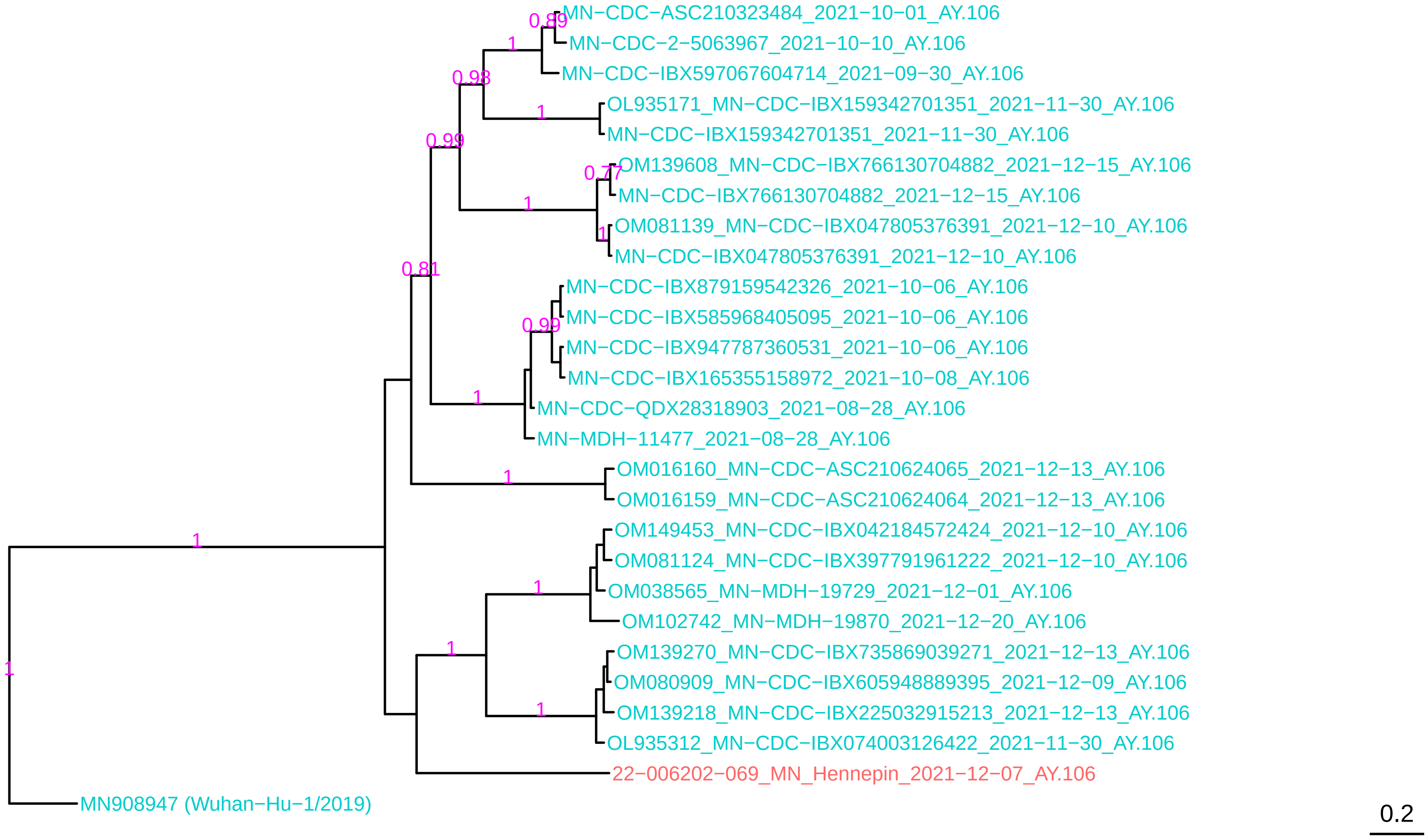
MN−CDC−ASC210323484_2021−10−01_AY.106
MN−CDC−2−5063967_2021−10−10_AY.106
MN−CDC−IBX597067604714_2021−09−30_AY.106
OL935171_MN−CDC−IBX159342701351_2021−11−30_AY.106
MN−CDC−IBX159342701351_2021−11−30_AY.106
OM139608_MN−CDC−IBX766130704882_2021−12−15_AY.106
MN−CDC−IBX766130704882_2021−12−15_AY.106
OM081139_MN−CDC−IBX047805376391_2021−12−10_AY.106
MN−CDC−IBX047805376391_2021−12−10_AY.106
MN−CDC−IBX879159542326_2021−10−06_AY.106
MN−CDC−IBX585968405095_2021−10−06_AY.106
MN−CDC−IBX947787360531_2021−10−06_AY.106
MN−CDC−IBX165355158972_2021−10−08_AY.106
MN−CDC−QDX28318903_2021−08−28_AY.106
MN−MDH−11477_2021−08−28_AY.106
OM016160_MN−CDC−ASC210624065_2021−12−13_AY.106
OM016159_MN−CDC−ASC210624064_2021−12−13_AY.106
OM149453_MN−CDC−IBX042184572424_2021−12−10_AY.106
OM081124_MN−CDC−IBX397791961222_2021−12−10_AY.106
OM038565_MN−MDH−19729_2021−12−01_AY.106
OM102742_MN−MDH−19870_2021−12−20_AY.106
OM139270_MN−CDC−IBX735869039271_2021−12−13_AY.106
OM080909_MN−CDC−IBX605948889395_2021−12−09_AY.106
OM139218_MN−CDC−IBX225032915213_2021−12−13_AY.106
OL935312_MN−CDC−IBX074003126422_2021−11−30_AY.106
22−006202−069_MN_Hennepin_2021−12−07_AY.106
MN908947 (Wuhan−Hu−1/2019)
0.89
1
0.98
1
0.99
0.77
1
1
0.81
0.99
1
1
1
1
1
1
1
0.2

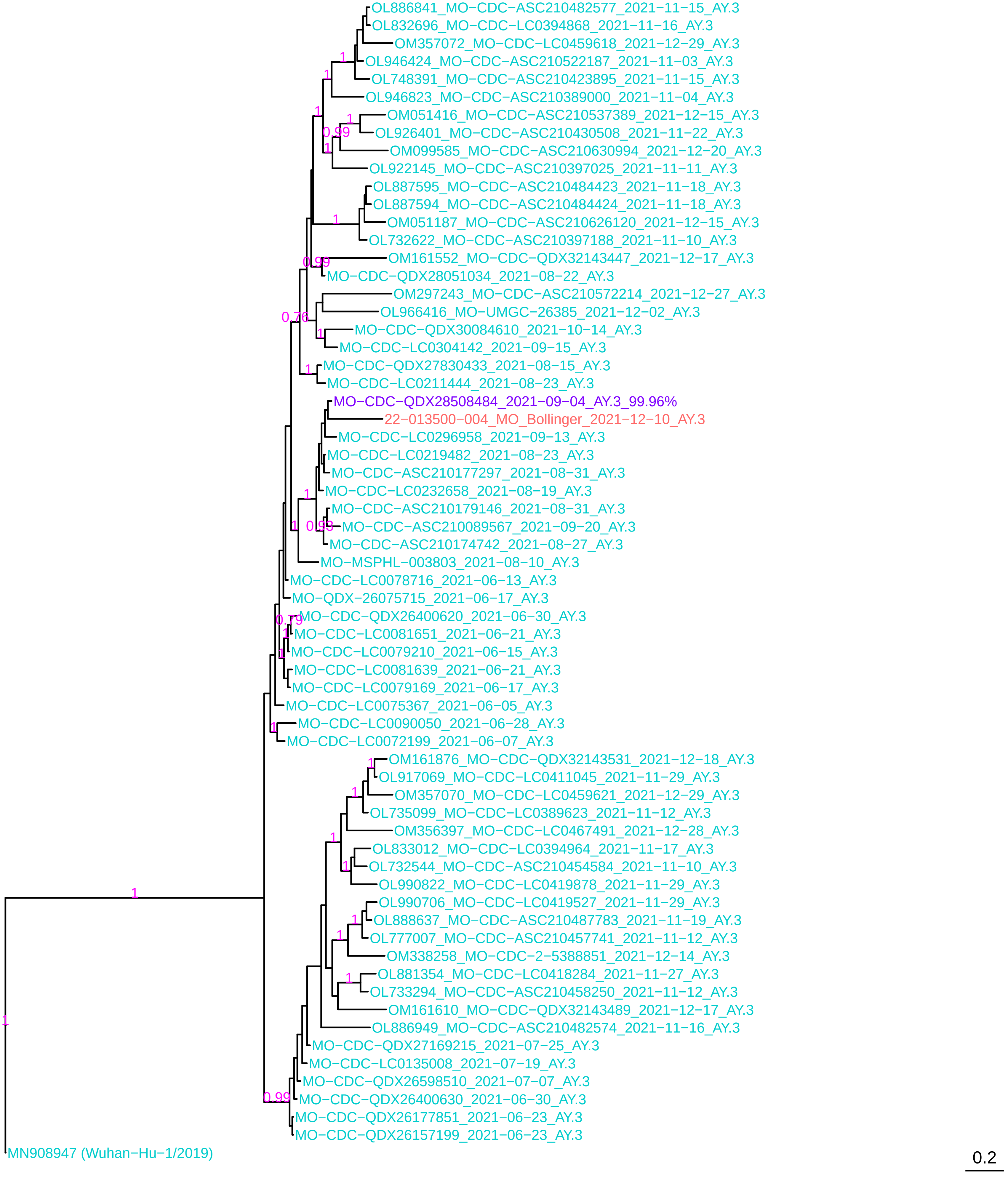
OL886841_MO−CDC−ASC210482577_2021−11−15_AY.3
OL832696_MO−CDC−LC0394868_2021−11−16_AY.3
OM357072_MO−CDC−LC0459618_2021−12−29_AY.3
OL946424_MO−CDC−ASC210522187_2021−11−03_AY.3
OL748391_MO−CDC−ASC210423895_2021−11−15_AY.3
OL946823_MO−CDC−ASC210389000_2021−11−04_AY.3
OM051416_MO−CDC−ASC210537389_2021−12−15_AY.3
OL926401_MO−CDC−ASC210430508_2021−11−22_AY.3
OM099585_MO−CDC−ASC210630994_2021−12−20_AY.3
OL922145_MO−CDC−ASC210397025_2021−11−11_AY.3
OL887595_MO−CDC−ASC210484423_2021−11−18_AY.3
OL887594_MO−CDC−ASC210484424_2021−11−18_AY.3
OM051187_MO−CDC−ASC210626120_2021−12−15_AY.3
OL732622_MO−CDC−ASC210397188_2021−11−10_AY.3
OM161552_MO−CDC−QDX32143447_2021−12−17_AY.3
MO−CDC−QDX28051034_2021−08−22_AY.3
OM297243_MO−CDC−ASC210572214_2021−12−27_AY.3
OL966416_MO−UMGC−26385_2021−12−02_AY.3
MO−CDC−QDX30084610_2021−10−14_AY.3
MO−CDC−LC0304142_2021−09−15_AY.3
MO−CDC−QDX27830433_2021−08−15_AY.3
MO−CDC−LC0211444_2021−08−23_AY.3
MO−CDC−QDX28508484_2021−09−04_AY.3_99.96%
22−013500−004_MO_Bollinger_2021−12−10_AY.3
MO−CDC−LC0296958_2021−09−13_AY.3
MO−CDC−LC0219482_2021−08−23_AY.3
MO−CDC−ASC210177297_2021−08−31_AY.3
MO−CDC−LC0232658_2021−08−19_AY.3
MO−CDC−ASC210179146_2021−08−31_AY.3
MO−CDC−ASC210089567_2021−09−20_AY.3
MO−CDC−ASC210174742_2021−08−27_AY.3
MO−MSPHL−003803_2021−08−10_AY.3
MO−CDC−LC0078716_2021−06−13_AY.3
MO−QDX−26075715_2021−06−17_AY.3
MO−CDC−QDX26400620_2021−06−30_AY.3
MO−CDC−LC0081651_2021−06−21_AY.3
MO−CDC−LC0079210_2021−06−15_AY.3
MO−CDC−LC0081639_2021−06−21_AY.3
MO−CDC−LC0079169_2021−06−17_AY.3
MO−CDC−LC0075367_2021−06−05_AY.3
MO−CDC−LC0090050_2021−06−28_AY.3
MO−CDC−LC0072199_2021−06−07_AY.3
OM161876_MO−CDC−QDX32143531_2021−12−18_AY.3
OL917069_MO−CDC−LC0411045_2021−11−29_AY.3
OM357070_MO−CDC−LC0459621_2021−12−29_AY.3
OL735099_MO−CDC−LC0389623_2021−11−12_AY.3
OM356397_MO−CDC−LC0467491_2021−12−28_AY.3
OL833012_MO−CDC−LC0394964_2021−11−17_AY.3
OL732544_MO−CDC−ASC210454584_2021−11−10_AY.3
OL990822_MO−CDC−LC0419878_2021−11−29_AY.3
OL990706_MO−CDC−LC0419527_2021−11−29_AY.3
OL888637_MO−CDC−ASC210487783_2021−11−19_AY.3
OL777007_MO−CDC−ASC210457741_2021−11−12_AY.3
OM338258_MO−CDC−2−5388851_2021−12−14_AY.3
OL881354_MO−CDC−LC0418284_2021−11−27_AY.3
OL733294_MO−CDC−ASC210458250_2021−11−12_AY.3
OM161610_MO−CDC−QDX32143489_2021−12−17_AY.3
OL886949_MO−CDC−ASC210482574_2021−11−16_AY.3
MO−CDC−QDX27169215_2021−07−25_AY.3
MO−CDC−LC0135008_2021−07−19_AY.3
MO−CDC−QDX26598510_2021−07−07_AY.3
MO−CDC−QDX26400630_2021−06−30_AY.3
MO−CDC−QDX26177851_2021−06−23_AY.3
MO−CDC−QDX26157199_2021−06−23_AY.3
MN908947 (Wuhan−Hu−1/2019)
0.2

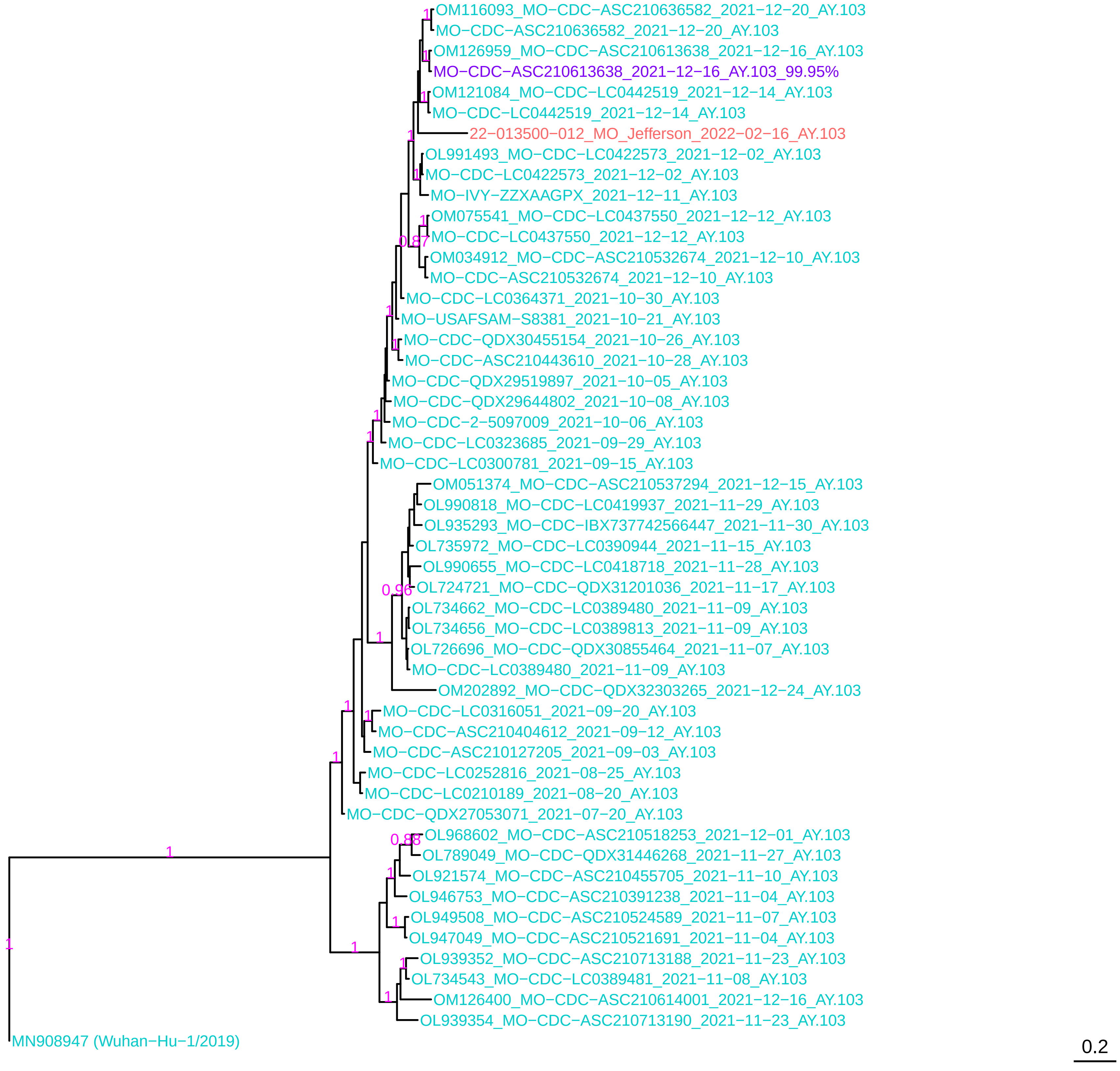
OM116093_MO−CDC−ASC210636582_2021−12−20_AY.103
MO−CDC−ASC210636582_2021−12−20_AY.103
OM126959_MO−CDC−ASC210613638_2021−12−16_AY.103
MO−CDC−ASC210613638_2021−12−16_AY.103_99.95%
OM121084_MO−CDC−LC0442519_2021−12−14_AY.103
MO−CDC−LC0442519_2021−12−14_AY.103
22−013500−012_MO_Jefferson_2022−02−16_AY.103
OL991493_MO−CDC−LC0422573_2021−12−02_AY.103
MO−CDC−LC0422573_2021−12−02_AY.103
MO−IVY−ZZXAAGPX_2021−12−11_AY.103
OM075541_MO−CDC−LC0437550_2021−12−12_AY.103
MO−CDC−LC0437550_2021−12−12_AY.103
OM034912_MO−CDC−ASC210532674_2021−12−10_AY.103
MO−CDC−ASC210532674_2021−12−10_AY.103
MO−CDC−LC0364371_2021−10−30_AY.103
MO−USAFSAM−S8381_2021−10−21_AY.103
MO−CDC−QDX30455154_2021−10−26_AY.103
MO−CDC−ASC210443610_2021−10−28_AY.103
MO−CDC−QDX29519897_2021−10−05_AY.103
MO−CDC−QDX29644802_2021−10−08_AY.103
MO−CDC−2−5097009_2021−10−06_AY.103
MO−CDC−LC0323685_2021−09−29_AY.103
MO−CDC−LC0300781_2021−09−15_AY.103
OM051374_MO−CDC−ASC210537294_2021−12−15_AY.103
OL990818_MO−CDC−LC0419937_2021−11−29_AY.103
OL935293_MO−CDC−IBX737742566447_2021−11−30_AY.103
OL735972_MO−CDC−LC0390944_2021−11−15_AY.103
OL990655_MO−CDC−LC0418718_2021−11−28_AY.103
OL724721_MO−CDC−QDX31201036_2021−11−17_AY.103
OL734662_MO−CDC−LC0389480_2021−11−09_AY.103
OL734656_MO−CDC−LC0389813_2021−11−09_AY.103
OL726696_MO−CDC−QDX30855464_2021−11−07_AY.103
MO−CDC−LC0389480_2021−11−09_AY.103
OM202892_MO−CDC−QDX32303265_2021−12−24_AY.103
MO−CDC−LC0316051_2021−09−20_AY.103
MO−CDC−ASC210404612_2021−09−12_AY.103
MO−CDC−ASC210127205_2021−09−03_AY.103
MO−CDC−LC0252816_2021−08−25_AY.103
MO−CDC−LC0210189_2021−08−20_AY.103
MO−CDC−QDX27053071_2021−07−20_AY.103
OL968602_MO−CDC−ASC210518253_2021−12−01_AY.103
OL789049_MO−CDC−QDX31446268_2021−11−27_AY.103
OL921574_MO−CDC−ASC210455705_2021−11−10_AY.103
OL946753_MO−CDC−ASC210391238_2021−11−04_AY.103
OL949508_MO−CDC−ASC210524589_2021−11−07_AY.103
OL947049_MO−CDC−ASC210521691_2021−11−04_AY.103
OL939352_MO−CDC−ASC210713188_2021−11−23_AY.103
OL734543_MO−CDC−LC0389481_2021−11−08_AY.103
OM126400_MO−CDC−ASC210614001_2021−12−16_AY.103
OL939354_MO−CDC−ASC210713190_2021−11−23_AY.103
MN908947 (Wuhan−Hu−1/2019)
0.87
0.96
0.88
0.2

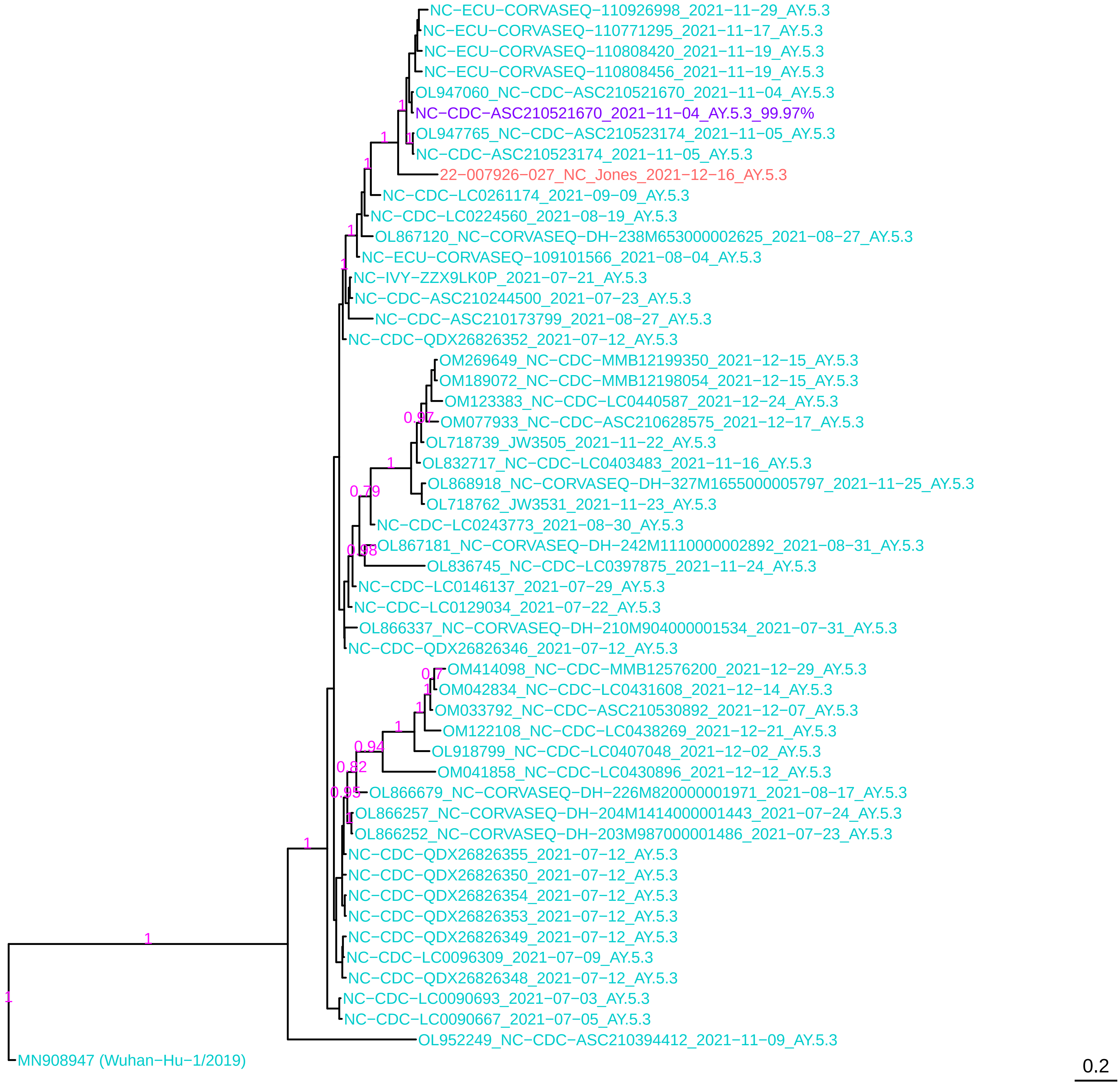
NC−ECU−CORVASEQ−110926998_2021−11−29_AY.5.3
NC−ECU−CORVASEQ−110771295_2021−11−17_AY.5.3
NC−ECU−CORVASEQ−110808420_2021−11−19_AY.5.3
NC−ECU−CORVASEQ−110808456_2021−11−19_AY.5.3
OL947060_NC−CDC−ASC210521670_2021−11−04_AY.5.3
NC−CDC−ASC210521670_2021−11−04_AY.5.3_99.97%
OL947765_NC−CDC−ASC210523174_2021−11−05_AY.5.3
NC−CDC−ASC210523174_2021−11−05_AY.5.3
22−007926−027_NC_Jones_2021−12−16_AY.5.3
NC−CDC−LC0261174_2021−09−09_AY.5.3
NC−CDC−LC0224560_2021−08−19_AY.5.3
OL867120_NC−CORVASEQ−DH−238M653000002625_2021−08−27_AY.5.3
NC−ECU−CORVASEQ−109101566_2021−08−04_AY.5.3
NC−IVY−ZZX9LK0P_2021−07−21_AY.5.3
NC−CDC−ASC210244500_2021−07−23_AY.5.3
NC−CDC−ASC210173799_2021−08−27_AY.5.3
NC−CDC−QDX26826352_2021−07−12_AY.5.3
OM269649_NC−CDC−MMB12199350_2021−12−15_AY.5.3
OM189072_NC−CDC−MMB12198054_2021−12−15_AY.5.3
OM123383_NC−CDC−LC0440587_2021−12−24_AY.5.3
OM077933_NC−CDC−ASC210628575_2021−12−17_AY.5.3
OL718739_JW3505_2021−11−22_AY.5.3
OL832717_NC−CDC−LC0403483_2021−11−16_AY.5.3
OL868918_NC−CORVASEQ−DH−327M1655000005797_2021−11−25_AY.5.3
OL718762_JW3531_2021−11−23_AY.5.3
NC−CDC−LC0243773_2021−08−30_AY.5.3
OL867181_NC−CORVASEQ−DH−242M1110000002892_2021−08−31_AY.5.3
OL836745_NC−CDC−LC0397875_2021−11−24_AY.5.3
NC−CDC−LC0146137_2021−07−29_AY.5.3
NC−CDC−LC0129034_2021−07−22_AY.5.3
OL866337_NC−CORVASEQ−DH−210M904000001534_2021−07−31_AY.5.3
NC−CDC−QDX26826346_2021−07−12_AY.5.3
OM414098_NC−CDC−MMB12576200_2021−12−29_AY.5.3
OM042834_NC−CDC−LC0431608_2021−12−14_AY.5.3
OM033792_NC−CDC−ASC210530892_2021−12−07_AY.5.3
OM122108_NC−CDC−LC0438269_2021−12−21_AY.5.3
OL918799_NC−CDC−LC0407048_2021−12−02_AY.5.3
OM041858_NC−CDC−LC0430896_2021−12−12_AY.5.3
OL866679_NC−CORVASEQ−DH−226M820000001971_2021−08−17_AY.5.3
OL866257_NC−CORVASEQ−DH−204M1414000001443_2021−07−24_AY.5.3
OL866252_NC−CORVASEQ−DH−203M987000001486_2021−07−23_AY.5.3
NC−CDC−QDX26826355_2021−07−12_AY.5.3
NC−CDC−QDX26826350_2021−07−12_AY.5.3
NC−CDC−QDX26826354_2021−07−12_AY.5.3
NC−CDC−QDX26826353_2021−07−12_AY.5.3
NC−CDC−QDX26826349_2021−07−12_AY.5.3
NC−CDC−LC0096309_2021−07−09_AY.5.3
NC−CDC−QDX26826348_2021−07−12_AY.5.3
NC−CDC−LC0090693_2021−07−03_AY.5.3
NC−CDC−LC0090667_2021−07−05_AY.5.3
OL952249_NC−CDC−ASC210394412_2021−11−09_AY.5.3
MN908947 (Wuhan−Hu−1/2019)
0.2

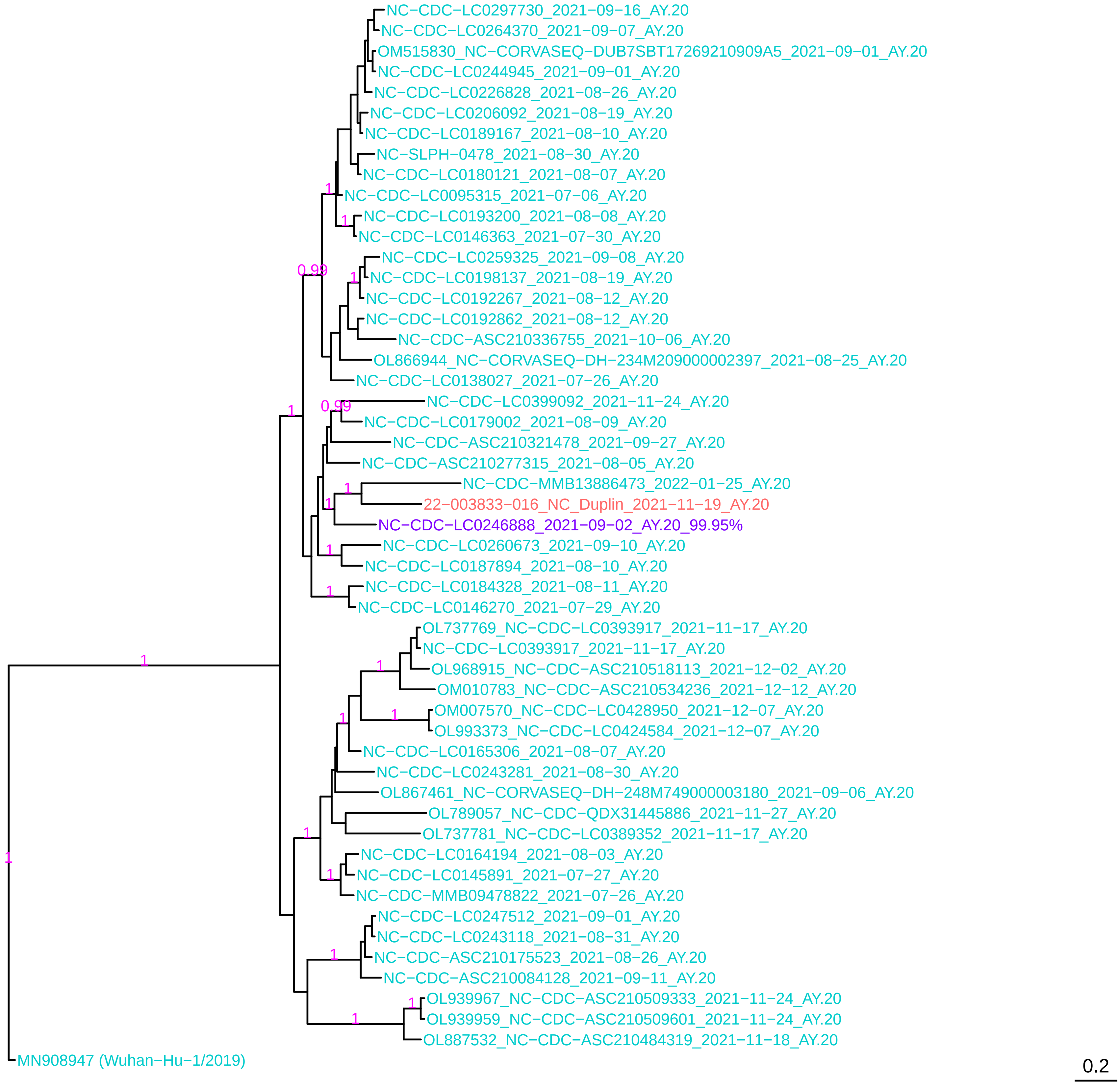
NC−CDC−LC0297730_2021−09−16_AY.20
NC−CDC−LC0264370_2021−09−07_AY.20
OM515830_NC−CORVASEQ−DUB7SBT17269210909A5_2021−09−01_AY.20
NC−CDC−LC0244945_2021−09−01_AY.20
NC−CDC−LC0226828_2021−08−26_AY.20
NC−CDC−LC0206092_2021−08−19_AY.20
NC−CDC−LC0189167_2021−08−10_AY.20
NC−SLPH−0478_2021−08−30_AY.20
NC−CDC−LC0180121_2021−08−07_AY.20
NC−CDC−LC0095315_2021−07−06_AY.20
NC−CDC−LC0193200_2021−08−08_AY.20
NC−CDC−LC0146363_2021−07−30_AY.20
NC−CDC−LC0259325_2021−09−08_AY.20
NC−CDC−LC0198137_2021−08−19_AY.20
NC−CDC−LC0192267_2021−08−12_AY.20
NC−CDC−LC0192862_2021−08−12_AY.20
NC−CDC−ASC210336755_2021−10−06_AY.20
OL866944_NC−CORVASEQ−DH−234M209000002397_2021−08−25_AY.20
NC−CDC−LC0138027_2021−07−26_AY.20
NC−CDC−LC0399092_2021−11−24_AY.20
NC−CDC−LC0179002_2021−08−09_AY.20
NC−CDC−ASC210321478_2021−09−27_AY.20
NC−CDC−ASC210277315_2021−08−05_AY.20
NC−CDC−MMB13886473_2022−01−25_AY.20
22−003833−016_NC_Duplin_2021−11−19_AY.20
NC−CDC−LC0246888_2021−09−02_AY.20_99.95%
NC−CDC−LC0260673_2021−09−10_AY.20
NC−CDC−LC0187894_2021−08−10_AY.20
NC−CDC−LC0184328_2021−08−11_AY.20
NC−CDC−LC0146270_2021−07−29_AY.20
OL737769_NC−CDC−LC0393917_2021−11−17_AY.20
NC−CDC−LC0393917_2021−11−17_AY.20
OL968915_NC−CDC−ASC210518113_2021−12−02_AY.20
OM010783_NC−CDC−ASC210534236_2021−12−12_AY.20
OM007570_NC−CDC−LC0428950_2021−12−07_AY.20
OL993373_NC−CDC−LC0424584_2021−12−07_AY.20
NC−CDC−LC0165306_2021−08−07_AY.20
NC−CDC−LC0243281_2021−08−30_AY.20
OL867461_NC−CORVASEQ−DH−248M749000003180_2021−09−06_AY.20
OL789057_NC−CDC−QDX31445886_2021−11−27_AY.20
OL737781_NC−CDC−LC0389352_2021−11−17_AY.20
NC−CDC−LC0164194_2021−08−03_AY.20
NC−CDC−LC0145891_2021−07−27_AY.20
NC−CDC−MMB09478822_2021−07−26_AY.20
NC−CDC−LC0247512_2021−09−01_AY.20
NC−CDC−LC0243118_2021−08−31_AY.20
NC−CDC−ASC210175523_2021−08−26_AY.20
NC−CDC−ASC210084128_2021−09−11_AY.20
OL939967_NC−CDC−ASC210509333_2021−11−24_AY.20
OL939959_NC−CDC−ASC210509601_2021−11−24_AY.20
OL887532_NC−CDC−ASC210484319_2021−11−18_AY.20
MN908947 (Wuhan−Hu−1/2019)
0.2

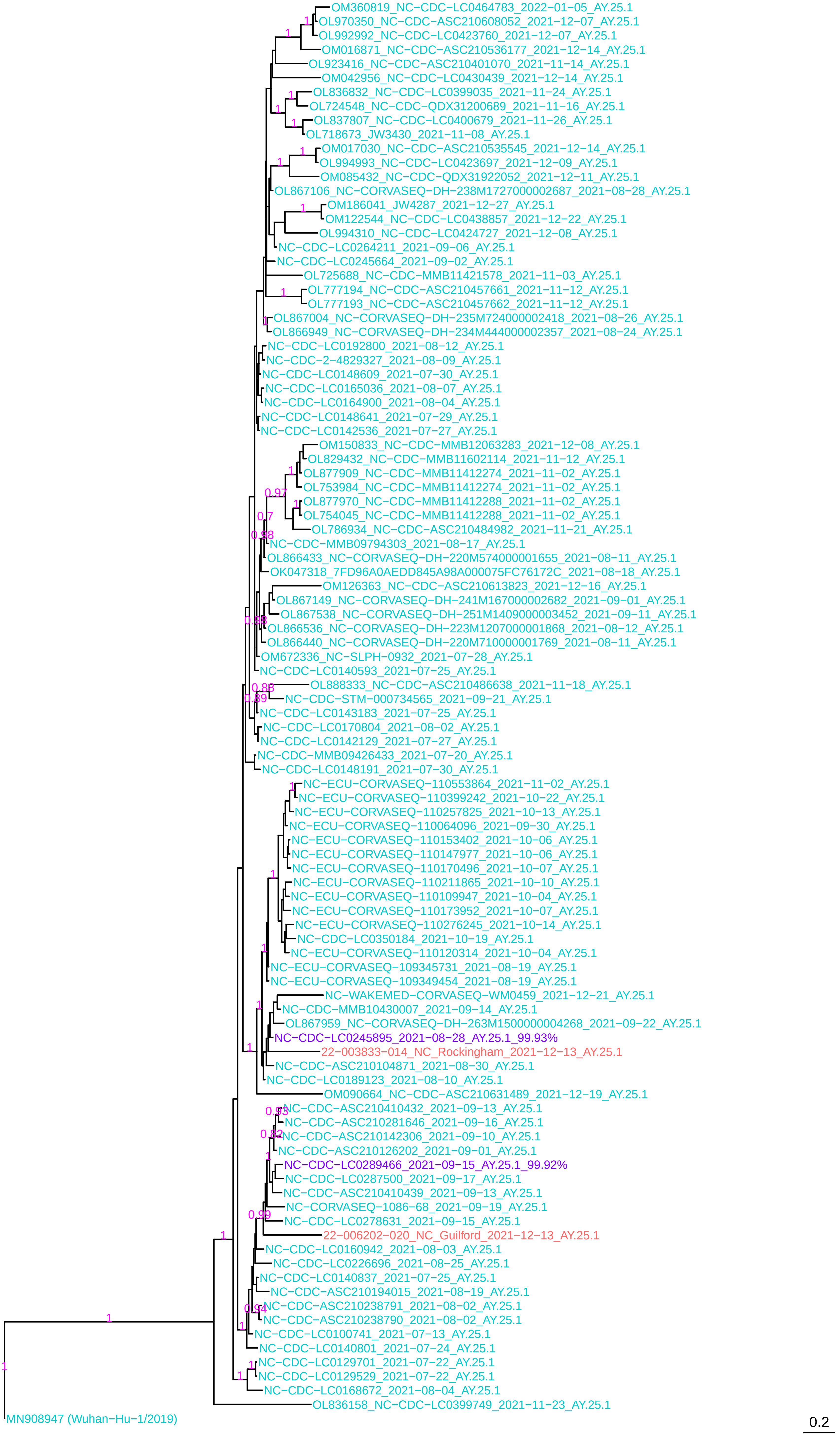
OM360819_NC−CDC−LC0464783_2022−01−05_AY.25.1
OL970350_NC−CDC−ASC210608052_2021−12−07_AY.25.1
OL992992_NC−CDC−LC0423760_2021−12−07_AY.25.1
OM016871_NC−CDC−ASC210536177_2021−12−14_AY.25.1
OL923416_NC−CDC−ASC210401070_2021−11−14_AY.25.1
OM042956_NC−CDC−LC0430439_2021−12−14_AY.25.1
OL836832_NC−CDC−LC0399035_2021−11−24_AY.25.1
OL724548_NC−CDC−QDX31200689_2021−11−16_AY.25.1
OL837807_NC−CDC−LC0400679_2021−11−26_AY.25.1
OL718673_JW3430_2021−11−08_AY.25.1
OM017030_NC−CDC−ASC210535545_2021−12−14_AY.25.1
OL994993_NC−CDC−LC0423697_2021−12−09_AY.25.1
OM085432_NC−CDC−QDX31922052_2021−12−11_AY.25.1
OL867106_NC−CORVASEQ−DH−238M1727000002687_2021−08−28_AY.25.1
OM186041_JW4287_2021−12−27_AY.25.1
OM122544_NC−CDC−LC0438857_2021−12−22_AY.25.1
OL994310_NC−CDC−LC0424727_2021−12−08_AY.25.1
NC−CDC−LC0264211_2021−09−06_AY.25.1
NC−CDC−LC0245664_2021−09−02_AY.25.1
OL725688_NC−CDC−MMB11421578_2021−11−03_AY.25.1
OL777194_NC−CDC−ASC210457661_2021−11−12_AY.25.1
OL777193_NC−CDC−ASC210457662_2021−11−12_AY.25.1
OL867004_NC−CORVASEQ−DH−235M724000002418_2021−08−26_AY.25.1
OL866949_NC−CORVASEQ−DH−234M444000002357_2021−08−24_AY.25.1
NC−CDC−LC0192800_2021−08−12_AY.25.1
NC−CDC−2−4829327_2021−08−09_AY.25.1
NC−CDC−LC0148609_2021−07−30_AY.25.1
NC−CDC−LC0165036_2021−08−07_AY.25.1
NC−CDC−LC0164900_2021−08−04_AY.25.1
NC−CDC−LC0148641_2021−07−29_AY.25.1
NC−CDC−LC0142536_2021−07−27_AY.25.1
OM150833_NC−CDC−MMB12063283_2021−12−08_AY.25.1
OL829432_NC−CDC−MMB11602114_2021−11−12_AY.25.1
OL877909_NC−CDC−MMB11412274_2021−11−02_AY.25.1
OL753984_NC−CDC−MMB11412274_2021−11−02_AY.25.1
OL877970_NC−CDC−MMB11412288_2021−11−02_AY.25.1
OL754045_NC−CDC−MMB11412288_2021−11−02_AY.25.1
OL786934_NC−CDC−ASC210484982_2021−11−21_AY.25.1
NC−CDC−MMB09794303_2021−08−17_AY.25.1
OL866433_NC−CORVASEQ−DH−220M574000001655_2021−08−11_AY.25.1
OK047318_7FD96A0AEDD845A98A000075FC76172C_2021−08−18_AY.25.1
OM126363_NC−CDC−ASC210613823_2021−12−16_AY.25.1
OL867149_NC−CORVASEQ−DH−241M167000002682_2021−09−01_AY.25.1
OL867538_NC−CORVASEQ−DH−251M1409000003452_2021−09−11_AY.25.1
OL866536_NC−CORVASEQ−DH−223M1207000001868_2021−08−12_AY.25.1
OL866440_NC−CORVASEQ−DH−220M710000001769_2021−08−11_AY.25.1
OM672336_NC−SLPH−0932_2021−07−28_AY.25.1
NC−CDC−LC0140593_2021−07−25_AY.25.1
OL888333_NC−CDC−ASC210486638_2021−11−18_AY.25.1
NC−CDC−STM−000734565_2021−09−21_AY.25.1
NC−CDC−LC0143183_2021−07−25_AY.25.1
NC−CDC−LC0170804_2021−08−02_AY.25.1
NC−CDC−LC0142129_2021−07−27_AY.25.1
NC−CDC−MMB09426433_2021−07−20_AY.25.1
NC−CDC−LC0148191_2021−07−30_AY.25.1
NC−ECU−CORVASEQ−110553864_2021−11−02_AY.25.1
NC−ECU−CORVASEQ−110399242_2021−10−22_AY.25.1
NC−ECU−CORVASEQ−110257825_2021−10−13_AY.25.1
NC−ECU−CORVASEQ−110064096_2021−09−30_AY.25.1
NC−ECU−CORVASEQ−110153402_2021−10−06_AY.25.1
NC−ECU−CORVASEQ−110147977_2021−10−06_AY.25.1
NC−ECU−CORVASEQ−110170496_2021−10−07_AY.25.1
NC−ECU−CORVASEQ−110211865_2021−10−10_AY.25.1
NC−ECU−CORVASEQ−110109947_2021−10−04_AY.25.1
NC−ECU−CORVASEQ−110173952_2021−10−07_AY.25.1
NC−ECU−CORVASEQ−110276245_2021−10−14_AY.25.1
NC−CDC−LC0350184_2021−10−19_AY.25.1
NC−ECU−CORVASEQ−110120314_2021−10−04_AY.25.1
NC−ECU−CORVASEQ−109345731_2021−08−19_AY.25.1
NC−ECU−CORVASEQ−109349454_2021−08−19_AY.25.1
NC−WAKEMED−CORVASEQ−WM0459_2021−12−21_AY.25.1
NC−CDC−MMB10430007_2021−09−14_AY.25.1
OL867959_NC−CORVASEQ−DH−263M1500000004268_2021−09−22_AY.25.1
NC−CDC−LC0245895_2021−08−28_AY.25.1_99.93%
22−003833−014_NC_Rockingham_2021−12−13_AY.25.1
NC−CDC−ASC210104871_2021−08−30_AY.25.1
NC−CDC−LC0189123_2021−08−10_AY.25.1
OM090664_NC−CDC−ASC210631489_2021−12−19_AY.25.1
NC−CDC−ASC210410432_2021−09−13_AY.25.1
NC−CDC−ASC210281646_2021−09−16_AY.25.1
NC−CDC−ASC210142306_2021−09−10_AY.25.1
NC−CDC−ASC210126202_2021−09−01_AY.25.1
NC−CDC−LC0289466_2021−09−15_AY.25.1_99.92%
NC−CDC−LC0287500_2021−09−17_AY.25.1
NC−CDC−ASC210410439_2021−09−13_AY.25.1
NC−CORVASEQ−1086−68_2021−09−19_AY.25.1
NC−CDC−LC0278631_2021−09−15_AY.25.1
22−006202−020_NC_Guilford_2021−12−13_AY.25.1
NC−CDC−LC0160942_2021−08−03_AY.25.1
NC−CDC−LC0226696_2021−08−25_AY.25.1
NC−CDC−LC0140837_2021−07−25_AY.25.1
NC−CDC−ASC210194015_2021−08−19_AY.25.1
NC−CDC−ASC210238791_2021−08−02_AY.25.1
NC−CDC−ASC210238790_2021−08−02_AY.25.1
NC−CDC−LC0100741_2021−07−13_AY.25.1
NC−CDC−LC0140801_2021−07−24_AY.25.1
NC−CDC−LC0129701_2021−07−22_AY.25.1
NC−CDC−LC0129529_2021−07−22_AY.25.1
NC−CDC−LC0168672_2021−08−04_AY.25.1
OL836158_NC−CDC−LC0399749_2021−11−23_AY.25.1
MN908947 (Wuhan−Hu−1/2019)
0.97
0.7
0.98
0.88
0.89
0.93
0.82
0.99
0.94
0.2

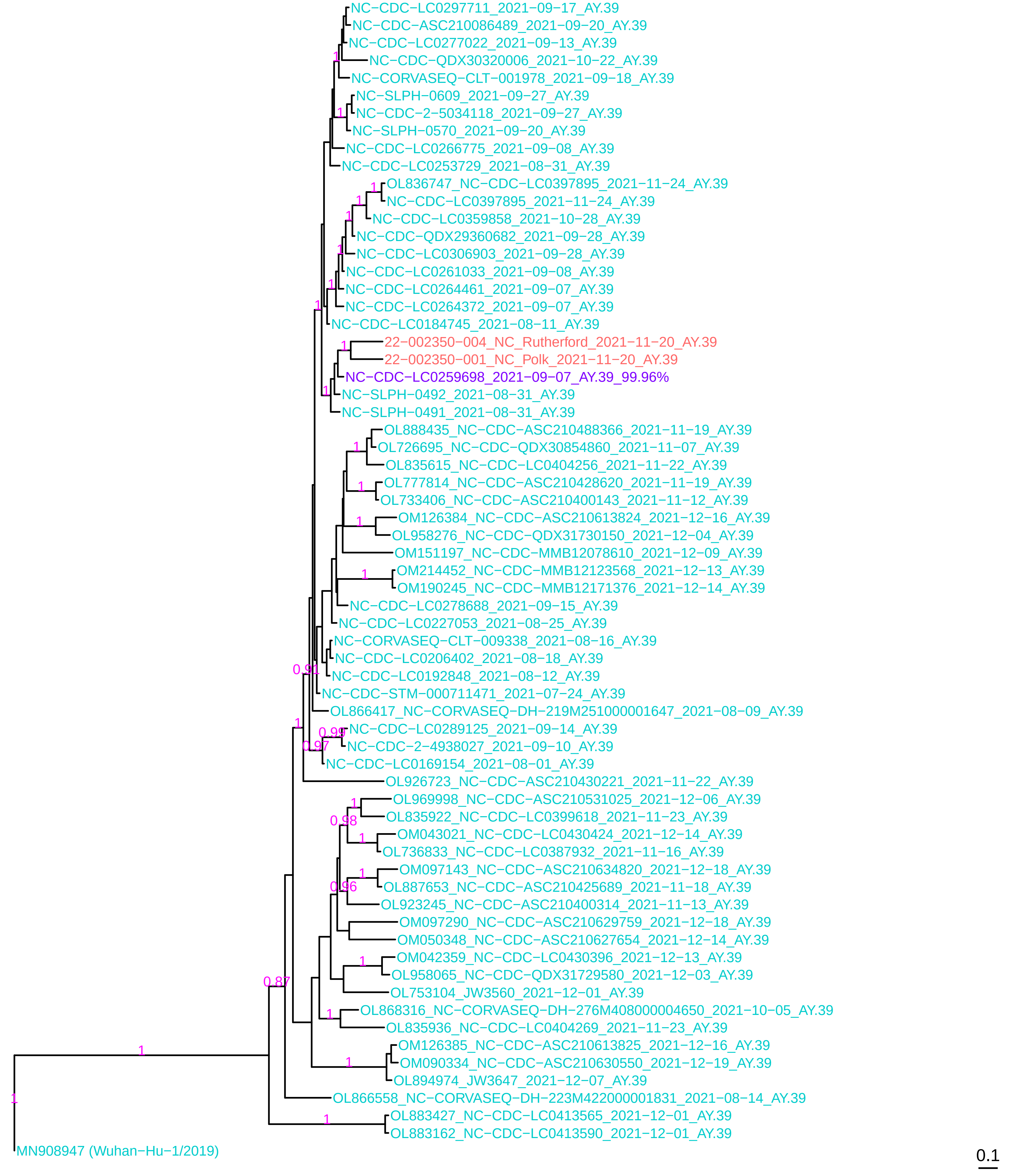
NC−CDC−LC0297711_2021−09−17_AY.39
NC−CDC−ASC210086489_2021−09−20_AY.39
NC−CDC−LC0277022_2021−09−13_AY.39
NC−CDC−QDX30320006_2021−10−22_AY.39
NC−CORVASEQ−CLT−001978_2021−09−18_AY.39
NC−SLPH−0609_2021−09−27_AY.39
NC−CDC−2−5034118_2021−09−27_AY.39
NC−SLPH−0570_2021−09−20_AY.39
NC−CDC−LC0266775_2021−09−08_AY.39
NC−CDC−LC0253729_2021−08−31_AY.39
OL836747_NC−CDC−LC0397895_2021−11−24_AY.39
NC−CDC−LC0397895_2021−11−24_AY.39
NC−CDC−LC0359858_2021−10−28_AY.39
NC−CDC−QDX29360682_2021−09−28_AY.39
NC−CDC−LC0306903_2021−09−28_AY.39
NC−CDC−LC0261033_2021−09−08_AY.39
NC−CDC−LC0264461_2021−09−07_AY.39
NC−CDC−LC0264372_2021−09−07_AY.39
NC−CDC−LC0184745_2021−08−11_AY.39
22−002350−004_NC_Rutherford_2021−11−20_AY.39
22−002350−001_NC_Polk_2021−11−20_AY.39
NC−CDC−LC0259698_2021−09−07_AY.39_99.96%
NC−SLPH−0492_2021−08−31_AY.39
NC−SLPH−0491_2021−08−31_AY.39
OL888435_NC−CDC−ASC210488366_2021−11−19_AY.39
OL726695_NC−CDC−QDX30854860_2021−11−07_AY.39
OL835615_NC−CDC−LC0404256_2021−11−22_AY.39
OL777814_NC−CDC−ASC210428620_2021−11−19_AY.39
OL733406_NC−CDC−ASC210400143_2021−11−12_AY.39
OM126384_NC−CDC−ASC210613824_2021−12−16_AY.39
OL958276_NC−CDC−QDX31730150_2021−12−04_AY.39
OM151197_NC−CDC−MMB12078610_2021−12−09_AY.39
OM214452_NC−CDC−MMB12123568_2021−12−13_AY.39
OM190245_NC−CDC−MMB12171376_2021−12−14_AY.39
NC−CDC−LC0278688_2021−09−15_AY.39
NC−CDC−LC0227053_2021−08−25_AY.39
NC−CORVASEQ−CLT−009338_2021−08−16_AY.39
NC−CDC−LC0206402_2021−08−18_AY.39
NC−CDC−LC0192848_2021−08−12_AY.39
NC−CDC−STM−000711471_2021−07−24_AY.39
OL866417_NC−CORVASEQ−DH−219M251000001647_2021−08−09_AY.39
NC−CDC−LC0289125_2021−09−14_AY.39
NC−CDC−2−4938027_2021−09−10_AY.39
NC−CDC−LC0169154_2021−08−01_AY.39
OL926723_NC−CDC−ASC210430221_2021−11−22_AY.39
OL969998_NC−CDC−ASC210531025_2021−12−06_AY.39
OL835922_NC−CDC−LC0399618_2021−11−23_AY.39
OM043021_NC−CDC−LC0430424_2021−12−14_AY.39
OL736833_NC−CDC−LC0387932_2021−11−16_AY.39
OM097143_NC−CDC−ASC210634820_2021−12−18_AY.39
OL887653_NC−CDC−ASC210425689_2021−11−18_AY.39
OL923245_NC−CDC−ASC210400314_2021−11−13_AY.39
OM097290_NC−CDC−ASC210629759_2021−12−18_AY.39
OM050348_NC−CDC−ASC210627654_2021−12−14_AY.39
OM042359_NC−CDC−LC0430396_2021−12−13_AY.39
OL958065_NC−CDC−QDX31729580_2021−12−03_AY.39
OL753104_JW3560_2021−12−01_AY.39
OL868316_NC−CORVASEQ−DH−276M408000004650_2021−10−05_AY.39
OL835936_NC−CDC−LC0404269_2021−11−23_AY.39
OM126385_NC−CDC−ASC210613825_2021−12−16_AY.39
OM090334_NC−CDC−ASC210630550_2021−12−19_AY.39
OL894974_JW3647_2021−12−07_AY.39
OL866558_NC−CORVASEQ−DH−223M422000001831_2021−08−14_AY.39
OL883427_NC−CDC−LC0413565_2021−12−01_AY.39
OL883162_NC−CDC−LC0413590_2021−12−01_AY.39
MN908947 (Wuhan−Hu−1/2019)
0.1

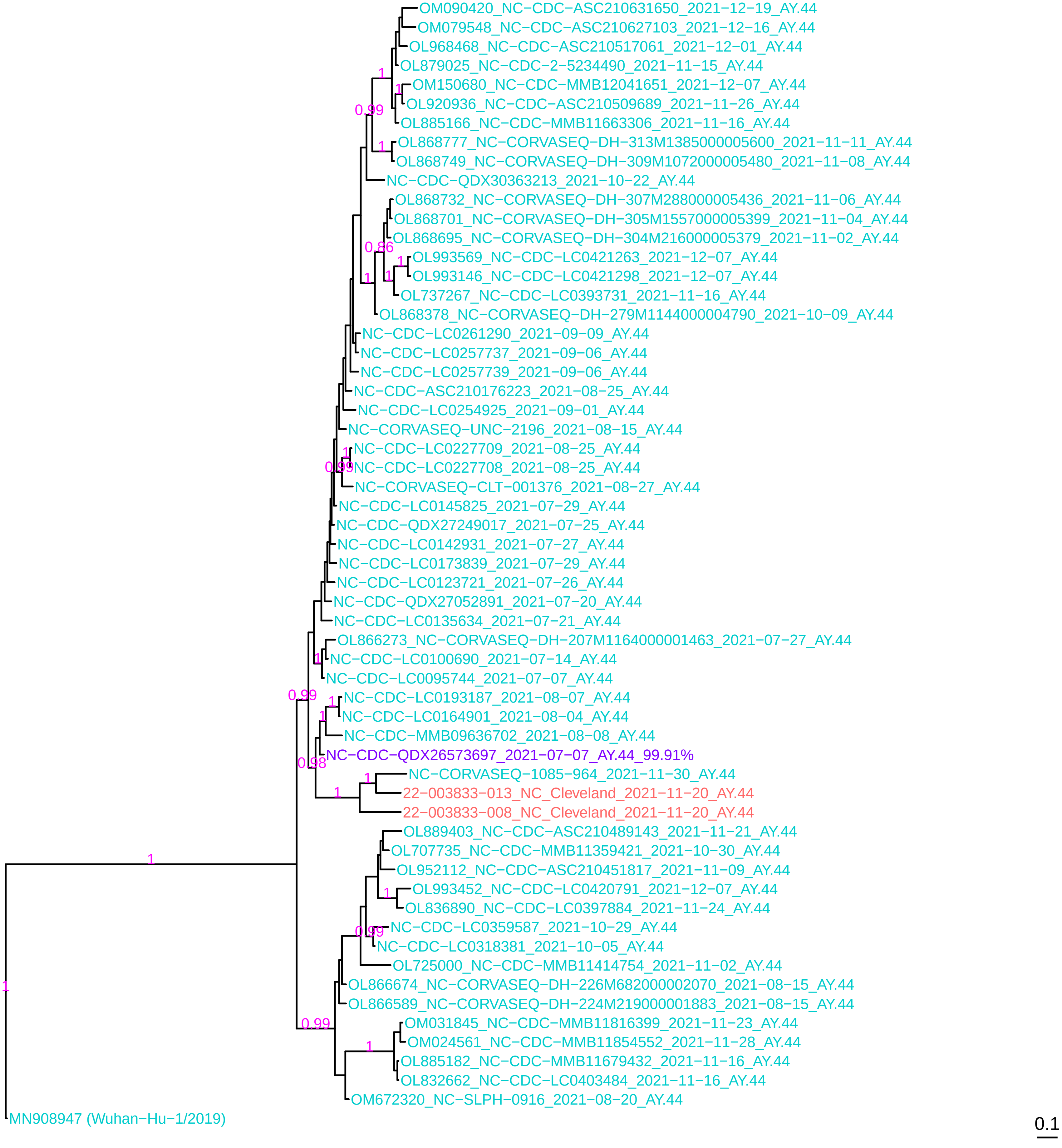
OM090420_NC−CDC−ASC210631650_2021−12−19_AY.44
OM079548_NC−CDC−ASC210627103_2021−12−16_AY.44
OL968468_NC−CDC−ASC210517061_2021−12−01_AY.44
OL879025_NC−CDC−2−5234490_2021−11−15_AY.44
OM150680_NC−CDC−MMB12041651_2021−12−07_AY.44
OL920936_NC−CDC−ASC210509689_2021−11−26_AY.44
OL885166_NC−CDC−MMB11663306_2021−11−16_AY.44
OL868777_NC−CORVASEQ−DH−313M1385000005600_2021−11−11_AY.44
OL868749_NC−CORVASEQ−DH−309M1072000005480_2021−11−08_AY.44
NC−CDC−QDX30363213_2021−10−22_AY.44
OL868732_NC−CORVASEQ−DH−307M288000005436_2021−11−06_AY.44
OL868701_NC−CORVASEQ−DH−305M1557000005399_2021−11−04_AY.44
OL868695_NC−CORVASEQ−DH−304M216000005379_2021−11−02_AY.44
OL993569_NC−CDC−LC0421263_2021−12−07_AY.44
OL993146_NC−CDC−LC0421298_2021−12−07_AY.44
OL737267_NC−CDC−LC0393731_2021−11−16_AY.44
OL868378_NC−CORVASEQ−DH−279M1144000004790_2021−10−09_AY.44
NC−CDC−LC0261290_2021−09−09_AY.44
NC−CDC−LC0257737_2021−09−06_AY.44
NC−CDC−LC0257739_2021−09−06_AY.44
NC−CDC−ASC210176223_2021−08−25_AY.44
NC−CDC−LC0254925_2021−09−01_AY.44
NC−CORVASEQ−UNC−2196_2021−08−15_AY.44
NC−CDC−LC0227709_2021−08−25_AY.44
NC−CDC−LC0227708_2021−08−25_AY.44
NC−CORVASEQ−CLT−001376_2021−08−27_AY.44
NC−CDC−LC0145825_2021−07−29_AY.44
NC−CDC−QDX27249017_2021−07−25_AY.44
NC−CDC−LC0142931_2021−07−27_AY.44
NC−CDC−LC0173839_2021−07−29_AY.44
NC−CDC−LC0123721_2021−07−26_AY.44
NC−CDC−QDX27052891_2021−07−20_AY.44
NC−CDC−LC0135634_2021−07−21_AY.44
OL866273_NC−CORVASEQ−DH−207M1164000001463_2021−07−27_AY.44
NC−CDC−LC0100690_2021−07−14_AY.44
NC−CDC−LC0095744_2021−07−07_AY.44
NC−CDC−LC0193187_2021−08−07_AY.44
NC−CDC−LC0164901_2021−08−04_AY.44
NC−CDC−MMB09636702_2021−08−08_AY.44
NC−CDC−QDX26573697_2021−07−07_AY.44_99.91%
NC−CORVASEQ−1085−964_2021−11−30_AY.44
22−003833−013_NC_Cleveland_2021−11−20_AY.44
22−003833−008_NC_Cleveland_2021−11−20_AY.44
OL889403_NC−CDC−ASC210489143_2021−11−21_AY.44
OL707735_NC−CDC−MMB11359421_2021−10−30_AY.44
OL952112_NC−CDC−ASC210451817_2021−11−09_AY.44
OL993452_NC−CDC−LC0420791_2021−12−07_AY.44
OL836890_NC−CDC−LC0397884_2021−11−24_AY.44
NC−CDC−LC0359587_2021−10−29_AY.44
NC−CDC−LC0318381_2021−10−05_AY.44
OL725000_NC−CDC−MMB11414754_2021−11−02_AY.44
OL866674_NC−CORVASEQ−DH−226M682000002070_2021−08−15_AY.44
OL866589_NC−CORVASEQ−DH−224M219000001883_2021−08−15_AY.44
OM031845_NC−CDC−MMB11816399_2021−11−23_AY.44
OM024561_NC−CDC−MMB11854552_2021−11−28_AY.44
OL885182_NC−CDC−MMB11679432_2021−11−16_AY.44
OL832662_NC−CDC−LC0403484_2021−11−16_AY.44
OM672320_NC−SLPH−0916_2021−08−20_AY.44
MN908947 (Wuhan−Hu−1/2019)
0.1

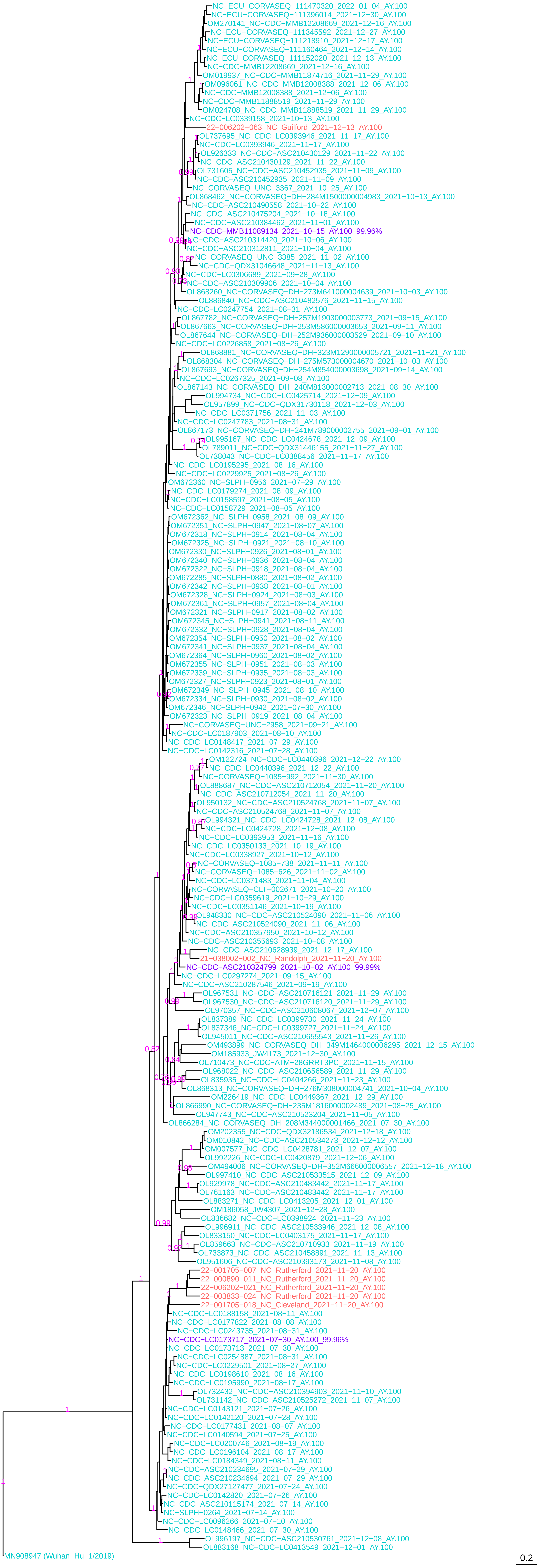
NC-ECU-CORVASEQ-111470320_2022-01-04_AY.100
NC-ECU-CORVASEQ-111396014_2021-12-30_AY.100
OM270141_NC-CDC-MMB12208669_2021-12-16_AY.100
NC-ECU-CORVASEQ-111345592_2021-12-27_AY.100
NC-ECU-CORVASEQ-111218910_2021-12-17_AY.100
NC-ECU-CORVASEQ-111160464_2021-12-14_AY.100
NC-ECU-CORVASEQ-111152020_2021-12-13_AY.100
NC-CDC-MMB12208669_2021-12-16_AY.100
OM019937_NC-CDC-MMB11874716_2021-11-29_AY.100
OM096061_NC-CDC-MMB12008388_2021-12-06_AY.100
NC-CDC-MMB12008388_2021-12-06_AY.100
NC-CDC-MMB11888519_2021-11-29_AY.100
OM024708_NC-CDC-MMB11888519_2021-11-29_AY.100
NC-CDC-LC0339158_2021-10-13_AY.100
22-006202-063_NC_Guilford_2021-12-13_AY.100
OL737695_NC-CDC-LC0393946_2021-11-17_AY.100
NC-CDC-LC0393946_2021-11-17_AY.100
OL926333_NC-CDC-ASC210430129_2021-11-22_AY.100
NC-CDC-ASC210430129_2021-11-22_AY.100
OL731605_NC-CDC-ASC210452935_2021-11-09_AY.100
NC-CDC-ASC210452935_2021-11-09_AY.100
NC-CORVASEQ-UNC-3367_2021-10-25_AY.100
OL868462_NC-CORVASEQ-DH-284M1500000004983_2021-10-13_AY.100
NC-CDC-ASC210490558_2021-10-22_AY.100
NC-CDC-ASC210475204_2021-10-18_AY.100
NC-CDC-ASC210384462_2021-11-01_AY.100
NC-CDC-MMB11089134_2021-10-15_AY.100_99.96%
NC-CDC-ASC210314420_2021-10-06_AY.100
NC-CDC-ASC210312811_2021-10-04_AY.100
NC-CORVASEQ-UNC-3385_2021-11-02_AY.100
NC-CDC-QDX31046648_2021-11-13_AY.100
NC-CDC-LC0306689_2021-09-28_AY.100
NC-CDC-ASC210309906_2021-10-04_AY.100
OL868260_NC-CORVASEQ-DH-273M641000004639_2021-10-03_AY.100
OL886840_NC-CDC-ASC210482576_2021-11-15_AY.100
NC-CDC-LC0247754_2021-08-31_AY.100
OL867782_NC-CORVASEQ-DH-257M1903000003773_2021-09-15_AY.100
OL867663_NC-CORVASEQ-DH-253M586000003653_2021-09-11_AY.100
OL867644_NC-CORVASEQ-DH-252M936000003529_2021-09-10_AY.100
NC-CDC-LC0226858_2021-08-26_AY.100
OL868881_NC-CORVASEQ-DH-323M1290000005721_2021-11-21_AY.100
OL868304_NC-CORVASEQ-DH-275M573000004670_2021-10-03_AY.100
OL867693_NC-CORVASEQ-DH-254M854000003698_2021-09-14_AY.100
NC-CDC-LC0267325_2021-09-08_AY.100
OL867143_NC-CORVASEQ-DH-240M813000002713_2021-08-30_AY.100
OL994734_NC-CDC-LC0425714_2021-12-09_AY.100
OL957899_NC-CDC-QDX31730118_2021-12-03_AY.100
NC-CDC-LC0371756_2021-11-03_AY.100
NC-CDC-LC0247783_2021-08-31_AY.100
OL867173_NC-CORVASEQ-DH-241M789000002755_2021-09-01_AY.100
OL995167_NC-CDC-LC0424678_2021-12-09_AY.100
OL789011_NC-CDC-QDX31446155_2021-11-27_AY.100
OL738043_NC-CDC-LC0388456_2021-11-17_AY.100
NC-CDC-LC0195295_2021-08-16_AY.100
NC-CDC-LC0229925_2021-08-26_AY.100
OM672360_NC-SLPH-0956_2021-07-29_AY.100
NC-CDC-LC0179274_2021-08-09_AY.100
NC-CDC-LC0158597_2021-08-05_AY.100
NC-CDC-LC0158729_2021-08-05_AY.100
OM672362_NC-SLPH-0958_2021-08-09_AY.100
OM672351_NC-SLPH-0947_2021-08-07_AY.100
OM672318_NC-SLPH-0914_2021-08-04_AY.100
OM672325_NC-SLPH-0921_2021-08-10_AY.100
OM672330_NC-SLPH-0926_2021-08-01_AY.100
OM672340_NC-SLPH-0936_2021-08-04_AY.100
OM672322_NC-SLPH-0918_2021-08-04_AY.100
OM672285_NC-SLPH-0880_2021-08-02_AY.100
OM672342_NC-SLPH-0938_2021-08-01_AY.100
OM672328_NC-SLPH-0924_2021-08-03_AY.100
OM672361_NC-SLPH-0957_2021-08-04_AY.100
OM672321_NC-SLPH-0917_2021-08-02_AY.100
OM672345_NC-SLPH-0941_2021-08-11_AY.100
OM672332_NC-SLPH-0928_2021-08-04_AY.100
OM672354_NC-SLPH-0950_2021-08-02_AY.100
OM672341_NC-SLPH-0937_2021-08-04_AY.100
OM672364_NC-SLPH-0960_2021-08-02_AY.100
OM672355_NC-SLPH-0951_2021-08-03_AY.100
OM672339_NC-SLPH-0935_2021-08-03_AY.100
OM672327_NC-SLPH-0923_2021-08-01_AY.100
OM672349_NC-SLPH-0945_2021-08-10_AY.100
OM672334_NC-SLPH-0930_2021-08-02_AY.100
OM672346_NC-SLPH-0942_2021-07-30_AY.100
OM672323_NC-SLPH-0919_2021-08-04_AY.100
NC-CORVASEQ-UNC-2958_2021-09-21_AY.100
NC-CDC-LC0187903_2021-08-10_AY.100
NC-CDC-LC0148417_2021-07-29_AY.100
NC-CDC-LC0142316_2021-07-28_AY.100
OM122724_NC-CDC-LC0440396_2021-12-22_AY.100
NC-CDC-LC0440396_2021-12-22_AY.100
NC-CORVASEQ-1085-992_2021-11-30_AY.100
OL888687_NC-CDC-ASC210712054_2021-11-20_AY.100
NC-CDC-ASC210712054_2021-11-20_AY.100
OL950132_NC-CDC-ASC210524768_2021-11-07_AY.100
NC-CDC-ASC210524768_2021-11-07_AY.100
OL994321_NC-CDC-LC0424728_2021-12-08_AY.100
NC-CDC-LC0424728_2021-12-08_AY.100
NC-CDC-LC0393953_2021-11-16_AY.100
NC-CDC-LC0350133_2021-10-19_AY.100
NC-CDC-LC0338927_2021-10-12_AY.100
NC-CORVASEQ-1085-738_2021-11-11_AY.100
NC-CORVASEQ-1085-626_2021-11-02_AY.100
NC-CDC-LC0371483_2021-11-04_AY.100
NC-CORVASEQ-CLT-002671_2021-10-20_AY.100
NC-CDC-LC0359619_2021-10-29_AY.100
NC-CDC-LC0351146_2021-10-19_AY.100
OL948330_NC-CDC-ASC210524090_2021-11-06_AY.100
NC-CDC-ASC210524090_2021-11-06_AY.100
NC-CDC-ASC210357950_2021-10-12_AY.100
NC-CDC-ASC210355693_2021-10-08_AY.100
NC-CDC-ASC210628939_2021-12-17_AY.100
21-038002-002_NC_Randolph_2021-11-20_AY.100
NC-CDC-ASC210324799_2021-10-02_AY.100_99.99%
NC-CDC-LC0297274_2021-09-15_AY.100
NC-CDC-ASC210287546_2021-09-19_AY.100
OL967531_NC-CDC-ASC210716121_2021-11-29_AY.100
OL967530_NC-CDC-ASC210716120_2021-11-29_AY.100
OL970357_NC-CDC-ASC210608067_2021-12-07_AY.100
OL837389_NC-CDC-LC0399730_2021-11-24_AY.100
OL837346_NC-CDC-LC0399727_2021-11-24_AY.100
OL945011_NC-CDC-ASC210655543_2021-11-26_AY.100
OM493899_NC-CORVASEQ-DH-349M1464000006295_2021-12-15_AY.100
OM185933_JW4173_2021-12-30_AY.100
OL710473_NC-CDC-ATM-28GRRT3PC_2021-11-15_AY.100
OL968022_NC-CDC-ASC210656589_2021-11-29_AY.100
OL835935_NC-CDC-LC0404266_2021-11-23_AY.100
OL868313_NC-CORVASEQ-DH-276M308000004741_2021-10-04_AY.100
OM226419_NC-CDC-LC0449367_2021-12-29_AY.100
OL866990_NC-CORVASEQ-DH-235M1816000002489_2021-08-25_AY.100
OL947743_NC-CDC-ASC210523204_2021-11-05_AY.100
OL866284_NC-CORVASEQ-DH-208M344000001466_2021-07-30_AY.100
OM202355_NC-CDC-QDX32186534_2021-12-18_AY.100
OM010842_NC-CDC-ASC210534273_2021-12-12_AY.100
OM007577_NC-CDC-LC0428781_2021-12-07_AY.100
OL992226_NC-CDC-LC0420879_2021-12-06_AY.100
OM494006_NC-CORVASEQ-DH-352M666000006557_2021-12-18_AY.100
OL997410_NC-CDC-ASC210533515_2021-12-09_AY.100
OL929978_NC-CDC-ASC210483442_2021-11-17_AY.100
OL761163_NC-CDC-ASC210483442_2021-11-17_AY.100
OL883271_NC-CDC-LC0413205_2021-12-01_AY.100
OM186058_JW4307_2021-12-28_AY.100
OL836682_NC-CDC-LC0398924_2021-11-23_AY.100
OL996911_NC-CDC-ASC210533946_2021-12-08_AY.100
OL833150_NC-CDC-LC0403175_2021-11-17_AY.100
OL859663_NC-CDC-ASC210710933_2021-11-19_AY.100
OL733873_NC-CDC-ASC210458891_2021-11-13_AY.100
OL951606_NC-CDC-ASC210393173_2021-11-08_AY.100
22-001705-007_NC_Rutherford_2021-11-20_AY.100
22-000890-011_NC_Rutherford_2021-11-20_AY.100
22-006202-021_NC_Rutherford_2021-11-20_AY.100
22-003833-024_NC_Rutherford_2021-11-20_AY.100
22-001705-018_NC_Cleveland_2021-11-20_AY.100
NC-CDC-LC0188158_2021-08-11_AY.100
NC-CDC-LC0177822_2021-08-08_AY.100
NC-CDC-LC0243735_2021-08-31_AY.100
NC-CDC-LC0173717_2021-07-30_AY.100_99.96%
NC-CDC-LC0173713_2021-07-30_AY.100
NC-CDC-LC0254887_2021-08-31_AY.100
NC-CDC-LC0229501_2021-08-27_AY.100
NC-CDC-LC0198610_2021-08-16_AY.100
NC-CDC-LC0195990_2021-08-17_AY.100
OL732432_NC-CDC-ASC210394903_2021-11-10_AY.100
OL731142_NC-CDC-ASC210525272_2021-11-07_AY.100
NC-CDC-LC0143121_2021-07-26_AY.100
NC-CDC-LC0142120_2021-07-28_AY.100
NC-CDC-LC0177431_2021-08-07_AY.100
NC-CDC-LC0140594_2021-07-25_AY.100
NC-CDC-LC0200746_2021-08-19_AY.100
NC-CDC-LC0196104_2021-08-17_AY.100
NC-CDC-LC0184349_2021-08-11_AY.100
NC-CDC-ASC210234695_2021-07-29_AY.100
NC-CDC-ASC210234694_2021-07-29_AY.100
NC-CDC-QDX27127477_2021-07-24_AY.100
NC-CDC-LC0142820_2021-07-26_AY.100
NC-CDC-ASC210115174_2021-07-14_AY.100
NC-SLPH-0264_2021-07-14_AY.100
NC-CDC-LC0096266_2021-07-10_AY.100
NC-CDC-LC0148466_2021-07-30_AY.100
OL996197_NC-CDC-ASC210530761_2021-12-08_AY.100
OL883168_NC-CDC-LC0413549_2021-12-01_AY.100
MN908947 (Wuhan-Hu-1/2019)
0.2

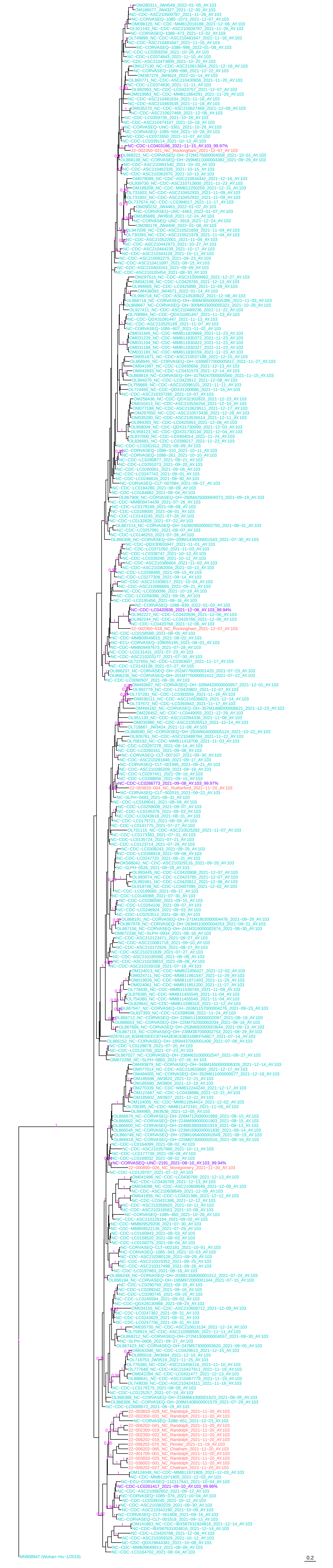

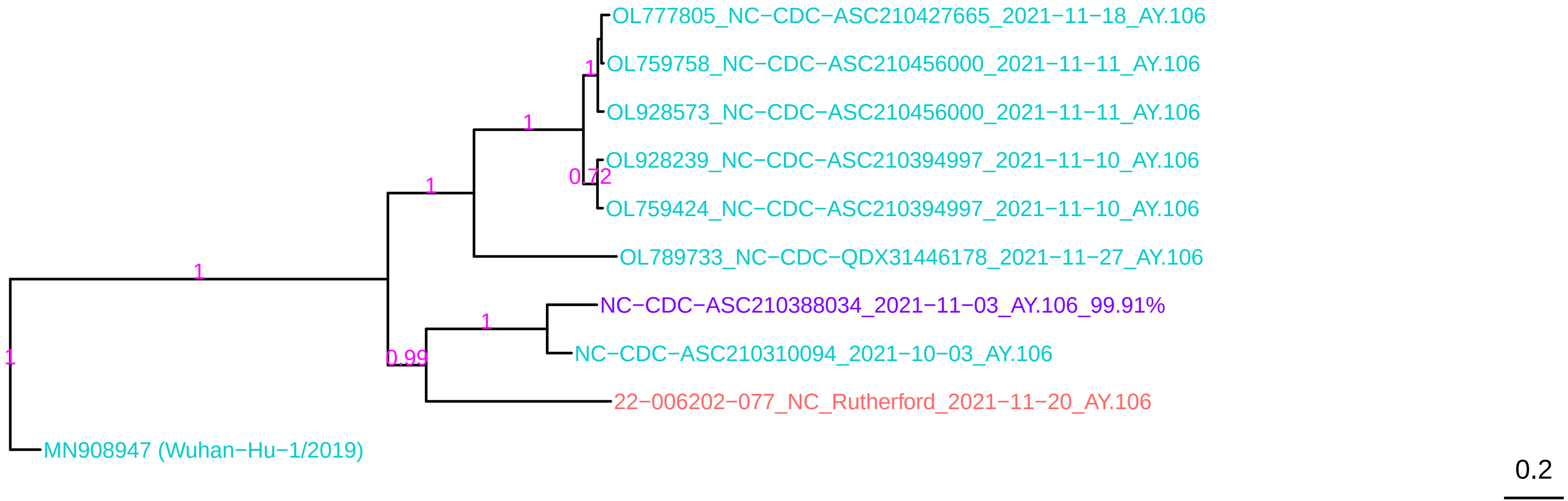

OL777805_NC−CDC−ASC210427665_2021−11−18_AY.106
OL759758_NC−CDC−ASC210456000_2021−11−11_AY.106
OL928573_NC−CDC−ASC210456000_2021−11−11_AY.106
OL928239_NC−CDC−ASC210394997_2021−11−10_AY.106
OL759424_NC−CDC−ASC210394997_2021−11−10_AY.106
OL789733_NC−CDC−QDX31446178_2021−11−27_AY.106
NC−CDC−ASC210388034_2021−11−03_AY.106_99.91%
NC−CDC−ASC210310094_2021−10−03_AY.106
22−006202−077_NC_Rutherford_2021−11−20_AY.106
MN908947 (Wuhan−Hu−1/2019)
1
1
1
0.72
1
1
1
0.99
1
0.2

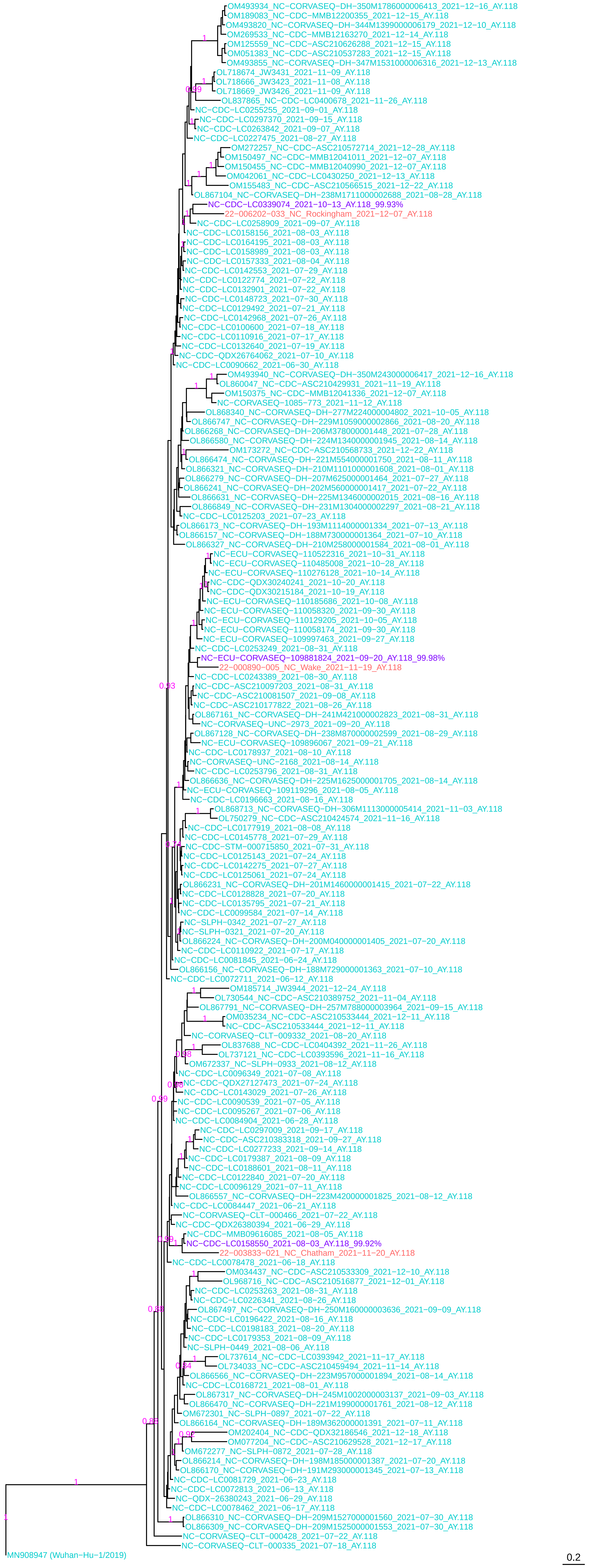
OM493934_NC-CORVASEQ-DH-350M1786000006413_2021-12-16_AY.118
OM189083_NC-CDC-MMB12200355_2021-12-15_AY.118
OM493820_NC-CORVASEQ-DH-344M1399000006179_2021-12-10_AY.118
OM269533_NC-CDC-MMB12163270_2021-12-14_AY.118
OM125559_NC-CDC-ASC210626288_2021-12-15_AY.118
OM051383_NC-CDC-ASC210537283_2021-12-15_AY.118
OM493855_NC-CORVASEQ-DH-347M1531000006316_2021-12-13_AY.118
OL718674_JW3431_2021-11-09_AY.118
OL718666_JW3423_2021-11-08_AY.118
OL718669_JW3426_2021-11-09_AY.118
OL837865_NC-CDC-LC0400678_2021-11-26_AY.118
NC-CDC-LC0255255_2021-09-01_AY.118
NC-CDC-LC0297370_2021-09-15_AY.118
NC-CDC-LC0263842_2021-09-07_AY.118
NC-CDC-LC0227475_2021-08-27_AY.118
OM272257_NC-CDC-ASC210572714_2021-12-28_AY.118
OM150497_NC-CDC-MMB12041011_2021-12-07_AY.118
OM150455_NC-CDC-MMB12040990_2021-12-07_AY.118
OM042061_NC-CDC-LC0430250_2021-12-13_AY.118
OM155483_NC-CDC-ASC210566515_2021-12-22_AY.118
OL867104_NC-CORVASEQ-DH-238M1711000002688_2021-08-28_AY.118
NC-CDC-LC0339074_2021-10-13_AY.118_99.93%
22-006202-033_NC_Rockingham_2021-12-07_AY.118
NC-CDC-LC0258909_2021-09-07_AY.118
NC-CDC-LC0158156_2021-08-03_AY.118
NC-CDC-LC0164195_2021-08-03_AY.118
NC-CDC-LC0158989_2021-08-03_AY.118
NC-CDC-LC0157333_2021-08-04_AY.118
NC-CDC-LC0142553_2021-07-29_AY.118
NC-CDC-LC0122774_2021-07-22_AY.118
NC-CDC-LC0132901_2021-07-22_AY.118
NC-CDC-LC0148723_2021-07-30_AY.118
NC-CDC-LC0129492_2021-07-21_AY.118
NC-CDC-LC0142968_2021-07-26_AY.118
NC-CDC-LC0100600_2021-07-18_AY.118
NC-CDC-LC0110916_2021-07-17_AY.118
NC-CDC-LC0132640_2021-07-19_AY.118
NC-CDC-QDX26764062_2021-07-10_AY.118
NC-CDC-LC0090662_2021-06-30_AY.118
OM493940_NC-CORVASEQ-DH-350M243000006417_2021-12-16_AY.118
OL860047_NC-CDC-ASC210429931_2021-11-19_AY.118
OM150375_NC-CDC-MMB12041336_2021-12-07_AY.118
NC-CORVASEQ-1085-773_2021-11-12_AY.118
OL868340_NC-CORVASEQ-DH-277M224000004802_2021-10-05_AY.118
OL866747_NC-CORVASEQ-DH-229M1059000002866_2021-08-20_AY.118
OL866268_NC-CORVASEQ-DH-206M378000001448_2021-07-28_AY.118
OL866580_NC-CORVASEQ-DH-224M1340000001945_2021-08-14_AY.118
OM173272_NC-CDC-ASC210568733_2021-12-22_AY.118
OL866474_NC-CORVASEQ-DH-221M554000001750_2021-08-11_AY.118
OL866321_NC-CORVASEQ-DH-210M1101000001608_2021-08-01_AY.118
OL866279_NC-CORVASEQ-DH-207M625000001464_2021-07-27_AY.118
OL866241_NC-CORVASEQ-DH-202M560000001417_2021-07-22_AY.118
OL866631_NC-CORVASEQ-DH-225M1346000002015_2021-08-16_AY.118
OL866849_NC-CORVASEQ-DH-231M1304000002297_2021-08-21_AY.118
NC-CDC-LC0125203_2021-07-23_AY.118
OL866173_NC-CORVASEQ-DH-193M1114000001334_2021-07-13_AY.118
OL866157_NC-CORVASEQ-DH-188M730000001364_2021-07-10_AY.118
OL866327_NC-CORVASEQ-DH-210M258000001584_2021-08-01_AY.118
NC-ECU-CORVASEQ-110522316_2021-10-31_AY.118
NC-ECU-CORVASEQ-110485008_2021-10-28_AY.118
NC-ECU-CORVASEQ-110276128_2021-10-14_AY.118
NC-CDC-QDX30240241_2021-10-20_AY.118
NC-CDC-QDX30215184_2021-10-19_AY.118
NC-ECU-CORVASEQ-110185686_2021-10-08_AY.118
NC-ECU-CORVASEQ-110058320_2021-09-30_AY.118
NC-ECU-CORVASEQ-110129205_2021-10-05_AY.118
NC-ECU-CORVASEQ-110058174_2021-09-30_AY.118
NC-ECU-CORVASEQ-109997463_2021-09-27_AY.118
NC-CDC-LC0253249_2021-08-31_AY.118
NC-ECU-CORVASEQ-109881824_2021-09-20_AY.118_99.98%
22-000890-005_NC_Wake_2021-11-19_AY.118
NC-CDC-LC0243389_2021-08-30_AY.118
NC-CDC-ASC210097203_2021-08-31_AY.118
NC-CDC-ASC210081507_2021-09-08_AY.118
NC-CDC-ASC210177822_2021-08-26_AY.118
OL867161_NC-CORVASEQ-DH-241M421000002823_2021-08-31_AY.118
NC-CORVASEQ-UNC-2973_2021-09-20_AY.118
OL867128_NC-CORVASEQ-DH-238M870000002599_2021-08-29_AY.118
NC-ECU-CORVASEQ-109896067_2021-09-21_AY.118
NC-CDC-LC0178937_2021-08-10_AY.118
NC-CORVASEQ-UNC-2168_2021-08-14_AY.118
NC-CDC-LC0253796_2021-08-31_AY.118
OL866636_NC-CORVASEQ-DH-225M1625000001705_2021-08-14_AY.118
NC-ECU-CORVASEQ-109119296_2021-08-05_AY.118
NC-CDC-LC0196663_2021-08-16_AY.118
OL868713_NC-CORVASEQ-DH-306M1113000005414_2021-11-03_AY.118
OL750279_NC-CDC-ASC210424574_2021-11-16_AY.118
NC-CDC-LC0177919_2021-08-08_AY.118
NC-CDC-LC0145778_2021-07-29_AY.118
NC-CDC-STM-000715850_2021-07-31_AY.118
NC-CDC-LC0125143_2021-07-24_AY.118
NC-CDC-LC0142275_2021-07-27_AY.118
NC-CDC-LC0125061_2021-07-24_AY.118
OL866231_NC-CORVASEQ-DH-201M1460000001415_2021-07-22_AY.118
NC-CDC-LC0128828_2021-07-20_AY.118
NC-CDC-LC0135795_2021-07-21_AY.118
NC-CDC-LC0099584_2021-07-14_AY.118
NC-SLPH-0342_2021-07-27_AY.118
NC-SLPH-0321_2021-07-20_AY.118
OL866224_NC-CORVASEQ-DH-200M040000001405_2021-07-20_AY.118
NC-CDC-LC0110922_2021-07-17_AY.118
NC-CDC-LC0081845_2021-06-24_AY.118
OL866156_NC-CORVASEQ-DH-188M729000001363_2021-07-10_AY.118
NC-CDC-LC0072711_2021-06-12_AY.118
OM185714_JW3944_2021-12-24_AY.118
OL730544_NC-CDC-ASC210389752_2021-11-04_AY.118
OL867791_NC-CORVASEQ-DH-257M788000003964_2021-09-15_AY.118
OM035234_NC-CDC-ASC210533444_2021-12-11_AY.118
NC-CDC-ASC210533444_2021-12-11_AY.118
NC-CORVASEQ-CLT-009332_2021-08-20_AY.118
OL837688_NC-CDC-LC0404392_2021-11-26_AY.118
OL737121_NC-CDC-LC0393596_2021-11-16_AY.118
OM672337_NC-SLPH-0933_2021-08-12_AY.118
NC-CDC-LC0096349_2021-07-08_AY.118
NC-CDC-QDX27127473_2021-07-24_AY.118
NC-CDC-LC0143029_2021-07-26_AY.118
NC-CDC-LC0090539_2021-07-05_AY.118
NC-CDC-LC0095267_2021-07-06_AY.118
NC-CDC-LC0084904_2021-06-28_AY.118
NC-CDC-LC0297009_2021-09-17_AY.118
NC-CDC-ASC210383318_2021-09-27_AY.118
NC-CDC-LC0277233_2021-09-14_AY.118
NC-CDC-LC0179387_2021-08-09_AY.118
NC-CDC-LC0188601_2021-08-11_AY.118
NC-CDC-LC0122840_2021-07-20_AY.118
NC-CDC-LC0096129_2021-07-11_AY.118
OL866557_NC-CORVASEQ-DH-223M420000001825_2021-08-12_AY.118
NC-CDC-LC0084447_2021-06-21_AY.118
NC-CORVASEQ-CLT-000466_2021-07-22_AY.118
NC-CDC-QDX26380394_2021-06-29_AY.118
NC-CDC-MMB09616085_2021-08-05_AY.118
NC-CDC-LC0158550_2021-08-03_AY.118_99.92%
22-003833-021_NC_Chatham_2021-11-20_AY.118
NC-CDC-LC0078478_2021-06-18_AY.118
OM034437_NC-CDC-ASC210533309_2021-12-10_AY.118
OL968716_NC-CDC-ASC210516877_2021-12-01_AY.118
NC-CDC-LC0253263_2021-08-31_AY.118
NC-CDC-LC0226341_2021-08-26_AY.118
OL867497_NC-CORVASEQ-DH-250M160000003636_2021-09-09_AY.118
NC-CDC-LC0196422_2021-08-16_AY.118
NC-CDC-LC0198183_2021-08-20_AY.118
NC-CDC-LC0179353_2021-08-09_AY.118
NC-SLPH-0449_2021-08-06_AY.118
OL737614_NC-CDC-LC0393942_2021-11-17_AY.118
OL734033_NC-CDC-ASC210459494_2021-11-14_AY.118
OL866566_NC-CORVASEQ-DH-223M957000001894_2021-08-14_AY.118
NC-CDC-LC0168721_2021-08-01_AY.118
OL867317_NC-CORVASEQ-DH-245M1002000003137_2021-09-03_AY.118
OL866470_NC-CORVASEQ-DH-221M199000001761_2021-08-12_AY.118
OM672301_NC-SLPH-0897_2021-07-22_AY.118
OL866164_NC-CORVASEQ-DH-189M362000001391_2021-07-11_AY.118
OM202404_NC-CDC-QDX32186546_2021-12-18_AY.118
OM077204_NC-CDC-ASC210629528_2021-12-17_AY.118
OM672277_NC-SLPH-0872_2021-07-28_AY.118
OL866214_NC-CORVASEQ-DH-198M185000001387_2021-07-20_AY.118
OL866170_NC-CORVASEQ-DH-191M293000001345_2021-07-13_AY.118
NC-CDC-LC0081729_2021-06-23_AY.118
NC-CDC-LC0072813_2021-06-13_AY.118
NC-QDX-26380243_2021-06-29_AY.118
NC-CDC-LC0078462_2021-06-17_AY.118
OL866310_NC-CORVASEQ-DH-209M1527000001560_2021-07-30_AY.118
OL866309_NC-CORVASEQ-DH-209M1525000001553_2021-07-30_AY.118
NC-CORVASEQ-CLT-000428_2021-07-22_AY.118
NC-CORVASEQ-CLT-000335_2021-07-18_AY.118
MN908947 (Wuhan-Hu-1/2019)
0.2

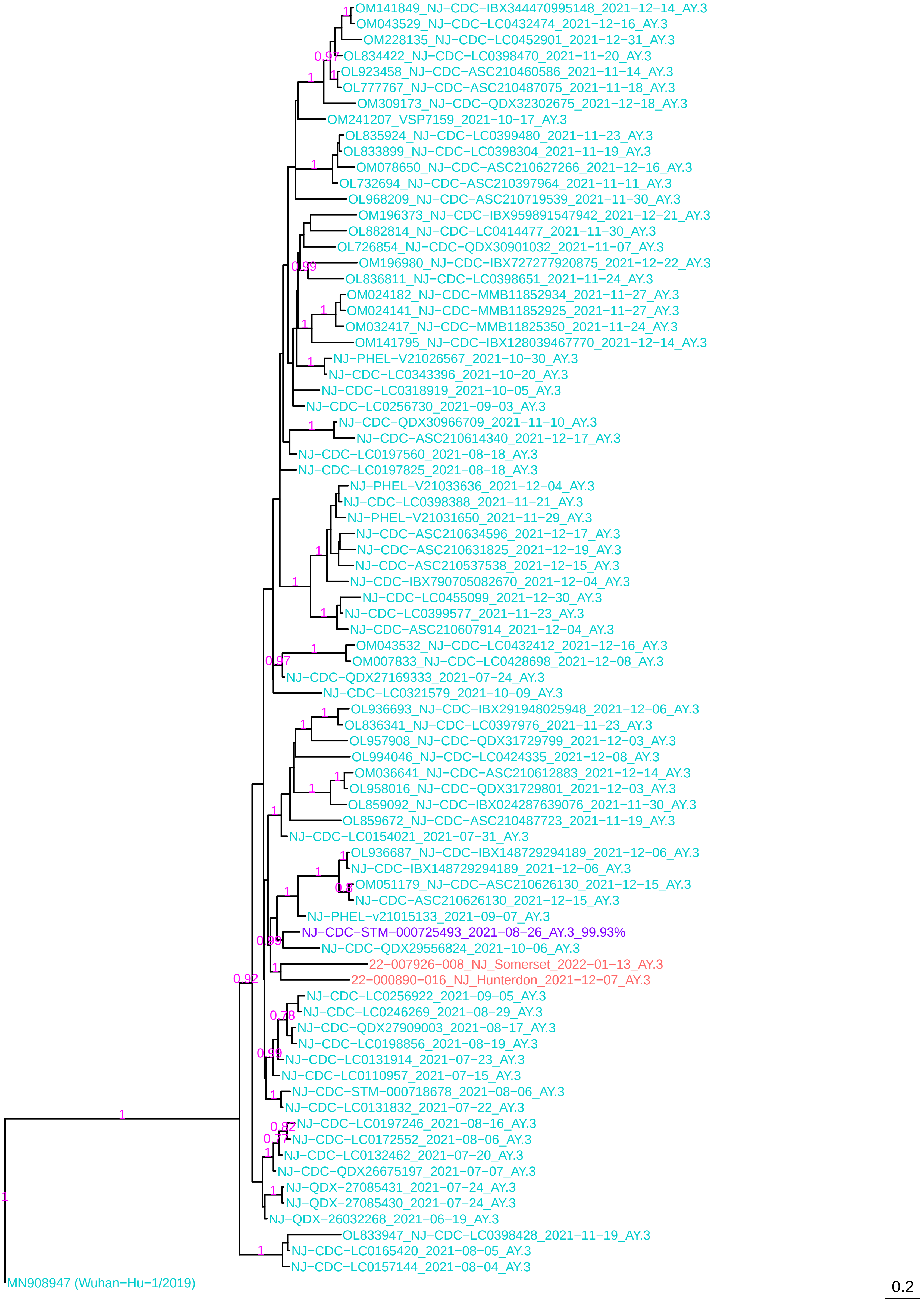
OM141849_NJ−CDC−IBX344470995148_2021−12−14_AY.3
OM043529_NJ−CDC−LC0432474_2021−12−16_AY.3
OM228135_NJ−CDC−LC0452901_2021−12−31_AY.3
OL834422_NJ−CDC−LC0398470_2021−11−20_AY.3
OL923458_NJ−CDC−ASC210460586_2021−11−14_AY.3
OL777767_NJ−CDC−ASC210487075_2021−11−18_AY.3
OM309173_NJ−CDC−QDX32302675_2021−12−18_AY.3
OM241207_VSP7159_2021−10−17_AY.3
OL835924_NJ−CDC−LC0399480_2021−11−23_AY.3
OL833899_NJ−CDC−LC0398304_2021−11−19_AY.3
OM078650_NJ−CDC−ASC210627266_2021−12−16_AY.3
OL732694_NJ−CDC−ASC210397964_2021−11−11_AY.3
OL968209_NJ−CDC−ASC210719539_2021−11−30_AY.3
OM196373_NJ−CDC−IBX959891547942_2021−12−21_AY.3
OL882814_NJ−CDC−LC0414477_2021−11−30_AY.3
OL726854_NJ−CDC−QDX30901032_2021−11−07_AY.3
OM196980_NJ−CDC−IBX727277920875_2021−12−22_AY.3
OL836811_NJ−CDC−LC0398651_2021−11−24_AY.3
OM024182_NJ−CDC−MMB11852934_2021−11−27_AY.3
OM024141_NJ−CDC−MMB11852925_2021−11−27_AY.3
OM032417_NJ−CDC−MMB11825350_2021−11−24_AY.3
OM141795_NJ−CDC−IBX128039467770_2021−12−14_AY.3
NJ−PHEL−V21026567_2021−10−30_AY.3
NJ−CDC−LC0343396_2021−10−20_AY.3
NJ−CDC−LC0318919_2021−10−05_AY.3
NJ−CDC−LC0256730_2021−09−03_AY.3
NJ−CDC−QDX30966709_2021−11−10_AY.3
NJ−CDC−ASC210614340_2021−12−17_AY.3
NJ−CDC−LC0197560_2021−08−18_AY.3
NJ−CDC−LC0197825_2021−08−18_AY.3
NJ−PHEL−V21033636_2021−12−04_AY.3
NJ−CDC−LC0398388_2021−11−21_AY.3
NJ−PHEL−V21031650_2021−11−29_AY.3
NJ−CDC−ASC210634596_2021−12−17_AY.3
NJ−CDC−ASC210631825_2021−12−19_AY.3
NJ−CDC−ASC210537538_2021−12−15_AY.3
NJ−CDC−IBX790705082670_2021−12−04_AY.3
NJ−CDC−LC0455099_2021−12−30_AY.3
NJ−CDC−LC0399577_2021−11−23_AY.3
NJ−CDC−ASC210607914_2021−12−04_AY.3
OM043532_NJ−CDC−LC0432412_2021−12−16_AY.3
OM007833_NJ−CDC−LC0428698_2021−12−08_AY.3
NJ−CDC−QDX27169333_2021−07−24_AY.3
NJ−CDC−LC0321579_2021−10−09_AY.3
OL936693_NJ−CDC−IBX291948025948_2021−12−06_AY.3
OL836341_NJ−CDC−LC0397976_2021−11−23_AY.3
OL957908_NJ−CDC−QDX31729799_2021−12−03_AY.3
OL994046_NJ−CDC−LC0424335_2021−12−08_AY.3
OM036641_NJ−CDC−ASC210612883_2021−12−14_AY.3
OL958016_NJ−CDC−QDX31729801_2021−12−03_AY.3
OL859092_NJ−CDC−IBX024287639076_2021−11−30_AY.3
OL859672_NJ−CDC−ASC210487723_2021−11−19_AY.3
NJ−CDC−LC0154021_2021−07−31_AY.3
OL936687_NJ−CDC−IBX148729294189_2021−12−06_AY.3
NJ−CDC−IBX148729294189_2021−12−06_AY.3
OM051179_NJ−CDC−ASC210626130_2021−12−15_AY.3
NJ−CDC−ASC210626130_2021−12−15_AY.3
NJ−PHEL−v21015133_2021−09−07_AY.3
NJ−CDC−STM−000725493_2021−08−26_AY.3_99.93%
NJ−CDC−QDX29556824_2021−10−06_AY.3
22−007926−008_NJ_Somerset_2022−01−13_AY.3
22−000890−016_NJ_Hunterdon_2021−12−07_AY.3
NJ−CDC−LC0256922_2021−09−05_AY.3
NJ−CDC−LC0246269_2021−08−29_AY.3
NJ−CDC−QDX27909003_2021−08−17_AY.3
NJ−CDC−LC0198856_2021−08−19_AY.3
NJ−CDC−LC0131914_2021−07−23_AY.3
NJ−CDC−LC0110957_2021−07−15_AY.3
NJ−CDC−STM−000718678_2021−08−06_AY.3
NJ−CDC−LC0131832_2021−07−22_AY.3
NJ−CDC−LC0197246_2021−08−16_AY.3
NJ−CDC−LC0172552_2021−08−06_AY.3
NJ−CDC−LC0132462_2021−07−20_AY.3
NJ−CDC−QDX26675197_2021−07−07_AY.3
NJ−QDX−27085431_2021−07−24_AY.3
NJ−QDX−27085430_2021−07−24_AY.3
NJ−QDX−26032268_2021−06−19_AY.3
OL833947_NJ−CDC−LC0398428_2021−11−19_AY.3
NJ−CDC−LC0165420_2021−08−05_AY.3
NJ−CDC−LC0157144_2021−08−04_AY.3
MN908947 (Wuhan−Hu−1/2019)
0.2

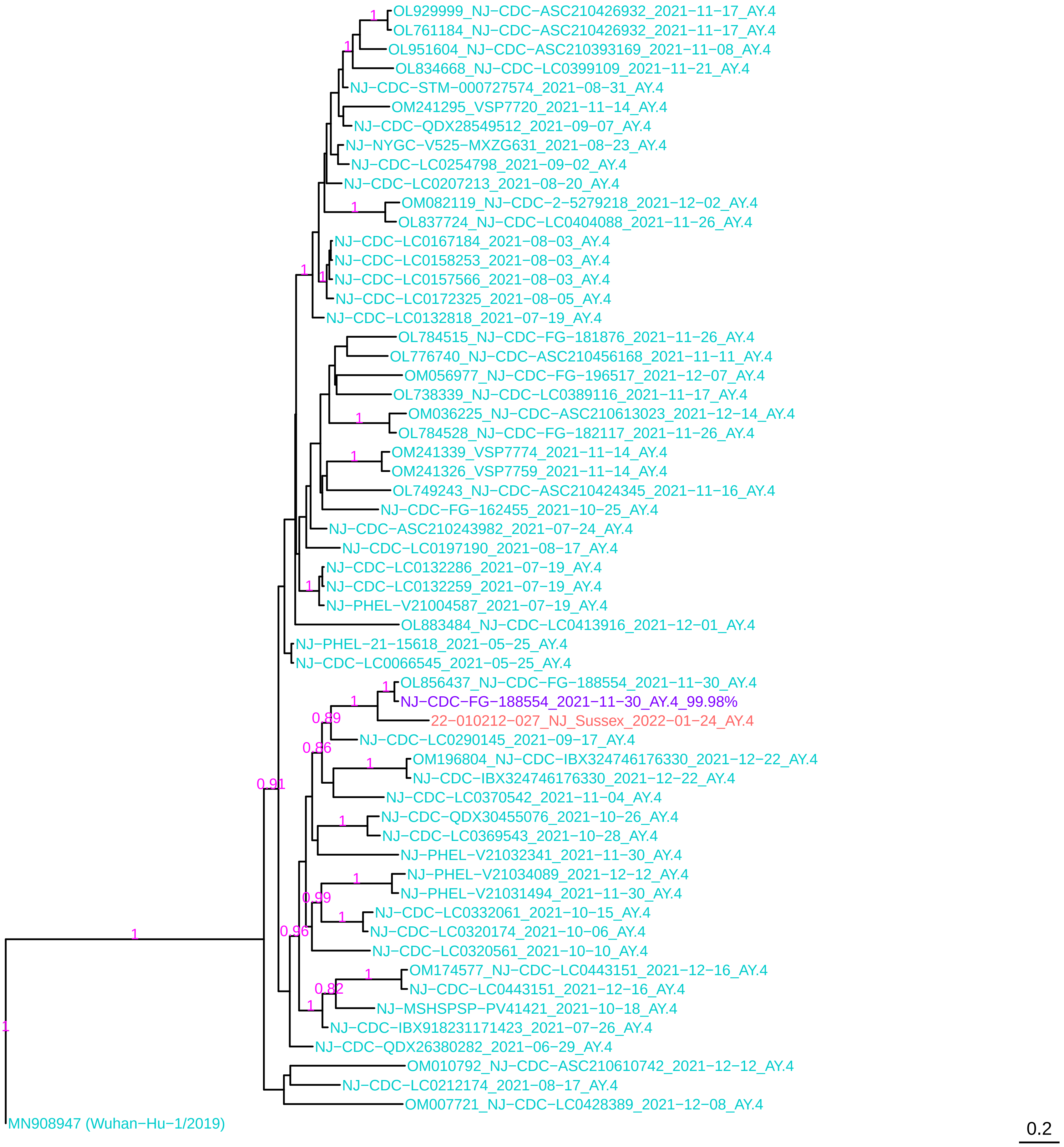

OL929999_NJ−CDC−ASC210426932_2021−11−17_AY.4
OL761184_NJ−CDC−ASC210426932_2021−11−17_AY.4
OL951604_NJ−CDC−ASC210393169_2021−11−08_AY.4
OL834668_NJ−CDC−LC0399109_2021−11−21_AY.4
NJ−CDC−STM−000727574_2021−08−31_AY.4
OM241295_VSP7720_2021−11−14_AY.4
NJ−CDC−QDX28549512_2021−09−07_AY.4
NJ−NYGC−V525−MXZG631_2021−08−23_AY.4
NJ−CDC−LC0254798_2021−09−02_AY.4
NJ−CDC−LC0207213_2021−08−20_AY.4
OM082119_NJ−CDC−2−5279218_2021−12−02_AY.4
OL837724_NJ−CDC−LC0404088_2021−11−26_AY.4
NJ−CDC−LC0167184_2021−08−03_AY.4
NJ−CDC−LC0158253_2021−08−03_AY.4
NJ−CDC−LC0157566_2021−08−03_AY.4
NJ−CDC−LC0172325_2021−08−05_AY.4
NJ−CDC−LC0132818_2021−07−19_AY.4
OL784515_NJ−CDC−FG−181876_2021−11−26_AY.4
OL776740_NJ−CDC−ASC210456168_2021−11−11_AY.4
OM056977_NJ−CDC−FG−196517_2021−12−07_AY.4
OL738339_NJ−CDC−LC0389116_2021−11−17_AY.4
OM036225_NJ−CDC−ASC210613023_2021−12−14_AY.4
OL784528_NJ−CDC−FG−182117_2021−11−26_AY.4
OM241339_VSP7774_2021−11−14_AY.4
OM241326_VSP7759_2021−11−14_AY.4
OL749243_NJ−CDC−ASC210424345_2021−11−16_AY.4
NJ−CDC−FG−162455_2021−10−25_AY.4
NJ−CDC−ASC210243982_2021−07−24_AY.4
NJ−CDC−LC0197190_2021−08−17_AY.4
NJ−CDC−LC0132286_2021−07−19_AY.4
NJ−CDC−LC0132259_2021−07−19_AY.4
NJ−PHEL−V21004587_2021−07−19_AY.4
OL883484_NJ−CDC−LC0413916_2021−12−01_AY.4
NJ−PHEL−21−15618_2021−05−25_AY.4
NJ−CDC−LC0066545_2021−05−25_AY.4
OL856437_NJ−CDC−FG−188554_2021−11−30_AY.4
NJ−CDC−FG−188554_2021−11−30_AY.4_99.98%
22−010212−027_NJ_Sussex_2022−01−24_AY.4
NJ−CDC−LC0290145_2021−09−17_AY.4
OM196804_NJ−CDC−IBX324746176330_2021−12−22_AY.4
NJ−CDC−IBX324746176330_2021−12−22_AY.4
NJ−CDC−LC0370542_2021−11−04_AY.4
NJ−CDC−QDX30455076_2021−10−26_AY.4
NJ−CDC−LC0369543_2021−10−28_AY.4
NJ−PHEL−V21032341_2021−11−30_AY.4
NJ−PHEL−V21034089_2021−12−12_AY.4
NJ−PHEL−V21031494_2021−11−30_AY.4
NJ−CDC−LC0332061_2021−10−15_AY.4
NJ−CDC−LC0320174_2021−10−06_AY.4
NJ−CDC−LC0320561_2021−10−10_AY.4
OM174577_NJ−CDC−LC0443151_2021−12−16_AY.4
NJ−CDC−LC0443151_2021−12−16_AY.4
NJ−MSHSPSP−PV41421_2021−10−18_AY.4
NJ−CDC−IBX918231171423_2021−07−26_AY.4
NJ−CDC−QDX26380282_2021−06−29_AY.4
OM010792_NJ−CDC−ASC210610742_2021−12−12_AY.4
NJ−CDC−LC0212174_2021−08−17_AY.4
OM007721_NJ−CDC−LC0428389_2021−12−08_AY.4
MN908947 (Wuhan−Hu−1/2019)
0.89
0.86
0.91
0.99
0.96
0.82
0.2

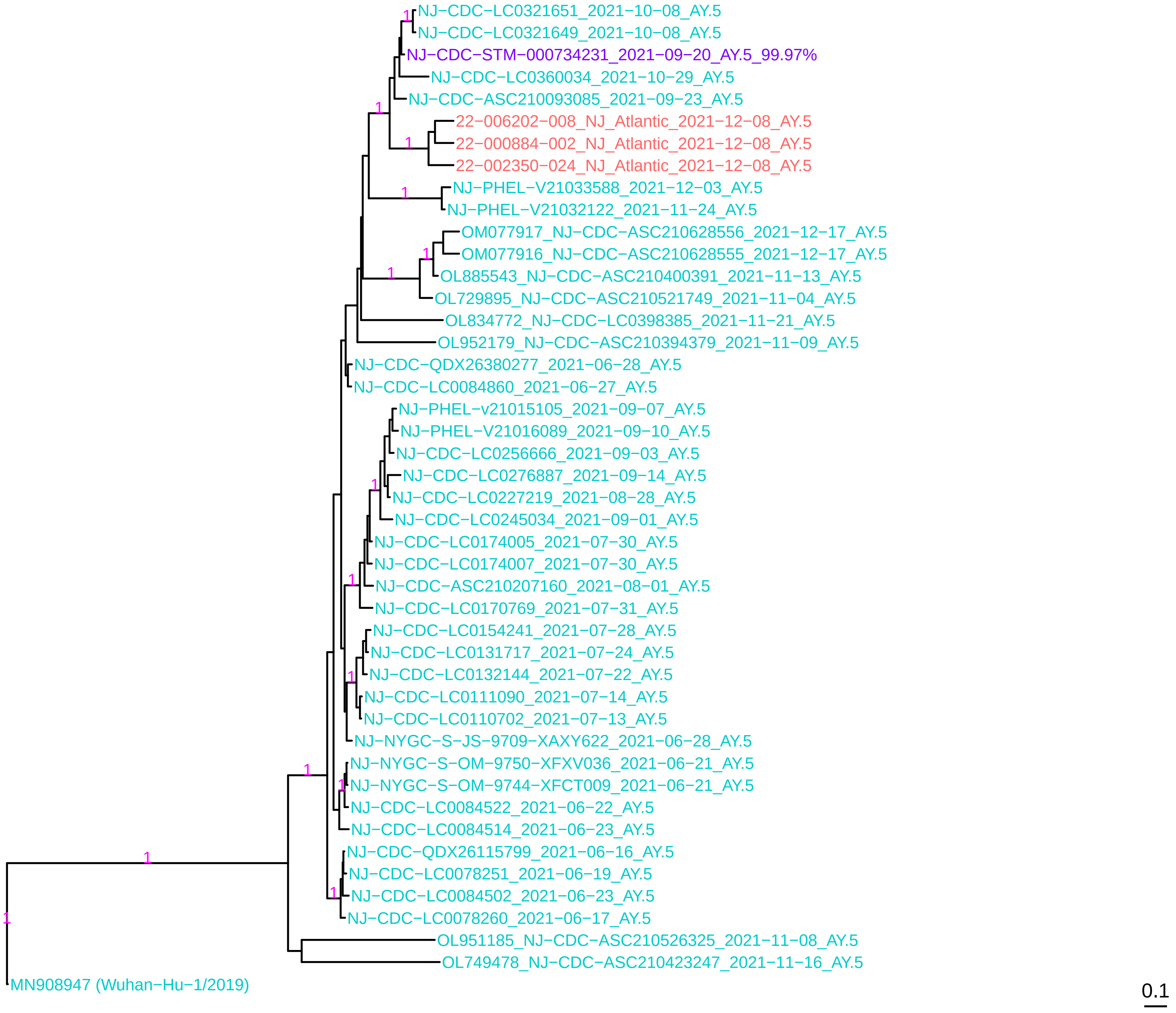
NJ−CDC−LC0321651_2021−10−08_AY.5
NJ−CDC−LC0321649_2021−10−08_AY.5
NJ−CDC−STM−000734231_2021−09−20_AY.5_99.97%
NJ−CDC−LC0360034_2021−10−29_AY.5
NJ−CDC−ASC210093085_2021−09−23_AY.5
22−006202−008_NJ_Atlantic_2021−12−08_AY.5
22−000884−002_NJ_Atlantic_2021−12−08_AY.5
22−002350−024_NJ_Atlantic_2021−12−08_AY.5
NJ−PHEL−V21033588_2021−12−03_AY.5
NJ−PHEL−V21032122_2021−11−24_AY.5
OM077917_NJ−CDC−ASC210628556_2021−12−17_AY.5
OM077916_NJ−CDC−ASC210628555_2021−12−17_AY.5
OL885543_NJ−CDC−ASC210400391_2021−11−13_AY.5
OL729895_NJ−CDC−ASC210521749_2021−11−04_AY.5
OL834772_NJ−CDC−LC0398385_2021−11−21_AY.5
OL952179_NJ−CDC−ASC210394379_2021−11−09_AY.5
NJ−CDC−QDX26380277_2021−06−28_AY.5
NJ−CDC−LC0084860_2021−06−27_AY.5
NJ−PHEL−v21015105_2021−09−07_AY.5
NJ−PHEL−V21016089_2021−09−10_AY.5
NJ−CDC−LC0256666_2021−09−03_AY.5
NJ−CDC−LC0276887_2021−09−14_AY.5
NJ−CDC−LC0227219_2021−08−28_AY.5
NJ−CDC−LC0245034_2021−09−01_AY.5
NJ−CDC−LC0174005_2021−07−30_AY.5
NJ−CDC−LC0174007_2021−07−30_AY.5
NJ−CDC−ASC210207160_2021−08−01_AY.5
NJ−CDC−LC0170769_2021−07−31_AY.5
NJ−CDC−LC0154241_2021−07−28_AY.5
NJ−CDC−LC0131717_2021−07−24_AY.5
NJ−CDC−LC0132144_2021−07−22_AY.5
NJ−CDC−LC0111090_2021−07−14_AY.5
NJ−CDC−LC0110702_2021−07−13_AY.5
NJ−NYGC−S−JS−9709−XAXY622_2021−06−28_AY.5
NJ−NYGC−S−OM−9750−XFXV036_2021−06−21_AY.5
NJ−NYGC−S−OM−9744−XFCT009_2021−06−21_AY.5
NJ−CDC−LC0084522_2021−06−22_AY.5
NJ−CDC−LC0084514_2021−06−23_AY.5
NJ−CDC−QDX26115799_2021−06−16_AY.5
NJ−CDC−LC0078251_2021−06−19_AY.5
NJ−CDC−LC0084502_2021−06−23_AY.5
NJ−CDC−LC0078260_2021−06−17_AY.5
OL951185_NJ−CDC−ASC210526325_2021−11−08_AY.5
OL749478_NJ−CDC−ASC210423247_2021−11−16_AY.5
MN908947 (Wuhan−Hu−1/2019)
0.1

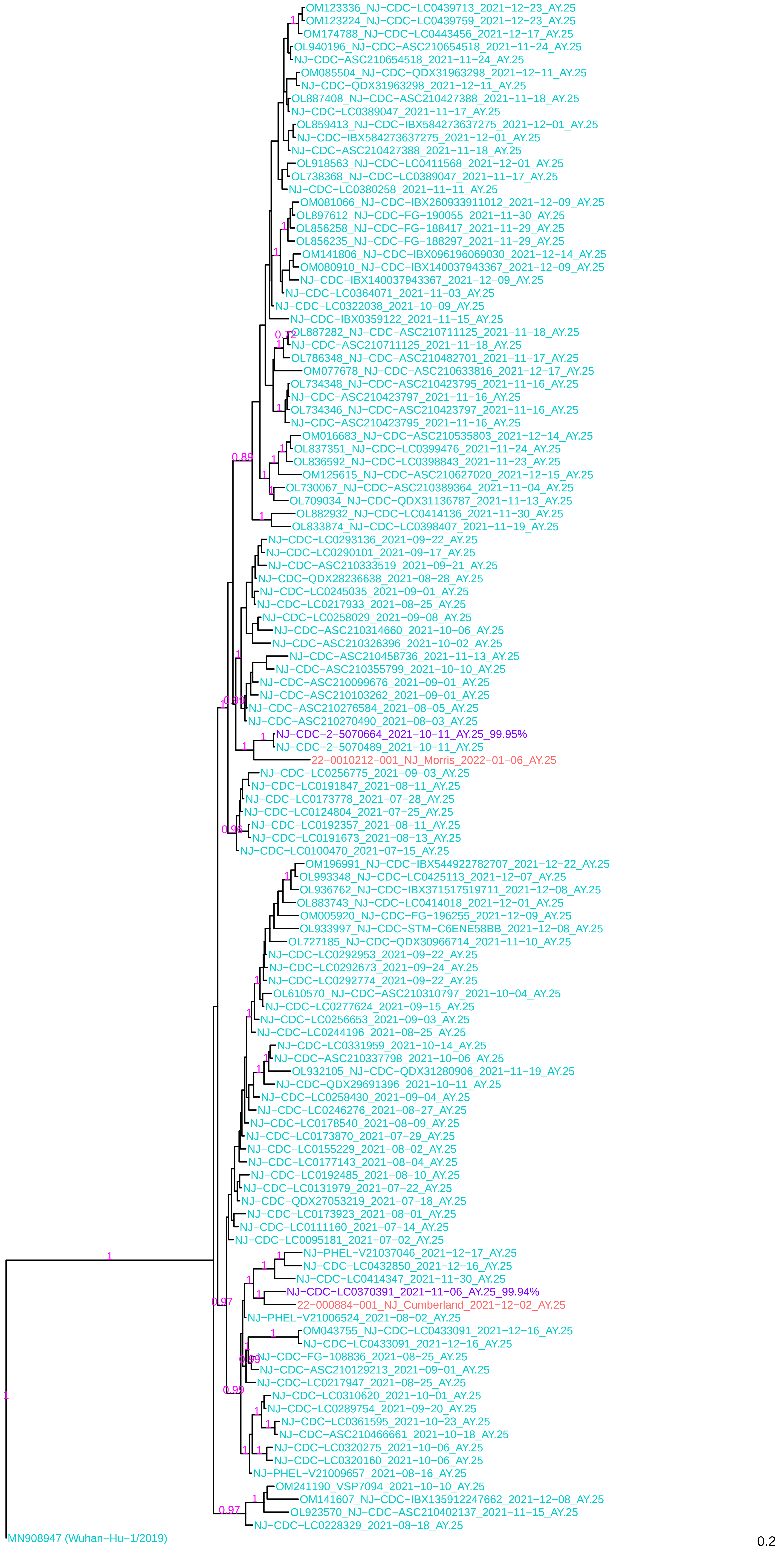
OM123336_NJ-CDC-LC0439713_2021-12-23_AY.25
OM123224_NJ-CDC-LC0439759_2021-12-23_AY.25
OM174788_NJ-CDC-LC0443456_2021-12-17_AY.25
OL940196_NJ-CDC-ASC210654518_2021-11-24_AY.25
NJ-CDC-ASC210654518_2021-11-24_AY.25
OM085504_NJ-CDC-QDX31963298_2021-12-11_AY.25
NJ-CDC-QDX31963298_2021-12-11_AY.25
OL887408_NJ-CDC-ASC210427388_2021-11-18_AY.25
NJ-CDC-LC0389047_2021-11-17_AY.25
OL859413_NJ-CDC-IBX584273637275_2021-12-01_AY.25
NJ-CDC-IBX584273637275_2021-12-01_AY.25
NJ-CDC-ASC210427388_2021-11-18_AY.25
OL918563_NJ-CDC-LC0411568_2021-12-01_AY.25
OL738368_NJ-CDC-LC0389047_2021-11-17_AY.25
NJ-CDC-LC0380258_2021-11-11_AY.25
OM081066_NJ-CDC-IBX260933911012_2021-12-09_AY.25
OL897612_NJ-CDC-FG-190055_2021-11-30_AY.25
OL856258_NJ-CDC-FG-188417_2021-11-29_AY.25
OL856235_NJ-CDC-FG-188297_2021-11-29_AY.25
OM141806_NJ-CDC-IBX096196069030_2021-12-14_AY.25
OM080910_NJ-CDC-IBX140037943367_2021-12-09_AY.25
NJ-CDC-IBX140037943367_2021-12-09_AY.25
NJ-CDC-LC0364071_2021-11-03_AY.25
NJ-CDC-LC0322038_2021-10-09_AY.25
NJ-CDC-IBX0359122_2021-11-15_AY.25
OL887282_NJ-CDC-ASC210711125_2021-11-18_AY.25
NJ-CDC-ASC210711125_2021-11-18_AY.25
OL786348_NJ-CDC-ASC210482701_2021-11-17_AY.25
OM077678_NJ-CDC-ASC210633816_2021-12-17_AY.25
OL734348_NJ-CDC-ASC210423795_2021-11-16_AY.25
NJ-CDC-ASC210423797_2021-11-16_AY.25
OL734346_NJ-CDC-ASC210423797_2021-11-16_AY.25
NJ-CDC-ASC210423795_2021-11-16_AY.25
OM016683_NJ-CDC-ASC210535803_2021-12-14_AY.25
OL837351_NJ-CDC-LC0399476_2021-11-24_AY.25
OL836592_NJ-CDC-LC0398843_2021-11-23_AY.25
OM125615_NJ-CDC-ASC210627020_2021-12-15_AY.25
OL730067_NJ-CDC-ASC210389364_2021-11-04_AY.25
OL709034_NJ-CDC-QDX31136787_2021-11-13_AY.25
OL882932_NJ-CDC-LC0414136_2021-11-30_AY.25
OL833874_NJ-CDC-LC0398407_2021-11-19_AY.25
NJ-CDC-LC0293136_2021-09-22_AY.25
NJ-CDC-LC0290101_2021-09-17_AY.25
NJ-CDC-ASC210333519_2021-09-21_AY.25
NJ-CDC-QDX28236638_2021-08-28_AY.25
NJ-CDC-LC0245035_2021-09-01_AY.25
NJ-CDC-LC0217933_2021-08-25_AY.25
NJ-CDC-LC0258029_2021-09-08_AY.25
NJ-CDC-ASC210314660_2021-10-06_AY.25
NJ-CDC-ASC210326396_2021-10-02_AY.25
NJ-CDC-ASC210458736_2021-11-13_AY.25
NJ-CDC-ASC210355799_2021-10-10_AY.25
NJ-CDC-ASC210099676_2021-09-01_AY.25
NJ-CDC-ASC210103262_2021-09-01_AY.25
NJ-CDC-ASC210276584_2021-08-05_AY.25
NJ-CDC-ASC210270490_2021-08-03_AY.25
NJ-CDC-2-5070664_2021-10-11_AY.25_99.95%
NJ-CDC-2-5070489_2021-10-11_AY.25
22-0010212-001_NJ_Morris_2022-01-06_AY.25
NJ-CDC-LC0256775_2021-09-03_AY.25
NJ-CDC-LC0191847_2021-08-11_AY.25
NJ-CDC-LC0173778_2021-07-28_AY.25
NJ-CDC-LC0124804_2021-07-25_AY.25
NJ-CDC-LC0192357_2021-08-11_AY.25
NJ-CDC-LC0191673_2021-08-13_AY.25
NJ-CDC-LC0100470_2021-07-15_AY.25
OM196991_NJ-CDC-IBX544922782707_2021-12-22_AY.25
OL993348_NJ-CDC-LC0425113_2021-12-07_AY.25
OL936762_NJ-CDC-IBX371517519711_2021-12-08_AY.25
OL883743_NJ-CDC-LC0414018_2021-12-01_AY.25
OM005920_NJ-CDC-FG-196255_2021-12-09_AY.25
OL933997_NJ-CDC-STM-C6ENE58BB_2021-12-08_AY.25
OL727185_NJ-CDC-QDX30966714_2021-11-10_AY.25
NJ-CDC-LC0292953_2021-09-22_AY.25
NJ-CDC-LC0292673_2021-09-24_AY.25
NJ-CDC-LC0292774_2021-09-22_AY.25
OL610570_NJ-CDC-ASC210310797_2021-10-04_AY.25
NJ-CDC-LC0277624_2021-09-15_AY.25
NJ-CDC-LC0256653_2021-09-03_AY.25
NJ-CDC-LC0244196_2021-08-25_AY.25
NJ-CDC-LC0331959_2021-10-14_AY.25
NJ-CDC-ASC210337798_2021-10-06_AY.25
OL932105_NJ-CDC-QDX31280906_2021-11-19_AY.25
NJ-CDC-QDX29691396_2021-10-11_AY.25
NJ-CDC-LC0258430_2021-09-04_AY.25
NJ-CDC-LC0246276_2021-08-27_AY.25
NJ-CDC-LC0178540_2021-08-09_AY.25
NJ-CDC-LC0173870_2021-07-29_AY.25
NJ-CDC-LC0155229_2021-08-02_AY.25
NJ-CDC-LC0177143_2021-08-04_AY.25
NJ-CDC-LC0192485_2021-08-10_AY.25
NJ-CDC-LC0131979_2021-07-22_AY.25
NJ-CDC-QDX27053219_2021-07-18_AY.25
NJ-CDC-LC0173923_2021-08-01_AY.25
NJ-CDC-LC0111160_2021-07-14_AY.25
NJ-CDC-LC0095181_2021-07-02_AY.25
NJ-PHEL-V21037046_2021-12-17_AY.25
NJ-CDC-LC0432850_2021-12-16_AY.25
NJ-CDC-LC0414347_2021-11-30_AY.25
NJ-CDC-LC0370391_2021-11-06_AY.25_99.94%
22-000884-001_NJ_Cumberland_2021-12-02_AY.25
NJ-PHEL-V21006524_2021-08-02_AY.25
OM043755_NJ-CDC-LC0433091_2021-12-16_AY.25
NJ-CDC-LC0433091_2021-12-16_AY.25
NJ-CDC-FG-108836_2021-08-25_AY.25
NJ-CDC-ASC210129213_2021-09-01_AY.25
NJ-CDC-LC0217947_2021-08-25_AY.25
NJ-CDC-LC0310620_2021-10-01_AY.25
NJ-CDC-LC0289754_2021-09-20_AY.25
NJ-CDC-LC0361595_2021-10-23_AY.25
NJ-CDC-ASC210466661_2021-10-18_AY.25
NJ-CDC-LC0320275_2021-10-06_AY.25
NJ-CDC-LC0320160_2021-10-06_AY.25
NJ-PHEL-V21009657_2021-08-16_AY.25
OM241190_VSP7094_2021-10-10_AY.25
OM141607_NJ-CDC-IBX135912247662_2021-12-08_AY.25
OL923570_NJ-CDC-ASC210402137_2021-11-15_AY.25
NJ-CDC-LC0228329_2021-08-18_AY.25
MN908947 (Wuhan-Hu-1/2019)
0.2

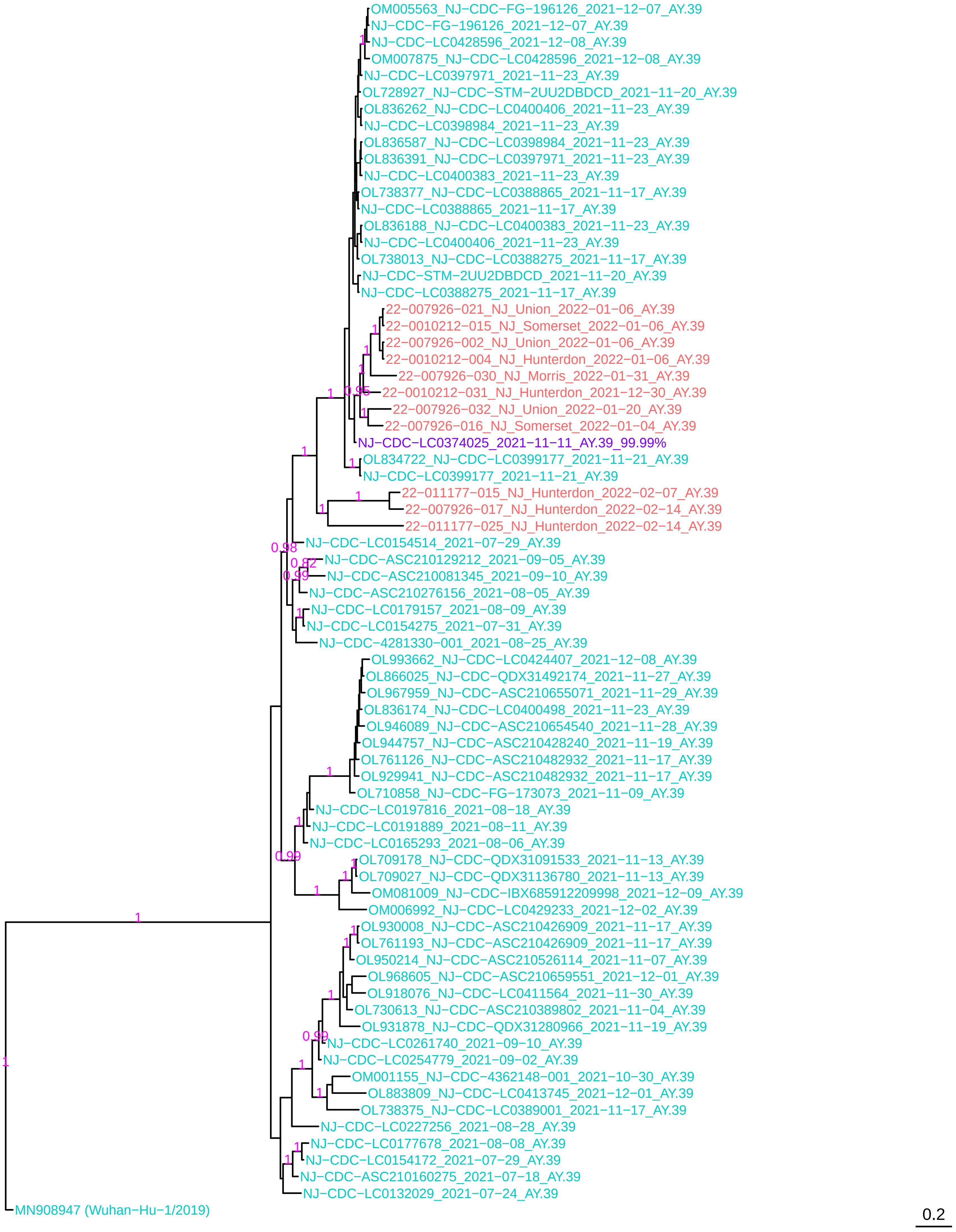
OM005563_NJ−CDC−FG−196126_2021−12−07_AY.39
NJ−CDC−FG−196126_2021−12−07_AY.39
NJ−CDC−LC0428596_2021−12−08_AY.39
OM007875_NJ−CDC−LC0428596_2021−12−08_AY.39
NJ−CDC−LC0397971_2021−11−23_AY.39
OL728927_NJ−CDC−STM−2UU2DBDCD_2021−11−20_AY.39
OL836262_NJ−CDC−LC0400406_2021−11−23_AY.39
NJ−CDC−LC0398984_2021−11−23_AY.39
OL836587_NJ−CDC−LC0398984_2021−11−23_AY.39
OL836391_NJ−CDC−LC0397971_2021−11−23_AY.39
NJ−CDC−LC0400383_2021−11−23_AY.39
OL738377_NJ−CDC−LC0388865_2021−11−17_AY.39
NJ−CDC−LC0388865_2021−11−17_AY.39
OL836188_NJ−CDC−LC0400383_2021−11−23_AY.39
NJ−CDC−LC0400406_2021−11−23_AY.39
OL738013_NJ−CDC−LC0388275_2021−11−17_AY.39
NJ−CDC−STM−2UU2DBDCD_2021−11−20_AY.39
NJ−CDC−LC0388275_2021−11−17_AY.39
22−007926−021_NJ_Union_2022−01−06_AY.39
22−0010212−015_NJ_Somerset_2022−01−06_AY.39
22−007926−002_NJ_Union_2022−01−06_AY.39
22−0010212−004_NJ_Hunterdon_2022−01−06_AY.39
22−007926−030_NJ_Morris_2022−01−31_AY.39
22−0010212−031_NJ_Hunterdon_2021−12−30_AY.39
22−007926−032_NJ_Union_2022−01−20_AY.39
22−007926−016_NJ_Somerset_2022−01−04_AY.39
NJ−CDC−LC0374025_2021−11−11_AY.39_99.99%
OL834722_NJ−CDC−LC0399177_2021−11−21_AY.39
NJ−CDC−LC0399177_2021−11−21_AY.39
22−011177−015_NJ_Hunterdon_2022−02−07_AY.39
22−007926−017_NJ_Hunterdon_2022−02−14_AY.39
22−011177−025_NJ_Hunterdon_2022−02−14_AY.39
NJ−CDC−LC0154514_2021−07−29_AY.39
NJ−CDC−ASC210129212_2021−09−05_AY.39
NJ−CDC−ASC210081345_2021−09−10_AY.39
NJ−CDC−ASC210276156_2021−08−05_AY.39
NJ−CDC−LC0179157_2021−08−09_AY.39
NJ−CDC−LC0154275_2021−07−31_AY.39
NJ−CDC−4281330−001_2021−08−25_AY.39
OL993662_NJ−CDC−LC0424407_2021−12−08_AY.39
OL866025_NJ−CDC−QDX31492174_2021−11−27_AY.39
OL967959_NJ−CDC−ASC210655071_2021−11−29_AY.39
OL836174_NJ−CDC−LC0400498_2021−11−23_AY.39
OL946089_NJ−CDC−ASC210654540_2021−11−28_AY.39
OL944757_NJ−CDC−ASC210428240_2021−11−19_AY.39
OL761126_NJ−CDC−ASC210482932_2021−11−17_AY.39
OL929941_NJ−CDC−ASC210482932_2021−11−17_AY.39
OL710858_NJ−CDC−FG−173073_2021−11−09_AY.39
NJ−CDC−LC0197816_2021−08−18_AY.39
NJ−CDC−LC0191889_2021−08−11_AY.39
NJ−CDC−LC0165293_2021−08−06_AY.39
OL709178_NJ−CDC−QDX31091533_2021−11−13_AY.39
OL709027_NJ−CDC−QDX31136780_2021−11−13_AY.39
OM081009_NJ−CDC−IBX685912209998_2021−12−09_AY.39
OM006992_NJ−CDC−LC0429233_2021−12−02_AY.39
OL930008_NJ−CDC−ASC210426909_2021−11−17_AY.39
OL761193_NJ−CDC−ASC210426909_2021−11−17_AY.39
OL950214_NJ−CDC−ASC210526114_2021−11−07_AY.39
OL968605_NJ−CDC−ASC210659551_2021−12−01_AY.39
OL918076_NJ−CDC−LC0411564_2021−11−30_AY.39
OL730613_NJ−CDC−ASC210389802_2021−11−04_AY.39
OL931878_NJ−CDC−QDX31280966_2021−11−19_AY.39
NJ−CDC−LC0261740_2021−09−10_AY.39
NJ−CDC−LC0254779_2021−09−02_AY.39
OM001155_NJ−CDC−4362148−001_2021−10−30_AY.39
OL883809_NJ−CDC−LC0413745_2021−12−01_AY.39
OL738375_NJ−CDC−LC0389001_2021−11−17_AY.39
NJ−CDC−LC0227256_2021−08−28_AY.39
NJ−CDC−LC0177678_2021−08−08_AY.39
NJ−CDC−LC0154172_2021−07−29_AY.39
NJ−CDC−ASC210160275_2021−07−18_AY.39
NJ−CDC−LC0132029_2021−07−24_AY.39
MN908947 (Wuhan−Hu−1/2019)
0.2

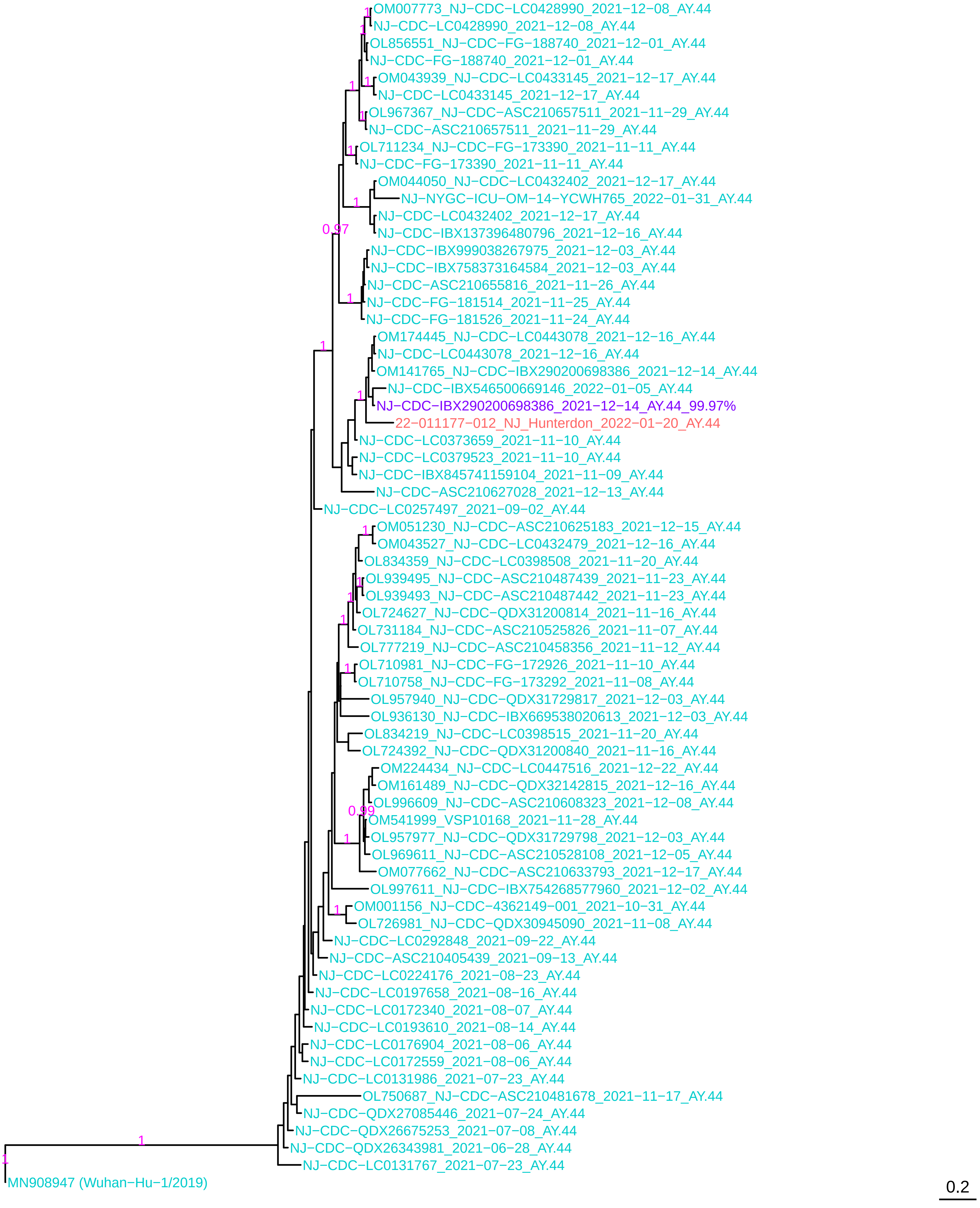
OM007773_NJ−CDC−LC0428990_2021−12−08_AY.44
NJ−CDC−LC0428990_2021−12−08_AY.44
OL856551_NJ−CDC−FG−188740_2021−12−01_AY.44
NJ−CDC−FG−188740_2021−12−01_AY.44
OM043939_NJ−CDC−LC0433145_2021−12−17_AY.44
NJ−CDC−LC0433145_2021−12−17_AY.44
OL967367_NJ−CDC−ASC210657511_2021−11−29_AY.44
NJ−CDC−ASC210657511_2021−11−29_AY.44
OL711234_NJ−CDC−FG−173390_2021−11−11_AY.44
NJ−CDC−FG−173390_2021−11−11_AY.44
OM044050_NJ−CDC−LC0432402_2021−12−17_AY.44
NJ−NYGC−ICU−OM−14−YCWH765_2022−01−31_AY.44
NJ−CDC−LC0432402_2021−12−17_AY.44
NJ−CDC−IBX137396480796_2021−12−16_AY.44
NJ−CDC−IBX99038267975_2021−12−03_AY.44
NJ−CDC−IBX758373164584_2021−12−03_AY.44
NJ−CDC−ASC210655816_2021−11−26_AY.44
NJ−CDC−FG−181514_2021−11−25_AY.44
NJ−CDC−FG−181526_2021−11−24_AY.44
OM174445_NJ−CDC−LC0443078_2021−12−16_AY.44
NJ−CDC−LC0443078_2021−12−16_AY.44
OM141765_NJ−CDC−IBX290200698386_2021−12−14_AY.44
NJ−CDC−IBX546500669146_2022−01−05_AY.44
NJ−CDC−IBX290200698386_2021−12−14_AY.44_99.97%
22−011177−012_NJ_Hunterdon_2022−01−20_AY.44
NJ−CDC−LC0373659_2021−11−10_AY.44
NJ−CDC−LC0379523_2021−11−10_AY.44
NJ−CDC−IBX845741159104_2021−11−09_AY.44
NJ−CDC−ASC210627028_2021−12−13_AY.44
NJ−CDC−LC0257497_2021−09−02_AY.44
OM051230_NJ−CDC−ASC210625183_2021−12−15_AY.44
OM043527_NJ−CDC−LC0432479_2021−12−16_AY.44
OL834359_NJ−CDC−LC0398508_2021−11−20_AY.44
OL939495_NJ−CDC−ASC210487439_2021−11−23_AY.44
OL939493_NJ−CDC−ASC210487442_2021−11−23_AY.44
OL724627_NJ−CDC−QDX31200814_2021−11−16_AY.44
OL731184_NJ−CDC−ASC210525826_2021−11−07_AY.44
OL777219_NJ−CDC−ASC210458356_2021−11−12_AY.44
OL710981_NJ−CDC−FG−172926_2021−11−10_AY.44
OL710758_NJ−CDC−FG−173292_2021−11−08_AY.44
OL957940_NJ−CDC−QDX31729817_2021−12−03_AY.44
OL936130_NJ−CDC−IBX669538020613_2021−12−03_AY.44
OL834219_NJ−CDC−LC0398515_2021−11−20_AY.44
OL724392_NJ−CDC−QDX31200840_2021−11−16_AY.44
OM224434_NJ−CDC−LC0447516_2021−12−22_AY.44
OM161489_NJ−CDC−QDX32142815_2021−12−16_AY.44
OL996609_NJ−CDC−ASC210608323_2021−12−08_AY.44
OM541999_VSP10168_2021−11−28_AY.44
OL957977_NJ−CDC−QDX31729798_2021−12−03_AY.44
OL969611_NJ−CDC−ASC210528108_2021−12−05_AY.44
OM077662_NJ−CDC−ASC210633793_2021−12−17_AY.44
OL997611_NJ−CDC−IBX754268577960_2021−12−02_AY.44
OM001156_NJ−CDC−4362149−001_2021−10−31_AY.44
OL726981_NJ−CDC−QDX30945090_2021−11−08_AY.44
NJ−CDC−LC0292848_2021−09−22_AY.44
NJ−CDC−ASC210405439_2021−09−13_AY.44
NJ−CDC−LC0224176_2021−08−23_AY.44
NJ−CDC−LC0197658_2021−08−16_AY.44
NJ−CDC−LC0172340_2021−08−07_AY.44
NJ−CDC−LC0193610_2021−08−14_AY.44
NJ−CDC−LC0176904_2021−08−06_AY.44
NJ−CDC−LC0172559_2021−08−06_AY.44
NJ−CDC−LC0131986_2021−07−23_AY.44
OL750687_NJ−CDC−ASC210481678_2021−11−17_AY.44
NJ−CDC−QDX27085446_2021−07−24_AY.44
NJ−CDC−QDX26675253_2021−07−08_AY.44
NJ−CDC−QDX26343981_2021−06−28_AY.44
NJ−CDC−LC0131767_2021−07−23_AY.44
MN908947 (Wuhan−Hu−1/2019)
0.97
0.99
0.2

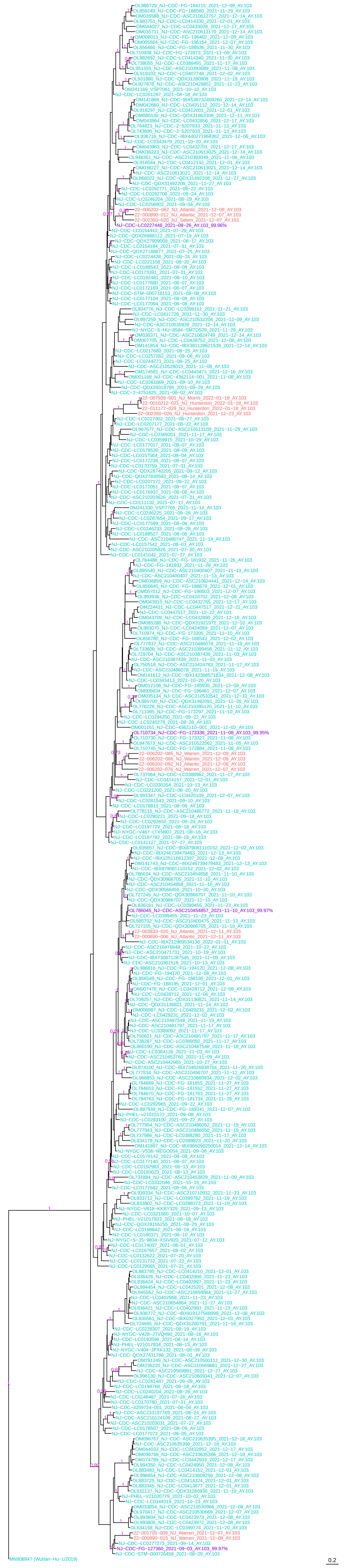
MN908947 (Wuhan-Hu-1/2019)
0.2

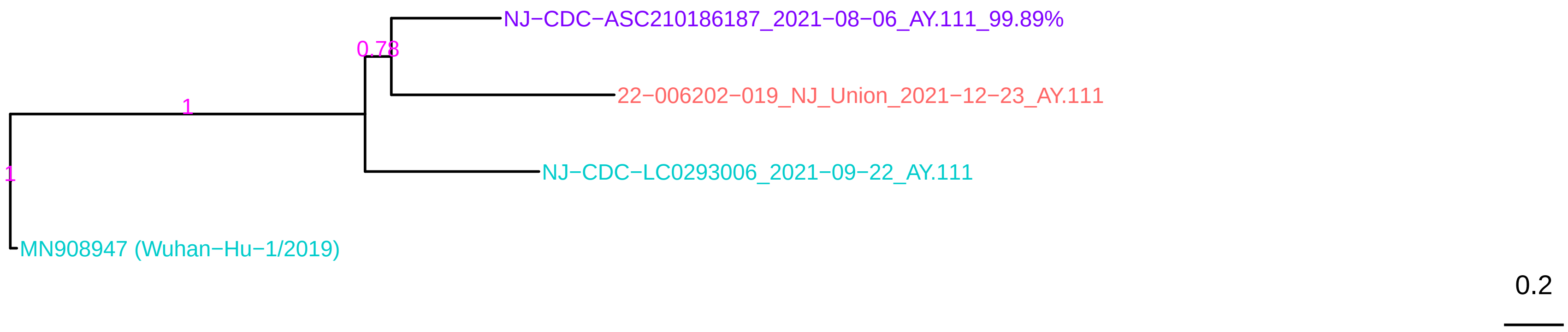
NJ−CDC−ASC210186187_2021−08−06_AY.111_99.89%
0.78
22−006202−019_NJ_Union_2021−12−23_AY.111
1
NJ−CDC−LC0293006_2021−09−22_AY.111
1
MN908947 (Wuhan−Hu−1/2019)
0.2

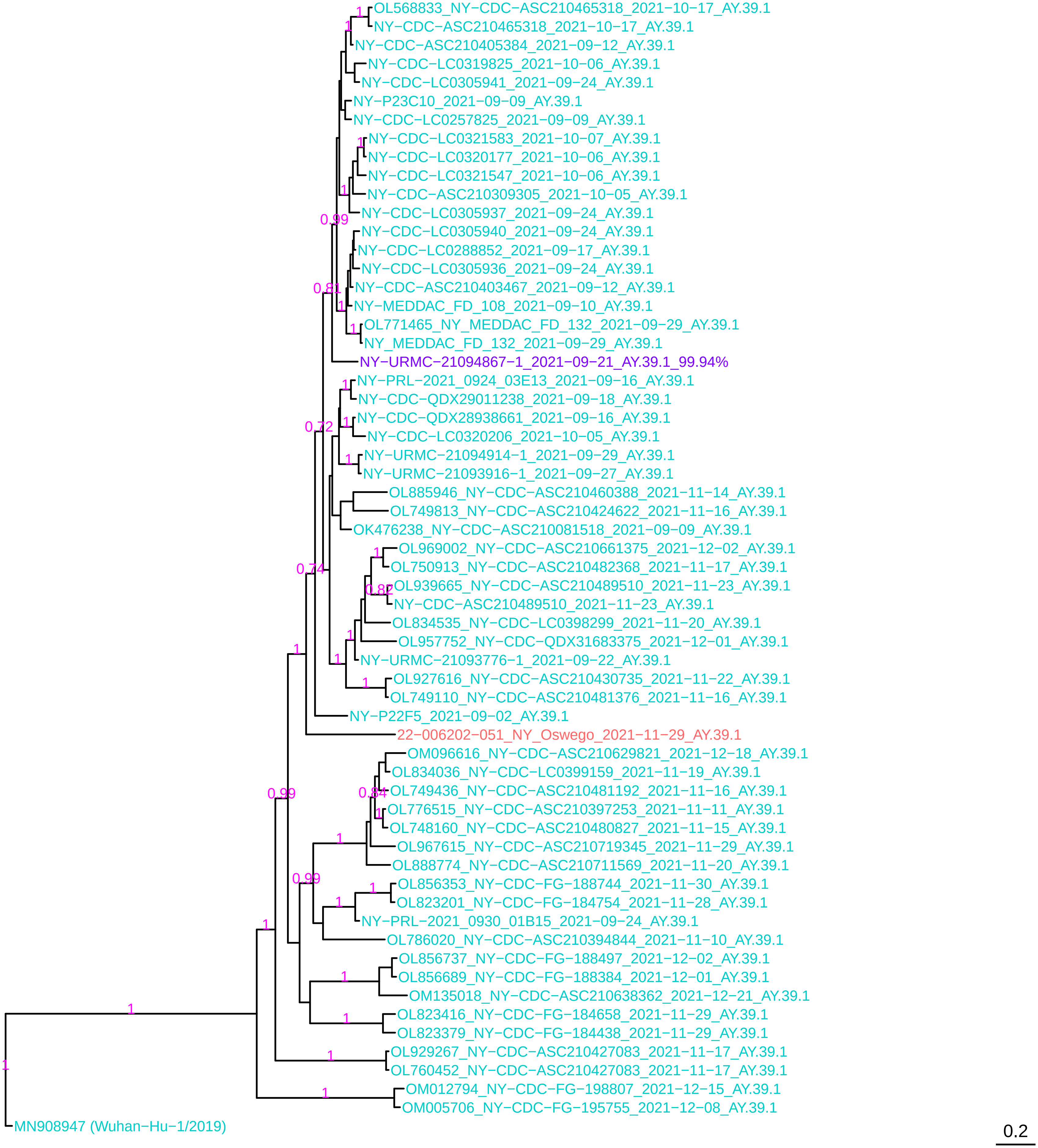
OL568833_NY−CDC−ASC210465318_2021−10−17_AY.39.1
NY−CDC−ASC210465318_2021−10−17_AY.39.1
NY−CDC−ASC210405384_2021−09−12_AY.39.1
NY−CDC−LC0319825_2021−10−06_AY.39.1
NY−CDC−LC0305941_2021−09−24_AY.39.1
NY−P23C10_2021−09−09_AY.39.1
NY−CDC−LC0257825_2021−09−09_AY.39.1
NY−CDC−LC0321583_2021−10−07_AY.39.1
NY−CDC−LC0320177_2021−10−06_AY.39.1
NY−CDC−LC0321547_2021−10−06_AY.39.1
NY−CDC−ASC210309305_2021−10−05_AY.39.1
NY−CDC−LC0305937_2021−09−24_AY.39.1
NY−CDC−LC0305940_2021−09−24_AY.39.1
NY−CDC−LC0288852_2021−09−17_AY.39.1
NY−CDC−LC0305936_2021−09−24_AY.39.1
NY−CDC−ASC210403467_2021−09−12_AY.39.1
NY−MEDDAC_FD_108_2021−09−10_AY.39.1
OL771465_NY_MEDDAC_FD_132_2021−09−29_AY.39.1
NY_MEDDAC_FD_132_2021−09−29_AY.39.1
NY−URMC−21094867−1_2021−09−21_AY.39.1_99.94%
NY−PRL−2021_0924_03E13_2021−09−16_AY.39.1
NY−CDC−QDX29011238_2021−09−18_AY.39.1
NY−CDC−QDX28938661_2021−09−16_AY.39.1
NY−CDC−LC0320206_2021−10−05_AY.39.1
NY−URMC−21094914−1_2021−09−29_AY.39.1
NY−URMC−21093916−1_2021−09−27_AY.39.1
OL885946_NY−CDC−ASC210460388_2021−11−14_AY.39.1
OL749813_NY−CDC−ASC210424622_2021−11−16_AY.39.1
OK476238_NY−CDC−ASC210081518_2021−09−09_AY.39.1
OL969002_NY−CDC−ASC210661375_2021−12−02_AY.39.1
OL750913_NY−CDC−ASC210482368_2021−11−17_AY.39.1
OL939665_NY−CDC−ASC210489510_2021−11−23_AY.39.1
NY−CDC−ASC210489510_2021−11−23_AY.39.1
OL834535_NY−CDC−LC0398299_2021−11−20_AY.39.1
OL957752_NY−CDC−QDX31683375_2021−12−01_AY.39.1
NY−URMC−21093776−1_2021−09−22_AY.39.1
OL927616_NY−CDC−ASC210430735_2021−11−22_AY.39.1
OL749110_NY−CDC−ASC210481376_2021−11−16_AY.39.1
NY−P22F5_2021−09−02_AY.39.1
22−006202−051_NY_Oswego_2021−11−29_AY.39.1
OM096616_NY−CDC−ASC210629821_2021−12−18_AY.39.1
OL834036_NY−CDC−LC0399159_2021−11−19_AY.39.1
OL749436_NY−CDC−ASC210481192_2021−11−16_AY.39.1
OL776515_NY−CDC−ASC210397253_2021−11−11_AY.39.1
OL748160_NY−CDC−ASC210480827_2021−11−15_AY.39.1
OL967615_NY−CDC−ASC210719345_2021−11−29_AY.39.1
OL888774_NY−CDC−ASC210711569_2021−11−20_AY.39.1
OL856353_NY−CDC−FG−188744_2021−11−30_AY.39.1
OL823201_NY−CDC−FG−184754_2021−11−28_AY.39.1
NY−PRL−2021_0930_01B15_2021−09−24_AY.39.1
OL786020_NY−CDC−ASC210394844_2021−11−10_AY.39.1
OL856737_NY−CDC−FG−188497_2021−12−02_AY.39.1
OL856689_NY−CDC−FG−188384_2021−12−01_AY.39.1
OM135018_NY−CDC−ASC210638362_2021−12−21_AY.39.1
OL823416_NY−CDC−FG−184658_2021−11−29_AY.39.1
OL823379_NY−CDC−FG−184438_2021−11−29_AY.39.1
OL929267_NY−CDC−ASC210427083_2021−11−17_AY.39.1
OL760452_NY−CDC−ASC210427083_2021−11−17_AY.39.1
OM012794_NY−CDC−FG−198807_2021−12−15_AY.39.1
OM005706_NY−CDC−FG−195755_2021−12−08_AY.39.1
MN908947 (Wuhan−Hu−1/2019)
0.99
0.81
0.72
0.74
0.82
0.99
0.84
0.99
0.2

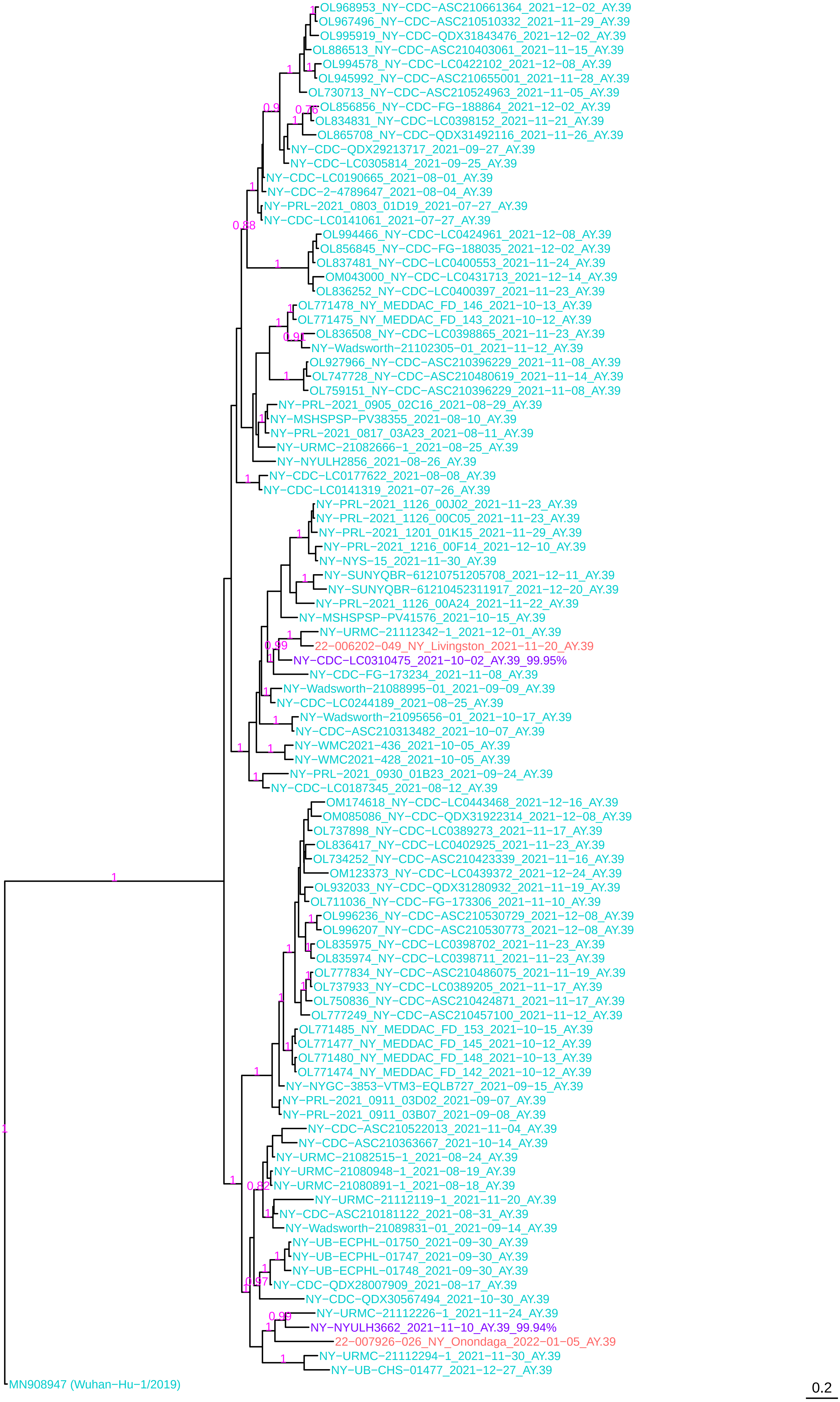

OL968953_NY−CDC−ASC210661364_2021−12−02_AY.39
OL967496_NY−CDC−ASC210510332_2021−11−29_AY.39
OL995919_NY−CDC−QDX31843476_2021−12−02_AY.39
OL886513_NY−CDC−ASC210403061_2021−11−15_AY.39
OL994578_NY−CDC−LC0422102_2021−12−08_AY.39
OL945992_NY−CDC−ASC210655001_2021−11−28_AY.39
OL730713_NY−CDC−ASC210524963_2021−11−05_AY.39
OL856856_NY−CDC−FG−188864_2021−12−02_AY.39
OL834831_NY−CDC−LC0398152_2021−11−21_AY.39
OL865708_NY−CDC−QDX31492116_2021−11−26_AY.39
NY−CDC−QDX29213717_2021−09−27_AY.39
NY−CDC−LC0305814_2021−09−25_AY.39
NY−CDC−LC0190665_2021−08−01_AY.39
NY−CDC−2−4789647_2021−08−04_AY.39
NY−PRL−2021_0803_01D19_2021−07−27_AY.39
NY−CDC−LC0141061_2021−07−27_AY.39
OL994466_NY−CDC−LC0424961_2021−12−08_AY.39
OL856845_NY−CDC−FG−188035_2021−12−02_AY.39
OL837481_NY−CDC−LC0400553_2021−11−24_AY.39
OM043000_NY−CDC−LC0431713_2021−12−14_AY.39
OL836252_NY−CDC−LC0400397_2021−11−23_AY.39
OL771478_NY_MEDDAC_FD_146_2021−10−13_AY.39
OL771475_NY_MEDDAC_FD_143_2021−10−12_AY.39
OL836508_NY−CDC−LC0398865_2021−11−23_AY.39
NY−Wadsworth−21102305−01_2021−11−12_AY.39
OL927966_NY−CDC−ASC210396229_2021−11−08_AY.39
OL747728_NY−CDC−ASC210480619_2021−11−14_AY.39
OL759151_NY−CDC−ASC210396229_2021−11−08_AY.39
NY−PRL−2021_0905_02C16_2021−08−29_AY.39
NY−MSHSPSP−PV38355_2021−08−10_AY.39
NY−PRL−2021_0817_03A23_2021−08−11_AY.39
NY−URMC−21082666−1_2021−08−25_AY.39
NY−NYULH2856_2021−08−26_AY.39
NY−CDC−LC0177622_2021−08−08_AY.39
NY−CDC−LC0141319_2021−07−26_AY.39
NY−PRL−2021_1126_00J02_2021−11−23_AY.39
NY−PRL−2021_1126_00C05_2021−11−23_AY.39
NY−PRL−2021_1201_01K15_2021−11−29_AY.39
NY−PRL−2021_1216_00F14_2021−12−10_AY.39
NY−NYS−15_2021−11−30_AY.39
NY−SUNYQBR−61210751205708_2021−12−11_AY.39
NY−SUNYQBR−61210452311917_2021−12−20_AY.39
NY−PRL−2021_1126_00A24_2021−11−22_AY.39
NY−MSHSPSP−PV41576_2021−10−15_AY.39
NY−URMC−21112342−1_2021−12−01_AY.39
22−006202−049_NY_Livingston_2021−11−20_AY.39
NY−CDC−LC0310475_2021−10−02_AY.39_99.95%
NY−CDC−FG−173234_2021−11−08_AY.39
NY−Wadsworth−21088995−01_2021−09−09_AY.39
NY−CDC−LC0244189_2021−08−25_AY.39
NY−Wadsworth−21095656−01_2021−10−17_AY.39
NY−CDC−ASC210313482_2021−10−07_AY.39
NY−WMC2021−436_2021−10−05_AY.39
NY−WMC2021−428_2021−10−05_AY.39
NY−PRL−2021_0930_01B23_2021−09−24_AY.39
NY−CDC−LC0187345_2021−08−12_AY.39
OM174618_NY−CDC−LC0443468_2021−12−16_AY.39
OM085086_NY−CDC−QDX31922314_2021−12−08_AY.39
OL737898_NY−CDC−LC0389273_2021−11−17_AY.39
OL836417_NY−CDC−LC0402925_2021−11−23_AY.39
OL734252_NY−CDC−ASC210423339_2021−11−16_AY.39
OM123373_NY−CDC−LC0439372_2021−12−24_AY.39
OL932033_NY−CDC−QDX31280932_2021−11−19_AY.39
OL711036_NY−CDC−FG−173306_2021−11−10_AY.39
OL996236_NY−CDC−ASC210530729_2021−12−08_AY.39
OL996207_NY−CDC−ASC210530773_2021−12−08_AY.39
OL835975_NY−CDC−LC0398702_2021−11−23_AY.39
OL835974_NY−CDC−LC0398711_2021−11−23_AY.39
OL777834_NY−CDC−ASC210486075_2021−11−19_AY.39
OL737933_NY−CDC−LC0389205_2021−11−17_AY.39
OL750836_NY−CDC−ASC210424871_2021−11−17_AY.39
OL777249_NY−CDC−ASC210457100_2021−11−12_AY.39
OL771485_NY_MEDDAC_FD_153_2021−10−15_AY.39
OL771477_NY_MEDDAC_FD_145_2021−10−12_AY.39
OL771480_NY_MEDDAC_FD_148_2021−10−13_AY.39
OL771474_NY_MEDDAC_FD_142_2021−10−12_AY.39
NY−NYGC−3853−VTM3−EQLB727_2021−09−15_AY.39
NY−PRL−2021_0911_03D02_2021−09−07_AY.39
NY−PRL−2021_0911_03B07_2021−09−08_AY.39
NY−CDC−ASC210522013_2021−11−04_AY.39
NY−CDC−ASC210363667_2021−10−14_AY.39
NY−URMC−21082515−1_2021−08−24_AY.39
NY−URMC−21080948−1_2021−08−19_AY.39
NY−URMC−21080891−1_2021−08−18_AY.39
NY−URMC−21112119−1_2021−11−20_AY.39
NY−CDC−ASC210181122_2021−08−31_AY.39
NY−Wadsworth−21089831−01_2021−09−14_AY.39
NY−UB−ECPHL−01750_2021−09−30_AY.39
NY−UB−ECPHL−01747_2021−09−30_AY.39
NY−UB−ECPHL−01748_2021−09−30_AY.39
NY−CDC−QDX28007909_2021−08−17_AY.39
NY−CDC−QDX30567494_2021−10−30_AY.39
NY−URMC−21112226−1_2021−11−24_AY.39
NY−NYULH3662_2021−11−10_AY.39_99.94%
22−007926−026_NY_Onondaga_2022−01−05_AY.39
NY−URMC−21112294−1_2021−11−30_AY.39
NY−UB−CHS−01477_2021−12−27_AY.39
MN908947 (Wuhan−Hu−1/2019)
0.2

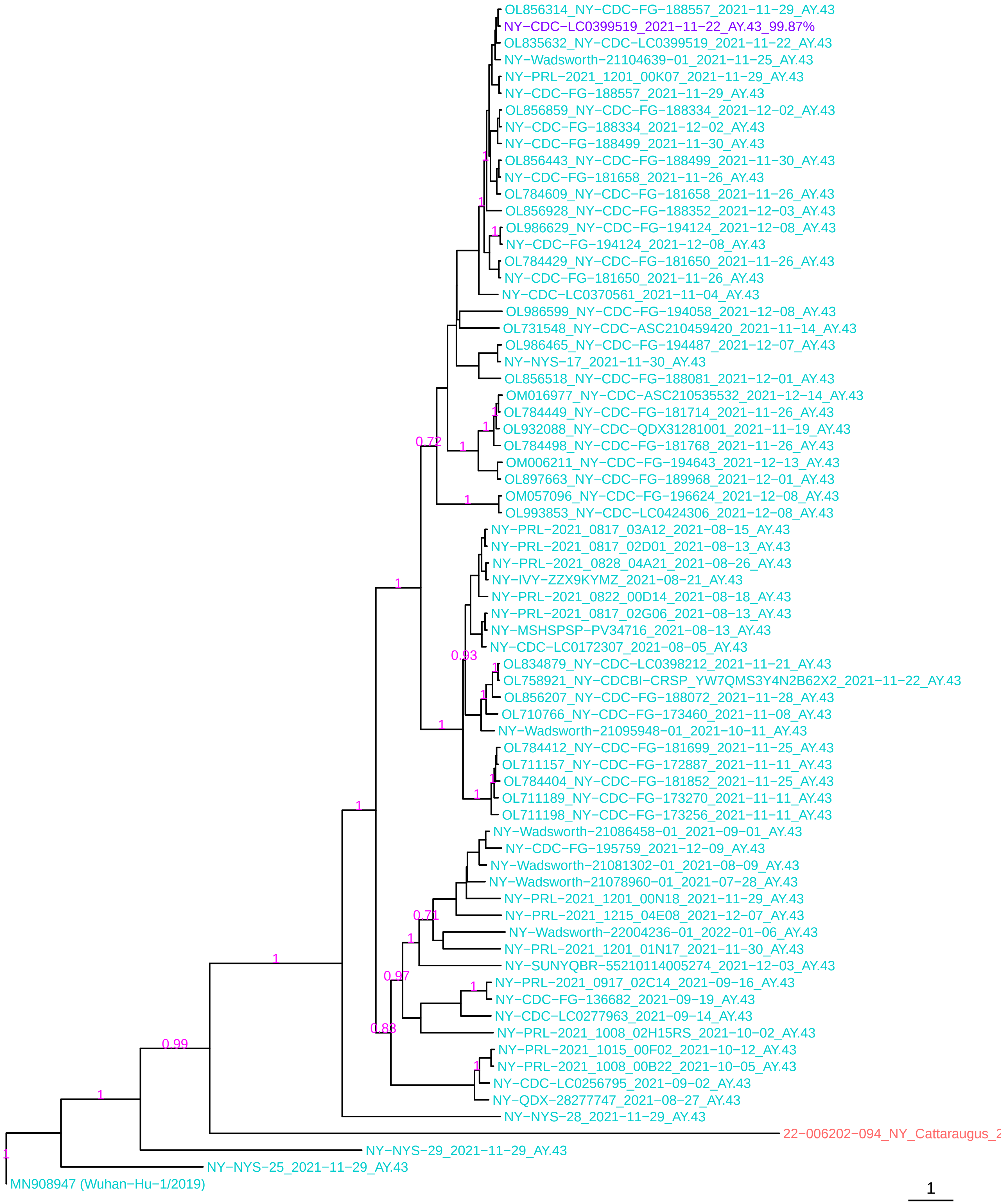

OL856314_NY−CDC−FG−188557_2021−11−29_AY.43
NY−CDC−LC0399519_2021−11−22_AY.43_99.87%
OL835632_NY−CDC−LC0399519_2021−11−22_AY.43
NY−Wadsworth−21104639−01_2021−11−25_AY.43
NY−PRL−2021_1201_00K07_2021−11−29_AY.43
NY−CDC−FG−188557_2021−11−29_AY.43
OL856859_NY−CDC−FG−188334_2021−12−02_AY.43
NY−CDC−FG−188334_2021−12−02_AY.43
NY−CDC−FG−188499_2021−11−30_AY.43
OL856443_NY−CDC−FG−188499_2021−11−30_AY.43
NY−CDC−FG−181658_2021−11−26_AY.43
OL784609_NY−CDC−FG−181658_2021−11−26_AY.43
OL856928_NY−CDC−FG−188352_2021−12−03_AY.43
OL986629_NY−CDC−FG−194124_2021−12−08_AY.43
NY−CDC−FG−194124_2021−12−08_AY.43
OL784429_NY−CDC−FG−181650_2021−11−26_AY.43
NY−CDC−FG−181650_2021−11−26_AY.43
NY−CDC−LC0370561_2021−11−04_AY.43
OL986599_NY−CDC−FG−194058_2021−12−08_AY.43
OL731548_NY−CDC−ASC210459420_2021−11−14_AY.43
OL986465_NY−CDC−FG−194487_2021−12−07_AY.43
NY−NYS−17_2021−11−30_AY.43
OL856518_NY−CDC−FG−188081_2021−12−01_AY.43
OM016977_NY−CDC−ASC210535532_2021−12−14_AY.43
OL784449_NY−CDC−FG−181714_2021−11−26_AY.43
OL932088_NY−CDC−QDX31281001_2021−11−19_AY.43
OL784498_NY−CDC−FG−181768_2021−11−26_AY.43
OM006211_NY−CDC−FG−194643_2021−12−13_AY.43
OL897663_NY−CDC−FG−189968_2021−12−01_AY.43
OM057096_NY−CDC−FG−196624_2021−12−08_AY.43
OL993853_NY−CDC−LC0424306_2021−12−08_AY.43
NY−PRL−2021_0817_03A12_2021−08−15_AY.43
NY−PRL−2021_0817_02D01_2021−08−13_AY.43
NY−PRL−2021_0828_04A21_2021−08−26_AY.43
NY−IVY−ZZX9KYMZ_2021−08−21_AY.43
NY−PRL−2021_0822_00D14_2021−08−18_AY.43
NY−PRL−2021_0817_02G06_2021−08−13_AY.43
NY−MSHSPSP−PV34716_2021−08−13_AY.43
NY−CDC−LC0172307_2021−08−05_AY.43
OL834879_NY−CDC−LC0398212_2021−11−21_AY.43
OL758921_NY−CDCBI−CRSP_YW7QMS3Y4N2B62X2_2021−11−22_AY.43
OL856207_NY−CDC−FG−188072_2021−11−28_AY.43
OL710766_NY−CDC−FG−173460_2021−11−08_AY.43
NY−Wadsworth−21095948−01_2021−10−11_AY.43
OL784412_NY−CDC−FG−181699_2021−11−25_AY.43
OL711157_NY−CDC−FG−172887_2021−11−11_AY.43
OL784404_NY−CDC−FG−181852_2021−11−25_AY.43
OL711189_NY−CDC−FG−173270_2021−11−11_AY.43
OL711198_NY−CDC−FG−173256_2021−11−11_AY.43
NY−Wadsworth−21086458−01_2021−09−01_AY.43
NY−CDC−FG−195759_2021−12−09_AY.43
NY−Wadsworth−21081302−01_2021−08−09_AY.43
NY−Wadsworth−21078960−01_2021−07−28_AY.43
NY−PRL−2021_1201_00N18_2021−11−29_AY.43
NY−PRL−2021_1215_04E08_2021−12−07_AY.43
NY−Wadsworth−22004236−01_2022−01−06_AY.43
NY−PRL−2021_1201_01N17_2021−11−30_AY.43
NY−SUNYQBR−55210114005274_2021−12−03_AY.43
NY−PRL−2021_0917_02C14_2021−09−16_AY.43
NY−CDC−FG−136682_2021−09−19_AY.43
NY−CDC−LC0277963_2021−09−14_AY.43
NY−PRL−2021_1008_02H15RS_2021−10−02_AY.43
NY−PRL−2021_1015_00F02_2021−10−12_AY.43
NY−PRL−2021_1008_00B22_2021−10−05_AY.43
NY−CDC−LC0256795_2021−09−02_AY.43
NY−QDX−28277747_2021−08−27_AY.43
NY−NYS−28_2021−11−29_AY.43
22−006202−094_NY_Cattaraugus_2
NY−NYS−29_2021−11−29_AY.43
NY−NYS−25_2021−11−29_AY.43
MN908947 (Wuhan−Hu−1/2019)
0.72
0.93
0.71
0.97
0.83
0.99
1

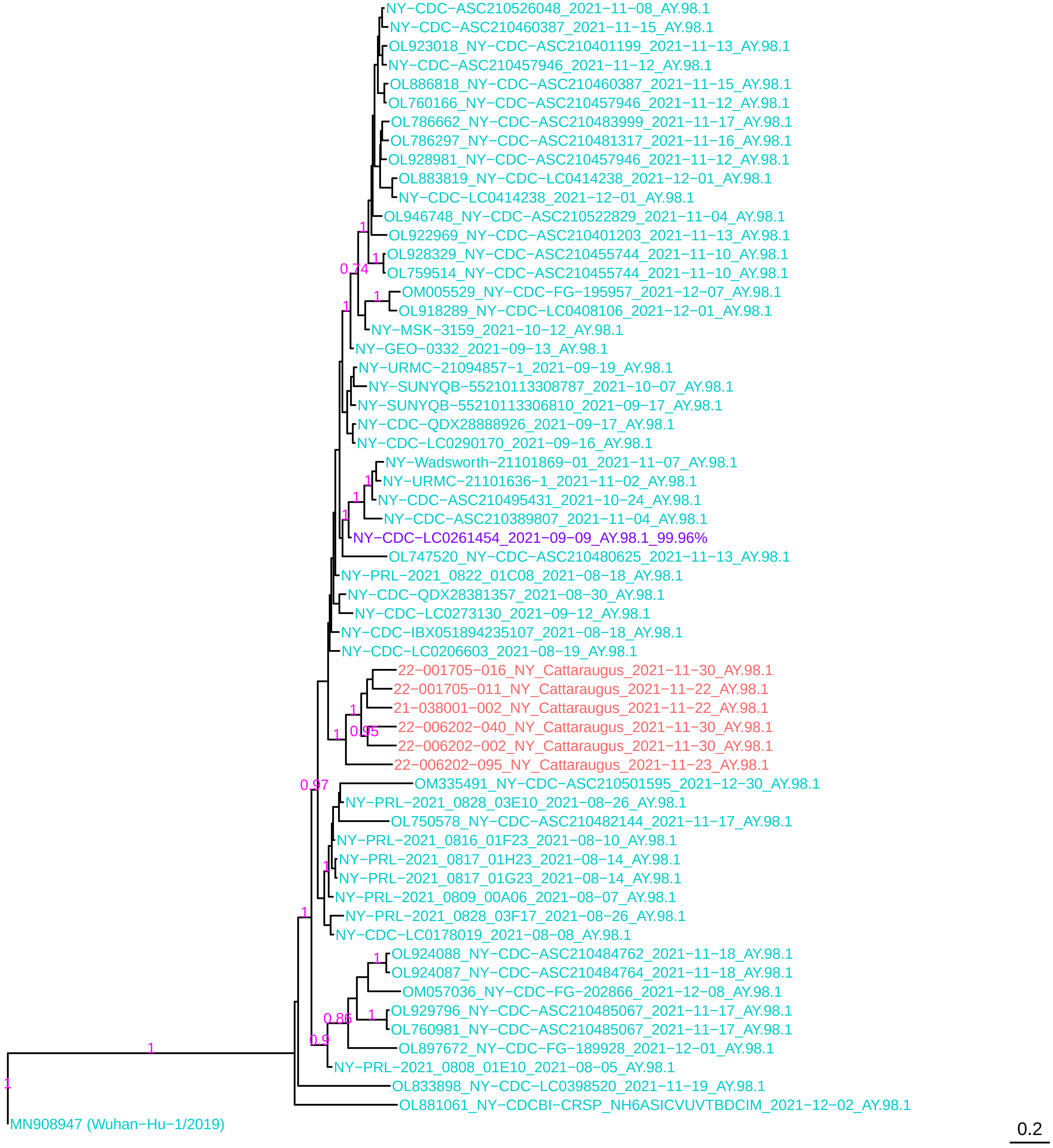

NY−CDC−ASC210526048_2021−11−08_AY.98.1
NY−CDC−ASC210460387_2021−11−15_AY.98.1
OL923018_NY−CDC−ASC210401199_2021−11−13_AY.98.1
NY−CDC−ASC210457946_2021−11−12_AY.98.1
OL886818_NY−CDC−ASC210460387_2021−11−15_AY.98.1
OL760166_NY−CDC−ASC210457946_2021−11−12_AY.98.1
OL786662_NY−CDC−ASC210483999_2021−11−17_AY.98.1
OL786297_NY−CDC−ASC210481317_2021−11−16_AY.98.1
OL928981_NY−CDC−ASC210457946_2021−11−12_AY.98.1
OL883819_NY−CDC−LC0414238_2021−12−01_AY.98.1
NY−CDC−LC0414238_2021−12−01_AY.98.1
OL946748_NY−CDC−ASC210522829_2021−11−04_AY.98.1
OL922969_NY−CDC−ASC210401203_2021−11−13_AY.98.1
OL928329_NY−CDC−ASC210455744_2021−11−10_AY.98.1
OL759514_NY−CDC−ASC210455744_2021−11−10_AY.98.1
OM005529_NY−CDC−FG−195957_2021−12−07_AY.98.1
OL918289_NY−CDC−LC0408106_2021−12−01_AY.98.1
NY−MSK−3159_2021−10−12_AY.98.1
NY−GEO−0332_2021−09−13_AY.98.1
NY−URMC−21094857−1_2021−09−19_AY.98.1
NY−SUNYQB−55210113308787_2021−10−07_AY.98.1
NY−SUNYQB−55210113306810_2021−09−17_AY.98.1
NY−CDC−QDX28888926_2021−09−17_AY.98.1
NY−CDC−LC0290170_2021−09−16_AY.98.1
NY−Wadsworth−21101869−01_2021−11−07_AY.98.1
NY−URMC−21101636−1_2021−11−02_AY.98.1
NY−CDC−ASC210495431_2021−10−24_AY.98.1
NY−CDC−ASC210389807_2021−11−04_AY.98.1
NY−CDC−LC0261454_2021−09−09_AY.98.1_99.96%
OL747520_NY−CDC−ASC210480625_2021−11−13_AY.98.1
NY−PRL−2021_0822_01C08_2021−08−18_AY.98.1
NY−CDC−QDX28381357_2021−08−30_AY.98.1
NY−CDC−LC0273130_2021−09−12_AY.98.1
NY−CDC−IBX051894235107_2021−08−18_AY.98.1
NY−CDC−LC0206603_2021−08−19_AY.98.1
22−001705−016_NY_Cattaraugus_2021−11−30_AY.98.1
22−001705−011_NY_Cattaraugus_2021−11−22_AY.98.1
21−038001−002_NY_Cattaraugus_2021−11−22_AY.98.1
22−006202−040_NY_Cattaraugus_2021−11−30_AY.98.1
22−006202−002_NY_Cattaraugus_2021−11−30_AY.98.1
22−006202−095_NY_Cattaraugus_2021−11−23_AY.98.1
OM335491_NY−CDC−ASC210501595_2021−12−30_AY.98.1
NY−PRL−2021_0828_03E10_2021−08−26_AY.98.1
OL750578_NY−CDC−ASC210482144_2021−11−17_AY.98.1
NY−PRL−2021_0816_01F23_2021−08−10_AY.98.1
NY−PRL−2021_0817_01H23_2021−08−14_AY.98.1
NY−PRL−2021_0817_01G23_2021−08−14_AY.98.1
NY−PRL−2021_0809_00A06_2021−08−07_AY.98.1
NY−PRL−2021_0828_03F17_2021−08−26_AY.98.1
NY−CDC−LC0178019_2021−08−08_AY.98.1
OL924088_NY−CDC−ASC210484762_2021−11−18_AY.98.1
OL924087_NY−CDC−ASC210484764_2021−11−18_AY.98.1
OM057036_NY−CDC−FG−202866_2021−12−08_AY.98.1
OL929796_NY−CDC−ASC210485067_2021−11−17_AY.98.1
OL760981_NY−CDC−ASC210485067_2021−11−17_AY.98.1
OL897672_NY−CDC−FG−189928_2021−12−01_AY.98.1
NY−PRL−2021_0808_01E10_2021−08−05_AY.98.1
OL833898_NY−CDC−LC0398520_2021−11−19_AY.98.1
OL881061_NY−CDCBI−CRSP_NH6ASICVUVTBDCIM_2021−12−02_AY.98.1
MN908947 (Wuhan−Hu−1/2019)
1
0.74
1
1
1
1
1
1
1
0.95
1
0.97
1
1
1
1
0.86
0.9
1
1
0.2

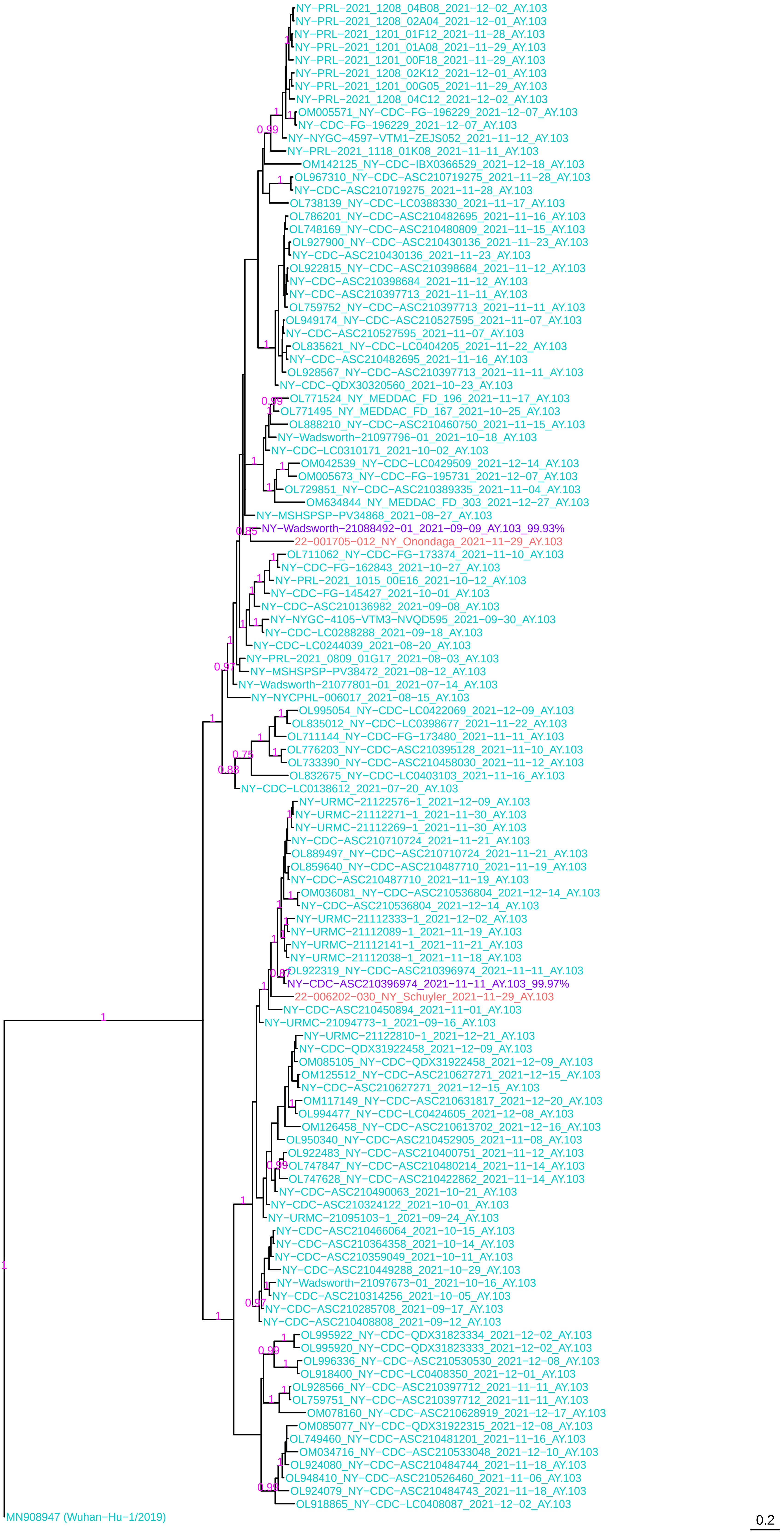
NY-PRL-2021_1208_04B08_2021-12-02_AY.103
NY-PRL-2021_1208_02A04_2021-12-01_AY.103
NY-PRL-2021_1201_01F12_2021-11-28_AY.103
NY-PRL-2021_1201_01A08_2021-11-29_AY.103
NY-PRL-2021_1201_00F18_2021-11-29_AY.103
NY-PRL-2021_1208_02K12_2021-12-01_AY.103
NY-PRL-2021_1201_00G05_2021-11-29_AY.103
NY-PRL-2021_1208_04C12_2021-12-02_AY.103
OM005571_NY-CDC-FG-196229_2021-12-07_AY.103
NY-CDC-FG-196229_2021-12-07_AY.103
NY-NYGC-4597-VTM1-ZEJS052_2021-11-12_AY.103
NY-PRL-2021_1118_01K08_2021-11-11_AY.103
OM142125_NY-CDC-IBX0366529_2021-12-18_AY.103
OL967310_NY-CDC-ASC210719275_2021-11-28_AY.103
NY-CDC-ASC210719275_2021-11-28_AY.103
OL738139_NY-CDC-LC0388330_2021-11-17_AY.103
OL786201_NY-CDC-ASC210482695_2021-11-16_AY.103
OL748169_NY-CDC-ASC210480809_2021-11-15_AY.103
OL927900_NY-CDC-ASC210430136_2021-11-23_AY.103
NY-CDC-ASC210430136_2021-11-23_AY.103
OL922815_NY-CDC-ASC210398684_2021-11-12_AY.103
NY-CDC-ASC210398684_2021-11-12_AY.103
NY-CDC-ASC210397713_2021-11-11_AY.103
OL759752_NY-CDC-ASC210397713_2021-11-11_AY.103
OL949174_NY-CDC-ASC210527595_2021-11-07_AY.103
NY-CDC-ASC210527595_2021-11-07_AY.103
OL835621_NY-CDC-LC0404205_2021-11-22_AY.103
NY-CDC-ASC210482695_2021-11-16_AY.103
OL928567_NY-CDC-ASC210397713_2021-11-11_AY.103
NY-CDC-QDX30320560_2021-10-23_AY.103
OL771524_NY_MEDDAC_FD_196_2021-11-17_AY.103
OL771495_NY_MEDDAC_FD_167_2021-10-25_AY.103
OL888210_NY-CDC-ASC210460750_2021-11-15_AY.103
NY-Wadsworth-21097796-01_2021-10-18_AY.103
NY-CDC-LC0310171_2021-10-02_AY.103
OM042539_NY-CDC-LC0429509_2021-12-14_AY.103
OM005673_NY-CDC-FG-195731_2021-12-07_AY.103
OL729851_NY-CDC-ASC210389335_2021-11-04_AY.103
OM634844_NY_MEDDAC_FD_303_2021-12-27_AY.103
NY-MSHSPSP-PV34868_2021-08-27_AY.103
NY-Wadsworth-21088492-01_2021-09-09_AY.103_99.93%
22-001705-012_NY_Onondaga_2021-11-29_AY.103
OL711062_NY-CDC-FG-173374_2021-11-10_AY.103
NY-CDC-FG-162843_2021-10-27_AY.103
NY-PRL-2021_1015_00E16_2021-10-12_AY.103
NY-CDC-FG-145427_2021-10-01_AY.103
NY-CDC-ASC210136982_2021-09-08_AY.103
NY-NYGC-4105-VTM3-NVQD595_2021-09-30_AY.103
NY-CDC-LC0288288_2021-09-18_AY.103
NY-CDC-LC0244039_2021-08-20_AY.103
NY-PRL-2021_0809_01G17_2021-08-03_AY.103
NY-MSHSPSP-PV38472_2021-08-12_AY.103
NY-Wadsworth-21077801-01_2021-07-14_AY.103
NY-NYCPHL-006017_2021-08-15_AY.103
OL995054_NY-CDC-LC0422069_2021-12-09_AY.103
OL835012_NY-CDC-LC0398677_2021-11-22_AY.103
OL711144_NY-CDC-FG-173480_2021-11-11_AY.103
OL776203_NY-CDC-ASC210395128_2021-11-10_AY.103
OL733390_NY-CDC-ASC210458030_2021-11-12_AY.103
OL832675_NY-CDC-LC0403103_2021-11-16_AY.103
NY-CDC-LC0138612_2021-07-20_AY.103
NY-URMC-21122576-1_2021-12-09_AY.103
NY-URMC-21112271-1_2021-11-30_AY.103
NY-URMC-21112269-1_2021-11-30_AY.103
NY-CDC-ASC210710724_2021-11-21_AY.103
OL889497_NY-CDC-ASC210710724_2021-11-21_AY.103
OL859640_NY-CDC-ASC210487710_2021-11-19_AY.103
NY-CDC-ASC210487710_2021-11-19_AY.103
OM036081_NY-CDC-ASC210536804_2021-12-14_AY.103
NY-CDC-ASC210536804_2021-12-14_AY.103
NY-URMC-21112333-1_2021-12-02_AY.103
NY-URMC-21112089-1_2021-11-19_AY.103
NY-URMC-21112141-1_2021-11-21_AY.103
NY-URMC-21112038-1_2021-11-18_AY.103
OL922319_NY-CDC-ASC210396974_2021-11-11_AY.103
NY-CDC-ASC210396974_2021-11-11_AY.103_99.97%
22-006202-030_NY_Schuyler_2021-11-29_AY.103
NY-CDC-ASC210450894_2021-11-01_AY.103
NY-URMC-21094773-1_2021-09-16_AY.103
NY-URMC-21122810-1_2021-12-21_AY.103
NY-CDC-QDX31922458_2021-12-09_AY.103
OM085105_NY-CDC-QDX31922458_2021-12-09_AY.103
OM125512_NY-CDC-ASC210627271_2021-12-15_AY.103
NY-CDC-ASC210627271_2021-12-15_AY.103
OM117149_NY-CDC-ASC210631817_2021-12-20_AY.103
OL994477_NY-CDC-LC0424605_2021-12-08_AY.103
OM126458_NY-CDC-ASC210613702_2021-12-16_AY.103
OL950340_NY-CDC-ASC210452905_2021-11-08_AY.103
OL922483_NY-CDC-ASC210400751_2021-11-12_AY.103
OL747847_NY-CDC-ASC210480214_2021-11-14_AY.103
OL747628_NY-CDC-ASC210422862_2021-11-14_AY.103
NY-CDC-ASC210490063_2021-10-21_AY.103
NY-CDC-ASC210324122_2021-10-01_AY.103
NY-URMC-21095103-1_2021-09-24_AY.103
NY-CDC-ASC210466064_2021-10-15_AY.103
NY-CDC-ASC210364358_2021-10-14_AY.103
NY-CDC-ASC210359049_2021-10-11_AY.103
NY-CDC-ASC210449288_2021-10-29_AY.103
NY-Wadsworth-21097673-01_2021-10-16_AY.103
NY-CDC-ASC210314256_2021-10-05_AY.103
NY-CDC-ASC210285708_2021-09-17_AY.103
NY-CDC-ASC210408808_2021-09-12_AY.103
OL995922_NY-CDC-QDX31823334_2021-12-02_AY.103
OL995920_NY-CDC-QDX31823333_2021-12-02_AY.103
OL996336_NY-CDC-ASC210530530_2021-12-08_AY.103
OL918400_NY-CDC-LC0408350_2021-12-01_AY.103
OL928566_NY-CDC-ASC210397712_2021-11-11_AY.103
OL759751_NY-CDC-ASC210397712_2021-11-11_AY.103
OM078160_NY-CDC-ASC210628919_2021-12-17_AY.103
OM085077_NY-CDC-QDX31922315_2021-12-08_AY.103
OL749460_NY-CDC-ASC210481201_2021-11-16_AY.103
OM034716_NY-CDC-ASC210533048_2021-12-10_AY.103
OL924080_NY-CDC-ASC210484744_2021-11-18_AY.103
OL948410_NY-CDC-ASC210526460_2021-11-06_AY.103
OL924079_NY-CDC-ASC210484743_2021-11-18_AY.103
OL918865_NY-CDC-LC0408087_2021-12-02_AY.103
MN908947 (Wuhan-Hu-1/2019)
0.2

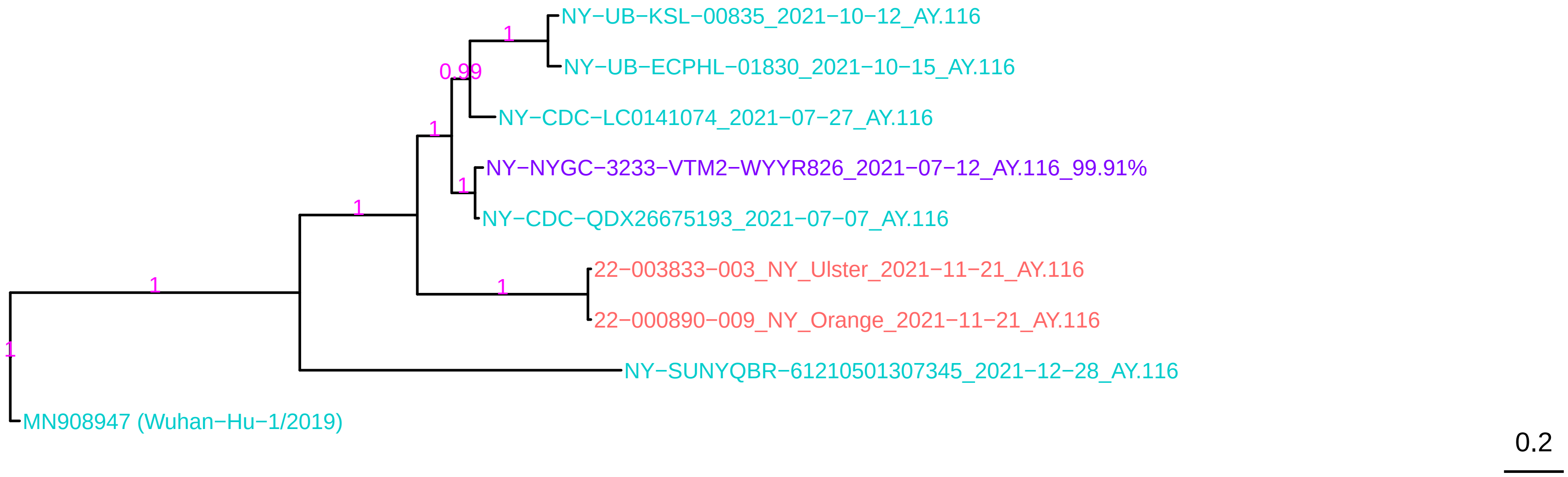
NY−UB−KSL−00835_2021−10−12_AY.116
NY−UB−ECPHL−01830_2021−10−15_AY.116
NY−CDC−LC0141074_2021−07−27_AY.116
NY−NYGC−3233−VTM2−WYYR826_2021−07−12_AY.116_99.91%
NY−CDC−QDX26675193_2021−07−07_AY.116
22−003833−003_NY_Ulster_2021−11−21_AY.116
22−000890−009_NY_Orange_2021−11−21_AY.116
NY−SUNYQBR−61210501307345_2021−12−28_AY.116
MN908947 (Wuhan−Hu−1/2019)
1
1
0.99
1
1
1
1
1
1
0.2

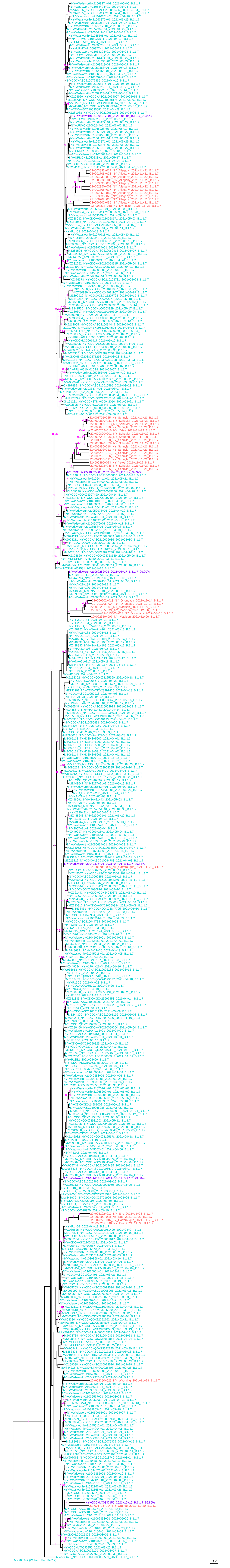

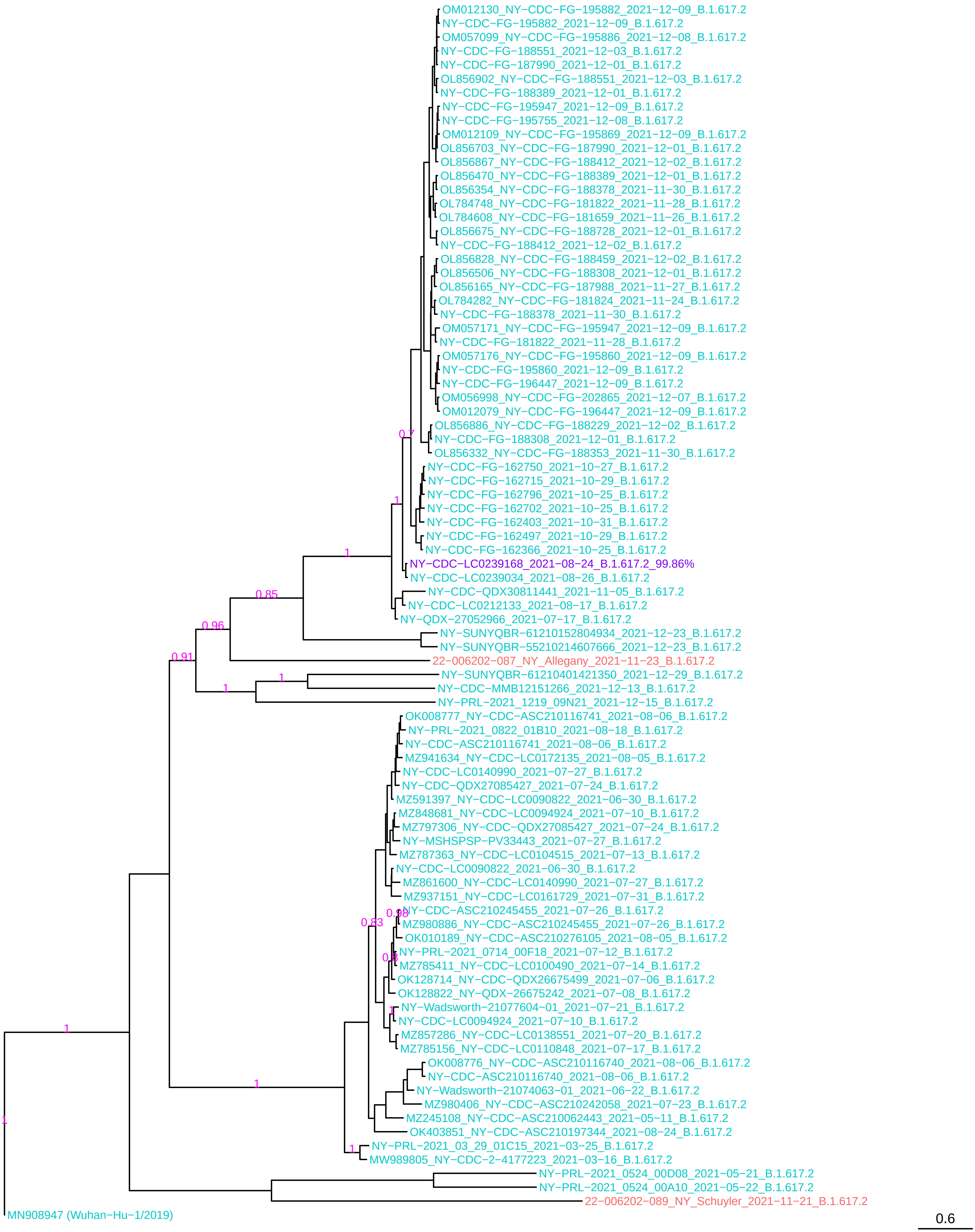
OM012130_NY-CDC-FG-195882_2021-12-09_B.1.617.2
NY-CDC-FG-195882_2021-12-09_B.1.617.2
OM057099_NY-CDC-FG-195886_2021-12-08_B.1.617.2
NY-CDC-FG-188551_2021-12-03_B.1.617.2
NY-CDC-FG-187990_2021-12-01_B.1.617.2
OL856902_NY-CDC-FG-188551_2021-12-03_B.1.617.2
NY-CDC-FG-188389_2021-12-01_B.1.617.2
NY-CDC-FG-195947_2021-12-09_B.1.617.2
NY-CDC-FG-195755_2021-12-08_B.1.617.2
OM012109_NY-CDC-FG-195869_2021-12-09_B.1.617.2
OL856703_NY-CDC-FG-187990_2021-12-01_B.1.617.2
OL856867_NY-CDC-FG-188412_2021-12-02_B.1.617.2
OL856470_NY-CDC-FG-188389_2021-12-01_B.1.617.2
OL856354_NY-CDC-FG-188378_2021-11-30_B.1.617.2
OL784748_NY-CDC-FG-181822_2021-11-28_B.1.617.2
OL784608_NY-CDC-FG-181659_2021-11-26_B.1.617.2
OL856675_NY-CDC-FG-188728_2021-12-01_B.1.617.2
NY-CDC-FG-188412_2021-12-02_B.1.617.2
OL856828_NY-CDC-FG-188459_2021-12-02_B.1.617.2
OL856506_NY-CDC-FG-188308_2021-12-01_B.1.617.2
OL856165_NY-CDC-FG-187988_2021-11-27_B.1.617.2
OL784282_NY-CDC-FG-181824_2021-11-24_B.1.617.2
NY-CDC-FG-188378_2021-11-30_B.1.617.2
OM057171_NY-CDC-FG-195947_2021-12-09_B.1.617.2
NY-CDC-FG-181822_2021-11-28_B.1.617.2
OM057176_NY-CDC-FG-195860_2021-12-09_B.1.617.2
NY-CDC-FG-195860_2021-12-09_B.1.617.2
NY-CDC-FG-196447_2021-12-09_B.1.617.2
OM056998_NY-CDC-FG-202865_2021-12-07_B.1.617.2
OM012079_NY-CDC-FG-196447_2021-12-09_B.1.617.2
OL856886_NY-CDC-FG-188229_2021-12-02_B.1.617.2
NY-CDC-FG-188308_2021-12-01_B.1.617.2
0.7
OL856332_NY-CDC-FG-188353_2021-11-30_B.1.617.2
NY-CDC-FG-162750_2021-10-27_B.1.617.2
NY-CDC-FG-162715_2021-10-29_B.1.617.2
NY-CDC-FG-162796_2021-10-25_B.1.617.2
1
NY-CDC-FG-162702_2021-10-25_B.1.617.2
NY-CDC-FG-162403_2021-10-31_B.1.617.2
NY-CDC-FG-162497_2021-10-29_B.1.617.2
1
NY-CDC-FG-162366_2021-10-25_B.1.617.2
NY-CDC-LC0239168_2021-08-24_B.1.617.2_99.86%
NY-CDC-LC0239034_2021-08-26_B.1.617.2
0.85
NY-CDC-QDX30811441_2021-11-05_B.1.617.2
NY-CDC-LC0212133_2021-08-17_B.1.617.2
0.96
NY-QDX-27052966_2021-07-17_B.1.617.2
NY-SUNYQBR-61210152804934_2021-12-23_B.1.617.2
NY-SUNYQBR-55210214607666_2021-12-23_B.1.617.2
0.91
22-006202-087_NY_Allegany_2021-11-23_B.1.617.2
1
NY-SUNYQBR-61210401421350_2021-12-29_B.1.617.2
1
NY-CDC-MMB12151266_2021-12-13_B.1.617.2
NY-PRL-2021_1219_09N21_2021-12-15_B.1.617.2
OK008777_NY-CDC-ASC210116741_2021-08-06_B.1.617.2
NY-PRL-2021_0822_01B10_2021-08-18_B.1.617.2
NY-CDC-ASC210116741_2021-08-06_B.1.617.2
MZ941634_NY-CDC-LC0172135_2021-08-05_B.1.617.2
NY-CDC-LC0140990_2021-07-27_B.1.617.2
NY-CDC-QDX27085427_2021-07-24_B.1.617.2
MZ591397_NY-CDC-LC0090822_2021-06-30_B.1.617.2
MZ848681_NY-CDC-LC0094924_2021-07-10_B.1.617.2
MZ797306_NY-CDC-QDX27085427_2021-07-24_B.1.617.2
NY-MSHSPSP-PV33443_2021-07-27_B.1.617.2
MZ787363_NY-CDC-LC0104515_2021-07-13_B.1.617.2
NY-CDC-LC0090822_2021-06-30_B.1.617.2
MZ861600_NY-CDC-LC0140990_2021-07-27_B.1.617.2
MZ937151_NY-CDC-LC0161729_2021-07-31_B.1.617.2
0.98
NY-CDC-ASC210245455_2021-07-26_B.1.617.2
0.83
MZ980886_NY-CDC-ASC210245455_2021-07-26_B.1.617.2
OK010189_NY-CDC-ASC210276105_2021-08-05_B.1.617.2
0.8
NY-PRL-2021_0714_00F18_2021-07-12_B.1.617.2
MZ785411_NY-CDC-LC0100490_2021-07-14_B.1.617.2
OK128714_NY-CDC-QDX26675499_2021-07-06_B.1.617.2
OK128822_NY-QDX-26675242_2021-07-08_B.1.617.2
1
NY-Wadsworth-21077604-01_2021-07-21_B.1.617.2
NY-CDC-LC0094924_2021-07-10_B.1.617.2
1
MZ857286_NY-CDC-LC0138551_2021-07-20_B.1.617.2
MZ785156_NY-CDC-LC0110848_2021-07-17_B.1.617.2
OK008776_NY-CDC-ASC210116740_2021-08-06_B.1.617.2
NY-CDC-ASC210116740_2021-08-06_B.1.617.2
1
NY-Wadsworth-21074063-01_2021-06-22_B.1.617.2
MZ980406_NY-CDC-ASC210242058_2021-07-23_B.1.617.2
1
MZ245108_NY-CDC-ASC210062443_2021-05-11_B.1.617.2
OK403851_NY-CDC-ASC210197344_2021-08-24_B.1.617.2
1
NY-PRL-2021_03_29_01C15_2021-03-25_B.1.617.2
MW989805_NY-CDC-2-4177223_2021-03-16_B.1.617.2
NY-PRL-2021_0524_00D08_2021-05-21_B.1.617.2
NY-PRL-2021_0524_00A10_2021-05-22_B.1.617.2
22-006202-089_NY_Schuyler_2021-11-21_B.1.617.2
MN908947 (Wuhan-Hu-1/2019)
0.6

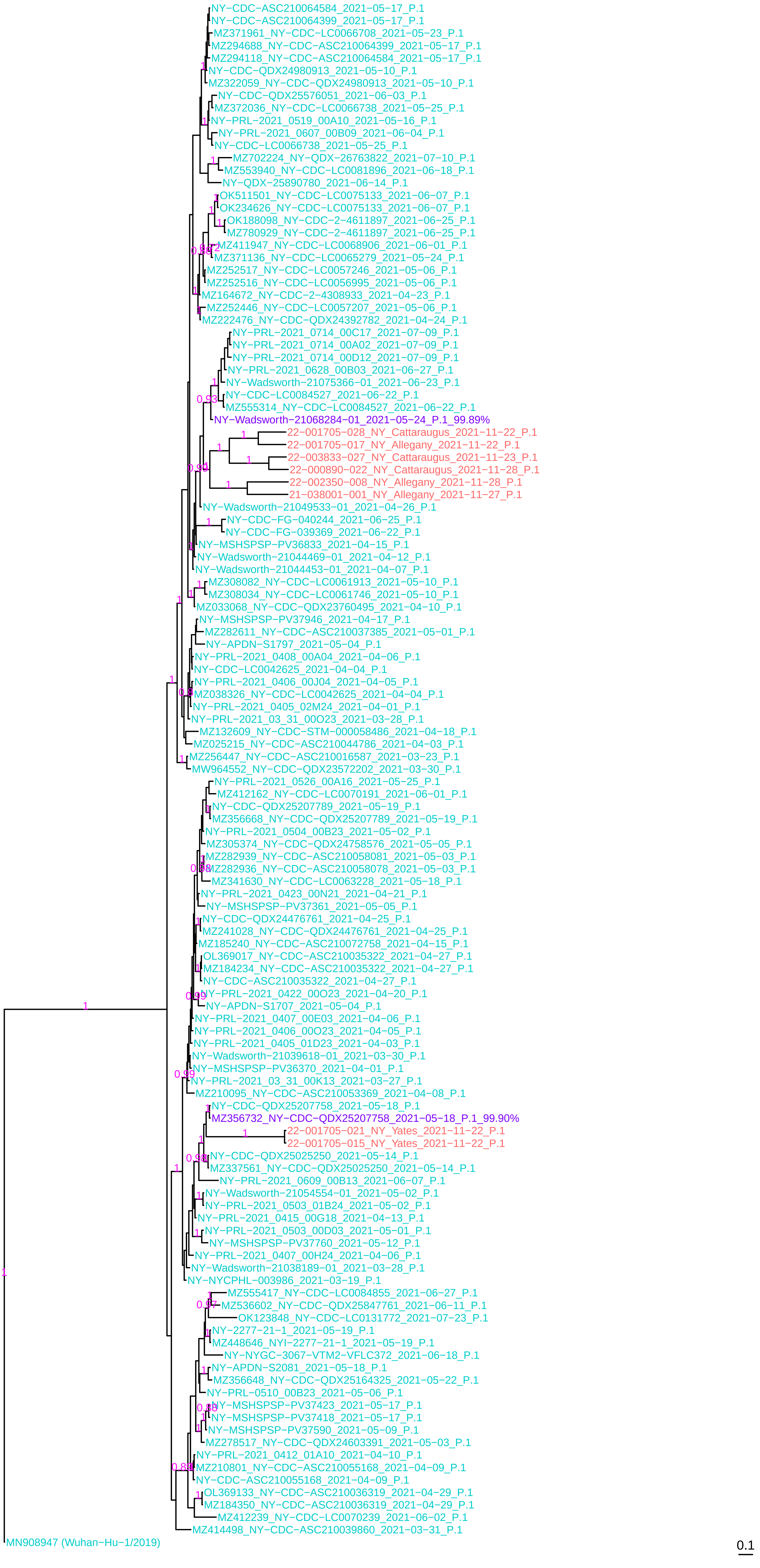
NY-CDC-ASC210064584_2021-05-17_P.1
NY-CDC-ASC210064399_2021-05-17_P.1
MZ371961_NY-CDC-LC0066708_2021-05-23_P.1
MZ294688_NY-CDC-ASC210064399_2021-05-17_P.1
MZ294118_NY-CDC-ASC210064584_2021-05-17_P.1
NY-CDC-QDX24980913_2021-05-10_P.1
MZ322059_NY-CDC-QDX24980913_2021-05-10_P.1
NY-CDC-QDX25576051_2021-06-03_P.1
MZ372036_NY-CDC-LC0066738_2021-05-25_P.1
NY-PRL-2021_0519_00A10_2021-05-16_P.1
NY-PRL-2021_0607_00B09_2021-06-04_P.1
NY-CDC-LC0066738_2021-05-25_P.1
MZ702224_NY-QDX-26763822_2021-07-10_P.1
MZ553940_NY-CDC-LC0081896_2021-06-18_P.1
NY-QDX-25890780_2021-06-14_P.1
OK511501_NY-CDC-LC0075133_2021-06-07_P.1
OK234626_NY-CDC-LC0075133_2021-06-07_P.1
OK188098_NY-CDC-2-4611897_2021-06-25_P.1
MZ780929_NY-CDC-2-4611897_2021-06-25_P.1
MZ411947_NY-CDC-LC0068906_2021-06-01_P.1
MZ371136_NY-CDC-LC0065279_2021-05-24_P.1
MZ252517_NY-CDC-LC0057246_2021-05-06_P.1
MZ252516_NY-CDC-LC0056995_2021-05-06_P.1
MZ164672_NY-CDC-2-4308933_2021-04-23_P.1
MZ252446_NY-CDC-LC0057207_2021-05-06_P.1
MZ222476_NY-CDC-QDX24392782_2021-04-24_P.1
NY-PRL-2021_0714_00C17_2021-07-09_P.1
NY-PRL-2021_0714_00A02_2021-07-09_P.1
NY-PRL-2021_0714_00D12_2021-07-09_P.1
NY-PRL-2021_0628_00B03_2021-06-27_P.1
NY-Wadsworth-21075366-01_2021-06-23_P.1
NY-CDC-LC0084527_2021-06-22_P.1
MZ555314_NY-CDC-LC0084527_2021-06-22_P.1
NY-Wadsworth-21068284-01_2021-05-24_P.1_99.89%
22-001705-028_NY_Cattaraugus_2021-11-22_P.1
22-001705-017_NY_Allegany_2021-11-22_P.1
22-003833-027_NY_Cattaraugus_2021-11-23_P.1
22-000890-022_NY_Cattaraugus_2021-11-28_P.1
22-002350-008_NY_Allegany_2021-11-28_P.1
21-038001-001_NY_Allegany_2021-11-27_P.1
NY-Wadsworth-21049533-01_2021-04-26_P.1
NY-CDC-FG-040244_2021-06-25_P.1
NY-CDC-FG-039369_2021-06-22_P.1
NY-MSHSPSP-PV36833_2021-04-15_P.1
NY-Wadsworth-21044469-01_2021-04-12_P.1
NY-Wadsworth-21044453-01_2021-04-07_P.1
MZ308082_NY-CDC-LC0061913_2021-05-10_P.1
MZ308034_NY-CDC-LC0061746_2021-05-10_P.1
MZ033068_NY-CDC-QDX23760495_2021-04-10_P.1
NY-MSHSPSP-PV37946_2021-04-17_P.1
MZ282611_NY-CDC-ASC210037385_2021-05-01_P.1
NY-APDN-S1797_2021-05-04_P.1
NY-PRL-2021_0408_00A04_2021-04-06_P.1
NY-CDC-LC0042625_2021-04-04_P.1
NY-PRL-2021_0406_00J04_2021-04-05_P.1
MZ038326_NY-CDC-LC0042625_2021-04-04_P.1
NY-PRL-2021_0405_02M24_2021-04-01_P.1
NY-PRL-2021_03_31_00O23_2021-03-28_P.1
MZ132609_NY-CDC-STM-000058486_2021-04-18_P.1
MZ025215_NY-CDC-ASC210044786_2021-04-03_P.1
MZ256447_NY-CDC-ASC210016587_2021-03-23_P.1
MW964552_NY-CDC-QDX23572202_2021-03-30_P.1
NY-PRL-2021_0526_00A16_2021-05-25_P.1
MZ412162_NY-CDC-LC0070191_2021-06-01_P.1
NY-CDC-QDX25207789_2021-05-19_P.1
MZ356668_NY-CDC-QDX25207789_2021-05-19_P.1
NY-PRL-2021_0504_00B23_2021-05-02_P.1
MZ305374_NY-CDC-QDX24758576_2021-05-05_P.1
MZ282939_NY-CDC-ASC210058081_2021-05-03_P.1
MZ282936_NY-CDC-ASC210058078_2021-05-03_P.1
MZ341630_NY-CDC-LC0063228_2021-05-18_P.1
NY-PRL-2021_0423_00N21_2021-04-21_P.1
NY-MSHSPSP-PV37361_2021-05-05_P.1
NY-CDC-QDX24476761_2021-04-25_P.1
MZ241028_NY-CDC-QDX24476761_2021-04-25_P.1
MZ185240_NY-CDC-ASC210072758_2021-04-15_P.1
OL369017_NY-CDC-ASC210035322_2021-04-27_P.1
MZ184234_NY-CDC-ASC210035322_2021-04-27_P.1
NY-CDC-ASC210035322_2021-04-27_P.1
NY-PRL-2021_0422_00O23_2021-04-20_P.1
NY-APDN-S1707_2021-05-04_P.1
NY-PRL-2021_0407_00E03_2021-04-06_P.1
NY-PRL-2021_0406_00O23_2021-04-05_P.1
NY-PRL-2021_0405_01D23_2021-04-03_P.1
NY-Wadsworth-21039618-01_2021-03-30_P.1
NY-MSHSPSP-PV36370_2021-04-01_P.1
NY-PRL-2021_03_31_00K13_2021-03-27_P.1
MZ210095_NY-CDC-ASC210053369_2021-04-08_P.1
NY-CDC-QDX25207758_2021-05-18_P.1
MZ356732_NY-CDC-QDX25207758_2021-05-18_P.1_99.90%
22-001705-021_NY_Yates_2021-11-22_P.1
22-001705-015_NY_Yates_2021-11-22_P.1
NY-CDC-QDX25025250_2021-05-14_P.1
MZ337561_NY-CDC-QDX25025250_2021-05-14_P.1
NY-PRL-2021_0609_00B13_2021-06-07_P.1
NY-Wadsworth-21054554-01_2021-05-02_P.1
NY-PRL-2021_0503_01B24_2021-05-02_P.1
NY-PRL-2021_0415_00G18_2021-04-13_P.1
NY-PRL-2021_0503_00D03_2021-05-01_P.1
NY-MSHSPSP-PV37760_2021-05-12_P.1
NY-PRL-2021_0407_00H24_2021-04-06_P.1
NY-Wadsworth-21038189-01_2021-03-28_P.1
NY-NYCPHL-003986_2021-03-19_P.1
MZ555417_NY-CDC-LC0084855_2021-06-27_P.1
MZ536602_NY-CDC-QDX25847761_2021-06-11_P.1
OK123848_NY-CDC-LC0131772_2021-07-23_P.1
NY-2277-21-1_2021-05-19_P.1
MZ448646_NYI-2277-21-1_2021-05-19_P.1
NY-NYGC-3067-VTM2-VFLC372_2021-06-18_P.1
NY-APDN-S2081_2021-05-18_P.1
MZ356648_NY-CDC-QDX25164325_2021-05-22_P.1
NY-PRL-0510_00B23_2021-05-06_P.1
NY-MSHSPSP-PV37423_2021-05-17_P.1
NY-MSHSPSP-PV37418_2021-05-17_P.1
NY-MSHSPSP-PV37590_2021-05-09_P.1
MZ278517_NY-CDC-QDX24603391_2021-05-03_P.1
NY-PRL-2021_0412_01A10_2021-04-10_P.1
MZ210801_NY-CDC-ASC210055168_2021-04-09_P.1
NY-CDC-ASC210055168_2021-04-09_P.1
OL369133_NY-CDC-ASC210036319_2021-04-29_P.1
MZ184350_NY-CDC-ASC210036319_2021-04-29_P.1
MZ412239_NY-CDC-LC0070239_2021-06-02_P.1
MZ414498_NY-CDC-ASC210039860_2021-03-31_P.1
MN908947 (Wuhan-Hu-1/2019)
0.1

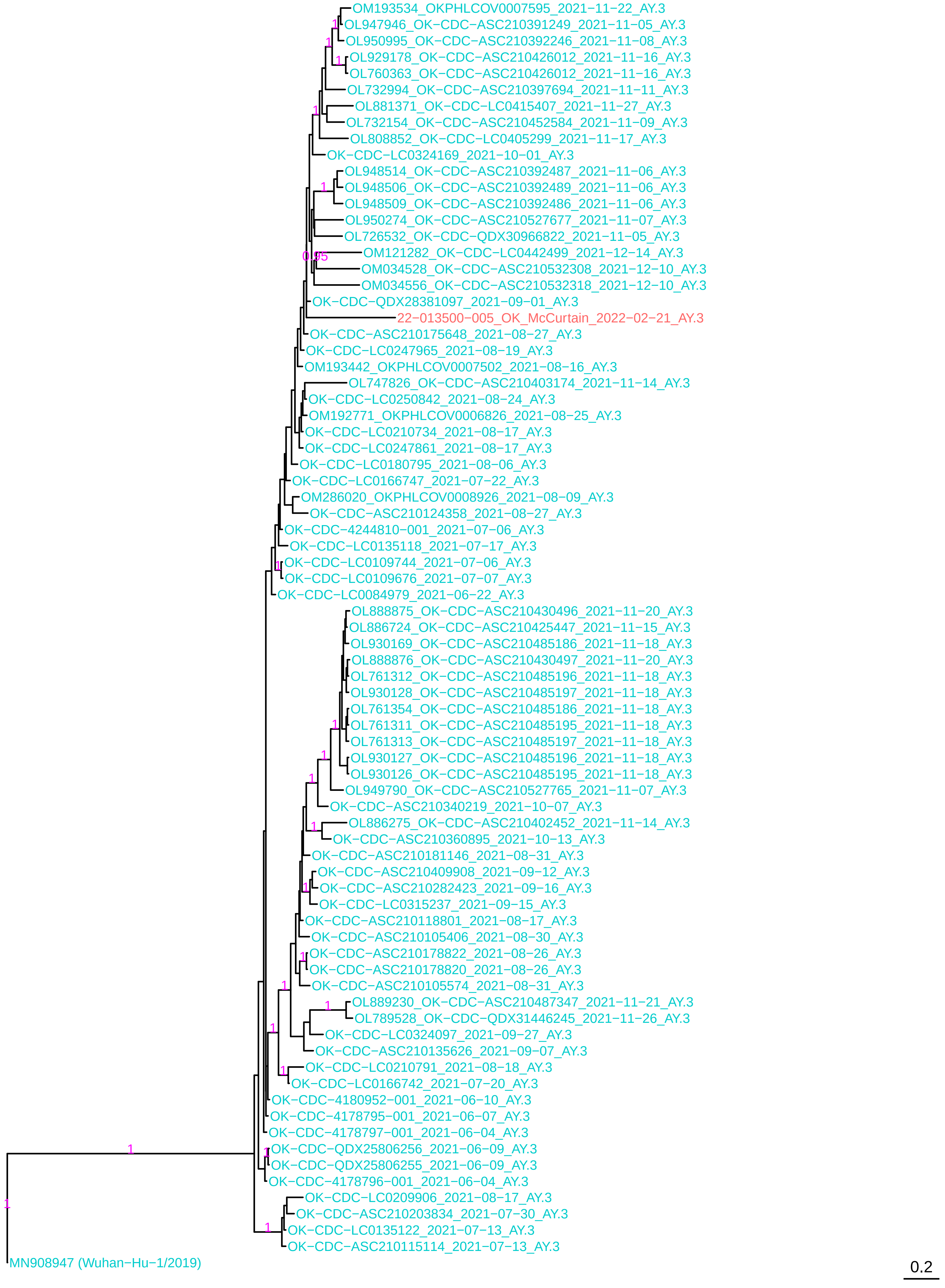
OM193534_OKPHLCOV0007595_2021−11−22_AY.3
OL947946_OK−CDC−ASC210391249_2021−11−05_AY.3
OL950995_OK−CDC−ASC210392246_2021−11−08_AY.3
OL929178_OK−CDC−ASC210426012_2021−11−16_AY.3
OL760363_OK−CDC−ASC210426012_2021−11−16_AY.3
OL732994_OK−CDC−ASC210397694_2021−11−11_AY.3
OL881371_OK−CDC−LC0415407_2021−11−27_AY.3
OL732154_OK−CDC−ASC210452584_2021−11−09_AY.3
OL808852_OK−CDC−LC0405299_2021−11−17_AY.3
OK−CDC−LC0324169_2021−10−01_AY.3
OL948514_OK−CDC−ASC210392487_2021−11−06_AY.3
OL948506_OK−CDC−ASC210392489_2021−11−06_AY.3
OL948509_OK−CDC−ASC210392486_2021−11−06_AY.3
OL950274_OK−CDC−ASC210527677_2021−11−07_AY.3
OL726532_OK−CDC−QDX30966822_2021−11−05_AY.3
OM121282_OK−CDC−LC0442499_2021−12−14_AY.3
OM034528_OK−CDC−ASC210532308_2021−12−10_AY.3
OM034556_OK−CDC−ASC210532318_2021−12−10_AY.3
OK−CDC−QDX28381097_2021−09−01_AY.3
22−013500−005_OK_McCurtain_2022−02−21_AY.3
OK−CDC−ASC210175648_2021−08−27_AY.3
OK−CDC−LC0247965_2021−08−19_AY.3
OM193442_OKPHLCOV0007502_2021−08−16_AY.3
OL747826_OK−CDC−ASC210403174_2021−11−14_AY.3
OK−CDC−LC0250842_2021−08−24_AY.3
OM192771_OKPHLCOV0006826_2021−08−25_AY.3
OK−CDC−LC0210734_2021−08−17_AY.3
OK−CDC−LC0247861_2021−08−17_AY.3
OK−CDC−LC0180795_2021−08−06_AY.3
OK−CDC−LC0166747_2021−07−22_AY.3
OM286020_OKPHLCOV0008926_2021−08−09_AY.3
OK−CDC−ASC210124358_2021−08−27_AY.3
OK−CDC−4244810−001_2021−07−06_AY.3
OK−CDC−LC0135118_2021−07−17_AY.3
OK−CDC−LC0109744_2021−07−06_AY.3
OK−CDC−LC0109676_2021−07−07_AY.3
OK−CDC−LC0084979_2021−06−22_AY.3
OL888875_OK−CDC−ASC210430496_2021−11−20_AY.3
OL886724_OK−CDC−ASC210425447_2021−11−15_AY.3
OL930169_OK−CDC−ASC210485186_2021−11−18_AY.3
OL888876_OK−CDC−ASC210430497_2021−11−20_AY.3
OL761312_OK−CDC−ASC210485196_2021−11−18_AY.3
OL930128_OK−CDC−ASC210485197_2021−11−18_AY.3
OL761354_OK−CDC−ASC210485186_2021−11−18_AY.3
OL761311_OK−CDC−ASC210485195_2021−11−18_AY.3
OL761313_OK−CDC−ASC210485197_2021−11−18_AY.3
OL930127_OK−CDC−ASC210485196_2021−11−18_AY.3
OL930126_OK−CDC−ASC210485195_2021−11−18_AY.3
OL949790_OK−CDC−ASC210527765_2021−11−07_AY.3
OK−CDC−ASC210340219_2021−10−07_AY.3
OL886275_OK−CDC−ASC210402452_2021−11−14_AY.3
OK−CDC−ASC210360895_2021−10−13_AY.3
OK−CDC−ASC210181146_2021−08−31_AY.3
OK−CDC−ASC210409908_2021−09−12_AY.3
OK−CDC−ASC210282423_2021−09−16_AY.3
OK−CDC−LC0315237_2021−09−15_AY.3
OK−CDC−ASC210118801_2021−08−17_AY.3
OK−CDC−ASC210105406_2021−08−30_AY.3
OK−CDC−ASC210178822_2021−08−26_AY.3
OK−CDC−ASC210178820_2021−08−26_AY.3
OK−CDC−ASC210105574_2021−08−31_AY.3
OL889230_OK−CDC−ASC210487347_2021−11−21_AY.3
OL789528_OK−CDC−QDX31446245_2021−11−26_AY.3
OK−CDC−LC0324097_2021−09−27_AY.3
OK−CDC−ASC210135626_2021−09−07_AY.3
OK−CDC−LC0210791_2021−08−18_AY.3
OK−CDC−LC0166742_2021−07−20_AY.3
OK−CDC−4180952−001_2021−06−10_AY.3
OK−CDC−4178795−001_2021−06−07_AY.3
OK−CDC−4178797−001_2021−06−04_AY.3
OK−CDC−QDX25806256_2021−06−09_AY.3
OK−CDC−QDX25806255_2021−06−09_AY.3
OK−CDC−4178796−001_2021−06−04_AY.3
OK−CDC−LC0209906_2021−08−17_AY.3
OK−CDC−ASC210203834_2021−07−30_AY.3
OK−CDC−LC0135122_2021−07−13_AY.3
OK−CDC−ASC210115114_2021−07−13_AY.3
MN908947 (Wuhan−Hu−1/2019)
0.2

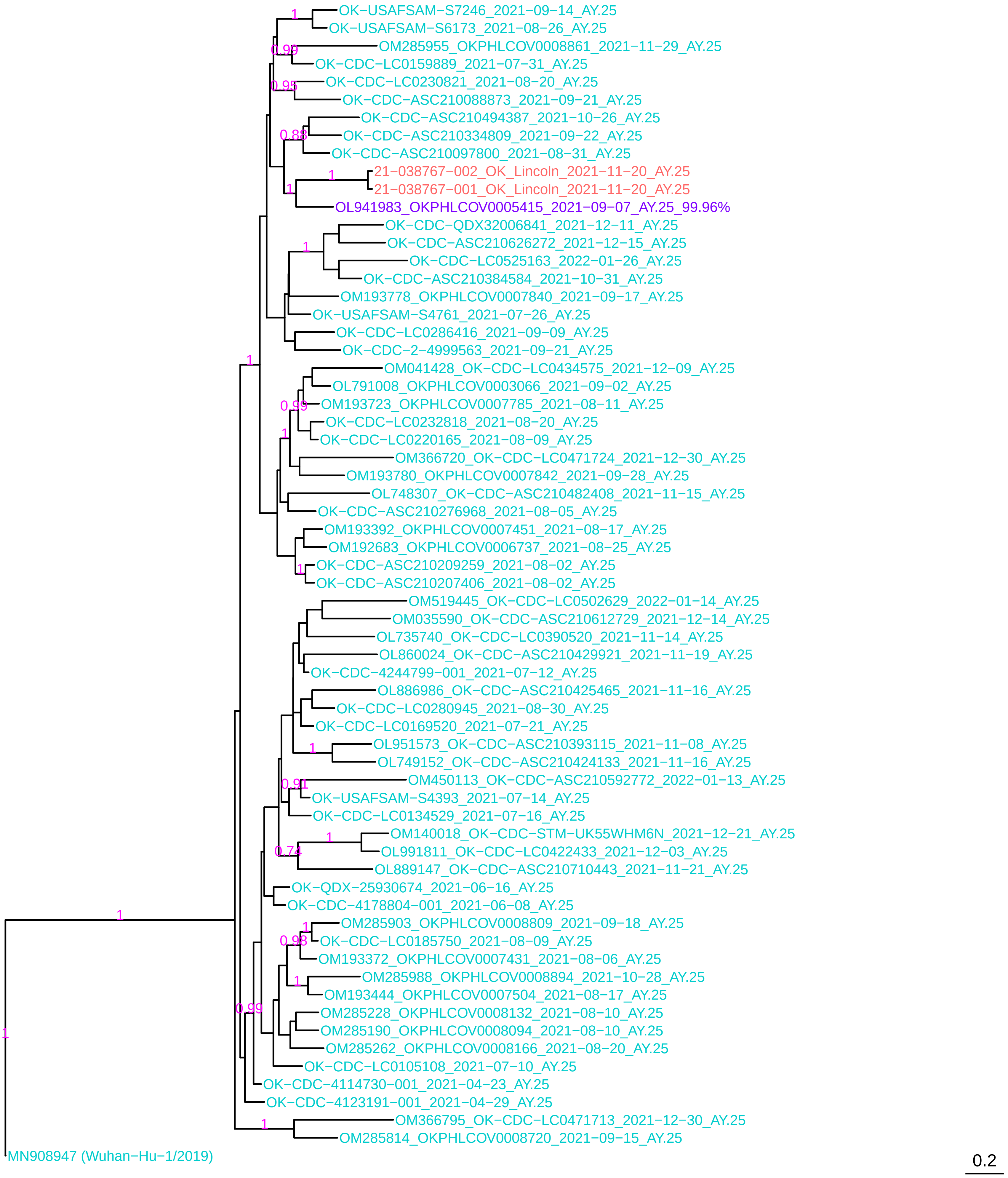

OK−USAFSAM−S7246_2021−09−14_AY.25
OK−USAFSAM−S6173_2021−08−26_AY.25
OM285955_OKPHLCOV0008861_2021−11−29_AY.25
OK−CDC−LC0159889_2021−07−31_AY.25
OK−CDC−LC0230821_2021−08−20_AY.25
OK−CDC−ASC210088873_2021−09−21_AY.25
OK−CDC−ASC210494387_2021−10−26_AY.25
OK−CDC−ASC210334809_2021−09−22_AY.25
OK−CDC−ASC210097800_2021−08−31_AY.25
21−038767−002_OK_Lincoln_2021−11−20_AY.25
21−038767−001_OK_Lincoln_2021−11−20_AY.25
OL941983_OKPHLCOV0005415_2021−09−07_AY.25_99.96%
OK−CDC−QDX32006841_2021−12−11_AY.25
OK−CDC−ASC210626272_2021−12−15_AY.25
OK−CDC−LC0525163_2022−01−26_AY.25
OK−CDC−ASC210384584_2021−10−31_AY.25
OM193778_OKPHLCOV0007840_2021−09−17_AY.25
OK−USAFSAM−S4761_2021−07−26_AY.25
OK−CDC−LC0286416_2021−09−09_AY.25
OK−CDC−2−4999563_2021−09−21_AY.25
OM041428_OK−CDC−LC0434575_2021−12−09_AY.25
OL791008_OKPHLCOV0003066_2021−09−02_AY.25
OM193723_OKPHLCOV0007785_2021−08−11_AY.25
OK−CDC−LC0232818_2021−08−20_AY.25
OK−CDC−LC0220165_2021−08−09_AY.25
OM366720_OK−CDC−LC0471724_2021−12−30_AY.25
OM193780_OKPHLCOV0007842_2021−09−28_AY.25
OL748307_OK−CDC−ASC210482408_2021−11−15_AY.25
OK−CDC−ASC210276968_2021−08−05_AY.25
OM193392_OKPHLCOV0007451_2021−08−17_AY.25
OM192683_OKPHLCOV0006737_2021−08−25_AY.25
OK−CDC−ASC210209259_2021−08−02_AY.25
OK−CDC−ASC210207406_2021−08−02_AY.25
OM519445_OK−CDC−LC0502629_2022−01−14_AY.25
OM035590_OK−CDC−ASC210612729_2021−12−14_AY.25
OL735740_OK−CDC−LC0390520_2021−11−14_AY.25
OL860024_OK−CDC−ASC210429921_2021−11−19_AY.25
OK−CDC−4244799−001_2021−07−12_AY.25
OL886986_OK−CDC−ASC210425465_2021−11−16_AY.25
OK−CDC−LC0280945_2021−08−30_AY.25
OK−CDC−LC0169520_2021−07−21_AY.25
OL951573_OK−CDC−ASC210393115_2021−11−08_AY.25
OL749152_OK−CDC−ASC210424133_2021−11−16_AY.25
OM450113_OK−CDC−ASC210592772_2022−01−13_AY.25
OK−USAFSAM−S4393_2021−07−14_AY.25
OK−CDC−LC0134529_2021−07−16_AY.25
OM140018_OK−CDC−STM−UK55WHM6N_2021−12−21_AY.25
OL991811_OK−CDC−LC0422433_2021−12−03_AY.25
OL889147_OK−CDC−ASC210710443_2021−11−21_AY.25
OK−QDX−25930674_2021−06−16_AY.25
OK−CDC−4178804−001_2021−06−08_AY.25
OM285903_OKPHLCOV0008809_2021−09−18_AY.25
OK−CDC−LC0185750_2021−08−09_AY.25
OM193372_OKPHLCOV0007431_2021−08−06_AY.25
OM285988_OKPHLCOV0008894_2021−10−28_AY.25
OM193444_OKPHLCOV0007504_2021−08−17_AY.25
OM285228_OKPHLCOV0008132_2021−08−10_AY.25
OM285190_OKPHLCOV0008094_2021−08−10_AY.25
OM285262_OKPHLCOV0008166_2021−08−20_AY.25
OK−CDC−LC0105108_2021−07−10_AY.25
OK−CDC−4114730−001_2021−04−23_AY.25
OK−CDC−4123191−001_2021−04−29_AY.25
OM366795_OK−CDC−LC0471713_2021−12−30_AY.25
OM285814_OKPHLCOV0008720_2021−09−15_AY.25
MN908947 (Wuhan−Hu−1/2019)
0.2

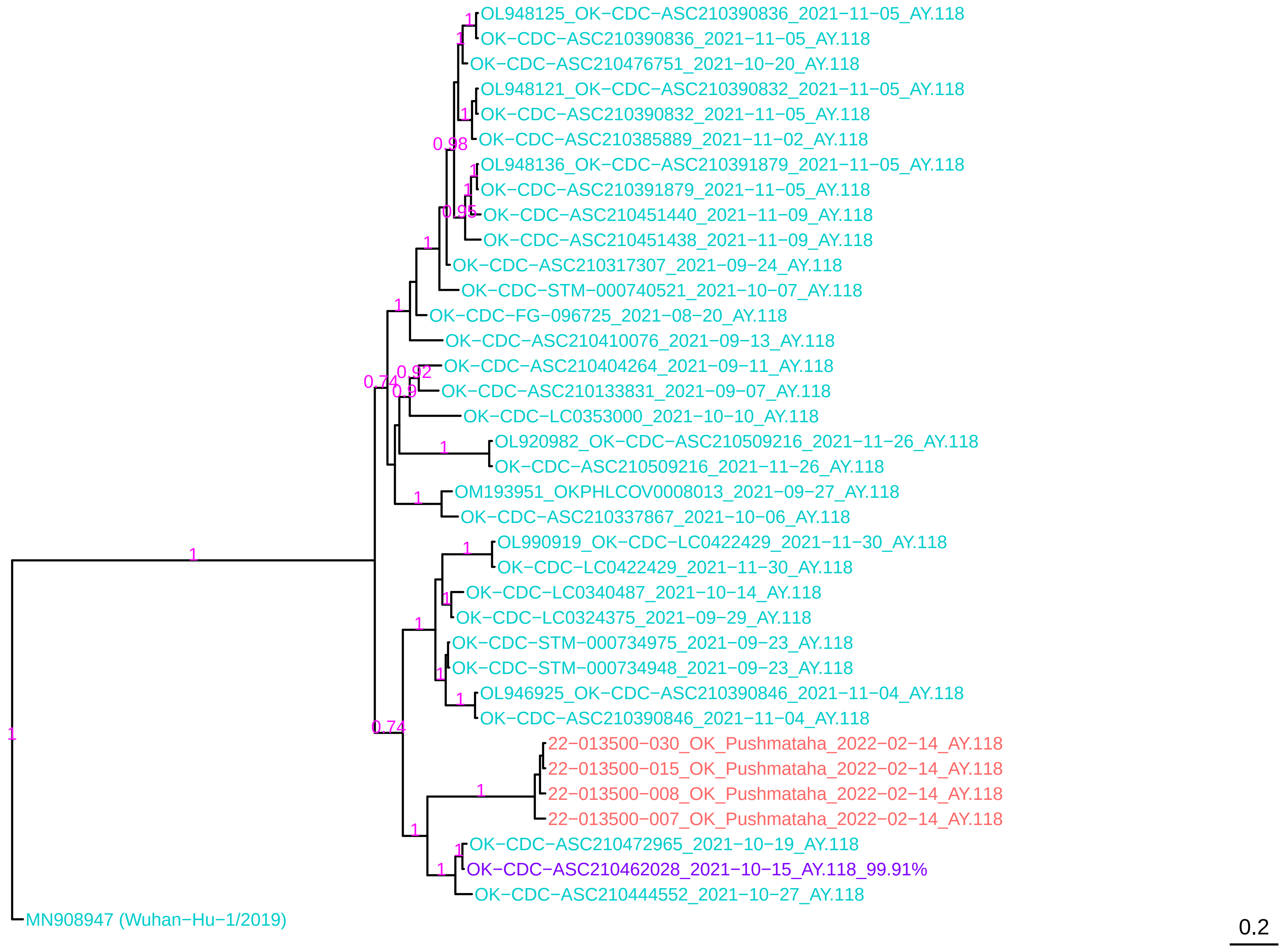
OL948125_OK−CDC−ASC210390836_2021−11−05_AY.118
OK−CDC−ASC210390836_2021−11−05_AY.118
OK−CDC−ASC210476751_2021−10−20_AY.118
OL948121_OK−CDC−ASC210390832_2021−11−05_AY.118
OK−CDC−ASC210390832_2021−11−05_AY.118
OK−CDC−ASC210385889_2021−11−02_AY.118
OL948136_OK−CDC−ASC210391879_2021−11−05_AY.118
OK−CDC−ASC210391879_2021−11−05_AY.118
OK−CDC−ASC210451440_2021−11−09_AY.118
OK−CDC−ASC210451438_2021−11−09_AY.118
OK−CDC−ASC210317307_2021−09−24_AY.118
OK−CDC−STM−000740521_2021−10−07_AY.118
OK−CDC−FG−096725_2021−08−20_AY.118
OK−CDC−ASC210410076_2021−09−13_AY.118
OK−CDC−ASC210404264_2021−09−11_AY.118
OK−CDC−ASC210133831_2021−09−07_AY.118
OK−CDC−LC0353000_2021−10−10_AY.118
OL920982_OK−CDC−ASC210509216_2021−11−26_AY.118
OK−CDC−ASC210509216_2021−11−26_AY.118
OM193951_OKPHLCOV0008013_2021−09−27_AY.118
OK−CDC−ASC210337867_2021−10−06_AY.118
OL990919_OK−CDC−LC0422429_2021−11−30_AY.118
OK−CDC−LC0422429_2021−11−30_AY.118
OK−CDC−LC0340487_2021−10−14_AY.118
OK−CDC−LC0324375_2021−09−29_AY.118
OK−CDC−STM−000734975_2021−09−23_AY.118
OK−CDC−STM−000734948_2021−09−23_AY.118
OL946925_OK−CDC−ASC210390846_2021−11−04_AY.118
OK−CDC−ASC210390846_2021−11−04_AY.118
22−013500−030_OK_Pushmataha_2022−02−14_AY.118
22−013500−015_OK_Pushmataha_2022−02−14_AY.118
22−013500−008_OK_Pushmataha_2022−02−14_AY.118
22−013500−007_OK_Pushmataha_2022−02−14_AY.118
OK−CDC−ASC210472965_2021−10−19_AY.118
OK−CDC−ASC210462028_2021−10−15_AY.118_99.91%
OK−CDC−ASC210444552_2021−10−27_AY.118
MN908947 (Wuhan−Hu−1/2019)
0.98
0.95
0.92
0.74
0.9
0.74
0.2

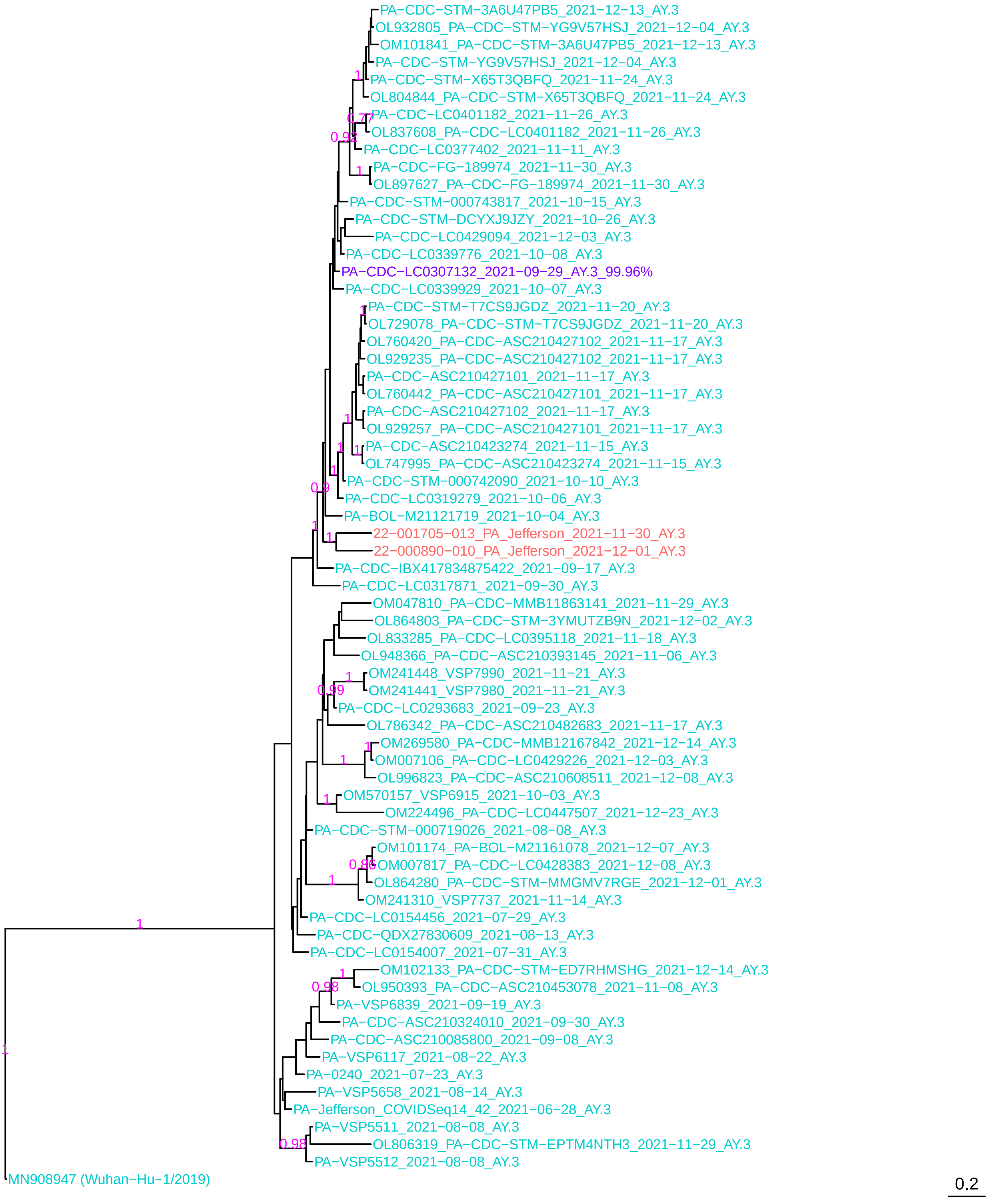
PA−CDC−STM−3A6U47PB5_2021−12−13_AY.3
OL932805_PA−CDC−STM−YG9V57HSJ_2021−12−04_AY.3
OM101841_PA−CDC−STM−3A6U47PB5_2021−12−13_AY.3
PA−CDC−STM−YG9V57HSJ_2021−12−04_AY.3
PA−CDC−STM−X65T3QBFQ_2021−11−24_AY.3
OL804844_PA−CDC−STM−X65T3QBFQ_2021−11−24_AY.3
PA−CDC−LC0401182_2021−11−26_AY.3
OL837608_PA−CDC−LC0401182_2021−11−26_AY.3
PA−CDC−LC0377402_2021−11−11_AY.3
PA−CDC−FG−189974_2021−11−30_AY.3
OL897627_PA−CDC−FG−189974_2021−11−30_AY.3
PA−CDC−STM−000743817_2021−10−15_AY.3
PA−CDC−STM−DCYXJ9JZY_2021−10−26_AY.3
PA−CDC−LC0429094_2021−12−03_AY.3
PA−CDC−LC0339776_2021−10−08_AY.3
PA−CDC−LC0307132_2021−09−29_AY.3_99.96%
PA−CDC−LC0339929_2021−10−07_AY.3
PA−CDC−STM−T7CS9JGDZ_2021−11−20_AY.3
OL729078_PA−CDC−STM−T7CS9JGDZ_2021−11−20_AY.3
OL760420_PA−CDC−ASC210427102_2021−11−17_AY.3
OL929235_PA−CDC−ASC210427102_2021−11−17_AY.3
PA−CDC−ASC210427101_2021−11−17_AY.3
OL760442_PA−CDC−ASC210427101_2021−11−17_AY.3
PA−CDC−ASC210427102_2021−11−17_AY.3
OL929257_PA−CDC−ASC210427101_2021−11−17_AY.3
PA−CDC−ASC210423274_2021−11−15_AY.3
OL747995_PA−CDC−ASC210423274_2021−11−15_AY.3
PA−CDC−STM−000742090_2021−10−10_AY.3
PA−CDC−LC0319279_2021−10−06_AY.3
PA−BOL−M21121719_2021−10−04_AY.3
22−001705−013_PA_Jefferson_2021−11−30_AY.3
22−000890−010_PA_Jefferson_2021−12−01_AY.3
PA−CDC−IBX417834875422_2021−09−17_AY.3
PA−CDC−LC0317871_2021−09−30_AY.3
OM047810_PA−CDC−MMB11863141_2021−11−29_AY.3
OL864803_PA−CDC−STM−3YMUTZB9N_2021−12−02_AY.3
OL833285_PA−CDC−LC0395118_2021−11−18_AY.3
OL948366_PA−CDC−ASC210393145_2021−11−06_AY.3
OM241448_VSP7990_2021−11−21_AY.3
OM241441_VSP7980_2021−11−21_AY.3
PA−CDC−LC0293683_2021−09−23_AY.3
OL786342_PA−CDC−ASC210482683_2021−11−17_AY.3
OM269580_PA−CDC−MMB12167842_2021−12−14_AY.3
OM007106_PA−CDC−LC0429226_2021−12−03_AY.3
OL996823_PA−CDC−ASC210608511_2021−12−08_AY.3
OM570157_VSP6915_2021−10−03_AY.3
OM224496_PA−CDC−LC0447507_2021−12−23_AY.3
PA−CDC−STM−000719026_2021−08−08_AY.3
OM101174_PA−BOL−M21161078_2021−12−07_AY.3
OM007817_PA−CDC−LC0428383_2021−12−08_AY.3
OL864280_PA−CDC−STM−MMGMV7RGE_2021−12−01_AY.3
OM241310_VSP7737_2021−11−14_AY.3
PA−CDC−LC0154456_2021−07−29_AY.3
PA−CDC−QDX27830609_2021−08−13_AY.3
PA−CDC−LC0154007_2021−07−31_AY.3
OM102133_PA−CDC−STM−ED7RHMSHG_2021−12−14_AY.3
OL950393_PA−CDC−ASC210453078_2021−11−08_AY.3
PA−VSP6839_2021−09−19_AY.3
PA−CDC−ASC210324010_2021−09−30_AY.3
PA−CDC−ASC210085800_2021−09−08_AY.3
PA−VSP6117_2021−08−22_AY.3
PA−0240_2021−07−23_AY.3
PA−VSP5658_2021−08−14_AY.3
PA−Jefferson_COVIDSeq14_42_2021−06−28_AY.3
PA−VSP5511_2021−08−08_AY.3
OL806319_PA−CDC−STM−EPTM4NTH3_2021−11−29_AY.3
PA−VSP5512_2021−08−08_AY.3
MN908947 (Wuhan−Hu−1/2019)
0.2

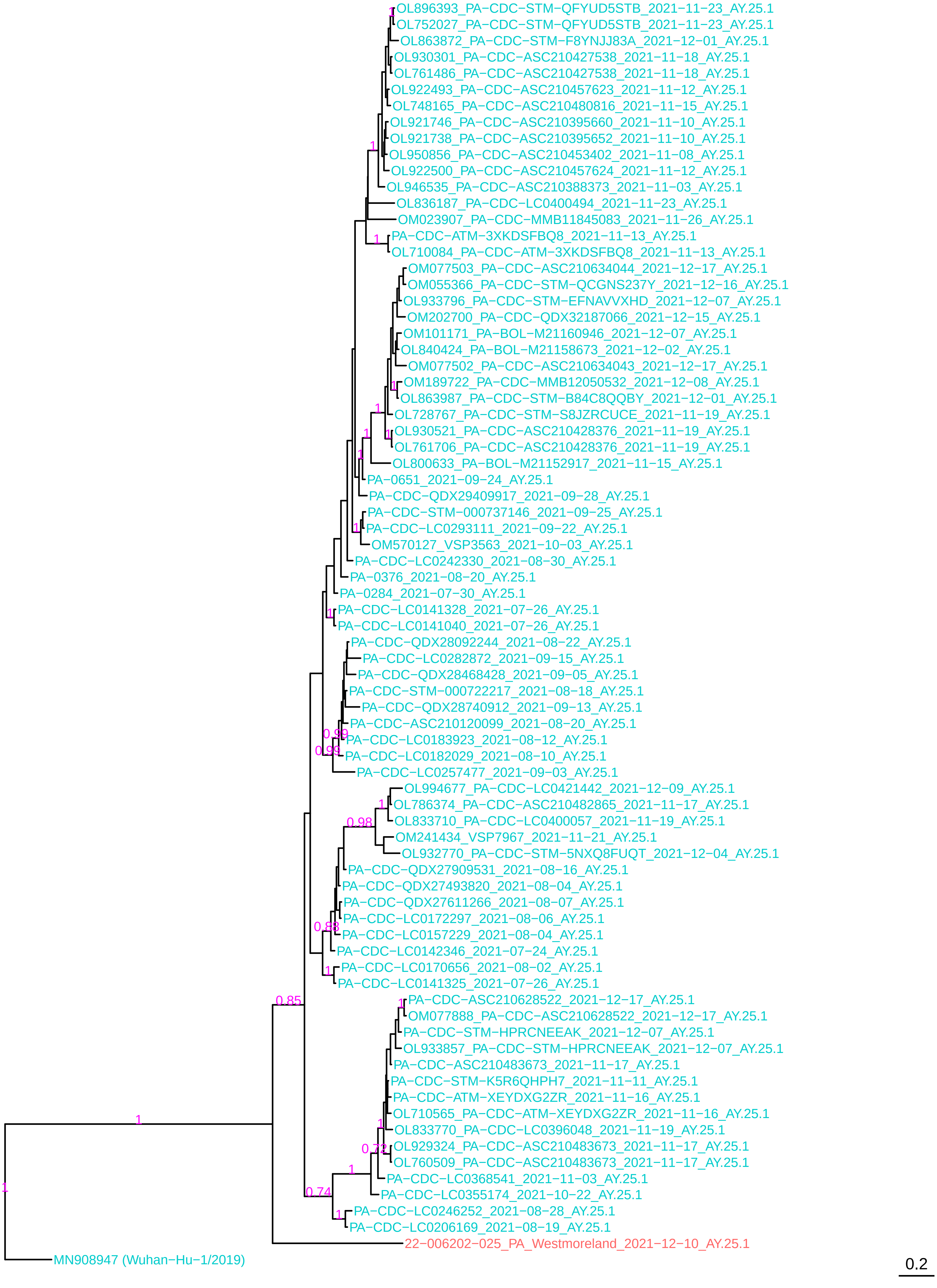

OL896393_PA−CDC−STM−QFYUD5STB_2021−11−23_AY.25.1
OL752027_PA−CDC−STM−QFYUD5STB_2021−11−23_AY.25.1
OL863872_PA−CDC−STM−F8YNJJ83A_2021−12−01_AY.25.1
OL930301_PA−CDC−ASC210427538_2021−11−18_AY.25.1
OL761486_PA−CDC−ASC210427538_2021−11−18_AY.25.1
OL922493_PA−CDC−ASC210457623_2021−11−12_AY.25.1
OL748165_PA−CDC−ASC210480816_2021−11−15_AY.25.1
OL921746_PA−CDC−ASC210395660_2021−11−10_AY.25.1
OL921738_PA−CDC−ASC210395652_2021−11−10_AY.25.1
OL950856_PA−CDC−ASC210453402_2021−11−08_AY.25.1
OL922500_PA−CDC−ASC210457624_2021−11−12_AY.25.1
OL946535_PA−CDC−ASC210388373_2021−11−03_AY.25.1
OL836187_PA−CDC−LC0400494_2021−11−23_AY.25.1
OM023907_PA−CDC−MMB11845083_2021−11−26_AY.25.1
PA−CDC−ATM−3XKDSFBQ8_2021−11−13_AY.25.1
OL710084_PA−CDC−ATM−3XKDSFBQ8_2021−11−13_AY.25.1
OM077503_PA−CDC−ASC210634044_2021−12−17_AY.25.1
OM055366_PA−CDC−STM−QCGNS237Y_2021−12−16_AY.25.1
OL933796_PA−CDC−STM−EFNAVVXHD_2021−12−07_AY.25.1
OM202700_PA−CDC−QDX32187066_2021−12−15_AY.25.1
OM101171_PA−BOL−M21160946_2021−12−07_AY.25.1
OL840424_PA−BOL−M21158673_2021−12−02_AY.25.1
OM077502_PA−CDC−ASC210634043_2021−12−17_AY.25.1
OM189722_PA−CDC−MMB12050532_2021−12−08_AY.25.1
OL863987_PA−CDC−STM−B84C8QQBY_2021−12−01_AY.25.1
OL728767_PA−CDC−STM−S8JZRCUCE_2021−11−19_AY.25.1
OL930521_PA−CDC−ASC210428376_2021−11−19_AY.25.1
OL761706_PA−CDC−ASC210428376_2021−11−19_AY.25.1
OL800633_PA−BOL−M21152917_2021−11−15_AY.25.1
PA−0651_2021−09−24_AY.25.1
PA−CDC−QDX29409917_2021−09−28_AY.25.1
PA−CDC−STM−000737146_2021−09−25_AY.25.1
PA−CDC−LC0293111_2021−09−22_AY.25.1
OM570127_VSP3563_2021−10−03_AY.25.1
PA−CDC−LC0242330_2021−08−30_AY.25.1
PA−0376_2021−08−20_AY.25.1
PA−0284_2021−07−30_AY.25.1
PA−CDC−LC0141328_2021−07−26_AY.25.1
PA−CDC−LC0141040_2021−07−26_AY.25.1
PA−CDC−QDX28092244_2021−08−22_AY.25.1
PA−CDC−LC0282872_2021−09−15_AY.25.1
PA−CDC−QDX28468428_2021−09−05_AY.25.1
PA−CDC−STM−000722217_2021−08−18_AY.25.1
PA−CDC−QDX28740912_2021−09−13_AY.25.1
PA−CDC−ASC210120099_2021−08−20_AY.25.1
PA−CDC−LC0183923_2021−08−12_AY.25.1
PA−CDC−LC0182029_2021−08−10_AY.25.1
PA−CDC−LC0257477_2021−09−03_AY.25.1
OL994677_PA−CDC−LC0421442_2021−12−09_AY.25.1
OL786374_PA−CDC−ASC210482865_2021−11−17_AY.25.1
OL833710_PA−CDC−LC0400057_2021−11−19_AY.25.1
OM241434_VSP7967_2021−11−21_AY.25.1
OL932770_PA−CDC−STM−5NXQ8FUQT_2021−12−04_AY.25.1
PA−CDC−QDX27909531_2021−08−16_AY.25.1
PA−CDC−QDX27493820_2021−08−04_AY.25.1
PA−CDC−QDX27611266_2021−08−07_AY.25.1
PA−CDC−LC0172297_2021−08−06_AY.25.1
PA−CDC−LC0157229_2021−08−04_AY.25.1
PA−CDC−LC0142346_2021−07−24_AY.25.1
PA−CDC−LC0170656_2021−08−02_AY.25.1
PA−CDC−LC0141325_2021−07−26_AY.25.1
PA−CDC−ASC210628522_2021−12−17_AY.25.1
OM077888_PA−CDC−ASC210628522_2021−12−17_AY.25.1
PA−CDC−STM−HPRCNEEAK_2021−12−07_AY.25.1
OL933857_PA−CDC−STM−HPRCNEEAK_2021−12−07_AY.25.1
PA−CDC−ASC210483673_2021−11−17_AY.25.1
PA−CDC−STM−K5R6QHPH7_2021−11−11_AY.25.1
PA−CDC−ATM−XEYDXG2ZR_2021−11−16_AY.25.1
OL710565_PA−CDC−ATM−XEYDXG2ZR_2021−11−16_AY.25.1
OL833770_PA−CDC−LC0396048_2021−11−19_AY.25.1
OL929324_PA−CDC−ASC210483673_2021−11−17_AY.25.1
OL760509_PA−CDC−ASC210483673_2021−11−17_AY.25.1
PA−CDC−LC0368541_2021−11−03_AY.25.1
PA−CDC−LC0355174_2021−10−22_AY.25.1
PA−CDC−LC0246252_2021−08−28_AY.25.1
PA−CDC−LC0206169_2021−08−19_AY.25.1
22−006202−025_PA_Westmoreland_2021−12−10_AY.25.1
MN908947 (Wuhan−Hu−1/2019)
0.2

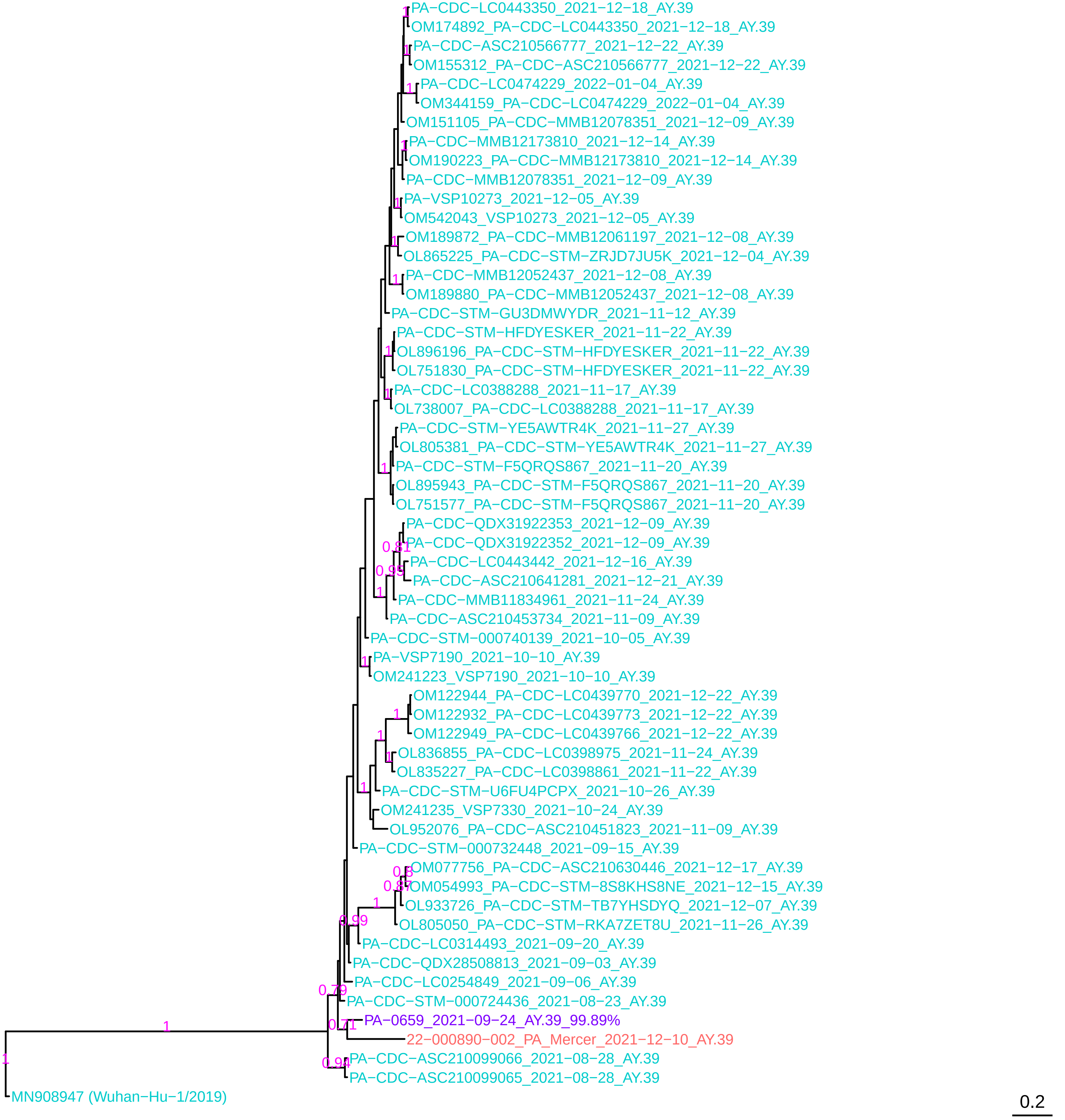

PA−CDC−LC0443350_2021−12−18_AY.39
OM174892_PA−CDC−LC0443350_2021−12−18_AY.39
PA−CDC−ASC210566777_2021−12−22_AY.39
OM155312_PA−CDC−ASC210566777_2021−12−22_AY.39
PA−CDC−LC0474229_2022−01−04_AY.39
OM344159_PA−CDC−LC0474229_2022−01−04_AY.39
OM151105_PA−CDC−MMB12078351_2021−12−09_AY.39
PA−CDC−MMB12173810_2021−12−14_AY.39
OM190223_PA−CDC−MMB12173810_2021−12−14_AY.39
PA−CDC−MMB12078351_2021−12−09_AY.39
PA−VSP10273_2021−12−05_AY.39
OM542043_VSP10273_2021−12−05_AY.39
OM189872_PA−CDC−MMB12061197_2021−12−08_AY.39
OL865225_PA−CDC−STM−ZRJD7JU5K_2021−12−04_AY.39
PA−CDC−MMB12052437_2021−12−08_AY.39
OM189880_PA−CDC−MMB12052437_2021−12−08_AY.39
PA−CDC−STM−GU3DMWYDR_2021−11−12_AY.39
PA−CDC−STM−HFDYESKER_2021−11−22_AY.39
OL896196_PA−CDC−STM−HFDYESKER_2021−11−22_AY.39
OL751830_PA−CDC−STM−HFDYESKER_2021−11−22_AY.39
PA−CDC−LC0388288_2021−11−17_AY.39
OL738007_PA−CDC−LC0388288_2021−11−17_AY.39
PA−CDC−STM−YE5AWTR4K_2021−11−27_AY.39
OL805381_PA−CDC−STM−YE5AWTR4K_2021−11−27_AY.39
PA−CDC−STM−F5QRQS867_2021−11−20_AY.39
OL895943_PA−CDC−STM−F5QRQS867_2021−11−20_AY.39
OL751577_PA−CDC−STM−F5QRQS867_2021−11−20_AY.39
PA−CDC−QDX31922353_2021−12−09_AY.39
0.81
PA−CDC−QDX31922352_2021−12−09_AY.39
PA−CDC−LC0443442_2021−12−16_AY.39
0.95
PA−CDC−ASC210641281_2021−12−21_AY.39
PA−CDC−MMB11834961_2021−11−24_AY.39
PA−CDC−ASC210453734_2021−11−09_AY.39
PA−CDC−STM−000740139_2021−10−05_AY.39
PA−VSP7190_2021−10−10_AY.39
OM241223_VSP7190_2021−10−10_AY.39
OM122944_PA−CDC−LC0439770_2021−12−22_AY.39
OM122932_PA−CDC−LC0439773_2021−12−22_AY.39
OM122949_PA−CDC−LC0439766_2021−12−22_AY.39
OL836855_PA−CDC−LC0398975_2021−11−24_AY.39
OL835227_PA−CDC−LC0398861_2021−11−22_AY.39
PA−CDC−STM−U6FU4PCPX_2021−10−26_AY.39
OM241235_VSP7330_2021−10−24_AY.39
OL952076_PA−CDC−ASC210451823_2021−11−09_AY.39
PA−CDC−STM−000732448_2021−09−15_AY.39
0.8
OM077756_PA−CDC−ASC210630446_2021−12−17_AY.39
0.87
OM054993_PA−CDC−STM−8S8KHS8NE_2021−12−15_AY.39
OL933726_PA−CDC−STM−TB7YHSDYQ_2021−12−07_AY.39
OL805050_PA−CDC−STM−RKA7ZET8U_2021−11−26_AY.39
0.99
PA−CDC−LC0314493_2021−09−20_AY.39
PA−CDC−QDX28508813_2021−09−03_AY.39
PA−CDC−LC0254849_2021−09−06_AY.39
0.79
PA−CDC−STM−000724436_2021−08−23_AY.39
PA−0659_2021−09−24_AY.39_99.89%
0.71
22−000890−002_PA_Mercer_2021−12−10_AY.39
0.94
PA−CDC−ASC210099066_2021−08−28_AY.39
PA−CDC−ASC210099065_2021−08−28_AY.39
MN908947 (Wuhan−Hu−1/2019)
0.2

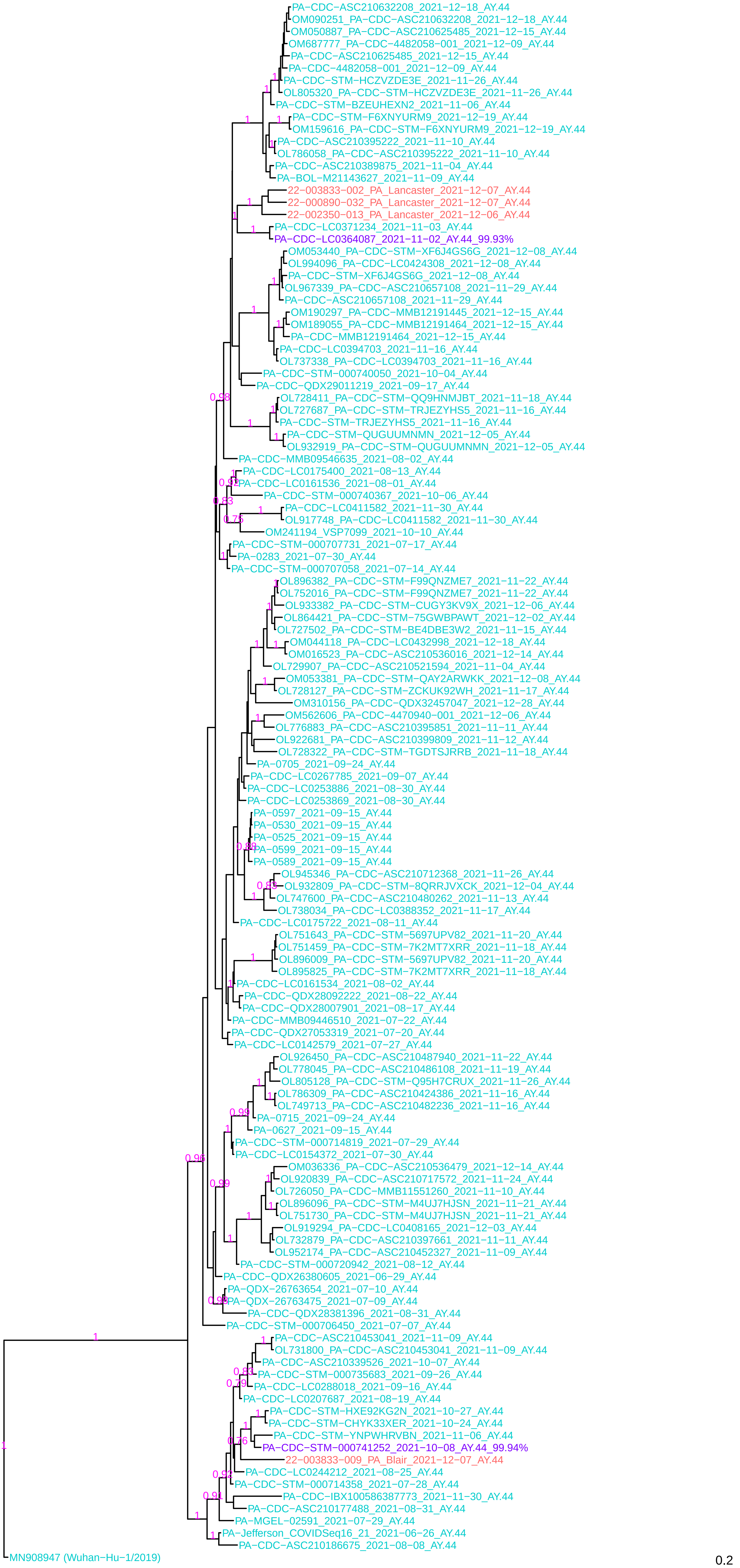
PA-CDC-ASC210632208_2021-12-18_AY.44
OM090251_PA-CDC-ASC210632208_2021-12-18_AY.44
OM050887_PA-CDC-ASC210625485_2021-12-15_AY.44
OM687777_PA-CDC-4482058-001_2021-12-09_AY.44
PA-CDC-ASC210625485_2021-12-15_AY.44
PA-CDC-4482058-001_2021-12-09_AY.44
PA-CDC-STM-HCZVZDE3E_2021-11-26_AY.44
OL805320_PA-CDC-STM-HCZVZDE3E_2021-11-26_AY.44
PA-CDC-STM-BZEUHEXN2_2021-11-06_AY.44
PA-CDC-STM-F6XNYURM9_2021-12-19_AY.44
OM159616_PA-CDC-STM-F6XNYURM9_2021-12-19_AY.44
PA-CDC-ASC210395222_2021-11-10_AY.44
OL786058_PA-CDC-ASC210395222_2021-11-10_AY.44
PA-CDC-ASC210389875_2021-11-04_AY.44
PA-BOL-M21143627_2021-11-09_AY.44
22-003833-002_PA_Lancaster_2021-12-07_AY.44
22-000890-032_PA_Lancaster_2021-12-07_AY.44
22-002350-013_PA_Lancaster_2021-12-06_AY.44
PA-CDC-LC0371234_2021-11-03_AY.44
PA-CDC-LC0364087_2021-11-02_AY.44_99.93%
OM053440_PA-CDC-STM-XF6J4GS6G_2021-12-08_AY.44
OL994096_PA-CDC-LC0424308_2021-12-08_AY.44
PA-CDC-STM-XF6J4GS6G_2021-12-08_AY.44
OL967339_PA-CDC-ASC210657108_2021-11-29_AY.44
PA-CDC-ASC210657108_2021-11-29_AY.44
OM190297_PA-CDC-MMB12191445_2021-12-15_AY.44
OM189055_PA-CDC-MMB12191464_2021-12-15_AY.44
PA-CDC-MMB12191464_2021-12-15_AY.44
PA-CDC-LC0394703_2021-11-16_AY.44
OL737338_PA-CDC-LC0394703_2021-11-16_AY.44
PA-CDC-STM-000740050_2021-10-04_AY.44
PA-CDC-QDX29011219_2021-09-17_AY.44
OL728411_PA-CDC-STM-QQ9HNMJBT_2021-11-18_AY.44
OL727687_PA-CDC-STM-TRJEZYHS5_2021-11-16_AY.44
PA-CDC-STM-TRJEZYHS5_2021-11-16_AY.44
PA-CDC-STM-QUGUUMNMN_2021-12-05_AY.44
OL932919_PA-CDC-STM-QUGUUMNMN_2021-12-05_AY.44
PA-CDC-MMB09546635_2021-08-02_AY.44
PA-CDC-LC0175400_2021-08-13_AY.44
PA-CDC-LC0161536_2021-08-01_AY.44
PA-CDC-STM-000740367_2021-10-06_AY.44
PA-CDC-LC0411582_2021-11-30_AY.44
OL917748_PA-CDC-LC0411582_2021-11-30_AY.44
OM241194_VSP7099_2021-10-10_AY.44
PA-CDC-STM-000707731_2021-07-17_AY.44
PA-0283_2021-07-30_AY.44
PA-CDC-STM-000707058_2021-07-14_AY.44
OL896382_PA-CDC-STM-F99QNZME7_2021-11-22_AY.44
OL752016_PA-CDC-STM-F99QNZME7_2021-11-22_AY.44
OL933382_PA-CDC-STM-CUGY3KV9X_2021-12-06_AY.44
OL864421_PA-CDC-STM-75GWBPAWT_2021-12-02_AY.44
OL727502_PA-CDC-STM-BE4DBE3W2_2021-11-15_AY.44
OM044118_PA-CDC-LC0432998_2021-12-18_AY.44
OM016523_PA-CDC-ASC210536016_2021-12-14_AY.44
OL729907_PA-CDC-ASC210521594_2021-11-04_AY.44
OM053381_PA-CDC-STM-QAY2ARWKK_2021-12-08_AY.44
OL728127_PA-CDC-STM-ZCKUK92WH_2021-11-17_AY.44
OM310156_PA-CDC-QDX32457047_2021-12-28_AY.44
OM562606_PA-CDC-4470940-001_2021-12-06_AY.44
OL776883_PA-CDC-ASC210395851_2021-11-11_AY.44
OL922681_PA-CDC-ASC210399809_2021-11-12_AY.44
OL728322_PA-CDC-STM-TGDTSJRRB_2021-11-18_AY.44
PA-0705_2021-09-24_AY.44
PA-CDC-LC0267785_2021-09-07_AY.44
PA-CDC-LC0253886_2021-08-30_AY.44
PA-CDC-LC0253869_2021-08-30_AY.44
PA-0597_2021-09-15_AY.44
PA-0530_2021-09-15_AY.44
PA-0525_2021-09-15_AY.44
PA-0599_2021-09-15_AY.44
PA-0589_2021-09-15_AY.44
OL945346_PA-CDC-ASC210712368_2021-11-26_AY.44
OL932809_PA-CDC-STM-8QRRJVXCK_2021-12-04_AY.44
OL747600_PA-CDC-ASC210480262_2021-11-13_AY.44
OL738034_PA-CDC-LC0388352_2021-11-17_AY.44
PA-CDC-LC0175722_2021-08-11_AY.44
OL751643_PA-CDC-STM-5697UPV82_2021-11-20_AY.44
OL751459_PA-CDC-STM-7K2MT7XRR_2021-11-18_AY.44
OL896009_PA-CDC-STM-5697UPV82_2021-11-20_AY.44
OL895825_PA-CDC-STM-7K2MT7XRR_2021-11-18_AY.44
PA-CDC-LC0161534_2021-08-02_AY.44
PA-CDC-QDX28092222_2021-08-22_AY.44
PA-CDC-QDX28007901_2021-08-17_AY.44
PA-CDC-MMB09446510_2021-07-22_AY.44
PA-CDC-QDX27053319_2021-07-20_AY.44
PA-CDC-LC0142579_2021-07-27_AY.44
OL926450_PA-CDC-ASC210487940_2021-11-22_AY.44
OL778045_PA-CDC-ASC210486108_2021-11-19_AY.44
OL805128_PA-CDC-STM-Q95H7CRUX_2021-11-26_AY.44
OL786309_PA-CDC-ASC210424386_2021-11-16_AY.44
OL749713_PA-CDC-ASC210482236_2021-11-16_AY.44
PA-0715_2021-09-24_AY.44
PA-0627_2021-09-15_AY.44
PA-CDC-STM-000714819_2021-07-29_AY.44
PA-CDC-LC0154372_2021-07-30_AY.44
OM036336_PA-CDC-ASC210536479_2021-12-14_AY.44
OL920839_PA-CDC-ASC210717572_2021-11-24_AY.44
OL726050_PA-CDC-MMB11551260_2021-11-10_AY.44
OL896096_PA-CDC-STM-M4UJ7HJSN_2021-11-21_AY.44
OL751730_PA-CDC-STM-M4UJ7HJSN_2021-11-21_AY.44
OL919294_PA-CDC-LC0408165_2021-12-03_AY.44
OL732879_PA-CDC-ASC210397661_2021-11-11_AY.44
OL952174_PA-CDC-ASC210452327_2021-11-09_AY.44
PA-CDC-STM-000720942_2021-08-12_AY.44
PA-CDC-QDX26380605_2021-06-29_AY.44
PA-QDX-26763654_2021-07-10_AY.44
PA-QDX-26763475_2021-07-09_AY.44
PA-CDC-QDX28381396_2021-08-31_AY.44
PA-CDC-STM-000706450_2021-07-07_AY.44
PA-CDC-ASC210453041_2021-11-09_AY.44
OL731800_PA-CDC-ASC210453041_2021-11-09_AY.44
PA-CDC-ASC210339526_2021-10-07_AY.44
PA-CDC-STM-000735683_2021-09-26_AY.44
PA-CDC-LC0288018_2021-09-16_AY.44
PA-CDC-LC0207687_2021-08-19_AY.44
PA-CDC-STM-HXE92KG2N_2021-10-27_AY.44
PA-CDC-STM-CHYK33XER_2021-10-24_AY.44
PA-CDC-STM-YNPWHRVBN_2021-11-06_AY.44
PA-CDC-STM-000741252_2021-10-08_AY.44_99.94%
22-003833-009_PA_Blair_2021-12-07_AY.44
PA-CDC-LC0244212_2021-08-25_AY.44
PA-CDC-STM-000714358_2021-07-28_AY.44
PA-CDC-IBX100586387773_2021-11-30_AY.44
PA-CDC-ASC210177488_2021-08-31_AY.44
PA-MGEL-02591_2021-07-29_AY.44
PA-Jefferson_COVIDSeq16_21_2021-06-26_AY.44
PA-CDC-ASC210186675_2021-08-08_AY.44
MN908947 (Wuhan-Hu-1/2019)
0.2

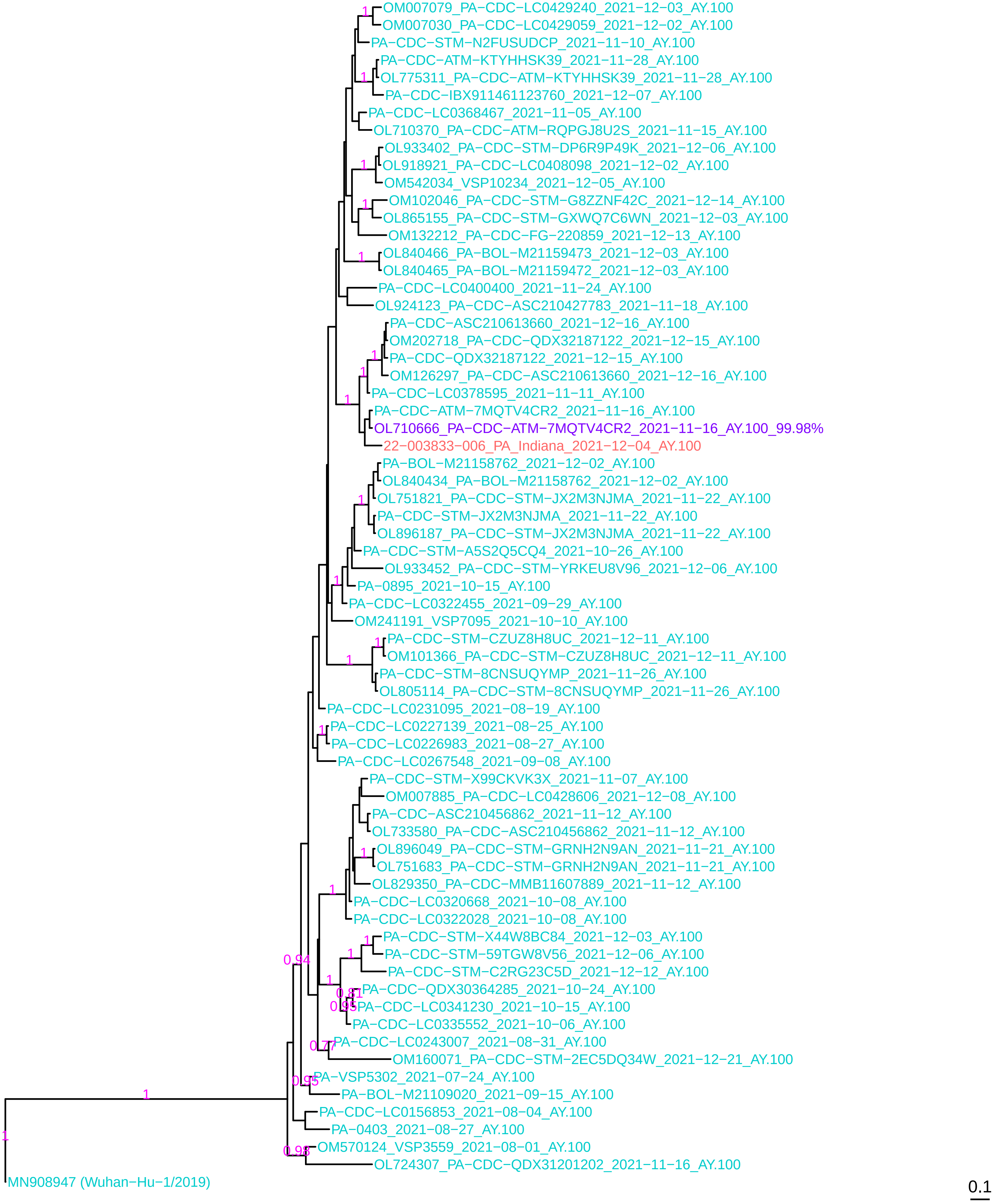
OM007079_PA−CDC−LC0429240_2021−12−03_AY.100
OM007030_PA−CDC−LC0429059_2021−12−02_AY.100
PA−CDC−STM−N2FUSUDCP_2021−11−10_AY.100
PA−CDC−ATM−KTYHHSK39_2021−11−28_AY.100
OL775311_PA−CDC−ATM−KTYHHSK39_2021−11−28_AY.100
PA−CDC−IBX911461123760_2021−12−07_AY.100
PA−CDC−LC0368467_2021−11−05_AY.100
OL710370_PA−CDC−ATM−RQPGJ8U2S_2021−11−15_AY.100
OL933402_PA−CDC−STM−DP6R9P49K_2021−12−06_AY.100
OL918921_PA−CDC−LC0408098_2021−12−02_AY.100
OM542034_VSP10234_2021−12−05_AY.100
OM102046_PA−CDC−STM−G8ZZNF42C_2021−12−14_AY.100
OL865155_PA−CDC−STM−GXWQ7C6WN_2021−12−03_AY.100
OM132212_PA−CDC−FG−220859_2021−12−13_AY.100
OL840466_PA−BOL−M21159473_2021−12−03_AY.100
OL840465_PA−BOL−M21159472_2021−12−03_AY.100
PA−CDC−LC0400400_2021−11−24_AY.100
OL924123_PA−CDC−ASC210427783_2021−11−18_AY.100
PA−CDC−ASC210613660_2021−12−16_AY.100
OM202718_PA−CDC−QDX32187122_2021−12−15_AY.100
PA−CDC−QDX32187122_2021−12−15_AY.100
OM126297_PA−CDC−ASC210613660_2021−12−16_AY.100
PA−CDC−LC0378595_2021−11−11_AY.100
PA−CDC−ATM−7MQTV4CR2_2021−11−16_AY.100
OL710666_PA−CDC−ATM−7MQTV4CR2_2021−11−16_AY.100_99.98%
22−003833−006_PA_Indiana_2021−12−04_AY.100
PA−BOL−M21158762_2021−12−02_AY.100
OL840434_PA−BOL−M21158762_2021−12−02_AY.100
OL751821_PA−CDC−STM−JX2M3NJMA_2021−11−22_AY.100
PA−CDC−STM−JX2M3NJMA_2021−11−22_AY.100
OL896187_PA−CDC−STM−JX2M3NJMA_2021−11−22_AY.100
PA−CDC−STM−A5S2Q5CQ4_2021−10−26_AY.100
OL933452_PA−CDC−STM−YRKEU8V96_2021−12−06_AY.100
PA−0895_2021−10−15_AY.100
PA−CDC−LC0322455_2021−09−29_AY.100
OM241191_VSP7095_2021−10−10_AY.100
PA−CDC−STM−CZUZ8H8UC_2021−12−11_AY.100
OM101366_PA−CDC−STM−CZUZ8H8UC_2021−12−11_AY.100
PA−CDC−STM−8CNSUQYMP_2021−11−26_AY.100
OL805114_PA−CDC−STM−8CNSUQYMP_2021−11−26_AY.100
PA−CDC−LC0231095_2021−08−19_AY.100
PA−CDC−LC0227139_2021−08−25_AY.100
PA−CDC−LC0226983_2021−08−27_AY.100
PA−CDC−LC0267548_2021−09−08_AY.100
PA−CDC−STM−X99CKVK3X_2021−11−07_AY.100
OM007885_PA−CDC−LC0428606_2021−12−08_AY.100
PA−CDC−ASC210456862_2021−11−12_AY.100
OL733580_PA−CDC−ASC210456862_2021−11−12_AY.100
OL896049_PA−CDC−STM−GRNH2N9AN_2021−11−21_AY.100
OL751683_PA−CDC−STM−GRNH2N9AN_2021−11−21_AY.100
OL829350_PA−CDC−MMB11607889_2021−11−12_AY.100
PA−CDC−LC0320668_2021−10−08_AY.100
PA−CDC−LC0322028_2021−10−08_AY.100
PA−CDC−STM−X44W8BC84_2021−12−03_AY.100
PA−CDC−STM−59TGW8V56_2021−12−06_AY.100
PA−CDC−STM−C2RG23C5D_2021−12−12_AY.100
PA−CDC−QDX30364285_2021−10−24_AY.100
PA−CDC−LC0341230_2021−10−15_AY.100
PA−CDC−LC0335552_2021−10−06_AY.100
PA−CDC−LC0243007_2021−08−31_AY.100
OM160071_PA−CDC−STM−2EC5DQ34W_2021−12−21_AY.100
PA−VSP5302_2021−07−24_AY.100
PA−BOL−M21109020_2021−09−15_AY.100
PA−CDC−LC0156853_2021−08−04_AY.100
PA−0403_2021−08−27_AY.100
OM570124_VSP3559_2021−08−01_AY.100
OL724307_PA−CDC−QDX31201202_2021−11−16_AY.100
MN908947 (Wuhan−Hu−1/2019)
0.1

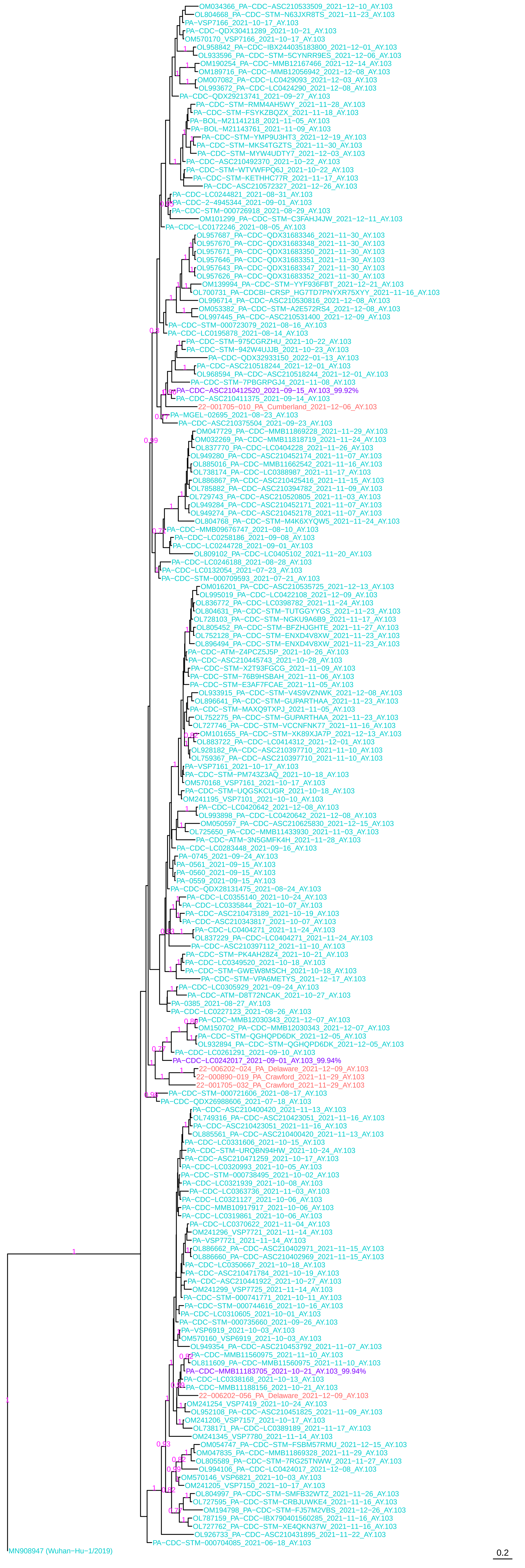
OM034366_PA-CDC-ASC210533509_2021-12-10_AY.103
OL804668_PA-CDC-STM-N63JXR8TS_2021-11-23_AY.103
PA-VSP7166_2021-10-17_AY.103
PA-CDC-QDX30411289_2021-10-21_AY.103
OM570170_VSP7166_2021-10-17_AY.103
OL958842_PA-CDC-IBX244035183800_2021-12-01_AY.103
OL933596_PA-CDC-STM-5CYNRR9ES_2021-12-06_AY.103
OM190254_PA-CDC-MMB12167466_2021-12-14_AY.103
OM189716_PA-CDC-MMB12056942_2021-12-08_AY.103
OM007082_PA-CDC-LC0429093_2021-12-03_AY.103
OL993672_PA-CDC-LC0424290_2021-12-08_AY.103
PA-CDC-QDX29213741_2021-09-27_AY.103
PA-CDC-STM-RMM4AH5WY_2021-11-28_AY.103
PA-CDC-STM-FSYKZBQZX_2021-11-18_AY.103
PA-BOL-M21141218_2021-11-05_AY.103
PA-BOL-M21143761_2021-11-09_AY.103
PA-CDC-STM-YMP9U3HT3_2021-12-19_AY.103
PA-CDC-STM-MKS4TGZTS_2021-11-30_AY.103
PA-CDC-STM-MYW4UDTY7_2021-12-03_AY.103
PA-CDC-ASC210492370_2021-10-22_AY.103
PA-CDC-STM-WTVWFPQ6J_2021-10-22_AY.103
PA-CDC-STM-KETHHC77R_2021-11-17_AY.103
PA-CDC-ASC210572327_2021-12-26_AY.103
PA-CDC-LC0244821_2021-08-31_AY.103
PA-CDC-2-4945344_2021-09-01_AY.103
PA-CDC-STM-000726918_2021-08-29_AY.103
OM101299_PA-CDC-STM-C3FAHJ4JW_2021-12-11_AY.103
PA-CDC-LC0172246_2021-08-05_AY.103
OL957687_PA-CDC-QDX31683346_2021-11-30_AY.103
OL957670_PA-CDC-QDX31683348_2021-11-30_AY.103
OL957671_PA-CDC-QDX31683350_2021-11-30_AY.103
OL957646_PA-CDC-QDX31683351_2021-11-30_AY.103
OL957643_PA-CDC-QDX31683347_2021-11-30_AY.103
OL957626_PA-CDC-QDX31683352_2021-11-30_AY.103
OM139994_PA-CDC-STM-YYF936FBT_2021-12-21_AY.103
OL700731_PA-CDCBI-CRSP_HG7TD7PNYXR75XYY_2021-11-16_AY.103
OL996714_PA-CDC-ASC210530816_2021-12-08_AY.103
OM053382_PA-CDC-STM-A2E572RS4_2021-12-08_AY.103
OL997445_PA-CDC-ASC210531400_2021-12-09_AY.103
PA-CDC-STM-000723079_2021-08-16_AY.103
PA-CDC-LC0195878_2021-08-14_AY.103
PA-CDC-STM-975CGRZHU_2021-10-22_AY.103
PA-CDC-STM-942W4UJJB_2021-10-23_AY.103
PA-CDC-QDX32933150_2022-01-13_AY.103
PA-CDC-ASC210518244_2021-12-01_AY.103
OL968594_PA-CDC-ASC210518244_2021-12-01_AY.103
PA-CDC-STM-7PBGRPGJ4_2021-11-08_AY.103
PA-CDC-ASC210412520_2021-09-15_AY.103_99.92%
PA-CDC-ASC210411375_2021-09-14_AY.103
22-001705-010_PA_Cumberland_2021-12-06_AY.103
PA-MGEL-02695_2021-08-23_AY.103
PA-CDC-ASC210375504_2021-09-23_AY.103
OM047729_PA-CDC-MMB11869228_2021-11-29_AY.103
OM032269_PA-CDC-MMB11818719_2021-11-24_AY.103
OL837770_PA-CDC-LC0404228_2021-11-26_AY.103
OL949280_PA-CDC-ASC210452174_2021-11-07_AY.103
OL885016_PA-CDC-MMB11662542_2021-11-16_AY.103
OL738174_PA-CDC-LC0388987_2021-11-17_AY.103
OL886867_PA-CDC-ASC210425416_2021-11-15_AY.103
OL785882_PA-CDC-ASC210394782_2021-11-09_AY.103
OL729743_PA-CDC-ASC210520805_2021-11-03_AY.103
OL949284_PA-CDC-ASC210452171_2021-11-07_AY.103
OL949274_PA-CDC-ASC210452178_2021-11-07_AY.103
OL804768_PA-CDC-STM-M4K6XYQW5_2021-11-24_AY.103
PA-CDC-MMB09676747_2021-08-10_AY.103
PA-CDC-LC0258186_2021-09-08_AY.103
PA-CDC-LC0244728_2021-09-01_AY.103
OL809102_PA-CDC-LC0405102_2021-11-20_AY.103
PA-CDC-LC0246188_2021-08-28_AY.103
PA-CDC-LC0132054_2021-07-23_AY.103
PA-CDC-STM-000709593_2021-07-21_AY.103
OM016201_PA-CDC-ASC210535725_2021-12-13_AY.103
OL995019_PA-CDC-LC0422108_2021-12-09_AY.103
OL836772_PA-CDC-LC0398782_2021-11-24_AY.103
OL804631_PA-CDC-STM-TUTGGYYGS_2021-11-23_AY.103
OL728103_PA-CDC-STM-NGKU9A6B9_2021-11-17_AY.103
OL805452_PA-CDC-STM-BFZHJGHTE_2021-11-27_AY.103
OL752128_PA-CDC-STM-ENXD4V8XW_2021-11-23_AY.103
OL896494_PA-CDC-STM-ENXD4V8XW_2021-11-23_AY.103
PA-CDC-ATM-Z4PCZ5J5P_2021-10-26_AY.103
PA-CDC-ASC210445743_2021-10-28_AY.103
PA-CDC-STM-X2T93FGCG_2021-11-09_AY.103
PA-CDC-STM-76B9HSBAH_2021-11-06_AY.103
PA-CDC-STM-E3AF7FCAE_2021-11-05_AY.103
OL933915_PA-CDC-STM-V4S9VZNWK_2021-12-08_AY.103
OL896641_PA-CDC-STM-GUPARTHAA_2021-11-23_AY.103
PA-CDC-STM-MAXQ9TXPJ_2021-11-05_AY.103
OL752275_PA-CDC-STM-GUPARTHAA_2021-11-23_AY.103
OL727746_PA-CDC-STM-VCCNFNK77_2021-11-16_AY.103
OM101655_PA-CDC-STM-XK89XJA7P_2021-12-13_AY.103
OL883722_PA-CDC-LC0414312_2021-12-01_AY.103
OL928182_PA-CDC-ASC210397710_2021-11-10_AY.103
OL759367_PA-CDC-ASC210397710_2021-11-10_AY.103
PA-VSP7161_2021-10-17_AY.103
PA-CDC-STM-PM743Z3AQ_2021-10-18_AY.103
OM570168_VSP7161_2021-10-17_AY.103
PA-CDC-STM-UQGSKCUGR_2021-10-18_AY.103
OM241195_VSP7101_2021-10-10_AY.103
PA-CDC-LC0420642_2021-12-08_AY.103
OL993898_PA-CDC-LC0420642_2021-12-08_AY.103
OM050597_PA-CDC-ASC210625830_2021-12-15_AY.103
OL725650_PA-CDC-MMB11433930_2021-11-03_AY.103
PA-CDC-ATM-3N5GMFK4H_2021-11-28_AY.103
PA-CDC-LC0283448_2021-09-16_AY.103
PA-0745_2021-09-24_AY.103
PA-0561_2021-09-15_AY.103
PA-0560_2021-09-15_AY.103
PA-0559_2021-09-15_AY.103
PA-CDC-QDX28131475_2021-08-24_AY.103
PA-CDC-LC0355140_2021-10-24_AY.103
PA-CDC-LC0335844_2021-10-07_AY.103
PA-CDC-ASC210473189_2021-10-19_AY.103
PA-CDC-ASC210343817_2021-10-07_AY.103
PA-CDC-LC0404271_2021-11-24_AY.103
OL837229_PA-CDC-LC0404271_2021-11-24_AY.103
PA-CDC-ASC210397112_2021-11-10_AY.103
PA-CDC-STM-PK4AH28Z4_2021-10-21_AY.103
PA-CDC-LC0349520_2021-10-18_AY.103
PA-CDC-STM-GWEW8MSCH_2021-10-18_AY.103
PA-CDC-STM-VPA6METYS_2021-12-17_AY.103
PA-CDC-LC0305929_2021-09-24_AY.103
PA-CDC-ATM-D8T72NCAK_2021-10-27_AY.103
PA-0385_2021-08-27_AY.103
PA-CDC-LC0227123_2021-08-26_AY.103
PA-CDC-MMB12030343_2021-12-07_AY.103
OM150702_PA-CDC-MMB12030343_2021-12-07_AY.103
PA-CDC-STM-QGHQPD6DK_2021-12-05_AY.103
OL932894_PA-CDC-STM-QGHQPD6DK_2021-12-05_AY.103
PA-CDC-LC0261291_2021-09-10_AY.103
PA-CDC-LC0242017_2021-09-01_AY.103_99.94%
22-006202-024_PA_Delaware_2021-12-09_AY.103
22-000890-019_PA_Crawford_2021-11-29_AY.103
22-001705-032_PA_Crawford_2021-11-29_AY.103
PA-CDC-STM-000721606_2021-08-17_AY.103
PA-CDC-QDX26988606_2021-07-18_AY.103
PA-CDC-ASC210400420_2021-11-13_AY.103
OL749316_PA-CDC-ASC210423051_2021-11-16_AY.103
PA-CDC-ASC210423051_2021-11-16_AY.103
OL885561_PA-CDC-ASC210400420_2021-11-13_AY.103
PA-CDC-LC0331606_2021-10-15_AY.103
PA-CDC-STM-URQBN94HW_2021-10-24_AY.103
PA-CDC-ASC210471259_2021-10-17_AY.103
PA-CDC-LC0320993_2021-10-05_AY.103
PA-CDC-STM-000738495_2021-10-02_AY.103
PA-CDC-LC0321939_2021-10-08_AY.103
PA-CDC-LC0363736_2021-11-03_AY.103
PA-CDC-LC0321127_2021-10-06_AY.103
PA-CDC-MMB10917917_2021-10-06_AY.103
PA-CDC-LC0319861_2021-10-06_AY.103
PA-CDC-LC0370622_2021-11-04_AY.103
OM241296_VSP7721_2021-11-14_AY.103
PA-VSP7721_2021-11-14_AY.103
OL886662_PA-CDC-ASC210402971_2021-11-15_AY.103
OL886660_PA-CDC-ASC210402969_2021-11-15_AY.103
PA-CDC-LC0350667_2021-10-18_AY.103
PA-CDC-ASC210471784_2021-10-19_AY.103
PA-CDC-ASC210441922_2021-10-27_AY.103
OM241299_VSP7725_2021-11-14_AY.103
PA-CDC-STM-000741771_2021-10-11_AY.103
PA-CDC-STM-000744616_2021-10-16_AY.103
PA-CDC-LC0310605_2021-10-01_AY.103
PA-CDC-STM-000735660_2021-09-26_AY.103
PA-VSP6919_2021-10-03_AY.103
OM570160_VSP6919_2021-10-03_AY.103
OL949354_PA-CDC-ASC210453792_2021-11-07_AY.103
PA-CDC-MMB11560975_2021-11-10_AY.103
OL811609_PA-CDC-MMB11560975_2021-11-10_AY.103
PA-CDC-MMB11183705_2021-10-21_AY.103_99.94%
PA-CDC-LC0338168_2021-10-13_AY.103
PA-CDC-MMB11188156_2021-10-21_AY.103
22-006202-056_PA_Delaware_2021-12-09_AY.103
OM241254_VSP7419_2021-10-24_AY.103
OL952108_PA-CDC-ASC210451825_2021-11-09_AY.103
OM241206_VSP7157_2021-10-17_AY.103
OL738171_PA-CDC-LC0389189_2021-11-17_AY.103
OM241345_VSP7780_2021-11-14_AY.103
OM054747_PA-CDC-STM-FSBM57RMU_2021-12-15_AY.103
OM047835_PA-CDC-MMB11869328_2021-11-29_AY.103
OL805589_PA-CDC-STM-7RG25TNWW_2021-11-27_AY.103
OL994106_PA-CDC-LC0424017_2021-12-08_AY.103
OM570146_VSP6821_2021-10-03_AY.103
OM241205_VSP7150_2021-10-17_AY.103
OL804997_PA-CDC-STM-SMFB32WTZ_2021-11-26_AY.103
OL727595_PA-CDC-STM-CRBJUWKE4_2021-11-16_AY.103
OM194798_PA-CDC-STM-FJ57M2VBS_2021-12-26_AY.103
OL787159_PA-CDC-IBX790401560285_2021-11-16_AY.103
OL727762_PA-CDC-STM-XE4QKN37W_2021-11-16_AY.103
OL926733_PA-CDC-ASC210431895_2021-11-22_AY.103
PA-CDC-STM-000704085_2021-06-18_AY.103
MN908947 (Wuhan-Hu-1/2019)
0.2

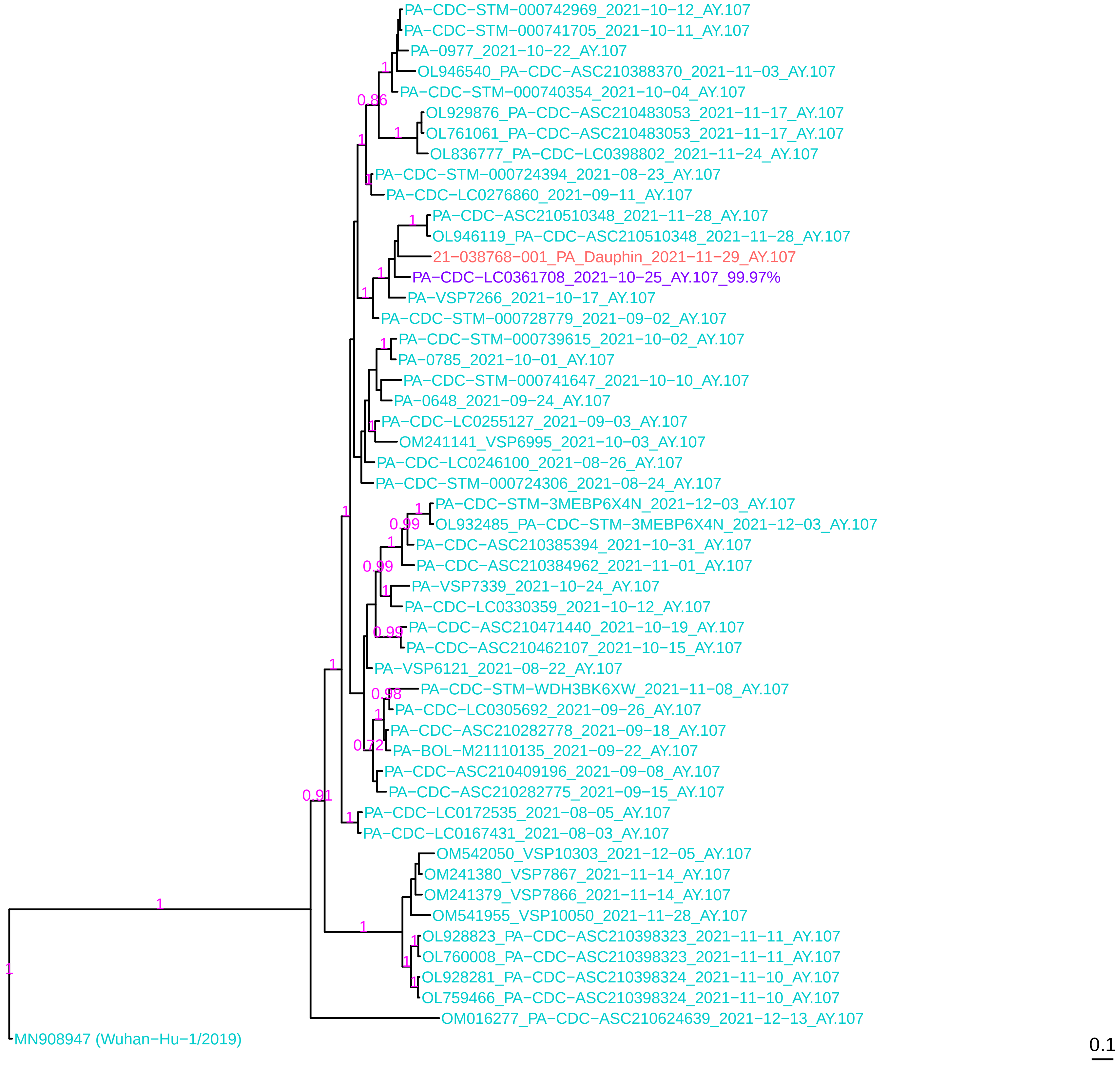
PA−CDC−STM−000742969_2021−10−12_AY.107
PA−CDC−STM−000741705_2021−10−11_AY.107
PA−0977_2021−10−22_AY.107
OL946540_PA−CDC−ASC210388370_2021−11−03_AY.107
PA−CDC−STM−000740354_2021−10−04_AY.107
OL929876_PA−CDC−ASC210483053_2021−11−17_AY.107
OL761061_PA−CDC−ASC210483053_2021−11−17_AY.107
OL836777_PA−CDC−LC0398802_2021−11−24_AY.107
PA−CDC−STM−000724394_2021−08−23_AY.107
PA−CDC−LC0276860_2021−09−11_AY.107
PA−CDC−ASC210510348_2021−11−28_AY.107
OL946119_PA−CDC−ASC210510348_2021−11−28_AY.107
21−038768−001_PA_Dauphin_2021−11−29_AY.107
PA−CDC−LC0361708_2021−10−25_AY.107_99.97%
PA−VSP7266_2021−10−17_AY.107
PA−CDC−STM−000728779_2021−09−02_AY.107
PA−CDC−STM−000739615_2021−10−02_AY.107
PA−0785_2021−10−01_AY.107
PA−CDC−STM−000741647_2021−10−10_AY.107
PA−0648_2021−09−24_AY.107
PA−CDC−LC0255127_2021−09−03_AY.107
OM241141_VSP6995_2021−10−03_AY.107
PA−CDC−LC0246100_2021−08−26_AY.107
PA−CDC−STM−000724306_2021−08−24_AY.107
PA−CDC−STM−3MEBP6X4N_2021−12−03_AY.107
OL932485_PA−CDC−STM−3MEBP6X4N_2021−12−03_AY.107
PA−CDC−ASC210385394_2021−10−31_AY.107
PA−CDC−ASC210384962_2021−11−01_AY.107
PA−VSP7339_2021−10−24_AY.107
PA−CDC−LC0330359_2021−10−12_AY.107
PA−CDC−ASC210471440_2021−10−19_AY.107
PA−CDC−ASC210462107_2021−10−15_AY.107
PA−VSP6121_2021−08−22_AY.107
PA−CDC−STM−WDH3BK6XW_2021−11−08_AY.107
PA−CDC−LC0305692_2021−09−26_AY.107
PA−CDC−ASC210282778_2021−09−18_AY.107
PA−BOL−M21110135_2021−09−22_AY.107
PA−CDC−ASC210409196_2021−09−08_AY.107
PA−CDC−ASC210282775_2021−09−15_AY.107
PA−CDC−LC0172535_2021−08−05_AY.107
PA−CDC−LC0167431_2021−08−03_AY.107
OM542050_VSP10303_2021−12−05_AY.107
OM241380_VSP7867_2021−11−14_AY.107
OM241379_VSP7866_2021−11−14_AY.107
OM541955_VSP10050_2021−11−28_AY.107
OL928823_PA−CDC−ASC210398323_2021−11−11_AY.107
OL760008_PA−CDC−ASC210398323_2021−11−11_AY.107
OL928281_PA−CDC−ASC210398324_2021−11−10_AY.107
OL759466_PA−CDC−ASC210398324_2021−11−10_AY.107
OM016277_PA−CDC−ASC210624639_2021−12−13_AY.107
MN908947 (Wuhan−Hu−1/2019)
0.1

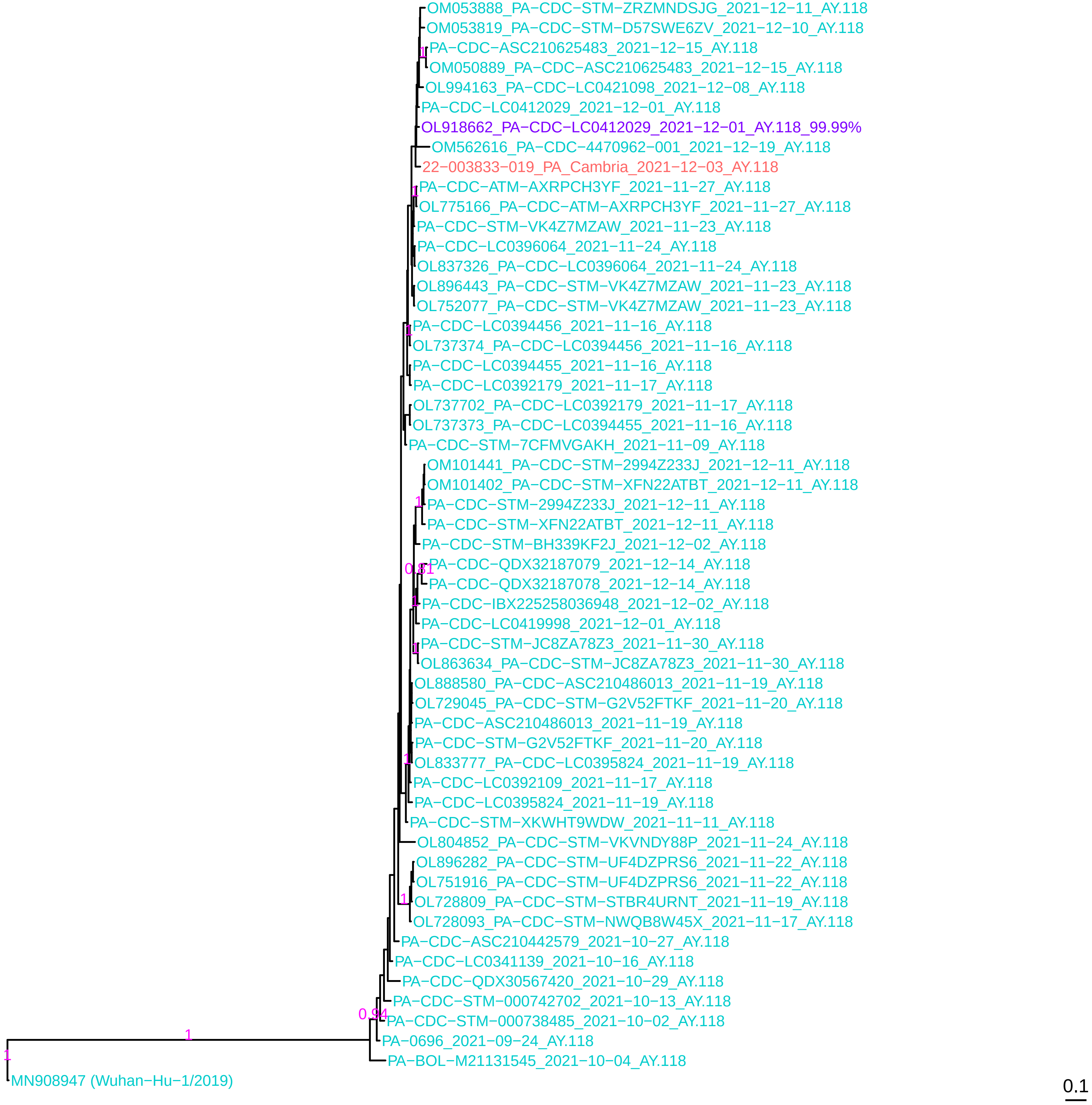
OM053888_PA−CDC−STM−ZRZMNDSJG_2021−12−11_AY.118
OM053819_PA−CDC−STM−D57SWE6ZV_2021−12−10_AY.118
PA−CDC−ASC210625483_2021−12−15_AY.118
OM050889_PA−CDC−ASC210625483_2021−12−15_AY.118
OL994163_PA−CDC−LC0421098_2021−12−08_AY.118
PA−CDC−LC0412029_2021−12−01_AY.118
OL918662_PA−CDC−LC0412029_2021−12−01_AY.118_99.99%
OM562616_PA−CDC−4470962−001_2021−12−19_AY.118
22−003833−019_PA_Cambria_2021−12−03_AY.118
PA−CDC−ATM−AXRPCH3YF_2021−11−27_AY.118
OL775166_PA−CDC−ATM−AXRPCH3YF_2021−11−27_AY.118
PA−CDC−STM−VK4Z7MZAW_2021−11−23_AY.118
PA−CDC−LC0396064_2021−11−24_AY.118
OL837326_PA−CDC−LC0396064_2021−11−24_AY.118
OL896443_PA−CDC−STM−VK4Z7MZAW_2021−11−23_AY.118
OL752077_PA−CDC−STM−VK4Z7MZAW_2021−11−23_AY.118
PA−CDC−LC0394456_2021−11−16_AY.118
OL737374_PA−CDC−LC0394456_2021−11−16_AY.118
PA−CDC−LC0394455_2021−11−16_AY.118
PA−CDC−LC0392179_2021−11−17_AY.118
OL737702_PA−CDC−LC0392179_2021−11−17_AY.118
OL737373_PA−CDC−LC0394455_2021−11−16_AY.118
PA−CDC−STM−7CFMVGAKH_2021−11−09_AY.118
OM101441_PA−CDC−STM−2994Z233J_2021−12−11_AY.118
OM101402_PA−CDC−STM−XFN22ATBT_2021−12−11_AY.118
PA−CDC−STM−2994Z233J_2021−12−11_AY.118
PA−CDC−STM−XFN22ATBT_2021−12−11_AY.118
PA−CDC−STM−BH339KF2J_2021−12−02_AY.118
PA−CDC−QDX32187079_2021−12−14_AY.118
PA−CDC−QDX32187078_2021−12−14_AY.118
PA−CDC−IBX225258036948_2021−12−02_AY.118
PA−CDC−LC0419998_2021−12−01_AY.118
PA−CDC−STM−JC8ZA78Z3_2021−11−30_AY.118
OL863634_PA−CDC−STM−JC8ZA78Z3_2021−11−30_AY.118
OL888580_PA−CDC−ASC210486013_2021−11−19_AY.118
OL729045_PA−CDC−STM−G2V52FTKF_2021−11−20_AY.118
PA−CDC−ASC210486013_2021−11−19_AY.118
PA−CDC−STM−G2V52FTKF_2021−11−20_AY.118
OL833777_PA−CDC−LC0395824_2021−11−19_AY.118
PA−CDC−LC0392109_2021−11−17_AY.118
PA−CDC−LC0395824_2021−11−19_AY.118
PA−CDC−STM−XKWHT9WDW_2021−11−11_AY.118
OL804852_PA−CDC−STM−VKVNDY88P_2021−11−24_AY.118
OL896282_PA−CDC−STM−UF4DZPRS6_2021−11−22_AY.118
OL751916_PA−CDC−STM−UF4DZPRS6_2021−11−22_AY.118
OL728809_PA−CDC−STM−STBR4URNT_2021−11−19_AY.118
OL728093_PA−CDC−STM−NWQB8W45X_2021−11−17_AY.118
PA−CDC−ASC210442579_2021−10−27_AY.118
PA−CDC−LC0341139_2021−10−16_AY.118
PA−CDC−QDX30567420_2021−10−29_AY.118
PA−CDC−STM−000742702_2021−10−13_AY.118
PA−CDC−STM−000738485_2021−10−02_AY.118
PA−0696_2021−09−24_AY.118
PA−BOL−M21131545_2021−10−04_AY.118
MN908947 (Wuhan−Hu−1/2019)
0.1

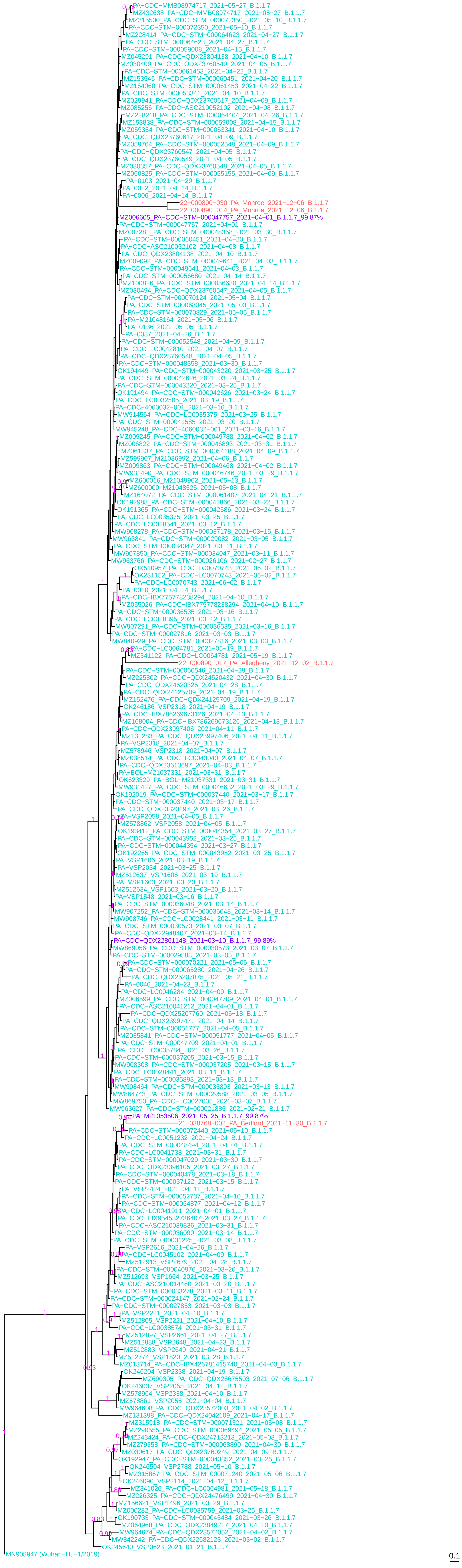
PA-CDC-MMB08974717_2021-05-27_B.1.1.7
MZ432638_PA-CDC-MMB08974717_2021-05-27_B.1.1.7
MZ315500_PA-CDC-STM-000072350_2021-05-10_B.1.1.7
PA-CDC-STM-000072350_2021-05-10_B.1.1.7
MZ228414_PA-CDC-STM-000064623_2021-04-27_B.1.1.7
PA-CDC-STM-000064623_2021-04-27_B.1.1.7
PA-CDC-STM-000059008_2021-04-15_B.1.1.7
MZ045291_PA-CDC-QDX23804138_2021-04-10_B.1.1.7
MZ030409_PA-CDC-QDX23760549_2021-04-05_B.1.1.7
PA-CDC-STM-000061453_2021-04-22_B.1.1.7
MZ153546_PA-CDC-STM-000060451_2021-04-20_B.1.1.7
MZ164060_PA-CDC-STM-000061453_2021-04-22_B.1.1.7
PA-CDC-STM-000053341_2021-04-10_B.1.1.7
MZ029941_PA-CDC-QDX23760617_2021-04-09_B.1.1.7
MZ085256_PA-CDC-ASC210052102_2021-04-08_B.1.1.7
MZ228218_PA-CDC-STM-000064404_2021-04-26_B.1.1.7
MZ153838_PA-CDC-STM-000059008_2021-04-15_B.1.1.7
MZ059354_PA-CDC-STM-000053341_2021-04-10_B.1.1.7
PA-CDC-QDX23760617_2021-04-09_B.1.1.7
MZ059764_PA-CDC-STM-000052548_2021-04-09_B.1.1.7
PA-CDC-QDX23760547_2021-04-05_B.1.1.7
PA-CDC-QDX23760549_2021-04-05_B.1.1.7
MZ030357_PA-CDC-QDX23760548_2021-04-05_B.1.1.7
MZ060825_PA-CDC-STM-000055155_2021-04-09_B.1.1.7
PA-0103_2021-04-29_B.1.1.7
PA-0022_2021-04-14_B.1.1.7
PA-0006_2021-04-14_B.1.1.7
22-000890-030_PA_Monroe_2021-12-06_B.1.1.7
22-000890-014_PA_Monroe_2021-12-06_B.1.1.7
MZ006605_PA-CDC-STM-000047757_2021-04-01_B.1.1.7_99.87%
PA-CDC-STM-000047757_2021-04-01_B.1.1.7
MZ007281_PA-CDC-STM-000048358_2021-03-30_B.1.1.7
PA-CDC-STM-000060451_2021-04-20_B.1.1.7
PA-CDC-ASC210052102_2021-04-08_B.1.1.7
PA-CDC-QDX23804138_2021-04-10_B.1.1.7
MZ009092_PA-CDC-STM-000049641_2021-04-03_B.1.1.7
PA-CDC-STM-000049641_2021-04-03_B.1.1.7
PA-CDC-STM-000056680_2021-04-14_B.1.1.7
MZ100826_PA-CDC-STM-000056680_2021-04-14_B.1.1.7
MZ030494_PA-CDC-QDX23760547_2021-04-05_B.1.1.7
PA-CDC-STM-000070124_2021-05-04_B.1.1.7
PA-CDC-STM-000068045_2021-05-03_B.1.1.7
PA-CDC-STM-000070829_2021-05-05_B.1.1.7
PA-M21048164_2021-05-06_B.1.1.7
PA-0136_2021-05-05_B.1.1.7
PA-0087_2021-04-26_B.1.1.7
PA-CDC-STM-000052548_2021-04-09_B.1.1.7
PA-CDC-LC0042810_2021-04-07_B.1.1.7
PA-CDC-QDX23760548_2021-04-05_B.1.1.7
PA-CDC-STM-000048358_2021-03-30_B.1.1.7
OK194449_PA-CDC-STM-000043220_2021-03-25_B.1.1.7
PA-CDC-STM-000042626_2021-03-24_B.1.1.7
PA-CDC-STM-000043220_2021-03-25_B.1.1.7
OK191494_PA-CDC-STM-000042626_2021-03-24_B.1.1.7
PA-CDC-LC0032505_2021-03-19_B.1.1.7
PA-CDC-4060032-001_2021-03-16_B.1.1.7
MW914564_PA-CDC-LC0035375_2021-03-25_B.1.1.7
PA-CDC-STM-000041585_2021-03-20_B.1.1.7
MW945248_PA-CDC-4060032-001_2021-03-16_B.1.1.7
MZ009245_PA-CDC-STM-000049788_2021-04-02_B.1.1.7
MZ006822_PA-CDC-STM-000046893_2021-03-31_B.1.1.7
MZ061337_PA-CDC-STM-000054188_2021-04-09_B.1.1.7
MZ599907_M21036992_2021-04-06_B.1.1.7
MZ009863_PA-CDC-STM-000049468_2021-04-02_B.1.1.7
MW931490_PA-CDC-STM-000046746_2021-03-29_B.1.1.7
MZ600016_M21049962_2021-05-13_B.1.1.7
MZ600000_M21048525_2021-05-08_B.1.1.7
MZ164072_PA-CDC-STM-000061407_2021-04-21_B.1.1.7
OK192988_PA-CDC-STM-000042866_2021-03-22_B.1.1.7
OK191365_PA-CDC-STM-000042586_2021-03-24_B.1.1.7
PA-CDC-LC0035375_2021-03-25_B.1.1.7
PA-CDC-LC0028541_2021-03-12_B.1.1.7
MW908278_PA-CDC-STM-000037178_2021-03-15_B.1.1.7
MW963841_PA-CDC-STM-000029082_2021-03-05_B.1.1.7
PA-CDC-STM-000034047_2021-03-11_B.1.1.7
MW907850_PA-CDC-STM-000034047_2021-03-11_B.1.1.7
MW963766_PA-CDC-STM-000026106_2021-02-27_B.1.1.7
OK510957_PA-CDC-LC0070743_2021-06-02_B.1.1.7
OK231152_PA-CDC-LC0070743_2021-06-02_B.1.1.7
PA-CDC-LC0070743_2021-06-02_B.1.1.7
PA-0010_2021-04-14_B.1.1.7
PA-CDC-IBX775778238294_2021-04-10_B.1.1.7
MZ055026_PA-CDC-IBX775778238294_2021-04-10_B.1.1.7
PA-CDC-STM-000036535_2021-03-16_B.1.1.7
PA-CDC-LC0028395_2021-03-12_B.1.1.7
MW907291_PA-CDC-STM-000036535_2021-03-16_B.1.1.7
PA-CDC-STM-000027816_2021-03-03_B.1.1.7
MW840929_PA-CDC-STM-000027816_2021-03-03_B.1.1.7
PA-CDC-LC0064781_2021-05-19_B.1.1.7
MZ341122_PA-CDC-LC0064781_2021-05-19_B.1.1.7
22-000890-017_PA_Allegheny_2021-12-02_B.1.1.7
PA-CDC-STM-000066546_2021-04-29_B.1.1.7
MZ225802_PA-CDC-QDX24520432_2021-04-30_B.1.1.7
PA-CDC-QDX24520325_2021-04-28_B.1.1.7
PA-CDC-QDX24125709_2021-04-19_B.1.1.7
MZ152476_PA-CDC-QDX24125709_2021-04-19_B.1.1.7
OK246186_VSP2318_2021-04-19_B.1.1.7
PA-CDC-IBX786269673126_2021-04-13_B.1.1.7
MZ168004_PA-CDC-IBX786269673126_2021-04-13_B.1.1.7
PA-CDC-QDX23997406_2021-04-11_B.1.1.7
MZ131283_PA-CDC-QDX23997406_2021-04-11_B.1.1.7
PA-VSP2318_2021-04-07_B.1.1.7
MZ578946_VSP2318_2021-04-07_B.1.1.7
MZ038514_PA-CDC-LC0043040_2021-04-07_B.1.1.7
PA-CDC-QDX23613697_2021-04-03_B.1.1.7
PA-BOL-M21037331_2021-03-31_B.1.1.7
OK623329_PA-BOL-M21037331_2021-03-31_B.1.1.7
MW931427_PA-CDC-STM-000046632_2021-03-29_B.1.1.7
OK192019_PA-CDC-STM-000037440_2021-03-17_B.1.1.7
PA-CDC-STM-000037440_2021-03-17_B.1.1.7
PA-CDC-QDX23320197_2021-03-26_B.1.1.7
PA-VSP2058_2021-04-05_B.1.1.7
MZ578862_VSP2058_2021-04-05_B.1.1.7
OK193412_PA-CDC-STM-000044354_2021-03-27_B.1.1.7
PA-CDC-STM-000043952_2021-03-25_B.1.1.7
PA-CDC-STM-000044354_2021-03-27_B.1.1.7
OK192265_PA-CDC-STM-000043952_2021-03-25_B.1.1.7
PA-VSP1606_2021-03-19_B.1.1.7
PA-VSP2034_2021-03-25_B.1.1.7
MZ512637_VSP1606_2021-03-19_B.1.1.7
PA-VSP1603_2021-03-20_B.1.1.7
MZ512634_VSP1603_2021-03-20_B.1.1.7
PA-VSP1548_2021-03-16_B.1.1.7
PA-CDC-STM-000036048_2021-03-14_B.1.1.7
MW907252_PA-CDC-STM-000036048_2021-03-14_B.1.1.7
MW908746_PA-CDC-LC0028441_2021-03-11_B.1.1.7
PA-CDC-STM-000030573_2021-03-07_B.1.1.7
PA-CDC-QDX22948407_2021-03-14_B.1.1.7
PA-CDC-QDX22861148_2021-03-10_B.1.1.7_99.89%
MW869056_PA-CDC-STM-000030573_2021-03-07_B.1.1.7
PA-CDC-STM-000029588_2021-03-05_B.1.1.7
PA-CDC-STM-000070221_2021-05-06_B.1.1.7
PA-CDC-STM-000065280_2021-04-26_B.1.1.7
PA-CDC-QDX25207875_2021-05-21_B.1.1.7
PA-0046_2021-04-23_B.1.1.7
PA-CDC-LC0046284_2021-04-09_B.1.1.7
MZ006599_PA-CDC-STM-000047709_2021-04-01_B.1.1.7
PA-CDC-ASC210041212_2021-04-01_B.1.1.7
PA-CDC-QDX25207760_2021-05-18_B.1.1.7
PA-CDC-QDX23997471_2021-04-14_B.1.1.7
PA-CDC-STM-000051777_2021-04-05_B.1.1.7
MZ035841_PA-CDC-STM-000051777_2021-04-05_B.1.1.7
PA-CDC-STM-000047709_2021-04-01_B.1.1.7
PA-CDC-LC0035784_2021-03-26_B.1.1.7
PA-CDC-STM-000037205_2021-03-15_B.1.1.7
MW908308_PA-CDC-STM-000037205_2021-03-15_B.1.1.7
PA-CDC-LC0028441_2021-03-11_B.1.1.7
PA-CDC-STM-000035893_2021-03-13_B.1.1.7
MW908464_PA-CDC-STM-000035893_2021-03-13_B.1.1.7
MW864743_PA-CDC-STM-000029588_2021-03-05_B.1.1.7
MW869750_PA-CDC-LC0027005_2021-03-07_B.1.1.7
MW963627_PA-CDC-STM-000021885_2021-02-21_B.1.1.7
PA-M21053506_2021-05-25_B.1.1.7_99.87%
21-038768-002_PA_Bedford_2021-11-30_B.1.1.7
PA-CDC-STM-000072440_2021-05-10_B.1.1.7
PA-CDC-LC0051232_2021-04-24_B.1.1.7
PA-CDC-STM-000048494_2021-04-01_B.1.1.7
PA-CDC-LC0041738_2021-03-31_B.1.1.7
PA-CDC-STM-000047029_2021-03-30_B.1.1.7
PA-CDC-QDX23396105_2021-03-27_B.1.1.7
PA-CDC-STM-000040478_2021-03-18_B.1.1.7
PA-CDC-STM-000037122_2021-03-15_B.1.1.7
PA-VSP2424_2021-04-11_B.1.1.7
PA-CDC-STM-000052737_2021-04-10_B.1.1.7
PA-CDC-STM-000054877_2021-04-12_B.1.1.7
PA-CDC-LC0041911_2021-04-01_B.1.1.7
PA-CDC-IBX954532736407_2021-03-27_B.1.1.7
PA-CDC-ASC210039836_2021-03-31_B.1.1.7
PA-CDC-STM-000036090_2021-03-14_B.1.1.7
PA-CDC-STM-000031225_2021-03-08_B.1.1.7
PA-VSP2616_2021-04-26_B.1.1.7
PA-CDC-LC0045102_2021-04-09_B.1.1.7
MZ512913_VSP2679_2021-04-28_B.1.1.7
PA-CDC-STM-000040976_2021-03-20_B.1.1.7
MZ512693_VSP1664_2021-03-25_B.1.1.7
PA-CDC-ASC210014460_2021-03-20_B.1.1.7
PA-CDC-STM-000033278_2021-03-11_B.1.1.7
PA-CDC-STM-000024147_2021-02-24_B.1.1.7
PA-CDC-STM-000027853_2021-03-03_B.1.1.7
PA-VSP2221_2021-04-10_B.1.1.7
MZ512805_VSP2221_2021-04-10_B.1.1.7
PA-CDC-LC0038574_2021-03-31_B.1.1.7
MZ512897_VSP2661_2021-04-27_B.1.1.7
MZ512888_VSP2648_2021-04-23_B.1.1.7
MZ512883_VSP2640_2021-04-21_B.1.1.7
MZ512774_VSP1820_2021-03-28_B.1.1.7
MZ013714_PA-CDC-IBX426781415740_2021-04-03_B.1.1.7
OK246204_VSP2338_2021-04-19_B.1.1.7
MZ690305_PA-CDC-QDX26675503_2021-07-06_B.1.1.7
OK246037_VSP2055_2021-04-12_B.1.1.7
MZ578964_VSP2338_2021-04-10_B.1.1.7
MZ578861_VSP2055_2021-04-04_B.1.1.7
MW964608_PA-CDC-QDX23572003_2021-04-02_B.1.1.7
MZ131398_PA-CDC-QDX24042109_2021-04-17_B.1.1.7
MZ315918_PA-CDC-STM-000071321_2021-05-08_B.1.1.7
MZ290555_PA-CDC-STM-000069494_2021-05-05_B.1.1.7
MZ243424_PA-CDC-QDX24713213_2021-05-03_B.1.1.7
MZ279358_PA-CDC-STM-000068890_2021-04-30_B.1.1.7
MZ030617_PA-CDC-QDX23760249_2021-04-09_B.1.1.7
OK192947_PA-CDC-STM-000043352_2021-03-25_B.1.1.7
OK246504_VSP2788_2021-05-10_B.1.1.7
MZ315867_PA-CDC-STM-000071240_2021-05-06_B.1.1.7
OK246090_VSP2114_2021-04-12_B.1.1.7
MZ341026_PA-CDC-LC0064981_2021-05-18_B.1.1.7
MZ226325_PA-CDC-QDX24476499_2021-04-30_B.1.1.7
MZ156621_VSP1496_2021-03-29_B.1.1.7
MZ000282_PA-CDC-LC0035759_2021-03-25_B.1.1.7
OK190733_PA-CDC-STM-000045484_2021-03-26_B.1.1.7
MZ064968_PA-CDC-QDX23849217_2021-04-10_B.1.1.7
MW964674_PA-CDC-QDX23572052_2021-04-02_B.1.1.7
MW842242_PA-CDC-QDX22682123_2021-03-02_B.1.1.7
OK245640_VSP0623_2021-01-21_B.1.1.7
MN908947 (Wuhan-Hu-1/2019)
0.1

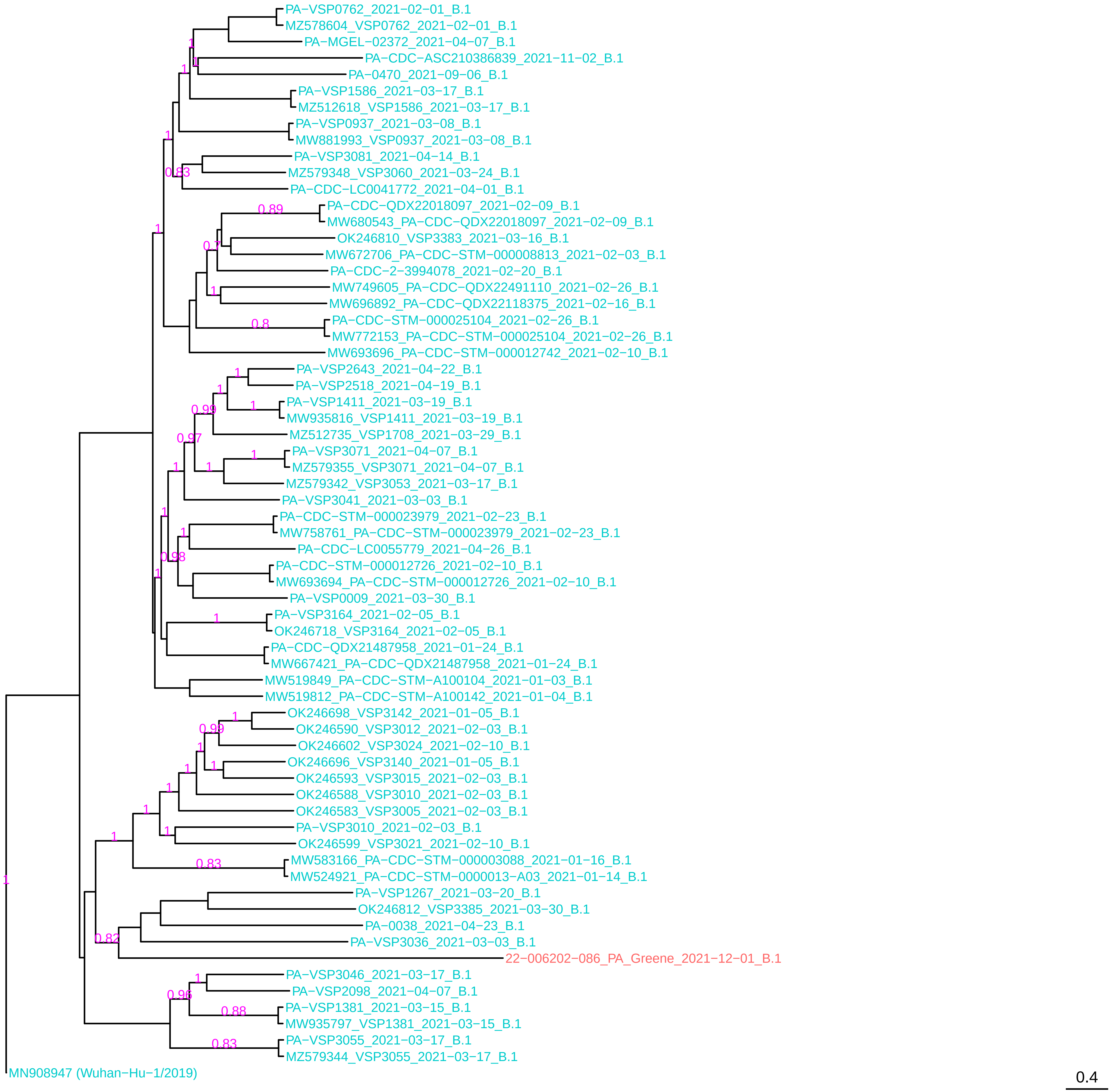
PA-VSP0762_2021-02-01_B.1
MZ578604_VSP0762_2021-02-01_B.1
PA-MGEL-02372_2021-04-07_B.1
PA-CDC-ASC210386839_2021-11-02_B.1
PA-0470_2021-09-06_B.1
PA-VSP1586_2021-03-17_B.1
MZ512618_VSP1586_2021-03-17_B.1
PA-VSP0937_2021-03-08_B.1
MW881993_VSP0937_2021-03-08_B.1
PA-VSP3081_2021-04-14_B.1
MZ579348_VSP3060_2021-03-24_B.1
PA-CDC-LC0041772_2021-04-01_B.1
PA-CDC-QDX22018097_2021-02-09_B.1
MW680543_PA-CDC-QDX22018097_2021-02-09_B.1
OK246810_VSP3383_2021-03-16_B.1
MW672706_PA-CDC-STM-000008813_2021-02-03_B.1
PA-CDC-2-3994078_2021-02-20_B.1
MW749605_PA-CDC-QDX22491110_2021-02-26_B.1
MW696892_PA-CDC-QDX22118375_2021-02-16_B.1
PA-CDC-STM-000025104_2021-02-26_B.1
MW772153_PA-CDC-STM-000025104_2021-02-26_B.1
MW693696_PA-CDC-STM-000012742_2021-02-10_B.1
PA-VSP2643_2021-04-22_B.1
PA-VSP2518_2021-04-19_B.1
PA-VSP1411_2021-03-19_B.1
MW935816_VSP1411_2021-03-19_B.1
MZ512735_VSP1708_2021-03-29_B.1
PA-VSP3071_2021-04-07_B.1
MZ579355_VSP3071_2021-04-07_B.1
MZ579342_VSP3053_2021-03-17_B.1
PA-VSP3041_2021-03-03_B.1
PA-CDC-STM-000023979_2021-02-23_B.1
MW758761_PA-CDC-STM-000023979_2021-02-23_B.1
PA-CDC-LC0055779_2021-04-26_B.1
PA-CDC-STM-000012726_2021-02-10_B.1
MW693694_PA-CDC-STM-000012726_2021-02-10_B.1
PA-VSP0009_2021-03-30_B.1
PA-VSP3164_2021-02-05_B.1
OK246718_VSP3164_2021-02-05_B.1
PA-CDC-QDX21487958_2021-01-24_B.1
MW667421_PA-CDC-QDX21487958_2021-01-24_B.1
MW519849_PA-CDC-STM-A100104_2021-01-03_B.1
MW519812_PA-CDC-STM-A100142_2021-01-04_B.1
OK246698_VSP3142_2021-01-05_B.1
OK246590_VSP3012_2021-02-03_B.1
OK246602_VSP3024_2021-02-10_B.1
OK246696_VSP3140_2021-01-05_B.1
OK246593_VSP3015_2021-02-03_B.1
OK246588_VSP3010_2021-02-03_B.1
OK246583_VSP3005_2021-02-03_B.1
PA-VSP3010_2021-02-03_B.1
OK246599_VSP3021_2021-02-10_B.1
MW583166_PA-CDC-STM-000003088_2021-01-16_B.1
MW524921_PA-CDC-STM-0000013-A03_2021-01-14_B.1
PA-VSP1267_2021-03-20_B.1
OK246812_VSP3385_2021-03-30_B.1
PA-0038_2021-04-23_B.1
PA-VSP3036_2021-03-03_B.1
22-006202-086_PA_Greene_2021-12-01_B.1
PA-VSP3046_2021-03-17_B.1
PA-VSP2098_2021-04-07_B.1
PA-VSP1381_2021-03-15_B.1
MW935797_VSP1381_2021-03-15_B.1
PA-VSP3055_2021-03-17_B.1
MZ579344_VSP3055_2021-03-17_B.1
MN908947 (Wuhan-Hu-1/2019)
0.4

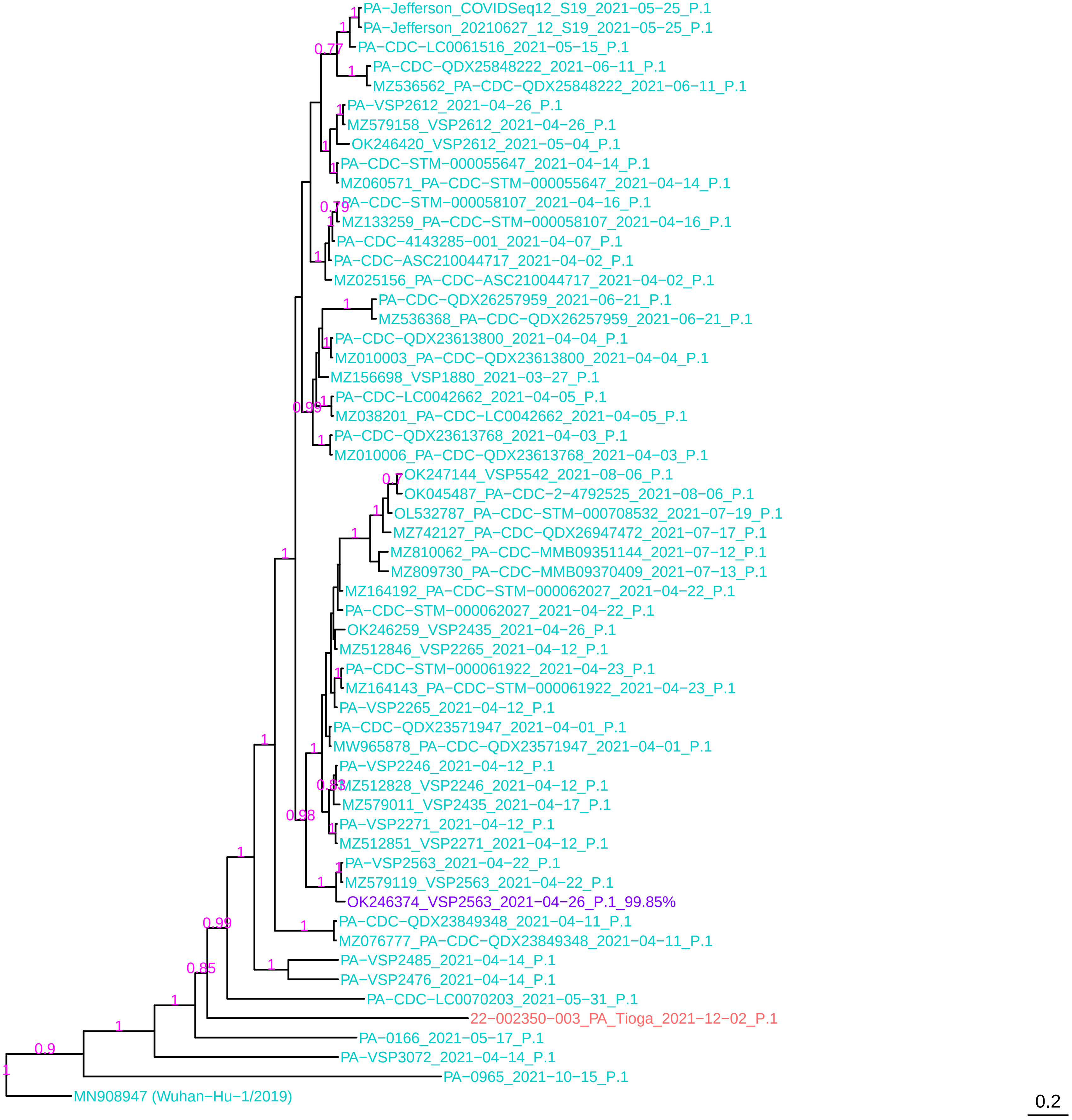
PA−Jefferson_COVIDSeq12_S19_2021−05−25_P.1
PA−Jefferson_20210627_12_S19_2021−05−25_P.1
PA−CDC−LC0061516_2021−05−15_P.1
PA−CDC−QDX25848222_2021−06−11_P.1
MZ536562_PA−CDC−QDX25848222_2021−06−11_P.1
PA−VSP2612_2021−04−26_P.1
MZ579158_VSP2612_2021−04−26_P.1
OK246420_VSP2612_2021−05−04_P.1
PA−CDC−STM−000055647_2021−04−14_P.1
MZ060571_PA−CDC−STM−000055647_2021−04−14_P.1
PA−CDC−STM−000058107_2021−04−16_P.1
MZ133259_PA−CDC−STM−000058107_2021−04−16_P.1
PA−CDC−4143285−001_2021−04−07_P.1
PA−CDC−ASC210044717_2021−04−02_P.1
MZ025156_PA−CDC−ASC210044717_2021−04−02_P.1
PA−CDC−QDX26257959_2021−06−21_P.1
MZ536368_PA−CDC−QDX26257959_2021−06−21_P.1
PA−CDC−QDX23613800_2021−04−04_P.1
MZ010003_PA−CDC−QDX23613800_2021−04−04_P.1
MZ156698_VSP1880_2021−03−27_P.1
PA−CDC−LC0042662_2021−04−05_P.1
MZ038201_PA−CDC−LC0042662_2021−04−05_P.1
PA−CDC−QDX23613768_2021−04−03_P.1
MZ010006_PA−CDC−QDX23613768_2021−04−03_P.1
OK247144_VSP5542_2021−08−06_P.1
OK045487_PA−CDC−2−4792525_2021−08−06_P.1
OL532787_PA−CDC−STM−000708532_2021−07−19_P.1
MZ742127_PA−CDC−QDX26947472_2021−07−17_P.1
MZ810062_PA−CDC−MMB09351144_2021−07−12_P.1
MZ809730_PA−CDC−MMB09370409_2021−07−13_P.1
MZ164192_PA−CDC−STM−000062027_2021−04−22_P.1
PA−CDC−STM−000062027_2021−04−22_P.1
OK246259_VSP2435_2021−04−26_P.1
MZ512846_VSP2265_2021−04−12_P.1
PA−CDC−STM−000061922_2021−04−23_P.1
MZ164143_PA−CDC−STM−000061922_2021−04−23_P.1
PA−VSP2265_2021−04−12_P.1
PA−CDC−QDX23571947_2021−04−01_P.1
MW965878_PA−CDC−QDX23571947_2021−04−01_P.1
PA−VSP2246_2021−04−12_P.1
MZ512828_VSP2246_2021−04−12_P.1
MZ579011_VSP2435_2021−04−17_P.1
PA−VSP2271_2021−04−12_P.1
MZ512851_VSP2271_2021−04−12_P.1
PA−VSP2563_2021−04−22_P.1
MZ579119_VSP2563_2021−04−22_P.1
OK246374_VSP2563_2021−04−26_P.1_99.85%
PA−CDC−QDX23849348_2021−04−11_P.1
MZ076777_PA−CDC−QDX23849348_2021−04−11_P.1
PA−VSP2485_2021−04−14_P.1
PA−VSP2476_2021−04−14_P.1
PA−CDC−LC0070203_2021−05−31_P.1
22−002350−003_PA_Tioga_2021−12−02_P.1
PA−0166_2021−05−17_P.1
PA−VSP3072_2021−04−14_P.1
PA−0965_2021−10−15_P.1
MN908947 (Wuhan−Hu−1/2019)
0.2

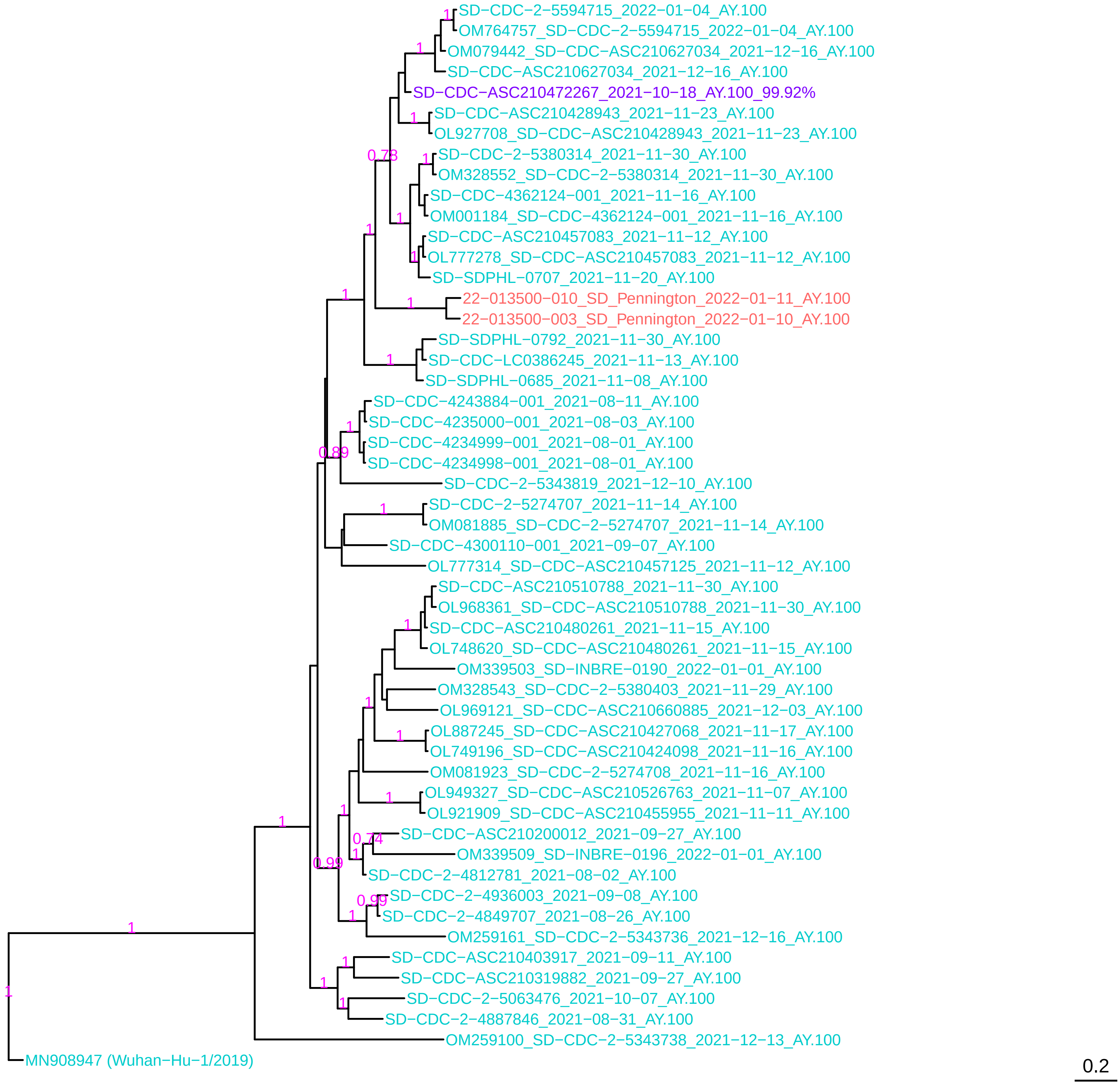

SD−CDC−2−5594715_2022−01−04_AY.100
OM764757_SD−CDC−2−5594715_2022−01−04_AY.100
OM079442_SD−CDC−ASC210627034_2021−12−16_AY.100
SD−CDC−ASC210627034_2021−12−16_AY.100
SD−CDC−ASC210472267_2021−10−18_AY.100_99.92%
SD−CDC−ASC210428943_2021−11−23_AY.100
OL927708_SD−CDC−ASC210428943_2021−11−23_AY.100
SD−CDC−2−5380314_2021−11−30_AY.100
OM328552_SD−CDC−2−5380314_2021−11−30_AY.100
SD−CDC−4362124−001_2021−11−16_AY.100
OM001184_SD−CDC−4362124−001_2021−11−16_AY.100
SD−CDC−ASC210457083_2021−11−12_AY.100
OL777278_SD−CDC−ASC210457083_2021−11−12_AY.100
SD−SDPHL−0707_2021−11−20_AY.100
22−013500−010_SD_Pennington_2022−01−11_AY.100
22−013500−003_SD_Pennington_2022−01−10_AY.100
SD−SDPHL−0792_2021−11−30_AY.100
SD−CDC−LC0386245_2021−11−13_AY.100
SD−SDPHL−0685_2021−11−08_AY.100
SD−CDC−4243884−001_2021−08−11_AY.100
SD−CDC−4235000−001_2021−08−03_AY.100
SD−CDC−4234999−001_2021−08−01_AY.100
SD−CDC−4234998−001_2021−08−01_AY.100
SD−CDC−2−5343819_2021−12−10_AY.100
SD−CDC−2−5274707_2021−11−14_AY.100
OM081885_SD−CDC−2−5274707_2021−11−14_AY.100
SD−CDC−4300110−001_2021−09−07_AY.100
OL777314_SD−CDC−ASC210457125_2021−11−12_AY.100
SD−CDC−ASC210510788_2021−11−30_AY.100
OL968361_SD−CDC−ASC210510788_2021−11−30_AY.100
SD−CDC−ASC210480261_2021−11−15_AY.100
OL748620_SD−CDC−ASC210480261_2021−11−15_AY.100
OM339503_SD−INBRE−0190_2022−01−01_AY.100
OM328543_SD−CDC−2−5380403_2021−11−29_AY.100
OL969121_SD−CDC−ASC210660885_2021−12−03_AY.100
OL887245_SD−CDC−ASC210427068_2021−11−17_AY.100
OL749196_SD−CDC−ASC210424098_2021−11−16_AY.100
OM081923_SD−CDC−2−5274708_2021−11−16_AY.100
OL949327_SD−CDC−ASC210526763_2021−11−07_AY.100
OL921909_SD−CDC−ASC210455955_2021−11−11_AY.100
SD−CDC−ASC210200012_2021−09−27_AY.100
OM339509_SD−INBRE−0196_2022−01−01_AY.100
SD−CDC−2−4812781_2021−08−02_AY.100
SD−CDC−2−4936003_2021−09−08_AY.100
SD−CDC−2−4849707_2021−08−26_AY.100
OM259161_SD−CDC−2−5343736_2021−12−16_AY.100
SD−CDC−ASC210403917_2021−09−11_AY.100
SD−CDC−ASC210319882_2021−09−27_AY.100
SD−CDC−2−5063476_2021−10−07_AY.100
SD−CDC−2−4887846_2021−08−31_AY.100
OM259100_SD−CDC−2−5343738_2021−12−13_AY.100
MN908947 (Wuhan−Hu−1/2019)
0.78
0.89
0.99
0.74
0.99
0.2

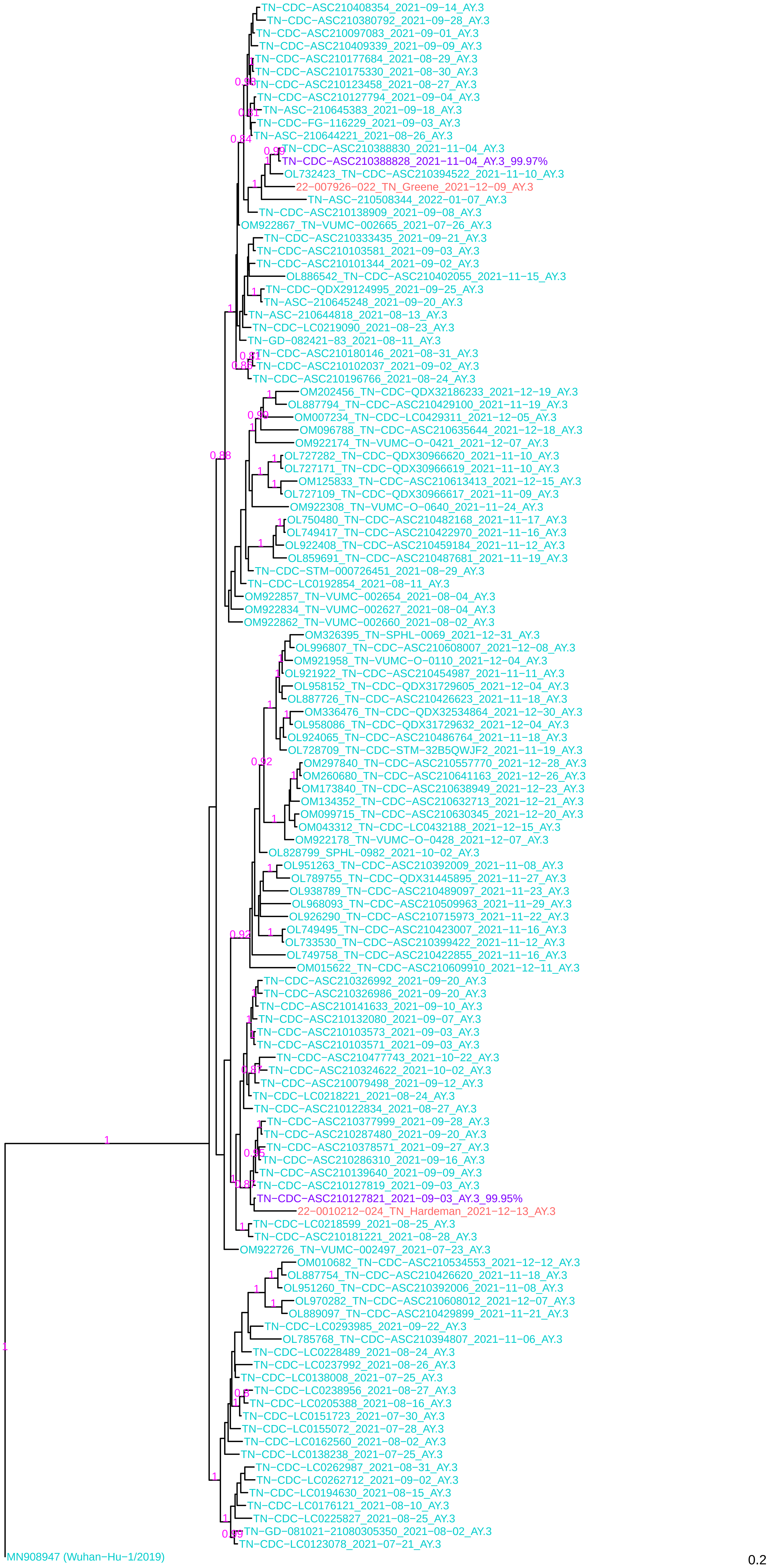
TN−CDC−ASC210408354_2021−09−14_AY.3
TN−CDC−ASC210380792_2021−09−28_AY.3
TN−CDC−ASC210097083_2021−09−01_AY.3
TN−CDC−ASC210409339_2021−09−09_AY.3
TN−CDC−ASC210177684_2021−08−29_AY.3
TN−CDC−ASC210175330_2021−08−30_AY.3
TN−CDC−ASC210123458_2021−08−27_AY.3
TN−CDC−ASC210127794_2021−09−04_AY.3
TN−ASC−210645383_2021−09−18_AY.3
TN−CDC−FG−116229_2021−09−03_AY.3
TN−ASC−210644221_2021−08−26_AY.3
TN−CDC−ASC210388830_2021−11−04_AY.3
TN−CDC−ASC210388828_2021−11−04_AY.3_99.97%
OL732423_TN−CDC−ASC210394522_2021−11−10_AY.3
22−007926−022_TN_Greene_2021−12−09_AY.3
TN−ASC−210508344_2022−01−07_AY.3
TN−CDC−ASC210138909_2021−09−08_AY.3
OM922867_TN−VUMC−002665_2021−07−26_AY.3
TN−CDC−ASC210333435_2021−09−21_AY.3
TN−CDC−ASC210103581_2021−09−03_AY.3
TN−CDC−ASC210101344_2021−09−02_AY.3
OL886542_TN−CDC−ASC210402055_2021−11−15_AY.3
TN−CDC−QDX29124995_2021−09−25_AY.3
TN−ASC−210645248_2021−09−20_AY.3
TN−ASC−210644818_2021−08−13_AY.3
TN−CDC−LC0219090_2021−08−23_AY.3
TN−GD−082421−83_2021−08−11_AY.3
TN−CDC−ASC210180146_2021−08−31_AY.3
TN−CDC−ASC210102037_2021−09−02_AY.3
TN−CDC−ASC210196766_2021−08−24_AY.3
OM202456_TN−CDC−QDX32186233_2021−12−19_AY.3
OL887794_TN−CDC−ASC210429100_2021−11−19_AY.3
OM007234_TN−CDC−LC0429311_2021−12−05_AY.3
OM096788_TN−CDC−ASC210635644_2021−12−18_AY.3
OM922174_TN−VUMC−O−0421_2021−12−07_AY.3
OL727282_TN−CDC−QDX30966620_2021−11−10_AY.3
OL727171_TN−CDC−QDX30966619_2021−11−10_AY.3
OM125833_TN−CDC−ASC210613413_2021−12−15_AY.3
OL727109_TN−CDC−QDX30966617_2021−11−09_AY.3
OM922308_TN−VUMC−O−0640_2021−11−24_AY.3
OL750480_TN−CDC−ASC210482168_2021−11−17_AY.3
OL749417_TN−CDC−ASC210422970_2021−11−16_AY.3
OL922408_TN−CDC−ASC210459184_2021−11−12_AY.3
OL859691_TN−CDC−ASC210487681_2021−11−19_AY.3
TN−CDC−STM−000726451_2021−08−29_AY.3
TN−CDC−LC0192854_2021−08−11_AY.3
OM922857_TN−VUMC−002654_2021−08−04_AY.3
OM922834_TN−VUMC−002627_2021−08−04_AY.3
OM922862_TN−VUMC−002660_2021−08−02_AY.3
OM326395_TN−SPHL−0069_2021−12−31_AY.3
OL996807_TN−CDC−ASC210608007_2021−12−08_AY.3
OM921958_TN−VUMC−O−0110_2021−12−04_AY.3
OL921922_TN−CDC−ASC210454987_2021−11−11_AY.3
OL958152_TN−CDC−QDX31729605_2021−12−04_AY.3
OL887726_TN−CDC−ASC210426623_2021−11−18_AY.3
OM336476_TN−CDC−QDX32534864_2021−12−30_AY.3
OL958086_TN−CDC−QDX31729632_2021−12−04_AY.3
OL924065_TN−CDC−ASC210486764_2021−11−18_AY.3
OL728709_TN−CDC−STM−32B5QWJF2_2021−11−19_AY.3
OM297840_TN−CDC−ASC210557770_2021−12−28_AY.3
OM260680_TN−CDC−ASC210641163_2021−12−26_AY.3
OM173840_TN−CDC−ASC210638949_2021−12−23_AY.3
OM134352_TN−CDC−ASC210632713_2021−12−21_AY.3
OM099715_TN−CDC−ASC210630345_2021−12−20_AY.3
OM043312_TN−CDC−LC0432188_2021−12−15_AY.3
OM922178_TN−VUMC−O−0428_2021−12−07_AY.3
OL828799_SPHL−0982_2021−10−02_AY.3
OL951263_TN−CDC−ASC210392009_2021−11−08_AY.3
OL789755_TN−CDC−QDX31445895_2021−11−27_AY.3
OL938789_TN−CDC−ASC210489097_2021−11−23_AY.3
OL968093_TN−CDC−ASC210509963_2021−11−29_AY.3
OL926290_TN−CDC−ASC210715973_2021−11−22_AY.3
OL749495_TN−CDC−ASC210423007_2021−11−16_AY.3
OL733530_TN−CDC−ASC210399422_2021−11−12_AY.3
OL749758_TN−CDC−ASC210422855_2021−11−16_AY.3
OM015622_TN−CDC−ASC210609910_2021−12−11_AY.3
TN−CDC−ASC210326992_2021−09−20_AY.3
TN−CDC−ASC210326986_2021−09−20_AY.3
TN−CDC−ASC210141633_2021−09−10_AY.3
TN−CDC−ASC210132080_2021−09−07_AY.3
TN−CDC−ASC210103573_2021−09−03_AY.3
TN−CDC−ASC210103571_2021−09−03_AY.3
TN−CDC−ASC210477743_2021−10−22_AY.3
TN−CDC−ASC210324622_2021−10−02_AY.3
TN−CDC−ASC210079498_2021−09−12_AY.3
TN−CDC−LC0218221_2021−08−24_AY.3
TN−CDC−ASC210122834_2021−08−27_AY.3
TN−CDC−ASC210377999_2021−09−28_AY.3
TN−CDC−ASC210287480_2021−09−20_AY.3
TN−CDC−ASC210378571_2021−09−27_AY.3
TN−CDC−ASC210286310_2021−09−16_AY.3
TN−CDC−ASC210139640_2021−09−09_AY.3
TN−CDC−ASC210127819_2021−09−03_AY.3
TN−CDC−ASC210127821_2021−09−03_AY.3_99.95%
22−0010212−024_TN_Hardeman_2021−12−13_AY.3
TN−CDC−LC0218599_2021−08−25_AY.3
TN−CDC−ASC210181221_2021−08−28_AY.3
OM922726_TN−VUMC−002497_2021−07−23_AY.3
OM010682_TN−CDC−ASC210534553_2021−12−12_AY.3
OL887754_TN−CDC−ASC210426620_2021−11−18_AY.3
OL951260_TN−CDC−ASC210392006_2021−11−08_AY.3
OL970282_TN−CDC−ASC210608012_2021−12−07_AY.3
OL889097_TN−CDC−ASC210429899_2021−11−21_AY.3
TN−CDC−LC0293985_2021−09−22_AY.3
OL785768_TN−CDC−ASC210394807_2021−11−06_AY.3
TN−CDC−LC0228489_2021−08−24_AY.3
TN−CDC−LC0237992_2021−08−26_AY.3
TN−CDC−LC0138008_2021−07−25_AY.3
TN−CDC−LC0238956_2021−08−27_AY.3
TN−CDC−LC0205388_2021−08−16_AY.3
TN−CDC−LC0151723_2021−07−30_AY.3
TN−CDC−LC0155072_2021−07−28_AY.3
TN−CDC−LC0162560_2021−08−02_AY.3
TN−CDC−LC0138238_2021−07−25_AY.3
TN−CDC−LC0262987_2021−08−31_AY.3
TN−CDC−LC0262712_2021−09−02_AY.3
TN−CDC−LC0194630_2021−08−15_AY.3
TN−CDC−LC0176121_2021−08−10_AY.3
TN−CDC−LC0225827_2021−08−25_AY.3
TN−GD−081021−21080305350_2021−08−02_AY.3
TN−CDC−LC0123078_2021−07−21_AY.3
MN908947 (Wuhan−Hu−1/2019)
0.2

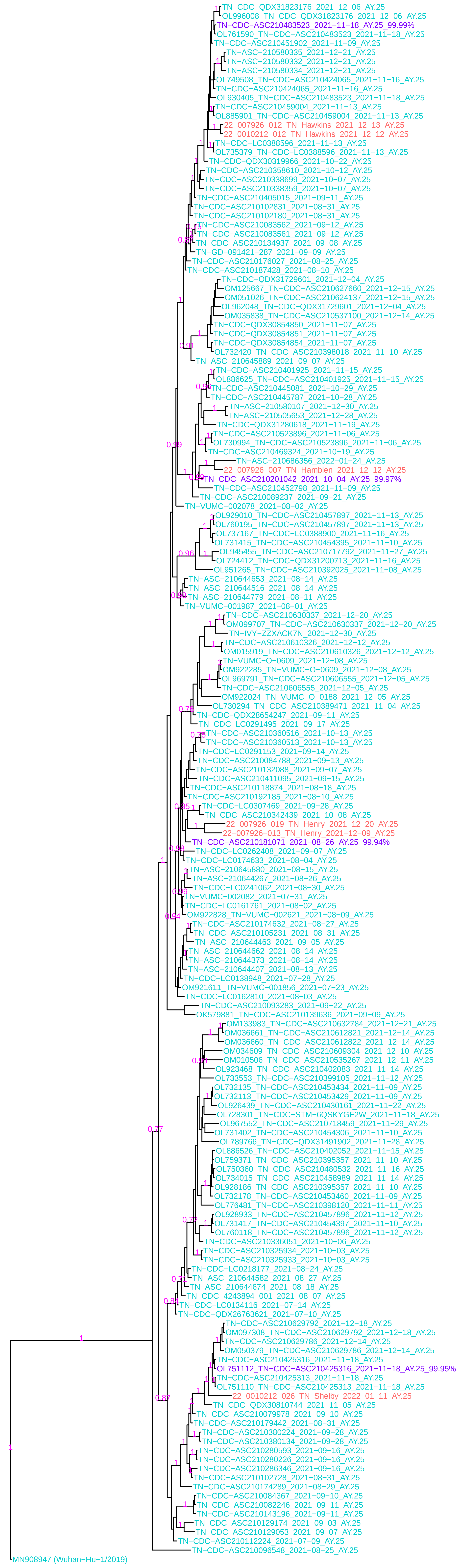
TN-CDC-QDX31823176_2021-12-06_AY.25
OL996008_TN-CDC-QDX31823176_2021-12-06_AY.25
TN-CDC-ASC210483523_2021-11-18_AY.25_99.99%
OL761590_TN-CDC-ASC210483523_2021-11-18_AY.25
TN-CDC-ASC210451902_2021-11-09_AY.25
TN-ASC-210580335_2021-12-21_AY.25
TN-ASC-210580332_2021-12-21_AY.25
TN-ASC-210580334_2021-12-21_AY.25
OL749508_TN-CDC-ASC210424065_2021-11-16_AY.25
TN-CDC-ASC210424065_2021-11-16_AY.25
OL930405_TN-CDC-ASC210483523_2021-11-18_AY.25
TN-CDC-ASC210459004_2021-11-13_AY.25
OL885901_TN-CDC-ASC210459004_2021-11-13_AY.25
22-007926-012_TN_Hawkins_2021-12-13_AY.25
22-0010212-012_TN_Hawkins_2021-12-12_AY.25
TN-CDC-LC0388596_2021-11-13_AY.25
OL735379_TN-CDC-LC0388596_2021-11-13_AY.25
TN-CDC-QDX30319966_2021-10-22_AY.25
TN-CDC-ASC210358610_2021-10-12_AY.25
TN-CDC-ASC210338699_2021-10-07_AY.25
TN-CDC-ASC210338359_2021-10-07_AY.25
TN-CDC-ASC210405015_2021-09-11_AY.25
TN-CDC-ASC210102831_2021-08-31_AY.25
TN-CDC-ASC210102180_2021-08-31_AY.25
TN-CDC-ASC210083562_2021-09-12_AY.25
TN-CDC-ASC210083561_2021-09-12_AY.25
TN-CDC-ASC210134937_2021-09-08_AY.25
TN-GD-091421-287_2021-09-09_AY.25
TN-CDC-ASC210176027_2021-08-25_AY.25
TN-CDC-ASC210187428_2021-08-10_AY.25
TN-CDC-QDX31729601_2021-12-04_AY.25
OM125667_TN-CDC-ASC210627660_2021-12-15_AY.25
OM051026_TN-CDC-ASC210624137_2021-12-15_AY.25
OL962048_TN-CDC-QDX31729601_2021-12-04_AY.25
OM035838_TN-CDC-ASC210537100_2021-12-14_AY.25
TN-CDC-QDX30854850_2021-11-07_AY.25
TN-CDC-QDX30854851_2021-11-07_AY.25
TN-CDC-QDX30854854_2021-11-07_AY.25
OL732420_TN-CDC-ASC210398018_2021-11-10_AY.25
TN-ASC-210645889_2021-09-07_AY.25
TN-CDC-ASC210401925_2021-11-15_AY.25
OL886625_TN-CDC-ASC210401925_2021-11-15_AY.25
TN-CDC-ASC210445081_2021-10-29_AY.25
TN-CDC-ASC210445787_2021-10-28_AY.25
TN-ASC-210580107_2021-12-30_AY.25
TN-ASC-210505653_2021-12-28_AY.25
TN-CDC-QDX31280618_2021-11-19_AY.25
TN-CDC-ASC210523896_2021-11-06_AY.25
OL730994_TN-CDC-ASC210523896_2021-11-06_AY.25
TN-CDC-ASC210469324_2021-10-19_AY.25
TN-ASC-210686356_2022-01-24_AY.25
22-007926-007_TN_Hamblen_2021-12-12_AY.25
TN-CDC-ASC210201042_2021-10-04_AY.25_99.97%
TN-CDC-ASC210452798_2021-11-09_AY.25
TN-CDC-ASC210089237_2021-09-21_AY.25
TN-VUMC-002078_2021-08-02_AY.25
OL929010_TN-CDC-ASC210457897_2021-11-13_AY.25
OL760195_TN-CDC-ASC210457897_2021-11-13_AY.25
OL737167_TN-CDC-LC0388900_2021-11-16_AY.25
OL731415_TN-CDC-ASC210454395_2021-11-10_AY.25
OL945455_TN-CDC-ASC210717792_2021-11-27_AY.25
OL724412_TN-CDC-QDX31200713_2021-11-16_AY.25
OL951265_TN-CDC-ASC210392025_2021-11-08_AY.25
TN-ASC-210644653_2021-08-14_AY.25
TN-ASC-210644516_2021-08-14_AY.25
TN-ASC-210644779_2021-08-11_AY.25
TN-VUMC-001987_2021-08-01_AY.25
TN-CDC-ASC210630337_2021-12-20_AY.25
OM099707_TN-CDC-ASC210630337_2021-12-20_AY.25
TN-IVY-ZZXACK7N_2021-12-30_AY.25
TN-CDC-ASC210610326_2021-12-12_AY.25
OM015919_TN-CDC-ASC210610326_2021-12-12_AY.25
TN-VUMC-O-0609_2021-12-08_AY.25
OM922285_TN-VUMC-O-0609_2021-12-08_AY.25
OL969791_TN-CDC-ASC210606555_2021-12-05_AY.25
TN-CDC-ASC210606555_2021-12-05_AY.25
OM922024_TN-VUMC-O-0188_2021-12-05_AY.25
OL730294_TN-CDC-ASC210389471_2021-11-04_AY.25
TN-CDC-QDX28654247_2021-09-11_AY.25
TN-CDC-LC0291495_2021-09-17_AY.25
TN-CDC-ASC210360516_2021-10-13_AY.25
TN-CDC-ASC210360513_2021-10-13_AY.25
TN-CDC-LC0291153_2021-09-14_AY.25
TN-CDC-ASC210084788_2021-09-13_AY.25
TN-CDC-ASC210132088_2021-09-07_AY.25
TN-CDC-ASC210411095_2021-09-15_AY.25
TN-CDC-ASC210118874_2021-08-18_AY.25
TN-CDC-ASC210192185_2021-08-10_AY.25
TN-CDC-LC0307469_2021-09-28_AY.25
TN-CDC-ASC210342439_2021-10-08_AY.25
22-007926-019_TN_Henry_2021-12-20_AY.25
22-007926-013_TN_Henry_2021-12-09_AY.25
TN-CDC-ASC210181071_2021-08-26_AY.25_99.94%
TN-CDC-LC0262408_2021-09-07_AY.25
TN-CDC-LC0174633_2021-08-04_AY.25
TN-ASC-210645880_2021-08-15_AY.25
TN-ASC-210644267_2021-08-26_AY.25
TN-CDC-LC0241062_2021-08-30_AY.25
TN-VUMC-002082_2021-07-31_AY.25
TN-CDC-LC0161761_2021-08-02_AY.25
OM922828_TN-VUMC-002621_2021-08-09_AY.25
TN-CDC-ASC210174632_2021-08-27_AY.25
TN-CDC-ASC210105231_2021-08-31_AY.25
TN-ASC-210644463_2021-09-05_AY.25
TN-ASC-210644662_2021-08-14_AY.25
TN-ASC-210644373_2021-08-14_AY.25
TN-ASC-210644407_2021-08-13_AY.25
TN-CDC-LC0138948_2021-07-28_AY.25
OM921611_TN-VUMC-001856_2021-07-23_AY.25
TN-CDC-LC0162810_2021-08-03_AY.25
TN-CDC-ASC210093283_2021-09-22_AY.25
OK579881_TN-CDC-ASC210139636_2021-09-09_AY.25
OM133983_TN-CDC-ASC210632784_2021-12-21_AY.25
OM036661_TN-CDC-ASC210612821_2021-12-14_AY.25
OM036660_TN-CDC-ASC210612822_2021-12-14_AY.25
OM034609_TN-CDC-ASC210609304_2021-12-10_AY.25
OM010506_TN-CDC-ASC210535267_2021-12-11_AY.25
OL923468_TN-CDC-ASC210402083_2021-11-14_AY.25
OL733553_TN-CDC-ASC210399105_2021-11-12_AY.25
OL732135_TN-CDC-ASC210453434_2021-11-09_AY.25
OL732113_TN-CDC-ASC210453429_2021-11-09_AY.25
OL926439_TN-CDC-ASC210430161_2021-11-22_AY.25
OL728301_TN-CDC-STM-6QSKYGF2W_2021-11-18_AY.25
OL967552_TN-CDC-ASC210718459_2021-11-29_AY.25
OL731402_TN-CDC-ASC210454306_2021-11-10_AY.25
OL789766_TN-CDC-QDX31491902_2021-11-28_AY.25
OL886526_TN-CDC-ASC210402052_2021-11-15_AY.25
OL759371_TN-CDC-ASC210395357_2021-11-10_AY.25
OL750360_TN-CDC-ASC210480532_2021-11-16_AY.25
OL734015_TN-CDC-ASC210458989_2021-11-14_AY.25
OL928186_TN-CDC-ASC210395357_2021-11-10_AY.25
OL732178_TN-CDC-ASC210453460_2021-11-09_AY.25
OL776481_TN-CDC-ASC210398120_2021-11-11_AY.25
OL928933_TN-CDC-ASC210457896_2021-11-12_AY.25
OL731417_TN-CDC-ASC210454397_2021-11-10_AY.25
OL760118_TN-CDC-ASC210457896_2021-11-12_AY.25
TN-CDC-ASC210336051_2021-10-06_AY.25
TN-CDC-ASC210325934_2021-10-03_AY.25
TN-CDC-ASC210325933_2021-10-03_AY.25
TN-CDC-LC0218177_2021-08-24_AY.25
TN-ASC-210644582_2021-08-27_AY.25
TN-ASC-210644674_2021-08-18_AY.25
TN-CDC-4243894-001_2021-08-07_AY.25
TN-CDC-LC0134116_2021-07-14_AY.25
TN-CDC-QDX26763621_2021-07-10_AY.25
TN-CDC-ASC210629792_2021-12-18_AY.25
OM097308_TN-CDC-ASC210629792_2021-12-18_AY.25
TN-CDC-ASC210629786_2021-12-14_AY.25
OM050379_TN-CDC-ASC210629786_2021-12-14_AY.25
TN-CDC-ASC210425316_2021-11-18_AY.25
OL751112_TN-CDC-ASC210425316_2021-11-18_AY.25_99.95%
TN-CDC-ASC210425313_2021-11-18_AY.25
OL751110_TN-CDC-ASC210425313_2021-11-18_AY.25
22-0010212-026_TN_Shelby_2022-01-11_AY.25
TN-CDC-QDX30810744_2021-11-05_AY.25
TN-CDC-ASC210079978_2021-09-10_AY.25
TN-CDC-ASC210179442_2021-08-31_AY.25
TN-CDC-ASC210380224_2021-09-28_AY.25
TN-CDC-ASC210380134_2021-09-28_AY.25
TN-CDC-ASC210280593_2021-09-16_AY.25
TN-CDC-ASC210280226_2021-09-16_AY.25
TN-CDC-ASC210286346_2021-09-16_AY.25
TN-CDC-ASC210102728_2021-08-31_AY.25
TN-CDC-ASC210174289_2021-08-29_AY.25
TN-CDC-ASC210084367_2021-09-10_AY.25
TN-CDC-ASC210082246_2021-09-11_AY.25
TN-CDC-ASC210143196_2021-09-11_AY.25
TN-CDC-ASC210129174_2021-09-03_AY.25
TN-CDC-ASC210129053_2021-09-07_AY.25
TN-CDC-ASC210112224_2021-07-09_AY.25
TN-CDC-ASC210096548_2021-08-25_AY.25
MN908947 (Wuhan-Hu-1/2019)
0.2

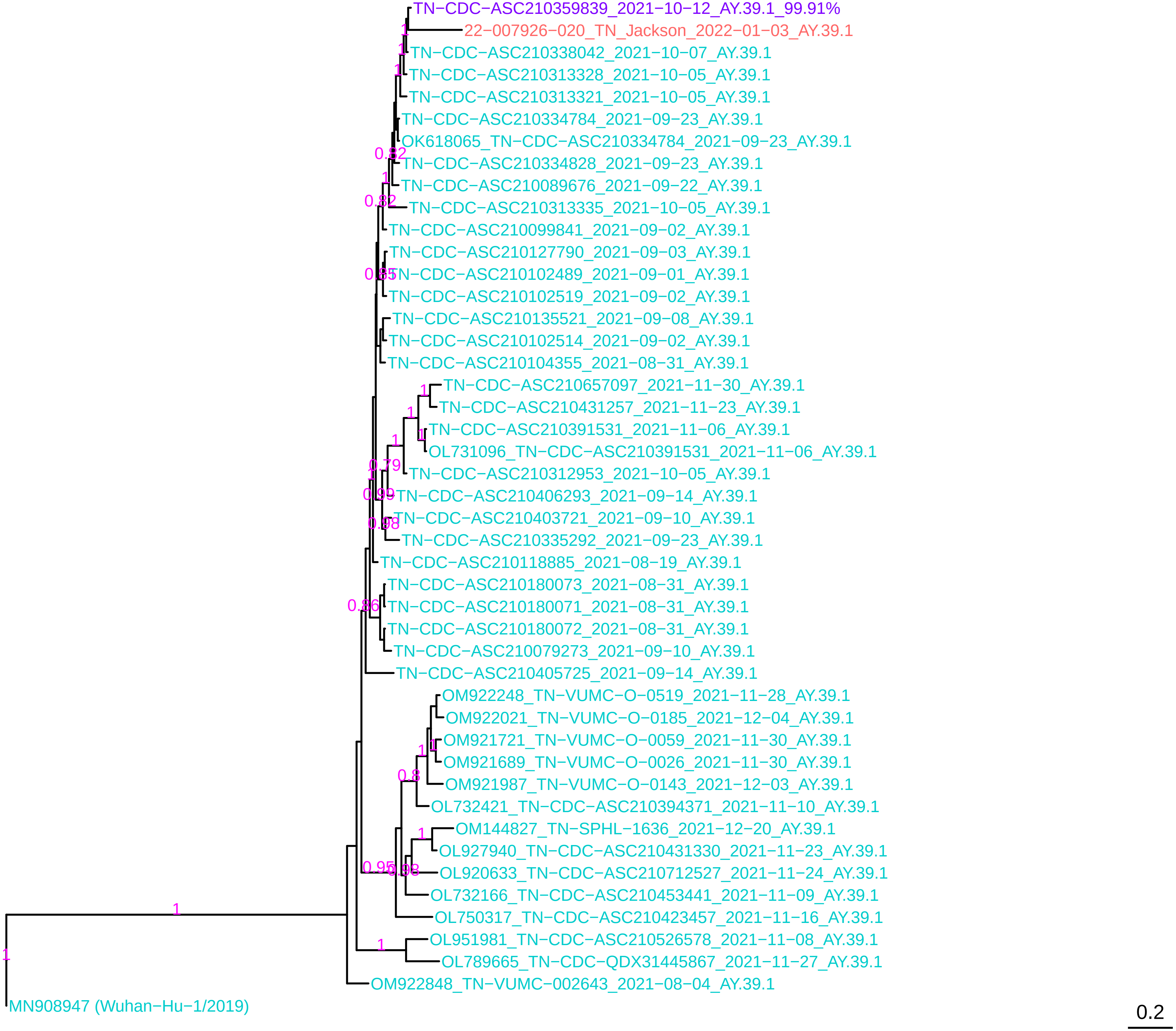
TN-CDC-ASC210359839_2021-10-12_AY.39.1_99.91%
22-007926-020_TN_Jackson_2022-01-03_AY.39.1
TN-CDC-ASC210338042_2021-10-07_AY.39.1
TN-CDC-ASC210313328_2021-10-05_AY.39.1
TN-CDC-ASC210313321_2021-10-05_AY.39.1
TN-CDC-ASC210334784_2021-09-23_AY.39.1
OK618065_TN-CDC-ASC210334784_2021-09-23_AY.39.1
TN-CDC-ASC210334828_2021-09-23_AY.39.1
TN-CDC-ASC210089676_2021-09-22_AY.39.1
TN-CDC-ASC210313335_2021-10-05_AY.39.1
TN-CDC-ASC210099841_2021-09-02_AY.39.1
TN-CDC-ASC210127790_2021-09-03_AY.39.1
TN-CDC-ASC210102489_2021-09-01_AY.39.1
TN-CDC-ASC210102519_2021-09-02_AY.39.1
TN-CDC-ASC210135521_2021-09-08_AY.39.1
TN-CDC-ASC210102514_2021-09-02_AY.39.1
TN-CDC-ASC210104355_2021-08-31_AY.39.1
TN-CDC-ASC210657097_2021-11-30_AY.39.1
TN-CDC-ASC210431257_2021-11-23_AY.39.1
TN-CDC-ASC210391531_2021-11-06_AY.39.1
OL731096_TN-CDC-ASC210391531_2021-11-06_AY.39.1
TN-CDC-ASC210312953_2021-10-05_AY.39.1
TN-CDC-ASC210406293_2021-09-14_AY.39.1
TN-CDC-ASC210403721_2021-09-10_AY.39.1
TN-CDC-ASC210335292_2021-09-23_AY.39.1
TN-CDC-ASC210118885_2021-08-19_AY.39.1
TN-CDC-ASC210180073_2021-08-31_AY.39.1
TN-CDC-ASC210180071_2021-08-31_AY.39.1
TN-CDC-ASC210180072_2021-08-31_AY.39.1
TN-CDC-ASC210079273_2021-09-10_AY.39.1
TN-CDC-ASC210405725_2021-09-14_AY.39.1
OM922248_TN-VUMC-O-0519_2021-11-28_AY.39.1
OM922021_TN-VUMC-O-0185_2021-12-04_AY.39.1
OM921721_TN-VUMC-O-0059_2021-11-30_AY.39.1
OM921689_TN-VUMC-O-0026_2021-11-30_AY.39.1
OM921987_TN-VUMC-O-0143_2021-12-03_AY.39.1
OL732421_TN-CDC-ASC210394371_2021-11-10_AY.39.1
OM144827_TN-SPHL-1636_2021-12-20_AY.39.1
OL927940_TN-CDC-ASC210431330_2021-11-23_AY.39.1
OL920633_TN-CDC-ASC210712527_2021-11-24_AY.39.1
OL732166_TN-CDC-ASC210453441_2021-11-09_AY.39.1
OL750317_TN-CDC-ASC210423457_2021-11-16_AY.39.1
OL951981_TN-CDC-ASC210526578_2021-11-08_AY.39.1
OL789665_TN-CDC-QDX31445867_2021-11-27_AY.39.1
OM922848_TN-VUMC-002643_2021-08-04_AY.39.1
MN908947 (Wuhan-Hu-1/2019)
0.2

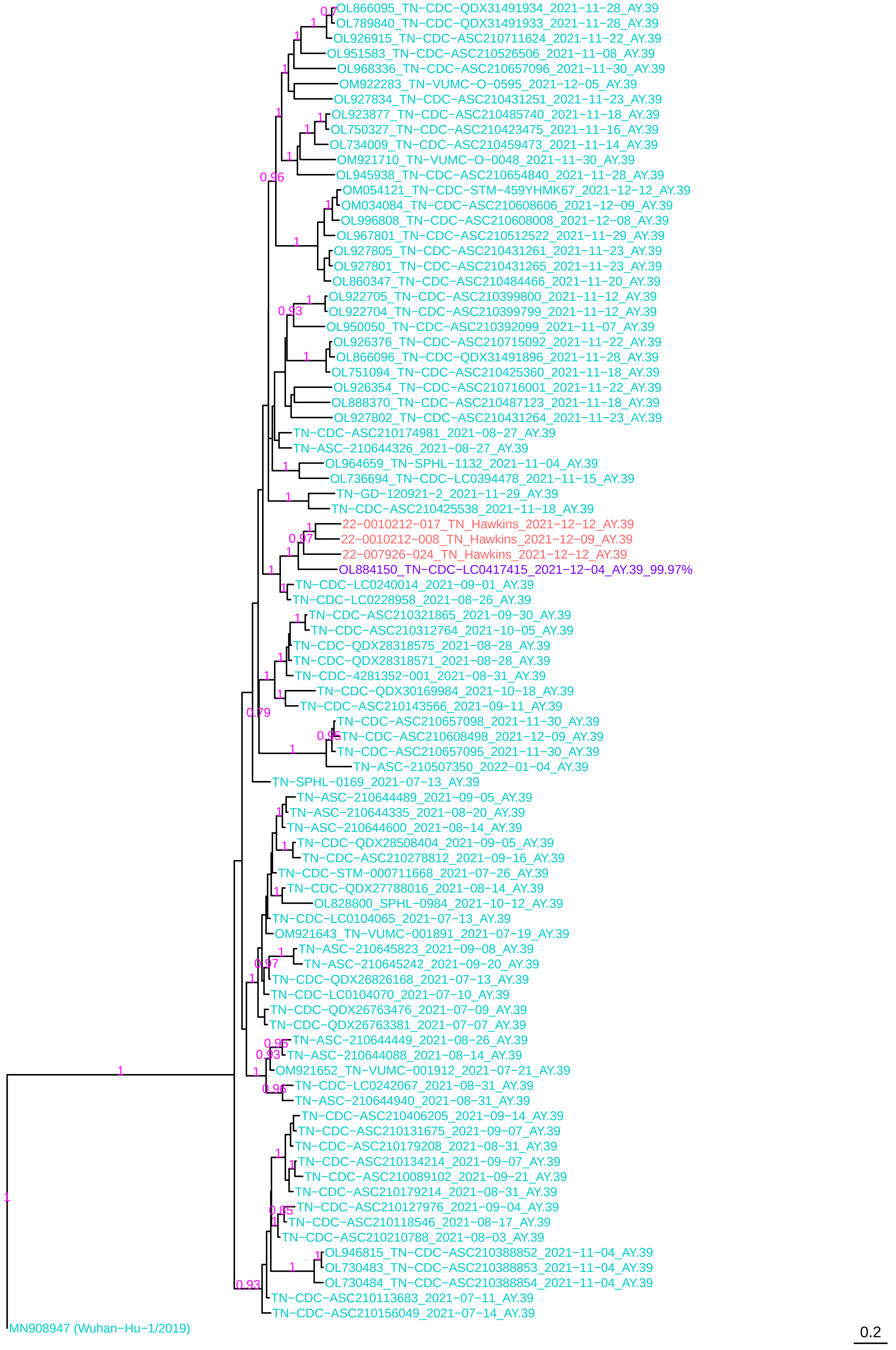
OL866095_TN−CDC−QDX31491934_2021−11−28_AY.39
OL789840_TN−CDC−QDX31491933_2021−11−28_AY.39
OL926915_TN−CDC−ASC210711624_2021−11−22_AY.39
OL951583_TN−CDC−ASC210526506_2021−11−08_AY.39
OL968336_TN−CDC−ASC210657096_2021−11−30_AY.39
OM922283_TN−VUMC−O−0595_2021−12−05_AY.39
OL927834_TN−CDC−ASC210431251_2021−11−23_AY.39
OL923877_TN−CDC−ASC210485740_2021−11−18_AY.39
OL750327_TN−CDC−ASC210423475_2021−11−16_AY.39
OL734009_TN−CDC−ASC210459473_2021−11−14_AY.39
OM921710_TN−VUMC−O−0048_2021−11−30_AY.39
OL945938_TN−CDC−ASC210654840_2021−11−28_AY.39
OM054121_TN−CDC−STM−459YHMK67_2021−12−12_AY.39
OM034084_TN−CDC−ASC210608606_2021−12−09_AY.39
OL996808_TN−CDC−ASC210608008_2021−12−08_AY.39
OL967801_TN−CDC−ASC210512522_2021−11−29_AY.39
OL927805_TN−CDC−ASC210431261_2021−11−23_AY.39
OL927801_TN−CDC−ASC210431265_2021−11−23_AY.39
OL860347_TN−CDC−ASC210484466_2021−11−20_AY.39
OL922705_TN−CDC−ASC210399800_2021−11−12_AY.39
OL922704_TN−CDC−ASC210399799_2021−11−12_AY.39
OL950050_TN−CDC−ASC210392099_2021−11−07_AY.39
OL926376_TN−CDC−ASC210715092_2021−11−22_AY.39
OL866096_TN−CDC−QDX31491896_2021−11−28_AY.39
OL751094_TN−CDC−ASC210425360_2021−11−18_AY.39
OL926354_TN−CDC−ASC210716001_2021−11−22_AY.39
OL888370_TN−CDC−ASC210487123_2021−11−18_AY.39
OL927802_TN−CDC−ASC210431264_2021−11−23_AY.39
TN−CDC−ASC210174981_2021−08−27_AY.39
TN−ASC−210644326_2021−08−27_AY.39
OL964659_TN−SPHL−1132_2021−11−04_AY.39
OL736694_TN−CDC−LC0394478_2021−11−15_AY.39
TN−GD−120921−2_2021−11−29_AY.39
TN−CDC−ASC210425538_2021−11−18_AY.39
22−0010212−017_TN_Hawkins_2021−12−12_AY.39
22−0010212−008_TN_Hawkins_2021−12−09_AY.39
22−007926−024_TN_Hawkins_2021−12−12_AY.39
OL884150_TN−CDC−LC0417415_2021−12−04_AY.39_99.97%
TN−CDC−LC0240014_2021−09−01_AY.39
TN−CDC−LC0228958_2021−08−26_AY.39
TN−CDC−ASC210321865_2021−09−30_AY.39
TN−CDC−ASC210312764_2021−10−05_AY.39
TN−CDC−QDX28318575_2021−08−28_AY.39
TN−CDC−QDX28318571_2021−08−28_AY.39
TN−CDC−4281352−001_2021−08−31_AY.39
TN−CDC−QDX30169984_2021−10−18_AY.39
TN−CDC−ASC210143566_2021−09−11_AY.39
TN−CDC−ASC210657098_2021−11−30_AY.39
TN−CDC−ASC210608498_2021−12−09_AY.39
TN−CDC−ASC210657095_2021−11−30_AY.39
TN−ASC−210507350_2022−01−04_AY.39
TN−SPHL−0169_2021−07−13_AY.39
TN−ASC−210644489_2021−09−05_AY.39
TN−ASC−210644335_2021−08−20_AY.39
TN−ASC−210644600_2021−08−14_AY.39
TN−CDC−QDX28508404_2021−09−05_AY.39
TN−CDC−ASC210278812_2021−09−16_AY.39
TN−CDC−STM−000711668_2021−07−26_AY.39
TN−CDC−QDX27788016_2021−08−14_AY.39
OL828800_SPHL−0984_2021−10−12_AY.39
TN−CDC−LC0104065_2021−07−13_AY.39
OM921643_TN−VUMC−001891_2021−07−19_AY.39
TN−ASC−210645823_2021−09−08_AY.39
TN−ASC−210645242_2021−09−20_AY.39
TN−CDC−QDX26826168_2021−07−13_AY.39
TN−CDC−LC0104070_2021−07−10_AY.39
TN−CDC−QDX26763476_2021−07−09_AY.39
TN−CDC−QDX26763381_2021−07−07_AY.39
TN−ASC−210644449_2021−08−26_AY.39
TN−ASC−210644088_2021−08−14_AY.39
OM921652_TN−VUMC−001912_2021−07−21_AY.39
TN−CDC−LC0242067_2021−08−31_AY.39
TN−ASC−210644940_2021−08−31_AY.39
TN−CDC−ASC210406205_2021−09−14_AY.39
TN−CDC−ASC210131675_2021−09−07_AY.39
TN−CDC−ASC210179208_2021−08−31_AY.39
TN−CDC−ASC210134214_2021−09−07_AY.39
TN−CDC−ASC210089102_2021−09−21_AY.39
TN−CDC−ASC210179214_2021−08−31_AY.39
TN−CDC−ASC210127976_2021−09−04_AY.39
TN−CDC−ASC210118546_2021−08−17_AY.39
TN−CDC−ASC210210788_2021−08−03_AY.39
OL946815_TN−CDC−ASC210388852_2021−11−04_AY.39
OL730483_TN−CDC−ASC210388853_2021−11−04_AY.39
OL730484_TN−CDC−ASC210388854_2021−11−04_AY.39
TN−CDC−ASC210113683_2021−07−11_AY.39
TN−CDC−ASC210156049_2021−07−14_AY.39
MN908947 (Wuhan−Hu−1/2019)
0.2

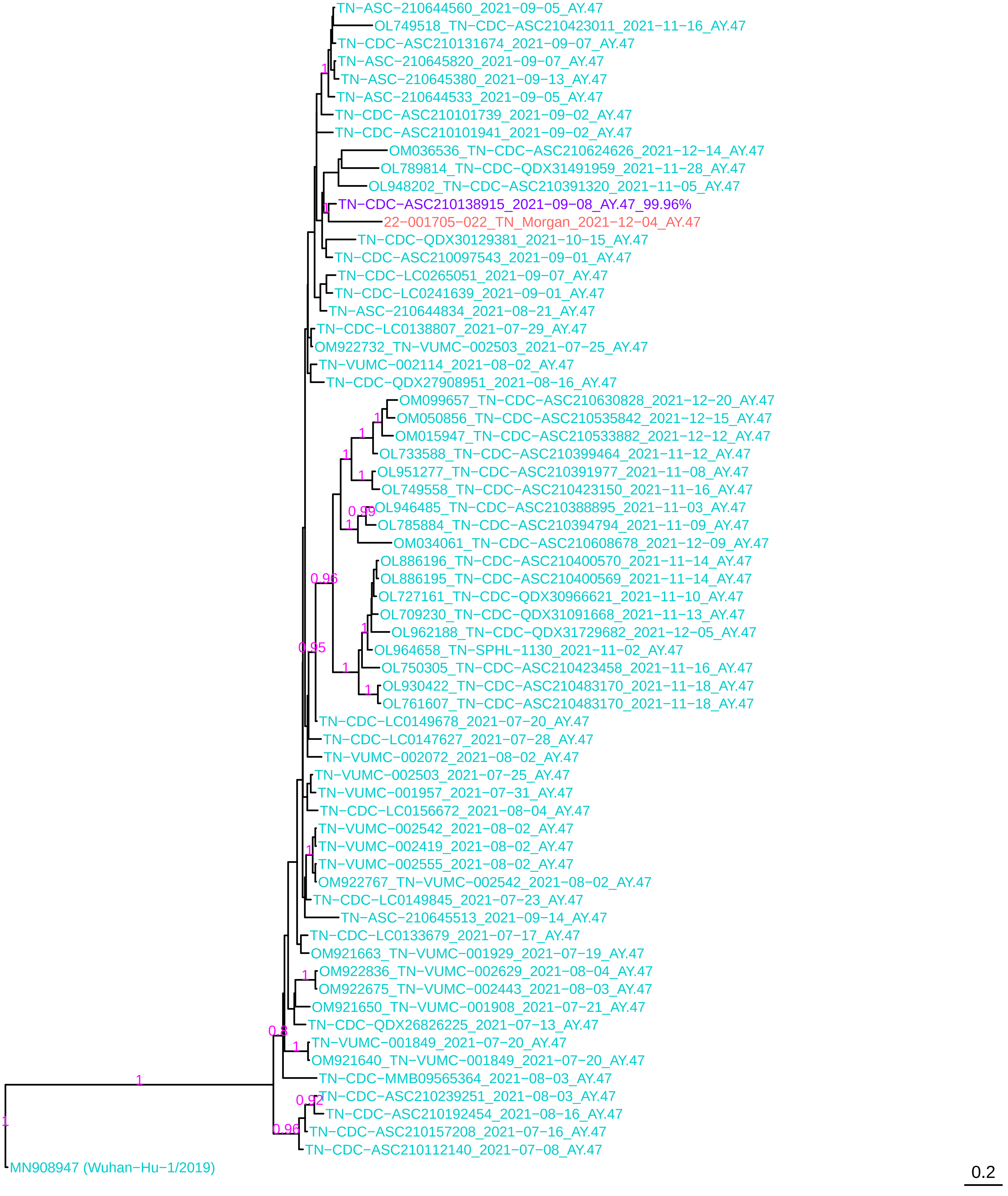
TN−ASC−210644560_2021−09−05_AY.47
OL749518_TN−CDC−ASC210423011_2021−11−16_AY.47
TN−CDC−ASC210131674_2021−09−07_AY.47
TN−ASC−210645820_2021−09−07_AY.47
TN−ASC−210645380_2021−09−13_AY.47
TN−ASC−210644533_2021−09−05_AY.47
TN−CDC−ASC210101739_2021−09−02_AY.47
TN−CDC−ASC210101941_2021−09−02_AY.47
OM036536_TN−CDC−ASC210624626_2021−12−14_AY.47
OL789814_TN−CDC−QDX31491959_2021−11−28_AY.47
OL948202_TN−CDC−ASC210391320_2021−11−05_AY.47
TN−CDC−ASC210138915_2021−09−08_AY.47_99.96%
22−001705−022_TN_Morgan_2021−12−04_AY.47
TN−CDC−QDX30129381_2021−10−15_AY.47
TN−CDC−ASC210097543_2021−09−01_AY.47
TN−CDC−LC0265051_2021−09−07_AY.47
TN−CDC−LC0241639_2021−09−01_AY.47
TN−ASC−210644834_2021−08−21_AY.47
TN−CDC−LC0138807_2021−07−29_AY.47
OM922732_TN−VUMC−002503_2021−07−25_AY.47
TN−VUMC−002114_2021−08−02_AY.47
TN−CDC−QDX27908951_2021−08−16_AY.47
OM099657_TN−CDC−ASC210630828_2021−12−20_AY.47
OM050856_TN−CDC−ASC210535842_2021−12−15_AY.47
OM015947_TN−CDC−ASC210533882_2021−12−12_AY.47
OL733588_TN−CDC−ASC210399464_2021−11−12_AY.47
OL951277_TN−CDC−ASC210391977_2021−11−08_AY.47
OL749558_TN−CDC−ASC210423150_2021−11−16_AY.47
OL946485_TN−CDC−ASC210388895_2021−11−03_AY.47
OL785884_TN−CDC−ASC210394794_2021−11−09_AY.47
OM034061_TN−CDC−ASC210608678_2021−12−09_AY.47
OL886196_TN−CDC−ASC210400570_2021−11−14_AY.47
OL886195_TN−CDC−ASC210400569_2021−11−14_AY.47
OL727161_TN−CDC−QDX30966621_2021−11−10_AY.47
OL709230_TN−CDC−QDX31091668_2021−11−13_AY.47
OL962188_TN−CDC−QDX31729682_2021−12−05_AY.47
OL964658_TN−SPHL−1130_2021−11−02_AY.47
OL750305_TN−CDC−ASC210423458_2021−11−16_AY.47
OL930422_TN−CDC−ASC210483170_2021−11−18_AY.47
OL761607_TN−CDC−ASC210483170_2021−11−18_AY.47
TN−CDC−LC0149678_2021−07−20_AY.47
TN−CDC−LC0147627_2021−07−28_AY.47
TN−VUMC−002072_2021−08−02_AY.47
TN−VUMC−002503_2021−07−25_AY.47
TN−VUMC−001957_2021−07−31_AY.47
TN−CDC−LC0156672_2021−08−04_AY.47
TN−VUMC−002542_2021−08−02_AY.47
TN−VUMC−002419_2021−08−02_AY.47
TN−VUMC−002555_2021−08−02_AY.47
OM922767_TN−VUMC−002542_2021−08−02_AY.47
TN−CDC−LC0149845_2021−07−23_AY.47
TN−ASC−210645513_2021−09−14_AY.47
TN−CDC−LC0133679_2021−07−17_AY.47
OM921663_TN−VUMC−001929_2021−07−19_AY.47
OM922836_TN−VUMC−002629_2021−08−04_AY.47
OM922675_TN−VUMC−002443_2021−08−03_AY.47
OM921650_TN−VUMC−001908_2021−07−21_AY.47
TN−CDC−QDX26826225_2021−07−13_AY.47
TN−VUMC−001849_2021−07−20_AY.47
OM921640_TN−VUMC−001849_2021−07−20_AY.47
TN−CDC−MMB09565364_2021−08−03_AY.47
TN−CDC−ASC210239251_2021−08−03_AY.47
TN−CDC−ASC210192454_2021−08−16_AY.47
TN−CDC−ASC210157208_2021−07−16_AY.47
TN−CDC−ASC210112140_2021−07−08_AY.47
MN908947 (Wuhan−Hu−1/2019)
0.2

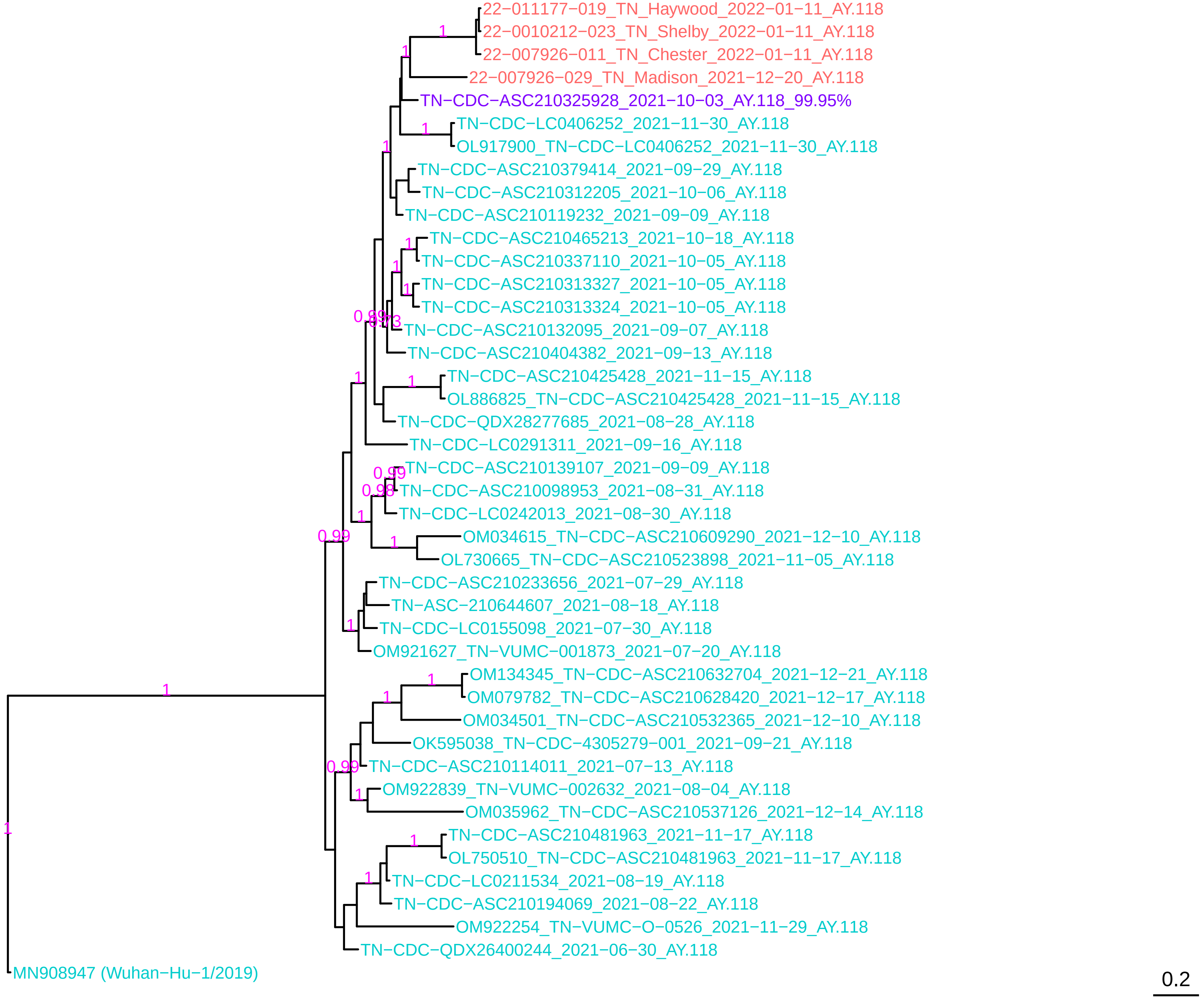

22−011177−019_TN_Haywood_2022−01−11_AY.118
22−0010212−023_TN_Shelby_2022−01−11_AY.118
22−007926−011_TN_Chester_2022−01−11_AY.118
22−007926−029_TN_Madison_2021−12−20_AY.118
TN−CDC−ASC210325928_2021−10−03_AY.118_99.95%
TN−CDC−LC0406252_2021−11−30_AY.118
OL917900_TN−CDC−LC0406252_2021−11−30_AY.118
TN−CDC−ASC210379414_2021−09−29_AY.118
TN−CDC−ASC210312205_2021−10−06_AY.118
TN−CDC−ASC210119232_2021−09−09_AY.118
TN−CDC−ASC210465213_2021−10−18_AY.118
TN−CDC−ASC210337110_2021−10−05_AY.118
TN−CDC−ASC210313327_2021−10−05_AY.118
TN−CDC−ASC210313324_2021−10−05_AY.118
TN−CDC−ASC210132095_2021−09−07_AY.118
TN−CDC−ASC210404382_2021−09−13_AY.118
TN−CDC−ASC210425428_2021−11−15_AY.118
OL886825_TN−CDC−ASC210425428_2021−11−15_AY.118
TN−CDC−QDX28277685_2021−08−28_AY.118
TN−CDC−LC0291311_2021−09−16_AY.118
TN−CDC−ASC210139107_2021−09−09_AY.118
TN−CDC−ASC210098953_2021−08−31_AY.118
TN−CDC−LC0242013_2021−08−30_AY.118
OM034615_TN−CDC−ASC210609290_2021−12−10_AY.118
OL730665_TN−CDC−ASC210523898_2021−11−05_AY.118
TN−CDC−ASC210233656_2021−07−29_AY.118
TN−ASC−210644607_2021−08−18_AY.118
TN−CDC−LC0155098_2021−07−30_AY.118
OM921627_TN−VUMC−001873_2021−07−20_AY.118
OM134345_TN−CDC−ASC210632704_2021−12−21_AY.118
OM079782_TN−CDC−ASC210628420_2021−12−17_AY.118
OM034501_TN−CDC−ASC210532365_2021−12−10_AY.118
OK595038_TN−CDC−4305279−001_2021−09−21_AY.118
TN−CDC−ASC210114011_2021−07−13_AY.118
OM922839_TN−VUMC−002632_2021−08−04_AY.118
OM035962_TN−CDC−ASC210537126_2021−12−14_AY.118
TN−CDC−ASC210481963_2021−11−17_AY.118
OL750510_TN−CDC−ASC210481963_2021−11−17_AY.118
TN−CDC−LC0211534_2021−08−19_AY.118
TN−CDC−ASC210194069_2021−08−22_AY.118
OM922254_TN−VUMC−O−0526_2021−11−29_AY.118
TN−CDC−QDX26400244_2021−06−30_AY.118
MN908947 (Wuhan−Hu−1/2019)
0.2

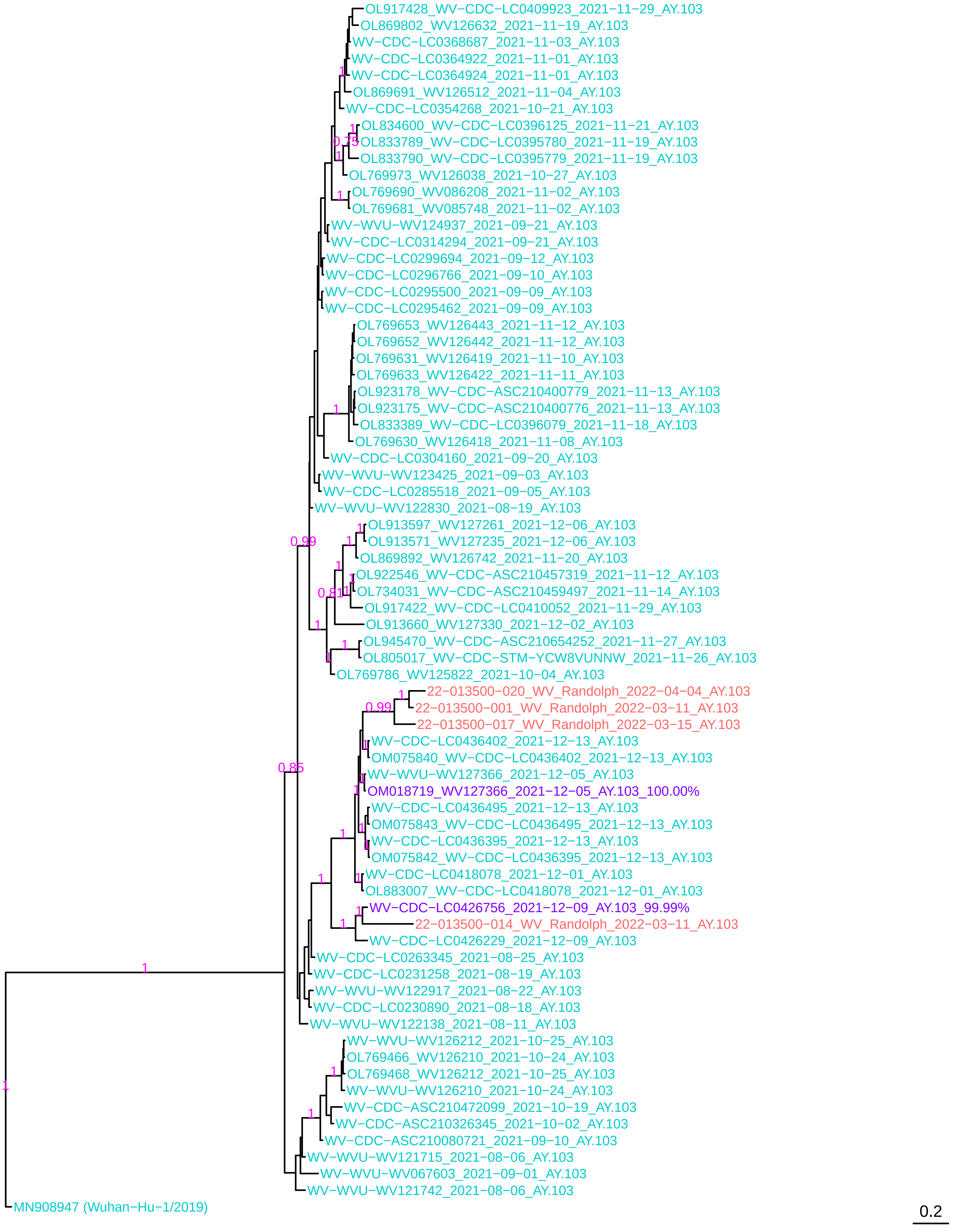
OL917428_WV−CDC−LC0409923_2021−11−29_AY.103
OL869802_WV126632_2021−11−19_AY.103
WV−CDC−LC0368687_2021−11−03_AY.103
WV−CDC−LC0364922_2021−11−01_AY.103
WV−CDC−LC0364924_2021−11−01_AY.103
OL869691_WV126512_2021−11−04_AY.103
WV−CDC−LC0354268_2021−10−21_AY.103
OL834600_WV−CDC−LC0396125_2021−11−21_AY.103
OL833789_WV−CDC−LC0395780_2021−11−19_AY.103
OL833790_WV−CDC−LC0395779_2021−11−19_AY.103
OL769973_WV126038_2021−10−27_AY.103
OL769690_WV086208_2021−11−02_AY.103
OL769681_WV085748_2021−11−02_AY.103
WV−WVU−WV124937_2021−09−21_AY.103
WV−CDC−LC0314294_2021−09−21_AY.103
WV−CDC−LC0299694_2021−09−12_AY.103
WV−CDC−LC0296766_2021−09−10_AY.103
WV−CDC−LC0295500_2021−09−09_AY.103
WV−CDC−LC0295462_2021−09−09_AY.103
OL769653_WV126443_2021−11−12_AY.103
OL769652_WV126442_2021−11−12_AY.103
OL769631_WV126419_2021−11−10_AY.103
OL769633_WV126422_2021−11−11_AY.103
OL923178_WV−CDC−ASC210400779_2021−11−13_AY.103
OL923175_WV−CDC−ASC210400776_2021−11−13_AY.103
OL833389_WV−CDC−LC0396079_2021−11−18_AY.103
OL769630_WV126418_2021−11−08_AY.103
WV−CDC−LC0304160_2021−09−20_AY.103
WV−WVU−WV123425_2021−09−03_AY.103
WV−CDC−LC0285518_2021−09−05_AY.103
WV−WVU−WV122830_2021−08−19_AY.103
OL913597_WV127261_2021−12−06_AY.103
OL913571_WV127235_2021−12−06_AY.103
OL869892_WV126742_2021−11−20_AY.103
OL922546_WV−CDC−ASC210457319_2021−11−12_AY.103
OL734031_WV−CDC−ASC210459497_2021−11−14_AY.103
OL917422_WV−CDC−LC0410052_2021−11−29_AY.103
OL913660_WV127330_2021−12−02_AY.103
OL945470_WV−CDC−ASC210654252_2021−11−27_AY.103
OL805017_WV−CDC−STM−YCW8VUNNW_2021−11−26_AY.103
OL769786_WV125822_2021−10−04_AY.103
22−013500−020_WV_Randolph_2022−04−04_AY.103
22−013500−001_WV_Randolph_2022−03−11_AY.103
22−013500−017_WV_Randolph_2022−03−15_AY.103
WV−CDC−LC0436402_2021−12−13_AY.103
OM075840_WV−CDC−LC0436402_2021−12−13_AY.103
WV−WVU−WV127366_2021−12−05_AY.103
OM018719_WV127366_2021−12−05_AY.103_100.00%
WV−CDC−LC0436495_2021−12−13_AY.103
OM075843_WV−CDC−LC0436495_2021−12−13_AY.103
WV−CDC−LC0436395_2021−12−13_AY.103
OM075842_WV−CDC−LC0436395_2021−12−13_AY.103
WV−CDC−LC0418078_2021−12−01_AY.103
OL883007_WV−CDC−LC0418078_2021−12−01_AY.103
WV−CDC−LC0426756_2021−12−09_AY.103_99.99%
22−013500−014_WV_Randolph_2022−03−11_AY.103
WV−CDC−LC0426229_2021−12−09_AY.103
WV−CDC−LC0263345_2021−08−25_AY.103
WV−CDC−LC0231258_2021−08−19_AY.103
WV−WVU−WV122917_2021−08−22_AY.103
WV−CDC−LC0230890_2021−08−18_AY.103
WV−WVU−WV122138_2021−08−11_AY.103
WV−WVU−WV126212_2021−10−25_AY.103
OL769466_WV126210_2021−10−24_AY.103
OL769468_WV126212_2021−10−25_AY.103
WV−WVU−WV126210_2021−10−24_AY.103
WV−CDC−ASC210472099_2021−10−19_AY.103
WV−CDC−ASC210326345_2021−10−02_AY.103
WV−CDC−ASC210080721_2021−09−10_AY.103
WV−WVU−WV121715_2021−08−06_AY.103
WV−WVU−WV067603_2021−09−01_AY.103
WV−WVU−WV121742_2021−08−06_AY.103
MN908947 (Wuhan−Hu−1/2019)
0.2

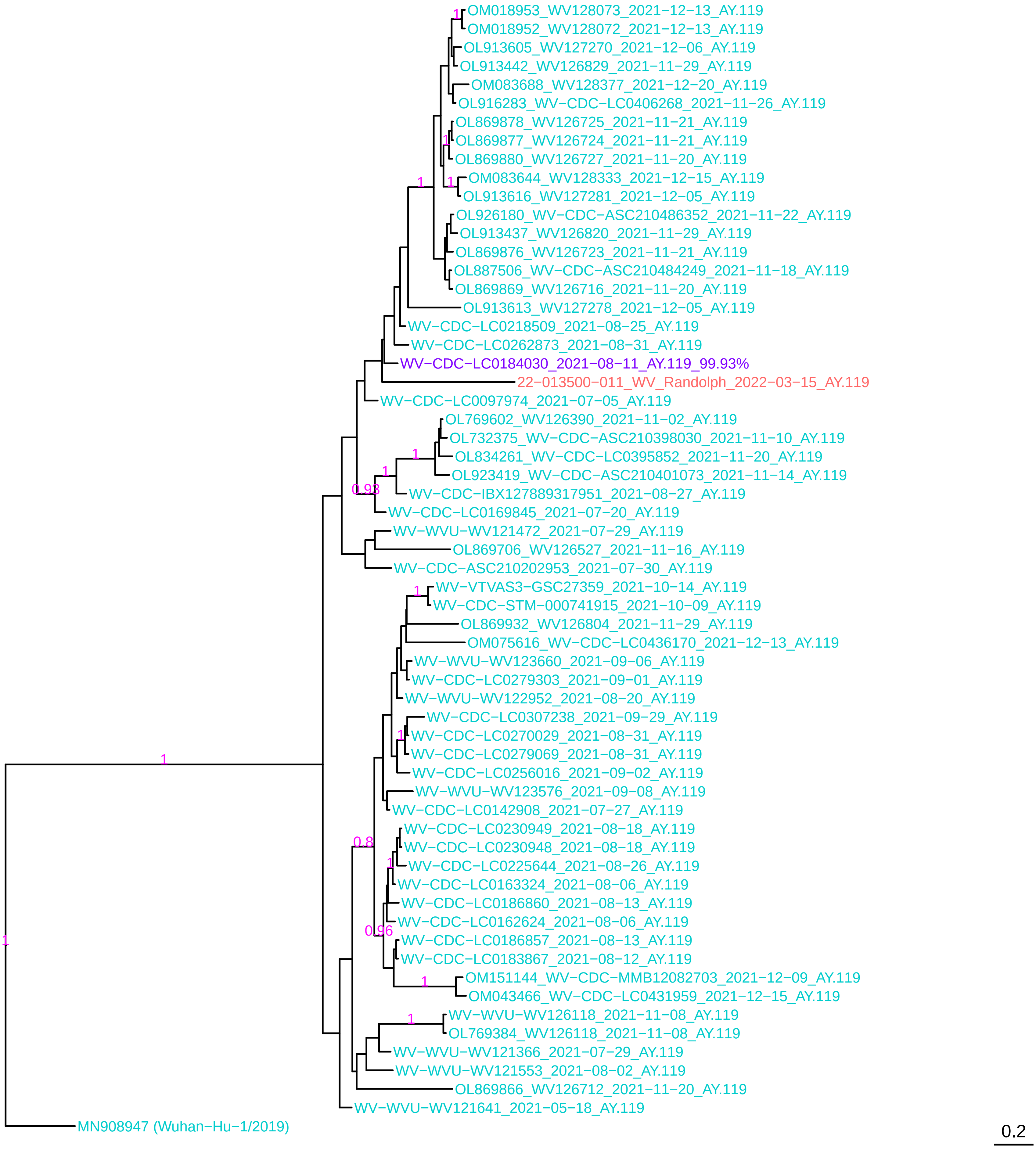
OM018953_WV128073_2021-12-13_AY.119
OM018952_WV128072_2021-12-13_AY.119
OL913605_WV127270_2021-12-06_AY.119
OL913442_WV126829_2021-11-29_AY.119
OM083688_WV128377_2021-12-20_AY.119
OL916283_WV-CDC-LC0406268_2021-11-26_AY.119
OL869878_WV126725_2021-11-21_AY.119
OL869877_WV126724_2021-11-21_AY.119
OL869880_WV126727_2021-11-20_AY.119
OM083644_WV128333_2021-12-15_AY.119
OL913616_WV127281_2021-12-05_AY.119
OL926180_WV-CDC-ASC210486352_2021-11-22_AY.119
OL913437_WV126820_2021-11-29_AY.119
OL869876_WV126723_2021-11-21_AY.119
OL887506_WV-CDC-ASC210484249_2021-11-18_AY.119
OL869869_WV126716_2021-11-20_AY.119
OL913613_WV127278_2021-12-05_AY.119
WV-CDC-LC0218509_2021-08-25_AY.119
WV-CDC-LC0262873_2021-08-31_AY.119
WV-CDC-LC0184030_2021-08-11_AY.119_99.93%
22-013500-011_WV_Randolph_2022-03-15_AY.119
WV-CDC-LC0097974_2021-07-05_AY.119
OL769602_WV126390_2021-11-02_AY.119
OL732375_WV-CDC-ASC210398030_2021-11-10_AY.119
OL834261_WV-CDC-LC0395852_2021-11-20_AY.119
OL923419_WV-CDC-ASC210401073_2021-11-14_AY.119
WV-CDC-IBX127889317951_2021-08-27_AY.119
WV-CDC-LC0169845_2021-07-20_AY.119
WV-WVU-WV121472_2021-07-29_AY.119
OL869706_WV126527_2021-11-16_AY.119
WV-CDC-ASC210202953_2021-07-30_AY.119
WV-VTVAS3-GSC27359_2021-10-14_AY.119
WV-CDC-STM-000741915_2021-10-09_AY.119
OL869932_WV126804_2021-11-29_AY.119
OM075616_WV-CDC-LC0436170_2021-12-13_AY.119
WV-WVU-WV123660_2021-09-06_AY.119
WV-CDC-LC0279303_2021-09-01_AY.119
WV-WVU-WV122952_2021-08-20_AY.119
WV-CDC-LC0307238_2021-09-29_AY.119
WV-CDC-LC0270029_2021-08-31_AY.119
WV-CDC-LC0279069_2021-08-31_AY.119
WV-CDC-LC0256016_2021-09-02_AY.119
WV-WVU-WV123576_2021-09-08_AY.119
WV-CDC-LC0142908_2021-07-27_AY.119
WV-CDC-LC0230949_2021-08-18_AY.119
WV-CDC-LC0230948_2021-08-18_AY.119
WV-CDC-LC0225644_2021-08-26_AY.119
WV-CDC-LC0163324_2021-08-06_AY.119
WV-CDC-LC0186860_2021-08-13_AY.119
WV-CDC-LC0162624_2021-08-06_AY.119
WV-CDC-LC0186857_2021-08-13_AY.119
WV-CDC-LC0183867_2021-08-12_AY.119
OM151144_WV-CDC-MMB12082703_2021-12-09_AY.119
OM043466_WV-CDC-LC0431959_2021-12-15_AY.119
WV-WVU-WV126118_2021-11-08_AY.119
OL769384_WV126118_2021-11-08_AY.119
WV-WVU-WV121366_2021-07-29_AY.119
WV-WVU-WV121553_2021-08-02_AY.119
OL869866_WV126712_2021-11-20_AY.119
WV-WVU-WV121641_2021-05-18_AY.119
MN908947 (Wuhan-Hu-1/2019)
0.93
0.8
0.96
0.2

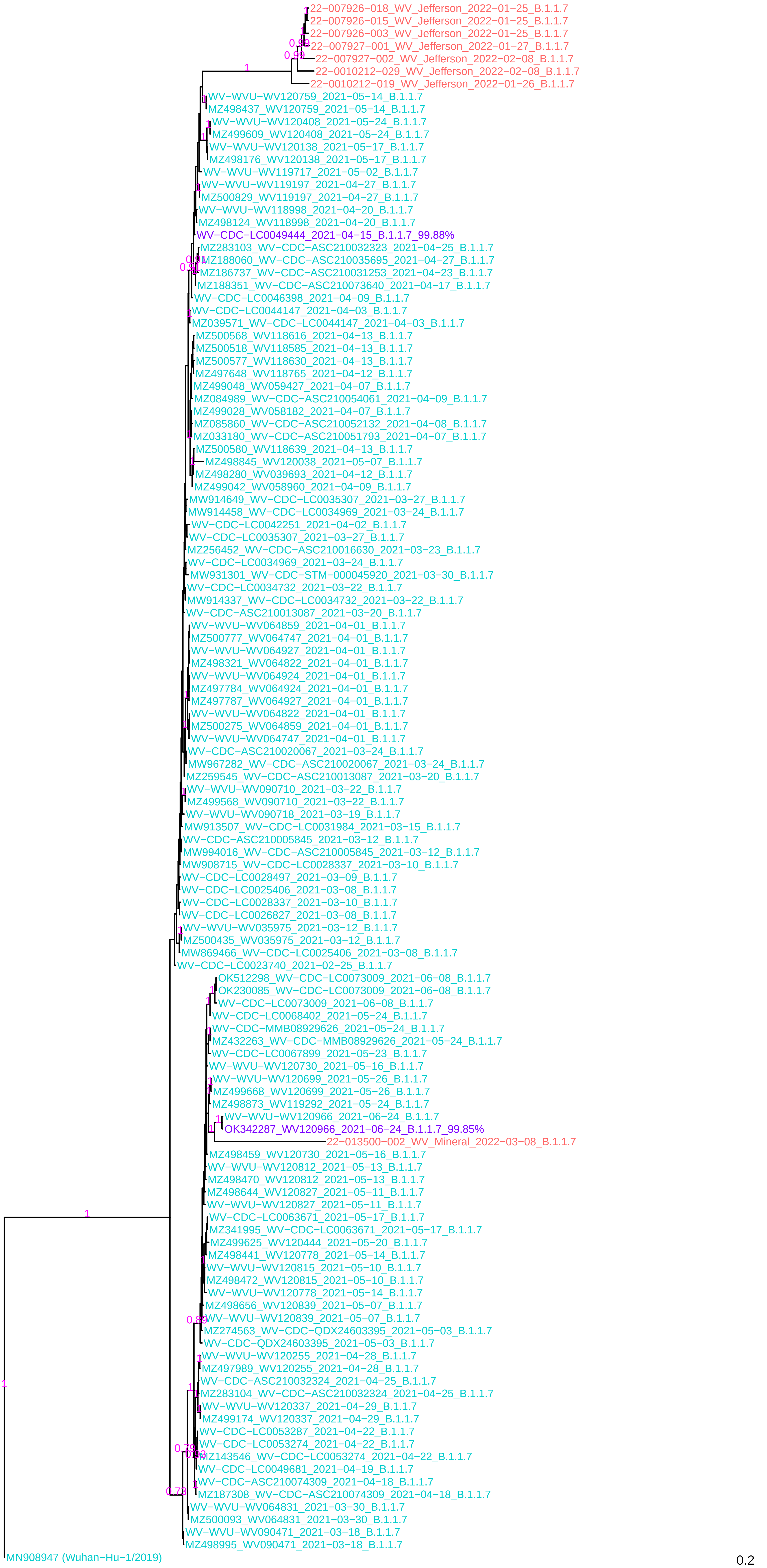
22−007926−018_WV_Jefferson_2022−01−25_B.1.1.7
22−007926−015_WV_Jefferson_2022−01−25_B.1.1.7
22−007926−003_WV_Jefferson_2022−01−25_B.1.1.7
22−007927−001_WV_Jefferson_2022−01−27_B.1.1.7
22−007927−002_WV_Jefferson_2022−02−08_B.1.1.7
22−0010212−029_WV_Jefferson_2022−02−08_B.1.1.7
22−0010212−019_WV_Jefferson_2022−01−26_B.1.1.7
WV−WVU−WV120759_2021−05−14_B.1.1.7
MZ498437_WV120759_2021−05−14_B.1.1.7
WV−WVU−WV120408_2021−05−24_B.1.1.7
MZ499609_WV120408_2021−05−24_B.1.1.7
WV−WVU−WV120138_2021−05−17_B.1.1.7
MZ498176_WV120138_2021−05−17_B.1.1.7
WV−WVU−WV119717_2021−05−02_B.1.1.7
WV−WVU−WV119197_2021−04−27_B.1.1.7
MZ500829_WV119197_2021−04−27_B.1.1.7
WV−WVU−WV118998_2021−04−20_B.1.1.7
MZ498124_WV118998_2021−04−20_B.1.1.7
WV−CDC−LC0049444_2021−04−15_B.1.1.7_99.88%
MZ283103_WV−CDC−ASC210032323_2021−04−25_B.1.1.7
MZ188060_WV−CDC−ASC210035695_2021−04−27_B.1.1.7
MZ186737_WV−CDC−ASC210031253_2021−04−23_B.1.1.7
MZ188351_WV−CDC−ASC210073640_2021−04−17_B.1.1.7
WV−CDC−LC0046398_2021−04−09_B.1.1.7
WV−CDC−LC0044147_2021−04−03_B.1.1.7
MZ039571_WV−CDC−LC0044147_2021−04−03_B.1.1.7
MZ500568_WV118616_2021−04−13_B.1.1.7
MZ500518_WV118585_2021−04−13_B.1.1.7
MZ500577_WV118630_2021−04−13_B.1.1.7
MZ497648_WV118765_2021−04−12_B.1.1.7
MZ499048_WV059427_2021−04−07_B.1.1.7
MZ084989_WV−CDC−ASC210054061_2021−04−09_B.1.1.7
MZ499028_WV058182_2021−04−07_B.1.1.7
MZ085860_WV−CDC−ASC210052132_2021−04−08_B.1.1.7
MZ033180_WV−CDC−ASC210051793_2021−04−07_B.1.1.7
MZ500580_WV118639_2021−04−13_B.1.1.7
MZ498845_WV120038_2021−05−07_B.1.1.7
MZ498280_WV039693_2021−04−12_B.1.1.7
MZ499042_WV058960_2021−04−09_B.1.1.7
MW914649_WV−CDC−LC0035307_2021−03−27_B.1.1.7
MW914458_WV−CDC−LC0034969_2021−03−24_B.1.1.7
WV−CDC−LC0042251_2021−04−02_B.1.1.7
WV−CDC−LC0035307_2021−03−27_B.1.1.7
MZ256452_WV−CDC−ASC210016630_2021−03−23_B.1.1.7
WV−CDC−LC0034969_2021−03−24_B.1.1.7
MW931301_WV−CDC−STM−000045920_2021−03−30_B.1.1.7
WV−CDC−LC0034732_2021−03−22_B.1.1.7
MW914337_WV−CDC−LC0034732_2021−03−22_B.1.1.7
WV−CDC−ASC210013087_2021−03−20_B.1.1.7
WV−WVU−WV064859_2021−04−01_B.1.1.7
MZ500777_WV064747_2021−04−01_B.1.1.7
WV−WVU−WV064927_2021−04−01_B.1.1.7
MZ498321_WV064822_2021−04−01_B.1.1.7
WV−WVU−WV064924_2021−04−01_B.1.1.7
MZ497784_WV064924_2021−04−01_B.1.1.7
MZ497787_WV064927_2021−04−01_B.1.1.7
WV−WVU−WV064822_2021−04−01_B.1.1.7
MZ500275_WV064859_2021−04−01_B.1.1.7
WV−WVU−WV064747_2021−04−01_B.1.1.7
WV−CDC−ASC210020067_2021−03−24_B.1.1.7
MW967282_WV−CDC−ASC210020067_2021−03−24_B.1.1.7
MZ259545_WV−CDC−ASC210013087_2021−03−20_B.1.1.7
WV−WVU−WV090710_2021−03−22_B.1.1.7
MZ499568_WV090710_2021−03−22_B.1.1.7
WV−WVU−WV090718_2021−03−19_B.1.1.7
MW913507_WV−CDC−LC0031984_2021−03−15_B.1.1.7
WV−CDC−ASC210005845_2021−03−12_B.1.1.7
MW994016_WV−CDC−ASC210005845_2021−03−12_B.1.1.7
MW908715_WV−CDC−LC0028337_2021−03−10_B.1.1.7
WV−CDC−LC0028497_2021−03−09_B.1.1.7
WV−CDC−LC0025406_2021−03−08_B.1.1.7
WV−CDC−LC0028337_2021−03−10_B.1.1.7
WV−CDC−LC0026827_2021−03−08_B.1.1.7
WV−WVU−WV035975_2021−03−12_B.1.1.7
MZ500435_WV035975_2021−03−12_B.1.1.7
MW869466_WV−CDC−LC0025406_2021−03−08_B.1.1.7
WV−CDC−LC0023740_2021−02−25_B.1.1.7
OK512298_WV−CDC−LC0073009_2021−06−08_B.1.1.7
OK230085_WV−CDC−LC0073009_2021−06−08_B.1.1.7
WV−CDC−LC0073009_2021−06−08_B.1.1.7
WV−CDC−LC0068402_2021−05−24_B.1.1.7
WV−CDC−MMB08929626_2021−05−24_B.1.1.7
MZ432263_WV−CDC−MMB08929626_2021−05−24_B.1.1.7
WV−CDC−LC0067899_2021−05−23_B.1.1.7
WV−WVU−WV120730_2021−05−16_B.1.1.7
WV−WVU−WV120699_2021−05−26_B.1.1.7
MZ499668_WV120699_2021−05−26_B.1.1.7
MZ498873_WV119292_2021−05−24_B.1.1.7
WV−WVU−WV120966_2021−06−24_B.1.1.7
OK342287_WV120966_2021−06−24_B.1.1.7_99.85%
22−013500−002_WV_Mineral_2022−03−08_B.1.1.7
MZ498459_WV120730_2021−05−16_B.1.1.7
WV−WVU−WV120812_2021−05−13_B.1.1.7
MZ498470_WV120812_2021−05−13_B.1.1.7
MZ498644_WV120827_2021−05−11_B.1.1.7
WV−WVU−WV120827_2021−05−11_B.1.1.7
WV−CDC−LC0063671_2021−05−17_B.1.1.7
MZ341995_WV−CDC−LC0063671_2021−05−17_B.1.1.7
MZ499625_WV120444_2021−05−20_B.1.1.7
MZ498441_WV120778_2021−05−14_B.1.1.7
WV−WVU−WV120815_2021−05−10_B.1.1.7
MZ498472_WV120815_2021−05−10_B.1.1.7
WV−WVU−WV120778_2021−05−14_B.1.1.7
MZ498656_WV120839_2021−05−07_B.1.1.7
WV−WVU−WV120839_2021−05−07_B.1.1.7
MZ274563_WV−CDC−QDX24603395_2021−05−03_B.1.1.7
WV−CDC−QDX24603395_2021−05−03_B.1.1.7
WV−WVU−WV120255_2021−04−28_B.1.1.7
MZ497989_WV120255_2021−04−28_B.1.1.7
WV−CDC−ASC210032324_2021−04−25_B.1.1.7
MZ283104_WV−CDC−ASC210032324_2021−04−25_B.1.1.7
WV−WVU−WV120337_2021−04−29_B.1.1.7
MZ499174_WV120337_2021−04−29_B.1.1.7
WV−CDC−LC0053287_2021−04−22_B.1.1.7
WV−CDC−LC0053274_2021−04−22_B.1.1.7
MZ143546_WV−CDC−LC0053274_2021−04−22_B.1.1.7
WV−CDC−LC0049681_2021−04−19_B.1.1.7
WV−CDC−ASC210074309_2021−04−18_B.1.1.7
MZ187308_WV−CDC−ASC210074309_2021−04−18_B.1.1.7
WV−WVU−WV064831_2021−03−30_B.1.1.7
MZ500093_WV064831_2021−03−30_B.1.1.7
WV−WVU−WV090471_2021−03−18_B.1.1.7
MZ498995_WV090471_2021−03−18_B.1.1.7
MN908947 (Wuhan−Hu−1/2019)
0.2
