# Supplementary material for: Transmission of SARS-CoV-2 in free-ranging white-tailed deer in the United States
Source: Nat Commun. 2023 Jul 10;14:4078. doi: 10.1038/s41467-023-39782-x (PMC10333304; doi:10.1038/s41467-023-39782-x)

Supplementary Data 12. Phylogenetic analyses of the white-tailed deer SARS-CoV-2 sequences (n = 282) and their potential precursor viruses in humans. Because of low coverage at the 5' untranslated region (before position 266) and 3' untranslated region (after position 29,674) of the genome, we excluded these positions from nucleotide and amino acid substitution analyses. In addition, 265 problematic positions summarized at https://github.com/W-L/ProblematicSites_SARS-CoV2 were marked before phylogenetic analyses. The estimates of divergence time were obtained by calculating the median node height of the 95% highest posterior density (HPD) interval from a maximum clade credibility tree generated using BEAST. The node bars, depicted in light blue, represent the 95%HPD interval for each node. The timescale of the phylogenetic tree was represented inunits of years, and the scale bar indicates the divergence time in years.

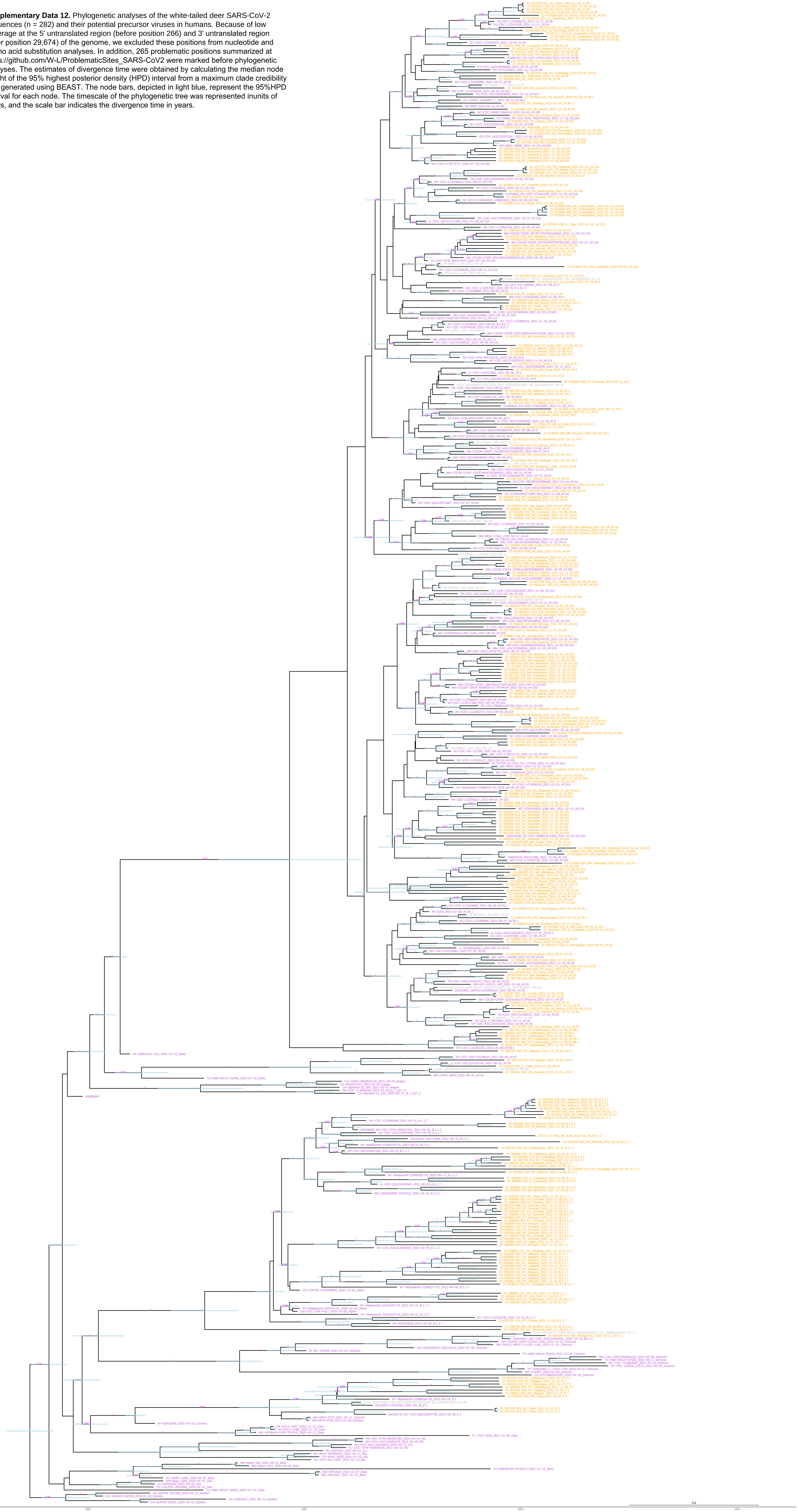

Supplement: Supplementary file 5 — Supplementary Data 10–12 [file 41467_2023_39782_MOESM5_ESM.zip › Supplementary Data 10-12/Supplementary Data 12.pdf]
