# Supplementary figures and images for: Transmission of SARS-CoV-2 in free-ranging white-tailed deer in the United States
Source: Nat Commun. 2023 Jul 10;14:4078. doi: 10.1038/s41467-023-39782-x (PMC10333304; doi:10.1038/s41467-023-39782-x)

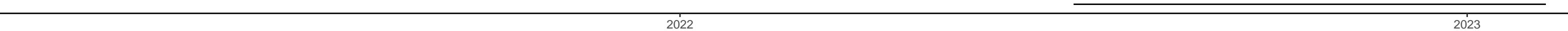

Supplement: Supplementary file 5 — Supplementary Data 10–12 [file 41467_2023_39782_MOESM5_ESM.zip › Supplementary Data 10-12/Supplementary Data 11.pdf]
