# Supplementary material for: Transmission of SARS-CoV-2 in free-ranging white-tailed deer in the United States
Source: Nat Commun. 2023 Jul 10;14:4078. doi: 10.1038/s41467-023-39782-x (PMC10333304; doi:10.1038/s41467-023-39782-x)
Supplement: Supplementary file 6 — Reporting Summary [file 41467_2023_39782_MOESM6_ESM.pdf]

nature portfolio

Corresponding author(s): Thomas J. DeLiberto and Xiu-Feng Wan

Last updated by author(s): Jun 6, 2023

Reporting Summary

Nature Portfolio wishes to improve the reproducibility of the work that we publish. This form provides structure for consistency and transparency in reporting. For further information on Nature Portfolio policies, see our Editorial Policies and the Editorial Policy Checklist.

Statistics

For all statistical analyses, confirm that the following items are present in the figure legend, table legend, main text, or Methods section.

n/a	Confirmed	
☐	☒	The exact sample size (n) for each experimental group/condition, given as a discrete number and unit of measurement
☐	☒	A statement on whether measurements were taken from distinct samples or whether the same sample was measured repeatedly
☒	☐	The statistical test(s) used AND whether they are one- or two-sided<br>*Only common tests should be described solely by name; describe more complex techniques in the Methods section.*
☒	☐	A description of all covariates tested
☒	☐	A description of any assumptions or corrections, such as tests of normality and adjustment for multiple comparisons
☐	☒	A full description of the statistical parameters including central tendency (e.g. means) or other basic estimates (e.g. regression coefficient) AND variation (e.g. standard deviation) or associated estimates of uncertainty (e.g. confidence intervals)
☒	☐	For null hypothesis testing, the test statistic (e.g. F, t, r) with confidence intervals, effect sizes, degrees of freedom and P value noted<br>*Give P values as exact values whenever suitable.*
☐	☒	For Bayesian analysis, information on the choice of priors and Markov chain Monte Carlo settings
☒	☐	For hierarchical and complex designs, identification of the appropriate level for tests and full reporting of outcomes
☒	☐	Estimates of effect sizes (e.g. Cohen's d, Pearson's r), indicating how they were calculated

Our web collection on statistics for biologists contains articles on many of the points above.

Software and code

Policy information about availability of computer code

Data collection	Iterative Refinement Meta-Assembler (IRMA) v1.0.2 used for sequence assembly. Qiagen CLC Genomics Workbench 20.0.4 used for consensus sequence validation
Data analysis	NextStrain v4.1.0 used to identify the nucleotide and amino acid substitutions. Outbreak.info R package v0.2.0 were used to obtain mutation frequency in all SARS-CoV-2 human viruses from the US and worldwide. PANGOLIN v4.0.6 used to determine PANGO lineages. Bayesian phylogenetic analysis by sampling trees (BEAST) v.1.10.4 used for Bayesian phylogenetic analyses. Babette R package v2.3.2 was used for batch BEAST processing. Tracer v1.7.2 was used to assess the BEAST results. The maximum clade credibility (MCC) tree was summarized by using TreeAnnotator v.1.10.4. Ggtree R package V3.8.0 was used for tree visualization. FastTree v1.4.4, Complete Composition Vector (CCV) program v1.0, and Ultrafast Sample placement on Existing tRees (UShER) used to select the closest sequences. Biopython v1.79 package59 were used for sequences data processing. HyPhy v2.5.42(MP) used for positive and negative selection. WTD sequences were aligned with the PDB template sequences by MUSCLE v5.1. Structural visualization was conducted by PyMOL v2.5.4. Custom codes are available at https://github.com/FluSysBio/WTD_SARS-CoV-2_Transmission and can also be accessed at https://doi.org/10.5281/zenodo.8010758.

For manuscripts utilizing custom algorithms or software that are central to the research but not yet described in published literature, software must be made available to editors and reviewers. We strongly encourage code deposition in a community repository (e.g. GitHub). See the Nature Portfolio guidelines for submitting code & software for further information.

Data

Policy information about availability of data

All manuscripts must include a data availability statement. This statement should provide the following information, where applicable:

- Accession codes, unique identifiers, or web links for publicly available datasets
- A description of any restrictions on data availability
- For clinical datasets or third party data, please ensure that the statement adheres to our policy

The genomic data of all SARS-CoV-2 viruses generated in this study have been submitted to GISAID (the Global Initiative on Sharing All Influenza Data). Supplementary Data 1 contains the corresponding GISAID accession numbers, strain names, and related descriptions for these sequences. In the case of human SARS-CoV-2 sequences, their associated strain names and GISAID accession numbers can be found in Supplementary Data 8. Additionally, other public data utilized in this study can be accessed from GISAID and GenBank, NCBI (National Center for Biotechnology Information). Additionally, the original data utilized for generating bar graphs and geospatial visualizations can be accessed in the Source Data file.

Human research participants

Policy information about studies involving human research participants and Sex and Gender in Research.

Reporting on sex and gender	The metadata submitted to the GISAID database includes information on sex and gender, in addition to the SARS-CoV-2 genomic sequences.
Population characteristics	Individuals tested positive for SARS-CoV-2.
Recruitment	The human swab samples used in this study were de-identified diagnostic samples. No targeted recruitment efforts were undertaken.
Ethics oversight	This study received approval from the University of Missouri Institutional Review Board (IRB) under protocol number #2025449.

Note that full information on the approval of the study protocol must also be provided in the manuscript.

Field-specific reporting

Please select the one below that is the best fit for your research. If you are not sure, read the appropriate sections before making your selection.

☐ Life sciences ☐ Behavioural & social sciences ☒ Ecological, evolutionary & environmental sciences

For a reference copy of the document with all sections, see nature.com/documents/nr-reporting-summary-flat.pdf

Life sciences study design

All studies must disclose on these points even when the disclosure is negative.

Sample size	Not applicable
Data exclusions	Not applicable
Replication	Not applicable
Randomization	Not applicable
Blinding	Not applicable

Behavioural & social sciences study design

All studies must disclose on these points even when the disclosure is negative.

Study description	Not applicable
Research sample	Not applicable
Sampling strategy	Not applicable
Data collection	Not applicable

Timing	Not applicable
Data exclusions	Not applicable
Non-participation	Not applicable
Randomization	Not applicable

Ecological, evolutionary & environmental sciences study design

All studies must disclose on these points even when the disclosure is negative.

Study description

In this study, we performed a large-scale surveillance for SARS-CoV-2 across free-ranging white-tailed deer populations in the United States. Our objectives were to understand the genetic diversity of SARS-CoV-2 in free-ranging white-tailed deer, to evaluate whether the virus circulated within the white-tailed deer populations, and to assess transmission frequencies associated with zoonotic infections. The experiments conducted in this study did not involve treatment factors, interactions, or specific design structures such as factorial, nested, or hierarchical designs. Additionally, the nature and number of experimental units and replicates were not taken into consideration for the purposes of this study.

Research sample

In this study, we carried out a comprehensive sampling effort between November 4, 2021, and April 4, 2022 and collected 8,830 nasal swab samples from white-tailed deer in Washington DC and 26 states across the Northeast, Midwest, and Southeastern regions of the United States. These regions are home to approximately 95% of the white-tailed deer population in the country. Moreover, these areas align with the primary hunting grounds where hunters annually harvest around 6 million white-tailed deer. In the majority of cases, a paired blood sample was collected on a Nobuto filter. The nasal swab samples containing high-quality RNA were utilized for genomic sequencing, while the serum samples were employed for serological analyses.

To compare intra-host intra-host single nucleotide variations between human and white-tailed deer SARS-CoV-2 viruses, the de-identified human samples testing positive for SARS-CoV-2 were selected from a cohort of routinely collected samples at University of Missouri Health Care which covers the Columbia, MO and the neighboring counties. For this study, we specifically identified and included all human samples (n=148) that belonged to the same lineages (AY.103, AY.119, and AY.44) as those identified in the white-tailed deer during the corresponding sampling period.

In addition to the aforementioned data sources, we expanded our dataset by incorporating additional SARS-CoV-2 genomic sequences sourced from publicly available databases, namely GISAID (the Global Initiative on Sharing All Influenza Data) and NCBI (National Center for Biotechnology Information).

Sampling strategy

The collection of white-tailed deer swab samples was opportunistic and carried out by hunters and USDA harvest agents, and the human samples were from SARS-CoV-2 positive patients. As no sample size calculation was conducted, our analyses incorporated all collected white-tailed deer swab samples as well as the human samples that exhibited viruses belonging to the same lineages (AY.103, AY.119, and AY.44) identified in the white-tailed deer during the corresponding sampling period.

Data collection

SARS-CoV-2 RNA was prepared from oral or nasal swab samples preserved in PrimeStore Molecular Transport Media (MTM, Longhorn Vaccines and Diagnostics LLC) using MagMAX™ CORE Nucleic Acid Purification Kits (Applied Biosystems) in accordance with the manufacturers' instructions. 5 µL of RNA extract was for qRT-PCR detection of SARS-CoV-2 N1 and N2 targets using the BioRad Reliance One-Step Supermix Kit with SARS-CoV-2 RUO Primers & Probes obtained from Integrated DNA Technologies. Reaction and thermocycling conditions were identical to those described for the BioRad Reliance SARS-CoV-2 RT-PCR assay, and data was acquired BioRad CFX96 Touch Real-Time PCR Detection System or CFX Opus Real-Time PCR System. The qRT-PCR screen was performed by National Wildlife Research Center (NWRC) and further validated by the National Veterinary Services Laboratory (NVSL).

Virus genomes were obtained utilizing the Illumina MiSeq sequencing platform by NVSL.

Antibodies were extracted from Nobuto filter paper strips and screened at a functional dilution of 1:20 by using a surrogate virus neutralization test (sVNT, Genscript cPass™) with data acquired using a VarioSkan Flash or Varioskan LUX multimode microplate reader (Thermo Fisher). The serological assays were performed by NWRC.

Timing and spatial scale

The white-tailed deer samples were collected between November 4, 2021 to April 4, 2022, when overlapped the hunting season, from Washington DC and 26 states, including Arkansas, Connecticut, Delaware, Illinois, Kansas, Kentucky, Louisiana, Maine, Maryland, Massachusetts, Michigan, Minnesota, Missouri, New Jersey, New York, North Carolina, North Dakota, Oklahoma, Pennsylvania, South Carolina, South Dakota, Tennessee, Texas, Virginia, West Virginia, and Wisconsin. Throughout this timeframe, in the human population, there was an initial predominance of the Delta variant of SARS-CoV-2, which was subsequently replaced by the Omicron variant, and the Alpha and Gamma variants were subordinate in prevalence.

The de-identified human samples testing positive for SARS-CoV-2 were selected from a cohort of routinely collected samples at University of Missouri Health Care which covers the Columbia, MO and the neighboring counties. For this study, we specifically identified and included all human samples that belonged to the same lineages (AY.103, AY.119, and AY.44) as those identified in the white-tailed deer during the corresponding sampling period. These human samples we selected spanned from May 23, 2021 to February 10, 2022.

Data exclusions

No data were excluded in our analyses.

Reproducibility

For the qRT-PCR experiments, all samples underwent screening for both SARS-CoV-2 N1 and N2 targets. In cases where a sample was sent to the National Veterinary Services Laboratory (NVSL) for further analysis, an additional technical replication of the N1 target

	was performed. The results obtained from this second technical replication of the SARS-CoV-2 N1 rPCR showed 100% agreement with the original result. The accuracy of these analyses is bolstered by the NGS (Next-Generation Sequencing) and Sanger sequencing results provided by NVSL. Essentially, when we reported the presence of the virus, NVSL independently confirmed its presence through their own comprehensive analyses.<br><br>For genomic analyses, we used two pipelines (IRMA and CLC workbench) to evaluate the consensus sequence.<br><br>For serological assays, two technical replicates of sVNT (serum neutralization test) were conducted on each sample. In the event of any discrepancy between the analytical results obtained from these replicates, we performed additional retesting as needed to address the discrepancy. It is worth noting that only a limited number of samples required evaluation for a third time to resolve any issues, and an even smaller subset of samples underwent a fourth technical replicate.
Randomization	Not applicable. This is a genetic and observational study.
Blinding	Not applicable. This is a genetic and observational study.

Did the study involve field work? ☒ Yes ☐ No

Field work, collection and transport

Field conditions	This study did not incorporate the evaluation of environmental conditions.
Location	The latitude and longitude information for each white-tailed deer (WTD) sample is unavailable. Instead, the WTD samples were recorded based on the collection states and counties, as documented in Supplementary Data 1.
Access & import/export	Sample collection was opportunistic and conducted in cooperation with regulatory agencies and as part of routine surveillance programs.
Disturbance	No disturbance was caused by the study.

Reporting for specific materials, systems and methods

We require information from authors about some types of materials, experimental systems and methods used in many studies. Here, indicate whether each material, system or method listed is relevant to your study. If you are not sure if a list item applies to your research, read the appropriate section before selecting a response.

Materials & experimental systems

n/a	Involved in the study	
☒	☐	Antibodies
☒	☐	Eukaryotic cell lines
☒	☐	Palaeontology and archaeology
☐	☒	Animals and other organisms
☒	☐	Clinical data
☒	☐	Dual use research of concern

Methods

n/a	Involved in the study	
☒	☐	ChIP-seq
☒	☐	Flow cytometry
☒	☐	MRI-based neuroimaging

Antibodies

Antibodies used	Not applicable
Validation	Not applicable

Eukaryotic cell lines

Policy information about cell lines and Sex and Gender in Research

Cell line source(s)	Not applicable
Authentication	Not applicable
Mycoplasma contamination	Not applicable
Commonly misidentified lines (See ICLAC register)	Not applicable

Palaeontology and Archaeology

Specimen provenance	Not applicable
Specimen deposition	Not applicable
Dating methods	Not applicable

- [ ] Tick this box to confirm that the raw and calibrated dates are available in the paper or in Supplementary Information.

Ethics oversight	Not applicable

Note that full information on the approval of the study protocol must also be provided in the manuscript.

Animals and other research organisms

Policy information about studies involving animals; ARRIVE guidelines recommended for reporting animal research, and Sex and Gender in Research

Laboratory animals	Not applicable. No laboratory animals were used.
Wild animals	The swab and serum samples used in this study were collected from wild free-ranging. Sample collection was opportunistic and occurred posthumously.
Reporting on sex	This study focuses on viral evolution and transmission and did not include an analysis of host gender/sex.
Field-collected samples	Harvested deer carcasses were transported to related central processing points where samples were collected. WTD nasal swab samples were collected and samples preserved in PrimeStore Molecular Transport Media (MTM, Longhorn Vaccines and Diagnostics LLC) using MagMAX™ CORE Nucleic Acid Purification Kits (Applied Biosystems) in accordance with the manufacturers' instructions.
Ethics oversight	White-tailed deer were captured under a wildlife damage management agreement administered by USDA/APHIS Wildlife Services.

Note that full information on the approval of the study protocol must also be provided in the manuscript.

Clinical data

Policy information about clinical studies

All manuscripts should comply with the ICMJEguidelines for publication of clinical research and a completedCONSORT checklist must be included with all submissions.

Clinical trial registration	Not applicable
Study protocol	Not applicable
Data collection	Not applicable
Outcomes	Not applicable

Dual use research of concern

Policy information about dual use research of concern

Hazards

Could the accidental, deliberate or reckless misuse of agents or technologies generated in the work, or the application of information presented in the manuscript, pose a threat to:

No	Yes	
☒	☐	Public health
☒	☐	National security
☒	☐	Crops and/or livestock
☒	☐	Ecosystems
☒	☐	Any other significant area

Experiments of concern

Does the work involve any of these experiments of concern:

No	Yes	
☒	☐	Demonstrate how to render a vaccine ineffective
☒	☐	Confer resistance to therapeutically useful antibiotics or antiviral agents
☒	☐	Enhance the virulence of a pathogen or render a nonpathogen virulent
☒	☐	Increase transmissibility of a pathogen
☒	☐	Alter the host range of a pathogen
☒	☐	Enable evasion of diagnostic/detection modalities
☒	☐	Enable the weaponization of a biological agent or toxin
☒	☐	Any other potentially harmful combination of experiments and agents

ChIP-seq

Data deposition

☐ Confirm that both raw and final processed data have been deposited in a public database such as GEO.

☐ Confirm that you have deposited or provided access to graph files (e.g. BED files) for the called peaks.

Data access links<br>*May remain private before publication.*	Not applicable
Files in database submission	Not applicable
Genome browser session<br>(e.g. UCSC)	Not applicable

Methodology

Replicates	Not applicable
Sequencing depth	Not applicable
Antibodies	Not applicable
Peak calling parameters	Not applicable
Data quality	Not applicable
Software	Not applicable

Flow Cytometry

Plots

Confirm that:

☐ The axis labels state the marker and fluorochrome used (e.g. CD4-FITC).

☐ The axis scales are clearly visible. Include numbers along axes only for bottom left plot of group (a 'group' is an analysis of identical markers).

☐ All plots are contour plots with outliers or pseudocolor plots.

☐ A numerical value for number of cells or percentage (with statistics) is provided.

Methodology

Sample preparation	Not applicable
Instrument	Not applicable
Software	Not applicable
Cell population abundance	Not applicable
Gating strategy	Not applicable

☐ Tick this box to confirm that a figure exemplifying the gating strategy is provided in the Supplementary Information.

Magnetic resonance imaging

Experimental design

Design type: Not applicable

Design specifications: Not applicable

Behavioral performance measures: Not applicable

Acquisition

Imaging type(s): Not applicable

Field strength: Not applicable

Sequence & imaging parameters: Not applicable

Area of acquisition: Not applicable

Diffusion MRI: ☐ Used ☒ Not used

Preprocessing

Preprocessing software: Not applicable

Normalization: Not applicable

Normalization template: Not applicable

Noise and artifact removal: Not applicable

Volume censoring: Not applicable

Statistical modeling & inference

Model type and settings: Not applicable

Effect(s) tested: Not applicable

Specify type of analysis: ☐ Whole brain ☐ ROI-based ☐ Both

Statistic type for inference (See Eklund et al. 2016): Not applicable

Correction: Not applicable

Models & analysis

n/a	Involved in the study
☒	☐ Functional and/or effective connectivity
☒	☐ Graph analysis
☒	☐ Multivariate modeling or predictive analysis
